# The Zagros Epipalaeolithic revisited: New excavations and ^14^C dates from Palegawra cave in Iraqi Kurdistan

**DOI:** 10.1371/journal.pone.0239564

**Published:** 2020-09-21

**Authors:** Eleni Asouti, Douglas Baird, Ceren Kabukcu, Kate Swinson, Louise Martin, Aroa García-Suárez, Emma Jenkins, Kamal Rasheed

**Affiliations:** 1 Department of Archaeology, Classics and Egyptology, University of Liverpool, Liverpool, United Kingdom; 2 Institute of Archaeology, University College London, London, United Kingdom; 3 Department of Archaeology, University of Reading, Reading, United Kingdom; 4 Department of Archaeology & Anthropology, Bournemouth University, Poole, United Kingdom; 5 Sulaymaniyah Directorate of Antiquities and Heritage, Kurdistan Region, Iraq; University at Buffalo - The State University of New York, UNITED STATES

## Abstract

Palegawra cave, alongside its neighbouring Zarzi, has been an emblematic site of the Epipalaeolithic (Zarzian) cultural horizon in the NW Zagros of Southwest Asia ever since its first exploration in 1951 by Bruce Howe and Robert Braidwood in the context of the Iraq-Jarmo project. At the time scientific excavation, sampling and analysis methods were either under-developed or did not exist. In this paper we present the first results of new excavations at Palegawra conducted in 2016–2017 by the Eastern Fertile Crescent (EFEC) project, a research collaboration of the University of Liverpool and the Sulaymaniyah Directorate of Antiquities and Heritage. Our research has produced the first radiometric evidence pushing back the chronology of the NW Zagros Epipalaeolithic to the Last Glacial Maximum, thus fully aligning it with Epipalaeolithic facies until now known only from the Levant and the south Anatolian coast. We have also unearthed, for the first time in the Palaeolithic of the Zagros, direct archaeobotanical evidence for hitherto elusive Zarzian plant exploitation and the vegetation of the NW Zagros piedmont zone from the LGM to the end of the Lateglacial (~19,600–13,000 cal BP). The new Palegawra chronology alongside our detailed studies of its material culture and faunal and botanical assemblages suggest that the prevailing Epipalaeolithic habitation pattern in the NW Zagros (centred on generalised persistent occupations of small caves and rock-shelters alongside task-oriented ephemeral open-air campsites) remained an enduring characteristic of the Zarzian horizon throughout this period. The Palegawra data clearly show that neither resource levels and climate conditions nor geographic and/or cultural isolation provide adequate explanations for the stability and longevity of Zarzian lifeways during this long timespan. More fieldwork is required, including the discovery, excavation and intensive sampling of other Zarzian sites, for reaching a data-informed understanding of the nature and evolution of the NW Zagros Epipalaeolithic.

## Introduction

**Fig 1:**
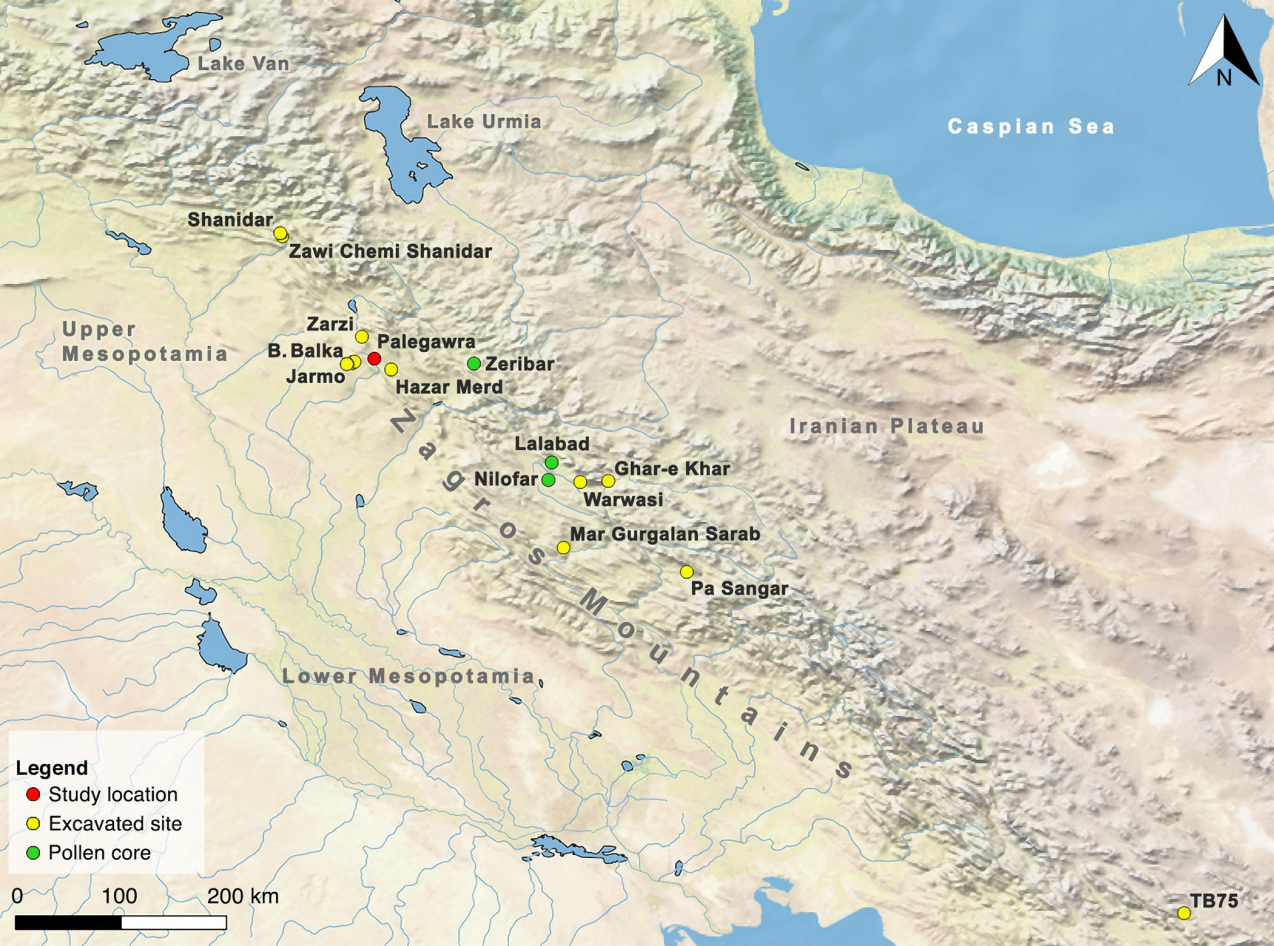
Map showing the location of sites mentioned in the text (map by C Kabukcu). Map created using QGIS 3.10.7 (free and open source) with Natural Earth (free vector and raster map data @ naturalearthdata.com).

**Fig 2:**
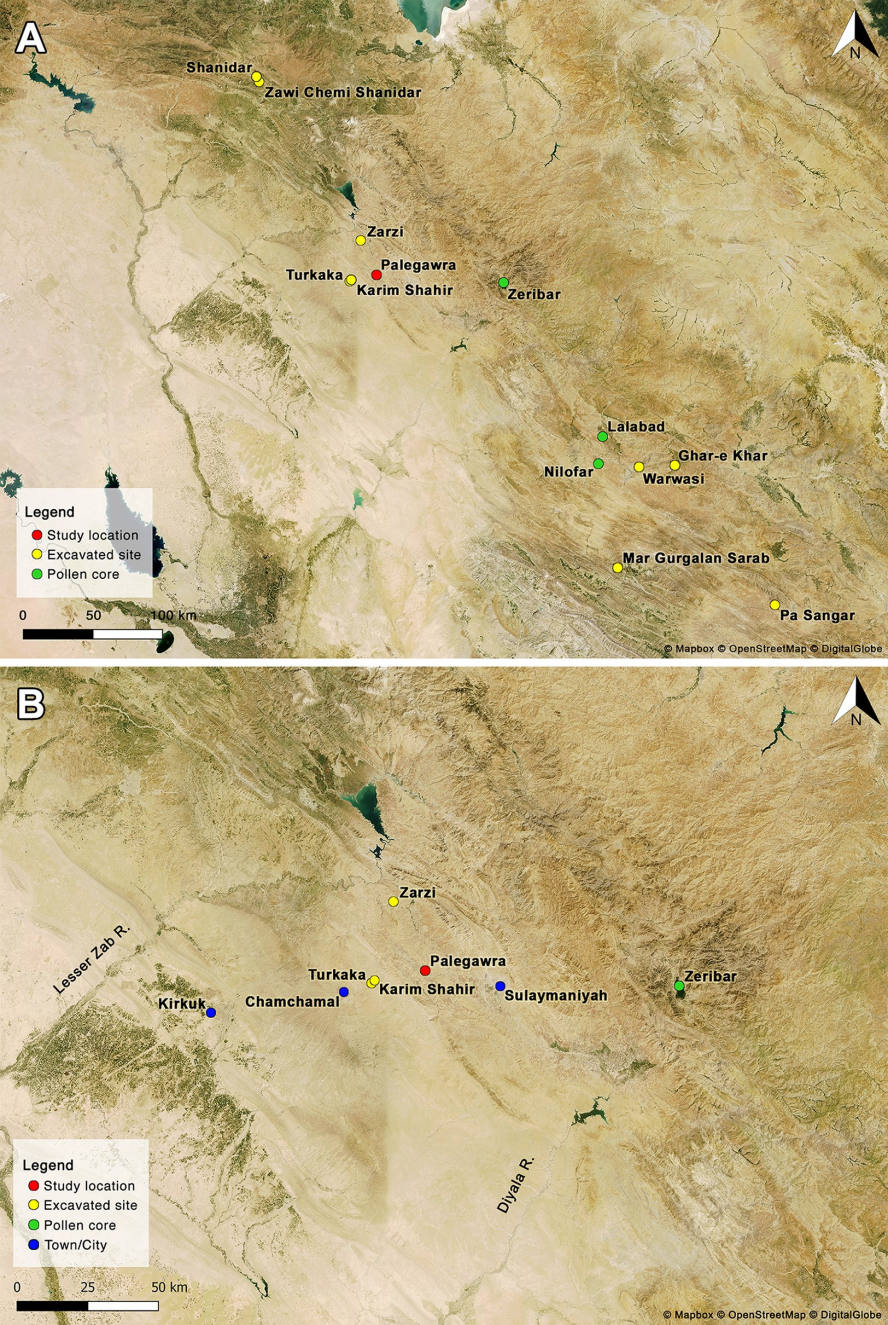
Satellite maps showing the location of late Palaeolithic sites in the NW and central Zagros (maps by C Kabukcu). Map created using QGIS 3.10.7 (free and open source) with tilesets downloaded from Mapbox. Satellite basemap used by permission under a CC BY license. Copyright © 2020 Mapbox. (A) NW and central Zagros; (B) Sulaymaniyah district area in Iraqi Kurdistan.

The Kurdistan region of northern Iraq occupies culturally, geographically and ecologically a central position in the Fertile Crescent of Southwest Asia as a natural bridge linking its eastern and western arcs. It thus represents a critical location for exploring the Palaeolithic antecedents of the Neolithic revolution and the regional environmental history during the Pleistocene-Holocene transition (~20,000–12,000 cal BP). However, due to recurrent cycles of political instability since the late 1950s, the local Palaeolithic archaeology remains critically under-explored compared to the volume of fieldwork conducted elsewhere in SW Asia (notably the Levant) despite the very prominent position of Iraqi Kurdistan in the history of Eurasian Palaeolithic research. The first Palaeolithic excavations in the Middle East were conducted in 1928 at the Kurdish cave sites of Zarzi and Hazar Merd in the Sulaymaniyah province by the British prehistorian Dorothy Garrod [1] (Figs 1 and 2). The ‘hilly flanks’ of the NW Zagros was also where problem-oriented fieldwork, aimed at investigating the origin and evolution of the earliest farming economies and their antecedents, took place for the first time in the late 1940s and 1950s by Robert and Linda Braidwood of the University of Chicago Oriental Institute, and Bruce Howe of Peabody Museum and the American Schools of Oriental Research. The Iraq-Jarmo project excavated the sites of Jarmo, Karim Shahir, Palegawra and Barda Balka in Sulaymaniyah, alongside surveying and test-trenching select sites in the Erbil province [2–4]. In the 1950s and early 1960s two other American archaeologists, Ralph and Rose Solecki of Columbia University, excavated Shanidar cave in Erbil uncovering successive Palaeolithic habitations including the first Neanderthal remains in Iraq and the neighbouring ‘Proto-Neolithic’ open-air site of Zawi Chemi Shanidar [5–7].

A significant shortcoming of these early field projects, of which Robert Braidwood had become acutely aware in later years ([8]: p.67) was the unavailability at the time of the scientific sampling and recording techniques that are used today for excavating prehistoric sites. The Braidwoods certainly appreciated the importance of studying botanical and faunal remains and of palaeoenvironmental reconstruction for prehistoric research. However, the field methods they had at their disposal were, by present-day standards, minimally developed. Dry sieving of excavated sediments was applied selectively; the use of water flotation for the recovery of archaeobiological remains from excavated sediments and what constitutes representative sampling in the field for archaeobotanical and zooarchaeological analyses, were also unknown ([2]: p.2). Furthermore, appropriate field sampling, recording and quality control protocols for collecting organic materials for radiocarbon dating were not applied because the impact of these factors on the reliability of radiocarbon dating was still unknown or poorly understood ([3]: p.541).

This paper presents the first results of the Eastern Fertile Crescent (EFEC) project, a research collaboration of the University of Liverpool and the Sulaymaniyah Directorate of Antiquities and Heritage directed by Eleni Asouti, Douglas Baird and Kamal Rasheed. The EFEC project focuses on the period between ~20,000–12,000 cal BP represented in the Zagros by the Epipalaeolithic Zarzian cultural horizon. Our main aim is to investigate the chronology, habitation patterns and landscape exploitation practices (including the use of biotic resources and raw materials) characterizing the Zarzian communities of northern Iraq, and how they compare with contemporaneous cultural entities known from the Iranian central and southern Zagros, and the Levant. During the first phase of the EFEC project (2014–2017) we surveyed the open-air sites of Karim Shahir and Turkaka and re-excavated Karim Shahir and Palegawra cave, both previously dug by Bruce Howe for the Iraq-Jarmo project. Our key research objectives were (i) to retrieve representative samples of material culture alongside faunal and (for the first time in the Palaeolithic of the Zagros) macrobotanical remains, and (ii) to obtain new reliable radiocarbon dates that would permit placing the results of our studies in a robust chronological framework and draw comparisons with Epipalaeolithic entities known from other SW Asian regions.

Even a cursory overview of the older and more recent literature on the Epipalaeolithic of northern Iraq suffices to demonstrate the ambiguity that still surrounds its chronology and habitation patterns, and the paucity of the available evidence regarding people-environment interactions and the impacts of climate change. The brief outline of the history of research on the Epipalaeolithic of the Zagros that follows in the next section of the paper highlights two particular areas of long-standing controversy and debate: (i) the degree to which the variation observed in Zarzian chipped stone assemblages can be attributed to chrono-typological and/or functional factors, and (ii) the chronology and duration of the Zarzian horizon. Specifically, whether it represents a long-lived cultural horizon organically connected to later ‘Proto-Neolithic’ and earlier Upper Palaeolithic (Baradostian) traditions; or, if there was instead a significant gap (~15,000 years according to some estimates) from the end of the Baradostian to the onset of the Zarzian, attributed to palaeoclimatic conditions during the LGM that were extremely adverse for human habitation. The main part of the paper describes and discusses the results of the 2016–2017 EFEC project excavations at Palegawra, set in the context of the limited materials published by the Iraq-Jarmo project and those available from other sites in the Zagros and, where appropriate, other regions of SW Asia. We present in turn the cave stratigraphy and chronology, followed by the results of our studies of the material culture (lithics, personal ornaments and ground stone) and faunal and botanical assemblages. The closing sections of the paper discuss the implications of the first results of the EFEC project for a novel, data-informed understanding of the nature of the Zarzian horizon in the NW Zagros and identify goals for future problem-oriented research in the Zagros Epipalaeolithic.

## The Zagros Epipalaeolithic: Current state of research

The first Palaeolithic excavations in Iraq were conducted by Dorothy Garrod between 3–11 November 1928 at Zarzi cave in the valley of Cham Tabin, 50km NW of Sulaymaniyah, followed by an equally short period of digging (20 November-6 December 1928) at the ‘Dark Cave’ (Ashkawty Tarik) of the Hazar Merd cave complex on the east-facing ridge of Baranand Dagh overlooking the Khanjiru Chai, 8km SW of Sulaymaniyah [1] (Figs 1 and 2). The Hazar Merd sequence comprised Middle Palaeolithic lithic industries (Garrod’s designated Mousterian Layer C) superimposed at variable depths by an Upper Palaeolithic industry (Layer B). At Zarzi, Garrod unearthed in Layer B (extending out from the cave on the terrace and down the slope at a depth 0.50–1.50m) what she originally identified as a late Upper Palaeolithic industry, which later became widely known as the ‘Zarzian’. Garrod tentatively subdivided this industry into ‘early’ and ‘late’ phases based on what she identified as increasing proportions and types of geometric microliths through time. This scheme was further elaborated by Braidwood and Howe based on their observations at Palegawra ([2]: pp.57-59). In later years this chronological distinction was qualified by the Iraqi prehistorian Ghanim Wahida. Upon re-excavating Layer B at Zarzi in 1971 and studying its chipped stone assemblage by comparison to lithic collections from other sites Wahida concluded that the Zarzian should be viewed instead as a unified late Upper Palaeolithic industry, with the chronological distinctions probably present at Zarzi (based on the frequency of geometrics) and other variations observed in assemblage composition between Zarzian sites being better attributed to functional and ecological rather than chronological factors [9–11].

**Fig 3:**
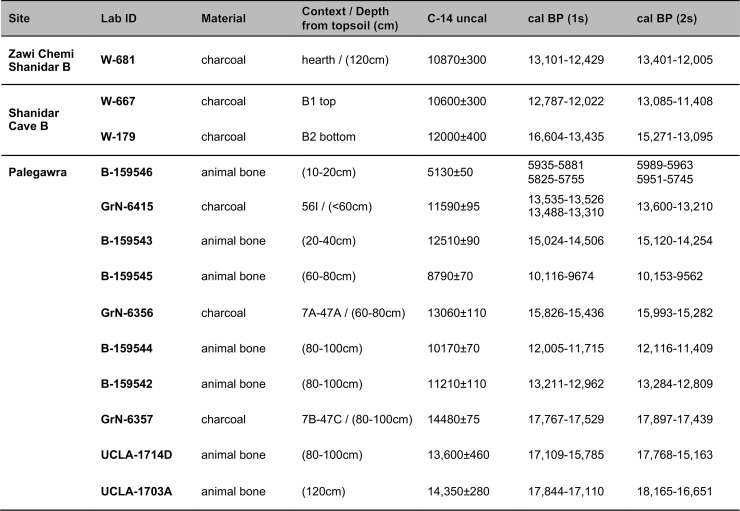
Radiocarbon dates from Zawi Chemi Shanidar Layer B, Shanidar cave (Layers B1 and B2), and the Palegawra Iraq-Jarmo project excavations. All dates re-calibrated using OxCal v4.3.2 [18] and the IntCal13 atmospheric curve [19]. Radiocarbon age determination sources: Zawi Chemi Shanidar B [20, 21]; Shanidar Cave B [22, 23]; Palegawra (Iraq-Jarmo project) [16, 17, 24].

The next major phase of prehistoric fieldwork took place in the 1950s and 1960s, in the context of the Iraq-Jarmo project, by Robert and Linda Braidwood and Bruce Howe. Between 1950–55 the Iraq-Jarmo team excavated 4 sites including Palegawra cave (Epipalaeolithic), and the open-air sites of Barda Balka (Lower Palaeolithic), Karim Shahir (‘Proto-Neolithic’) and Jarmo (Neolithic) [2–4, 12–15]. Of the excavated Palaeolithic sites only Palegawra has been previously radiocarbon dated to ~17,500–13,400 cal BP [16]. This range (obtained from charcoal samples) was subsequently narrowed down by Melinda Zeder to ~15,000–12,000 cal BP using AMS dates obtained from animal bone collagen samples [17] (Fig 3). Other possible Epipalaeolithic open-air sites surveyed in 1951 by the Iraq-Jarmo project in the Chamchamal area included Turkaka and Kowri Khan, both of which produced Zarzian-like surface lithic collections. The apparent absence of geometric microliths from these sites suggested that they might predate Zarzi and Palegawra. However, Braidwood and Howe also entertained the possibility that their lithic assemblages might reflect functional variants, attributed to seasonal and/or task-specific occupations, and concluded that these ambiguities could only be resolved through future excavation and radiometric dating ([2]: pp.55-56).

Karim Shahir (undated) comprised an artificial rock and pebble scatter interspersed with hearths, pits and occupation waste. Howe considered the site a seasonal encampment. He identified the closest parallels to its chipped stone industry in the ‘Proto-Neolithic’ industries of Shanidar cave (Layer B1) and Zawi Chemi Shanidar (Layer B) ([3]: p.114; see also [5, 6]). Ralph Solecki’s excavations at Shanidar cave revealed a series of deposits including Layer B, in which he identified both Zarzian (B2) and ‘Proto-Neolithic’ (B1) facies, preceded by Upper (Baradostian Layer C) and Middle Palaeolithic (Mousterian Layer D) strata. B2 was noted for its higher concentration of microliths and the absence of ground stone. Its lithic industry was comparable to that of Palegawra as reported by Howe, including the ubiquity of geometric microliths and backed blades. By contrast, the material culture assemblage of the stratigraphically later B1 presented close affinities with the purportedly ‘Proto-Neolithic’ Layer B at Zawi Chemi including lithics, worked bone and various ground stone implements such as querns, mortars, hand stones and grooved stones, fragments of matting or basketry, and sporadic evidence for the use of obsidian and (possibly) bitumen too. Solecki also discovered a burial site towards the back of the Shanidar cave chamber comprising 26 skeletons, stone platforms and an arc-like alignment of flat stones. Although he reported the burials as stratigraphically associated with B1 [7] it seems more probable that they were cut into the main B1 deposits [25]. A single radiocarbon determination from the bottom of Shanidar B2 placed the onset of its deposition at ~14,000 cal BP (possibly earlier) while the top part of B1 was dated at ~12,400 cal BP (although the range could extend as late as 11,500 cal BP). At Zawi Chemi a single charcoal sample from a depth of ~1.20m near the base of Layer B produced a date of ~12,800 cal BP (possibly as late as 12,000 cal BP) (see also Fig 3). These dates are unreliable due to their very large standard errors and the limited information available on the stratigraphic position and contextual associations of the dated samples. Ideally, new samples should be excavated and retrieved from deposits that may still be preserved at both sites, in order to obtain accurate estimates of the chronologies of these strata.

Solecki had claimed a hiatus of ~2000 years between the end of Shanidar B2 and the onset of B1. This he based on the differences observed in their lithic industries and material culture, and the apparent length of time that had elapsed between the onset of B2 and the end of B1 as indicated by the above-mentioned radiocarbon dates. A later re-examination of the Shanidar B1 and B2 industries by Wahida ([11]: p.200) found no major shifts in lithic typologies but, instead, evidence for technological continuity and local evolution. The only significant differences were discerned in the abundance of ground stone implements and personal ornaments in B1. The purported gap between B1 and B2 might thus represent at best sporadic episodes of seasonal habitation inside the cave (instead of its abandonment during the Lateglacial and its re-occupation at a later time) or, at worst, an artefact of the inherently flawed radiocarbon chronology and the stratigraphic ambiguities observed in B1 related to the intrusive burials [25]. Overall, the ambiguity plaguing the interpretation of the Shanidar B1-B2 sequence and of Zawi Chemi B means that on presently available evidence it is not possible to assess whether Shanidar B1 and Zawi Chemi B overlap, at least in part, with the terminal Pleistocene or they date mostly to the early Holocene as proposed by some authors [26].

**Fig 4:**
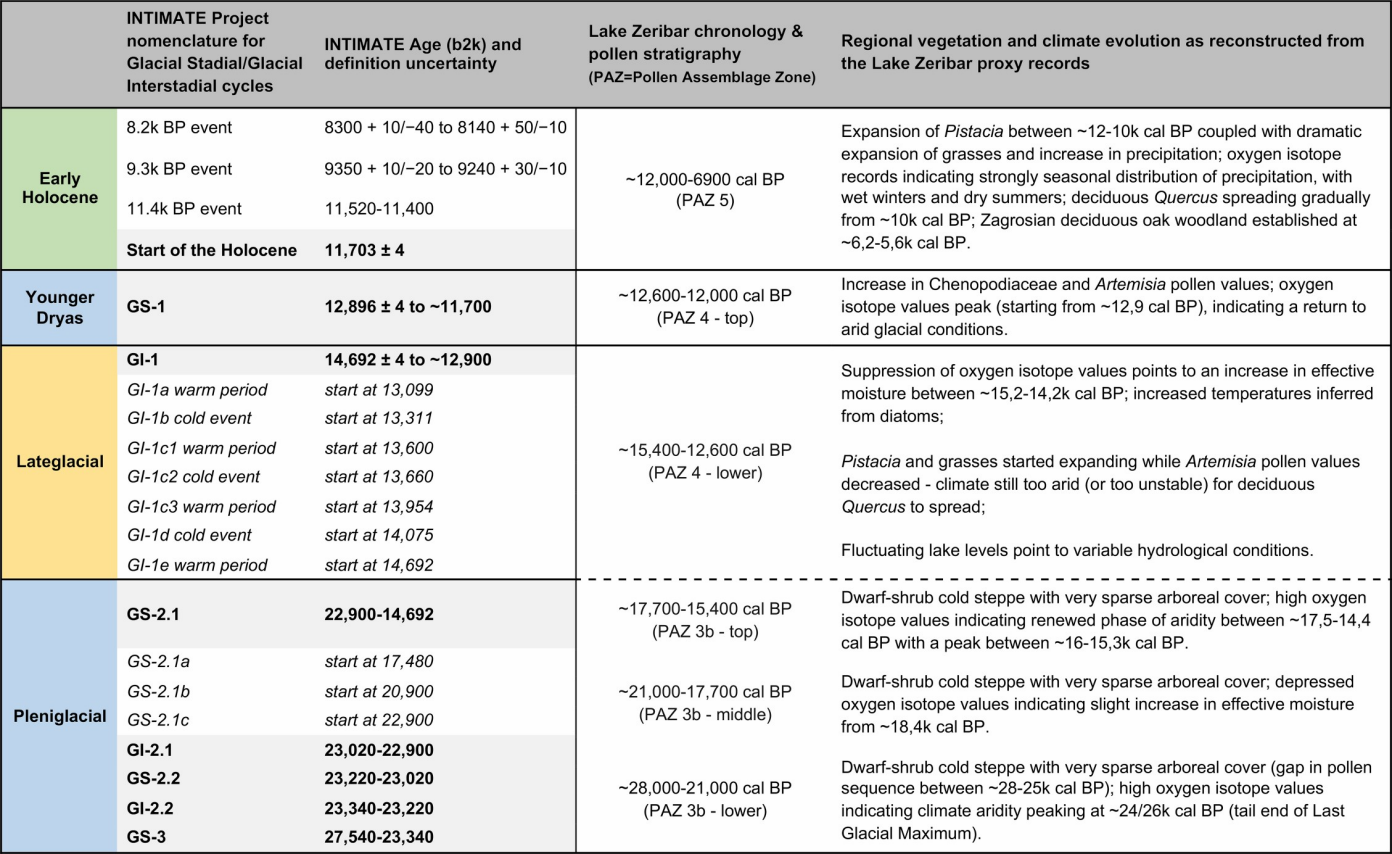
Correlation of the lake Zeribar (Iranian Zagros) proxy data with the INTIMATE project Greenland ice core chronostratigraphy. Data compiled from [29, 30].

If the dating of the ‘end’ of the Zarzian horizon in the northern Zagros is contested, the same applies to its beginning. The impression that the northern Zagros Epipalaeolithic was largely confined to the Lateglacial is widespread in the literature, due to the perceived inhospitality of the region for human habitation during the Last Glacial Maximum (LGM) [26, 27]. Two lines of evidence have been invoked in support this argument: (a) at Shanidar the ~20,000 year gap that separates the Baradostian Layer C, dated between ~42,000–35,000 cal BP [28] from the base of the Zarzian Layer B2, and (b) the late onset of the Zarzian at the two excavated type sites, Zarzi and Palegawra. Wahida’s 1971 re-excavation of Zarzi did not produce radiocarbon dates. However, he uncovered a more refined stratigraphic sequence comprising 5 superimposed geological strata (A-F from top to bottom) with no discernible hiatuses down to a depth of 2.45m ([10]: p.22). Pollen analysis by Leroi-Gourhan ([10]: pp.33-36) indicated that the onset of the Lateglacial climatic amelioration (dated at lake Zeribar ~14,400 cal BP) could be tentatively matched with the pollen assemblage of Layer D, which also contained the stratigraphically earliest significant concentration of Zarzian lithics (for a summary of the lake Zeribar palaeoenvironmental proxies compared to the Greenland ice core chronostratigraphy see Fig 4). No tree pollen was found in the Zarzi strata E-F, which contained very low densities of cultural materials. Thus, taking also into account the radiocarbon dates already available from Palegawra, the start of the Zarzian horizon in the NW Zagros has been considered to date sometime around 15,000 cal BP with some authors accepting a date as early as ~17,000 cal BP [26].

Other scholars researching the late Palaeolithic of the Iranian central Zagros have questioned the assumption of a long break between the late Upper Palaeolithic and the Zarzian. Evidence for stratigraphic and lithic techno-typological continuity between successive Baradostian (or ‘Zagros Aurignacian’) and Zarzian phases has been claimed for the Warwasi, Pa Sangar and Ghar-e Khar rock-shelters [25, 31–34] suggesting that the Zarzian horizon may extend back to >20,000 cal BP. This proposition has been received with caution due to the continuing lack of radiometric chronologies from Zarzian sites in both the NW and the central Zagros [27]. However, radiocarbon dates more recently obtained from the Eshkaft-e Haji Bahrami cave (TB75) by the joint Iran-Japan expedition at Tang-e Bolaghi (Fars province) place its Epipalaeolithic layers between ~20,000–14,000 cal BP [35, 36]. It thus appears probable that, at least in the southern Zagros, the start of the Zarzian horizon dates back to the late Pleniglacial (GS-2.1).

## The study area

Palegawra cave (35°35'59.61"N, 45° 8'42.67"E; ~854m a.s.l.) is located on the NW Zagros piedmont zone, on the part of it that is delimited to the north by the Lesser Zab river, to the west by the city of Kirkuk, marking its boundary with the Mesopotamian upper plain (Jezireh), and to the south by the Diyala (Sirwan) river. The piedmont zone consists of a series of dissected hills and successive anticlinal limestone ridges that rise eastward towards the Zagros high folded zone, running parallel to it in a NW-SE direction. These ridges are separated by flat valleys forming the Chamchamal, Bazian and Sulaymaniyah plains (Fig 2 and S1–S3 Figs).

Climate data covering the last 80 years from the Bazian (829m a.s.l.) and Sulaymaniyah (885m a.s.l.) meteorological stations nearest to Palegawra, record annual precipitation averages of ~580-700mm. Precipitation is seasonally distributed, with ~75% falling between the months of November-March and ~50% between December-February. Temperature is also markedly seasonally distributed, with the lowest averages (6–8°C) recorded for the months of December-February and the highest (29–33°C) for June-August. The local climate is thus characterised as Mediterranean to continental being semi-humid to semi-arid in spring and autumn, humid and cold in winter, and distinctly dry and warm during the summer months [37, 38].

The NW Zagros piedmont zone forms part of the Irano-Anatolian subdivision of the Irano-Turanian phytogeographical region. Its vegetation has been described in detail by various sources [39–43]. It comprises two major formations: the dry (~300-500mm of annual rainfall) *Amygdalus-Pistacia* open woodland and scrub, extending from the eastern boundary of the Jezireh to an elevation of ~700m a.s.l., which partly overlaps with Guest’s ‘moist steppe’ zone, and the Kurdo-Zagrosian forest (coinciding with Guest’s ‘forest zone’) found at elevations of ~700-1800m a.s.l. receiving >500-1400mm of annual rainfall. At the lower end of its altitudinal range *Quercus brantii* woodland, where not obliterated by forest fires, cultivation, logging and charcoal making, occurs in association with *Pistacia atlantica* ssp. *kurdica* and *P*. *khinjuk*. These species can tolerate low rainfall (*Q*. *brantii* reportedly as low as 330mm p.a.) and temperatures, but they are intolerant of heavy snowfall and frost and normally fail to regenerate from seed under exceedingly warm and dry summer conditions [43]. Other constituents of the Kurdo-Zagrosian forest include *Rhamnus kurdica*, *Ficus carica*, *Pyrus syriaca*, *Crataegus azarolus*, *C*. *aronia*, *Celtis tournefortii*, *Amygdalus orientalis*, *A*. *scoparia*, *Cerasus microcarpa* and *Fraxinus rotundifolia*. At higher altitudes (>1200-1600m a.s.l.) and in sheltered habitats (e.g., ravine bottoms and slopes) *Quercus brantii* is replaced by the more mesic *Q*. *libani* and *Q*. *infectoria*; arboreal flora may also include *Acer cinerascens*, *Lonicera nummularia*, *Amygdalus eleagnifolia* and *Juniperus excelsa*. Riparian taxa such as *Populus euphratica*, *Salix* and *Tamarix* grow on alluvial plains along watercourses; at higher altitudes they may be accompanied by *Ulmus*, *Crataegus monogyna* and *Eleagnus angustifolia*.

## Materials and methods

**Fig 5:**
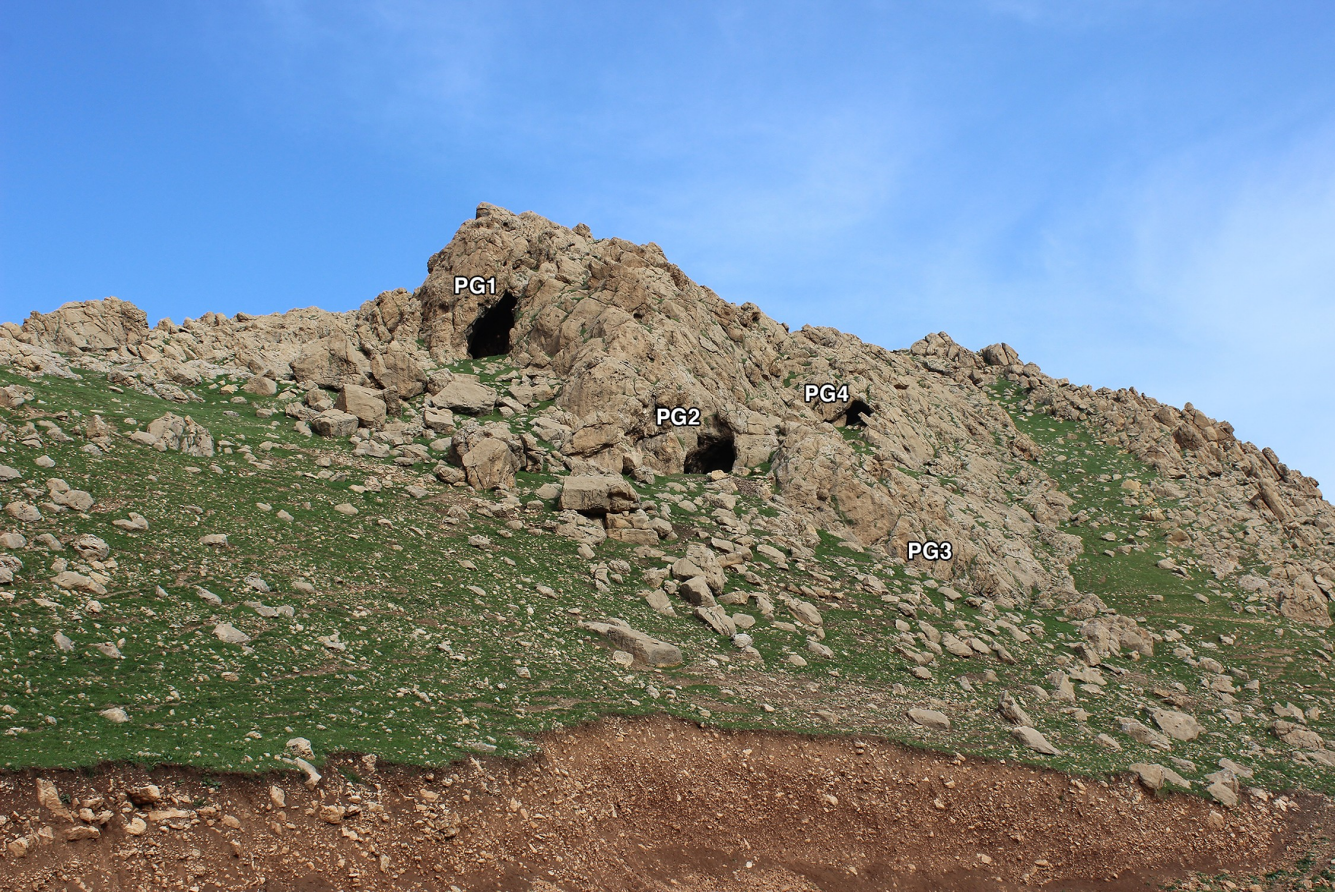
Present-day configuration of the Palegawra cave complex (photo by E Asouti).

The Palegawra (PG) cave complex consists of 3 caves located ~30m apart from each other along the NW-SE oriented line of the northern extension of the Baranand Dagh ridge flanking the NE edge of the Bazian valley (S1–S3 Figs). It is located on the western side of a small wadi discharging into the valley. A very shallow rock-shelter is located downslope to the NE of the caves. We have numbered these caves and the rock-shelter consecutively from S-N: the first two caves PG1, PG2, the rock-shelter PG3 and the northernmost cave PG4 (Fig 5).

**Fig 6:**
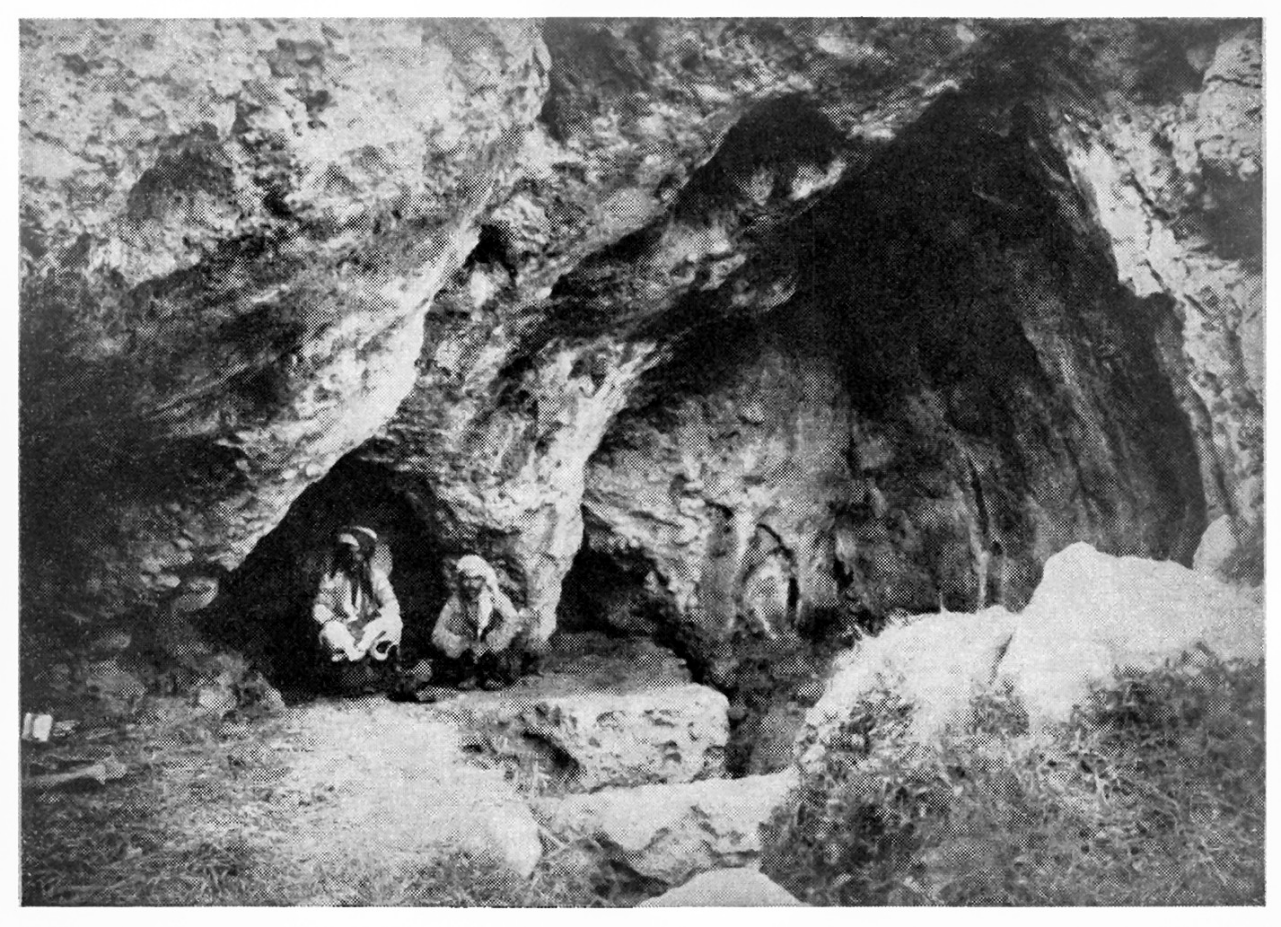
View from the cave entrance showing the location of Howe’s trench inside the PG chamber. Reproduced from [44] (p.1422) with the permission of the American Association for the Advancement of Science.

The Iraq-Jarmo project excavated in 1951 and 1955 the chamber of PG2 (henceforth denoted as PG) (Fig 6). An area of ~30m^2^ was excavated down to the bedrock, which was reached at a depth of ~2m near the eastern edge of the trench. The upper layer (~0.60m deep) contained heavily disturbed and mixed cultural materials dating from the Chalcolithic to the Islamic periods, also including some human burials and scattered bone. Howe located near the base of this upper layer occasional finds of possible Neolithic date including ground and knapped stone. Zarzian cultural materials were located mostly underneath this layer between ~0.60–1.30m.

**Fig 7:**
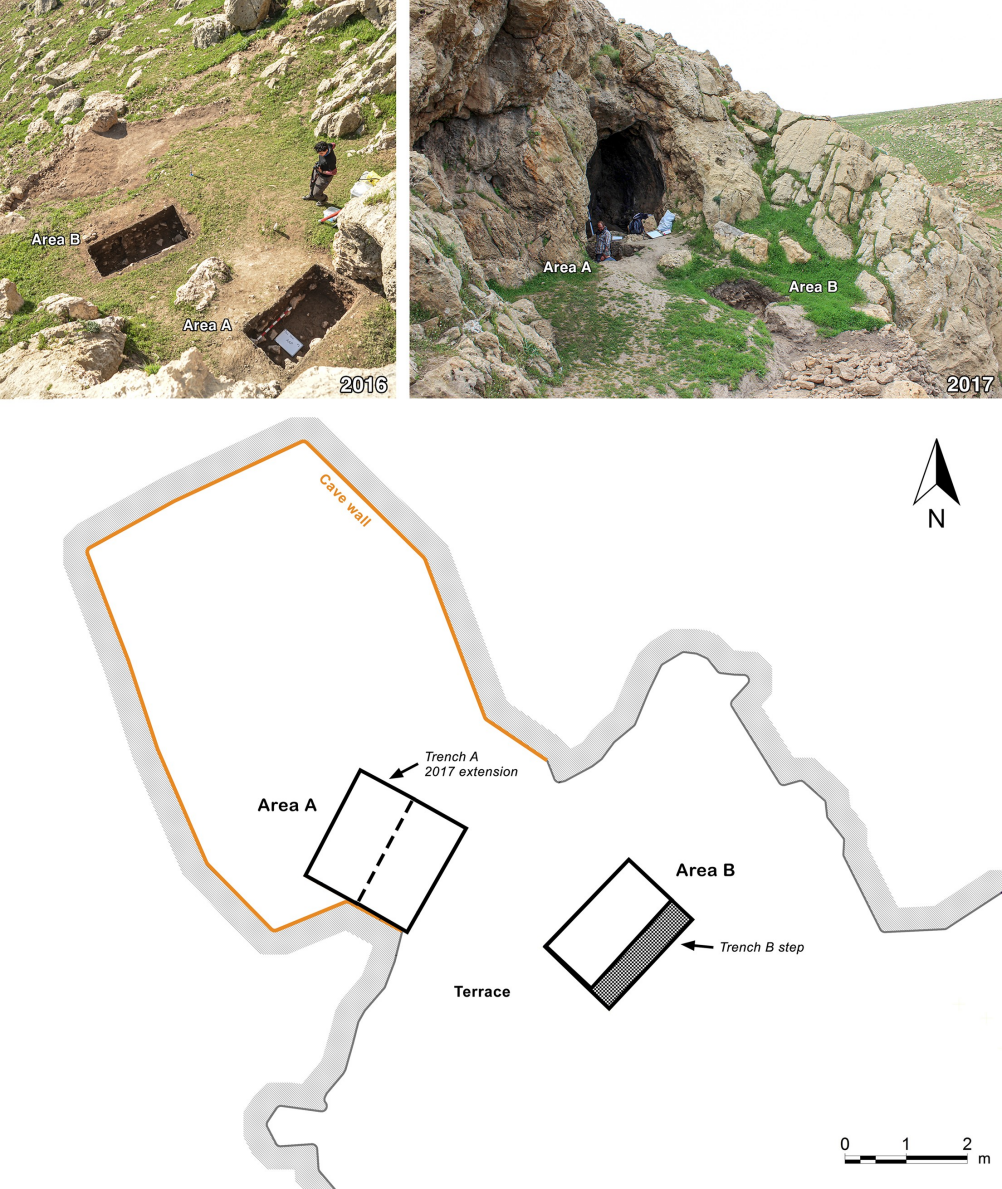
General views of the EFEC project trenches A and B as excavated in 2016 and 2017, and plan of the PG cave chamber and terrace showing the location of A and B as excavated in 2017. (2016: photo by A Amin; 2017: photo by E Asouti).

The EFEC project excavations in 2016 and 2017 targeted the threshold of PG and the terrace in front of it, which had been left unexcavated by Howe. Two 2 x 2m trenches were excavated (Fig 7). Area A is located on the southern side of the modern entrance to the cave (oriented SW-NE along the mouth of the cave) straddling the drip line of the entrance area. Area B (oriented N-S) is located to the east of A, on the terrace in front of the cave mouth. Its eastern half was only excavated through the topsoil to provide a ~1m-wide step for deeper excavation through the terrace slope. Both trenches were excavated to the bedrock, although the full terrace base was not exposed in Area B due to the high volume of rockfall debris.

There was very little variation in the colour and texture of the deposits excavated within the main late Pleistocene sequence. Excavation was thus conducted in a series of spits varying in thickness between ~0.05–0.10/15m. Where distinct deposits were encountered or there were indications of slope these contexts were excavated separately, and the slope was followed. All sediment contained in animal burrows and root action located within the Pleistocene sediments was removed separately from the main deposit, in order to reduce the risk of contamination by intrusive materials. This was a persistent problem for the Iraq-Jarmo project excavation as evidenced by the regular occurrence of potsherds down to the base of the Zarzian deposits dug in Howe’s trench ([2]: p.58).

All deposits below topsoil that were free of macroscopic bioturbation were processed with machine-assisted water flotation for recovering charred plant remains alongside debitage, other artefacts, animal bone and microfauna. In total we recovered 63 sediment samples from all excavated deposits amounting to 2652.95 litres of soil (S1 Table). These samples were processed with a high-capacity recycling water flotation system consisting of 1 flot tank and 2 settling tanks that was set up at the Sulaymaniyah Directorate of Antiquities and Heritage dighouse at the Bazian Pass near the town of Takiya, which was the base of the EFEC project during the 2016–2017 field seasons. The flot fractions were captured with a 250μm nylon mesh and the heavy residues containing small artefacts, debitage and animal bone were retained in a 500μm nylon mesh. Once dried, all heavy residue fractions were passed through a stack of geological sieves (apertures 4, 2 and 1mm plus retainer) in order to facilitate their sorting.

In Area A ~75% of the late Pleistocene deposits were processed by flotation at 100% (i.e., the entire excavated contexts were collected as flotation samples). The remaining 25% included mostly contexts excavated in the bioturbated upper part of the sequence and were dry sieved in the field with a locally procured ~5mm mesh sieve. ~50% of the Area B late Pleistocene sediments were processed by flotation while the remaining 50% were dry sieved. In addition, we collected 4 sediment block samples for micromorphological analysis from undisturbed Area A contexts, and a total of 71 soil samples from Areas A and B for phytolith analysis.

The field research leading to this paper was conducted in collaboration with the Sulaymaniyah Directorate of Antiquities and Heritage under a permit issued by the General Directorate of Antiquities in Iraqi Kurdistan. All the chipped stone, ground stone, worked bone and shell items reported in the following sections are held at the Sulaymaniyah Directorate of Antiquities and Heritage in Iraqi Kurdistan. Materials exported to the UK for further laboratory analyses with the permission of the Sulaymaniyah Directorate of Antiquities and Heritage include the following: the lithic items displayed in S8–S13 Figs, all the archaeobotanical flotation samples and the charred plant remains contained in these samples, and the micromorphological slides reported in this paper, which are held at the Department of Archaeology, Classics and Egyptology of the University of Liverpool; all phytolith soil samples and slides, which are held at the Department of Archaeology and Anthropology of Bournemouth University; all diagnostic animal bone specimens reported in this paper, which are held at the Institute of Archaeology of University College London. All the above-mentioned materials are accessible upon request.

## Results

### The PG stratigraphy

#### Area A

**Fig 8:**
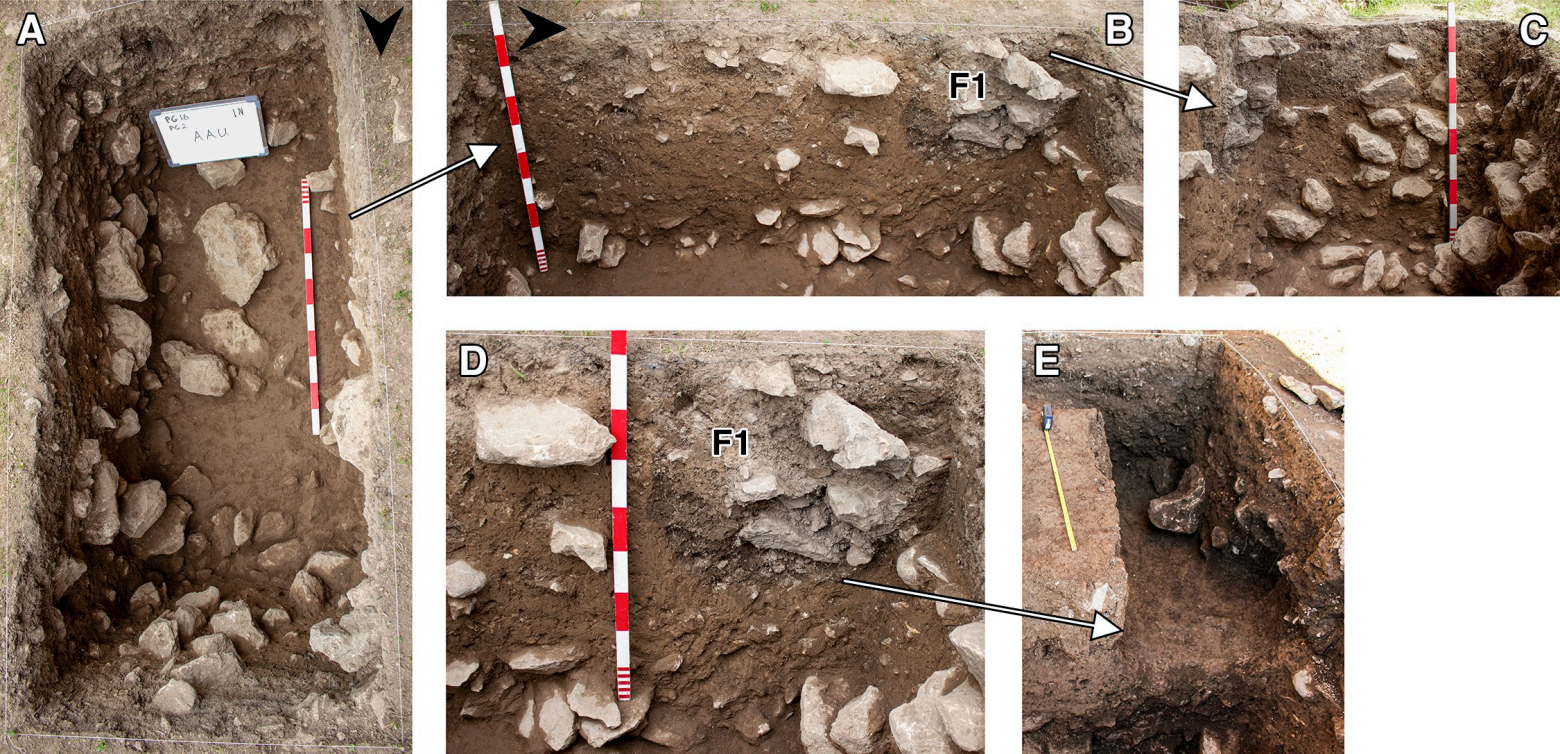
View of the western half of Trench A (photos by E Asouti). (A) Trench A as excavated at the end of the 2016 season down to the top of context AAU; (B-D) Location of Feature 1 (F1 = Howe’s step) on the western section of Trench A in 2016; (E) View of F1 cleared of Phase 4 deposits at the start of the 2017 season (Scale for E: 0.50m).

Trench A (Fig 7) was excavated to a maximum depth of ~1.50m from the topsoil, including sediment infilling well-defined natural cavities in the bedrock. The upper part of the deposit comprised a soft loamy dark soil containing a mixture of 20^th^ century materials that had accumulated over the cave threshold during and after the Iraq-Jarmo project excavations interspersed with Neolithic, Chalcolithic, Bronze Age (third millennium BC) and Medieval material. We successfully isolated the line of the edge of Howe’s trench immediately to the NW and to the NE of Trench A; a step into their excavation area was also uncovered in the NW corner of the trench (Fig 8).

We excavated between ~1.025–1.20m of *in situ* preserved late Pleistocene deposits in A where their upper part had not been removed by recent disturbance or by the edge of the Iraq-Jarmo project excavations. Howe’s trench reached a maximum depth of ~2m of which the top 0.20–0.60m consisted of mixed Holocene sediments; Zarzian deposits were found at depths between 0.60–1.30m (i.e., ~0.70m deep) ([2]: p.58). The Trench A late Pleistocene sequence was thus somewhat deeper than the Zarzian layers previously excavated at PG.

**Fig 9:**
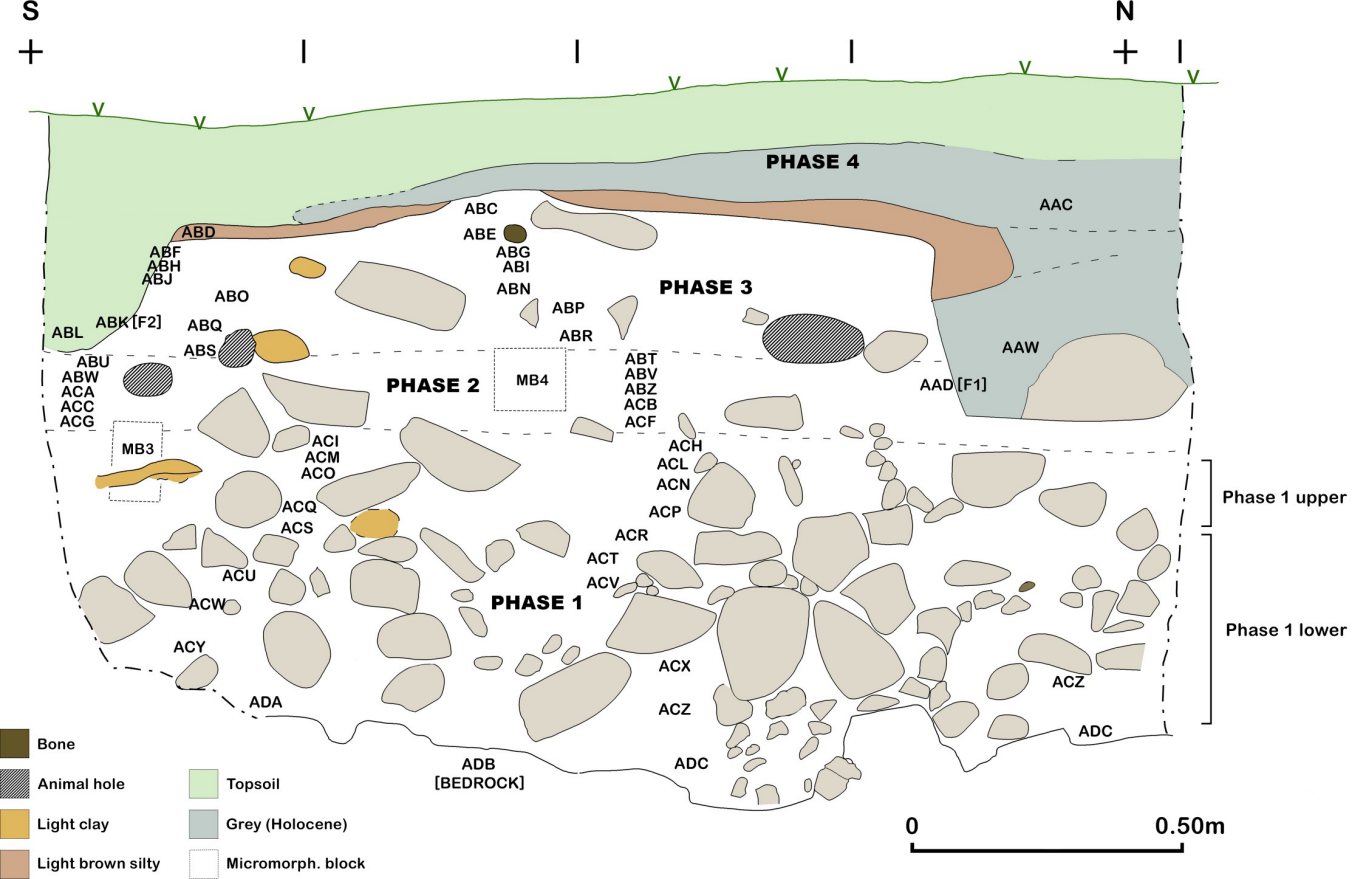
Trench A west section at the end of the 2017 season.

**Fig 10:**
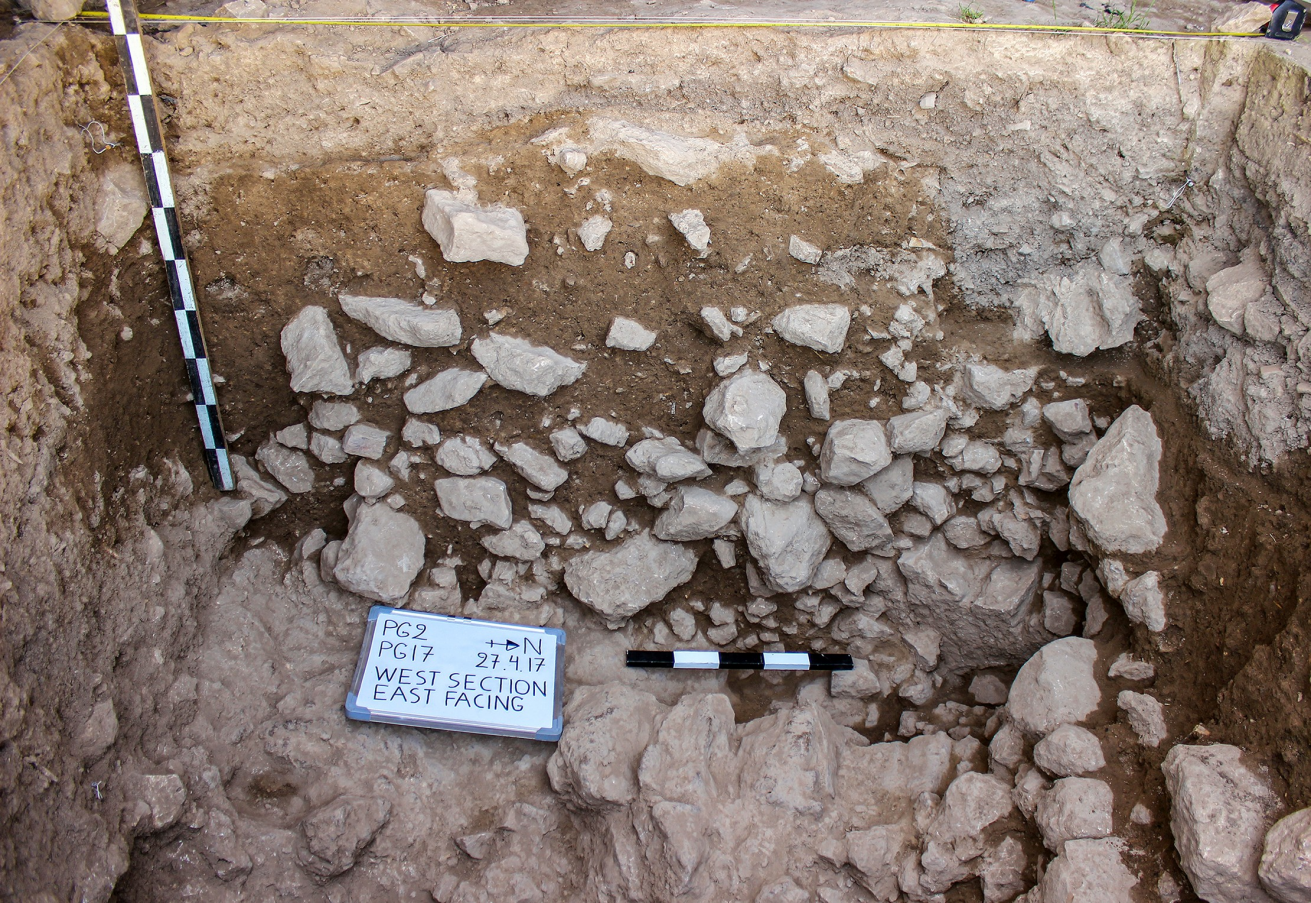
View of the Trench A west section at the end of the 2017 season (photo by E Asouti).

**Fig 11:**
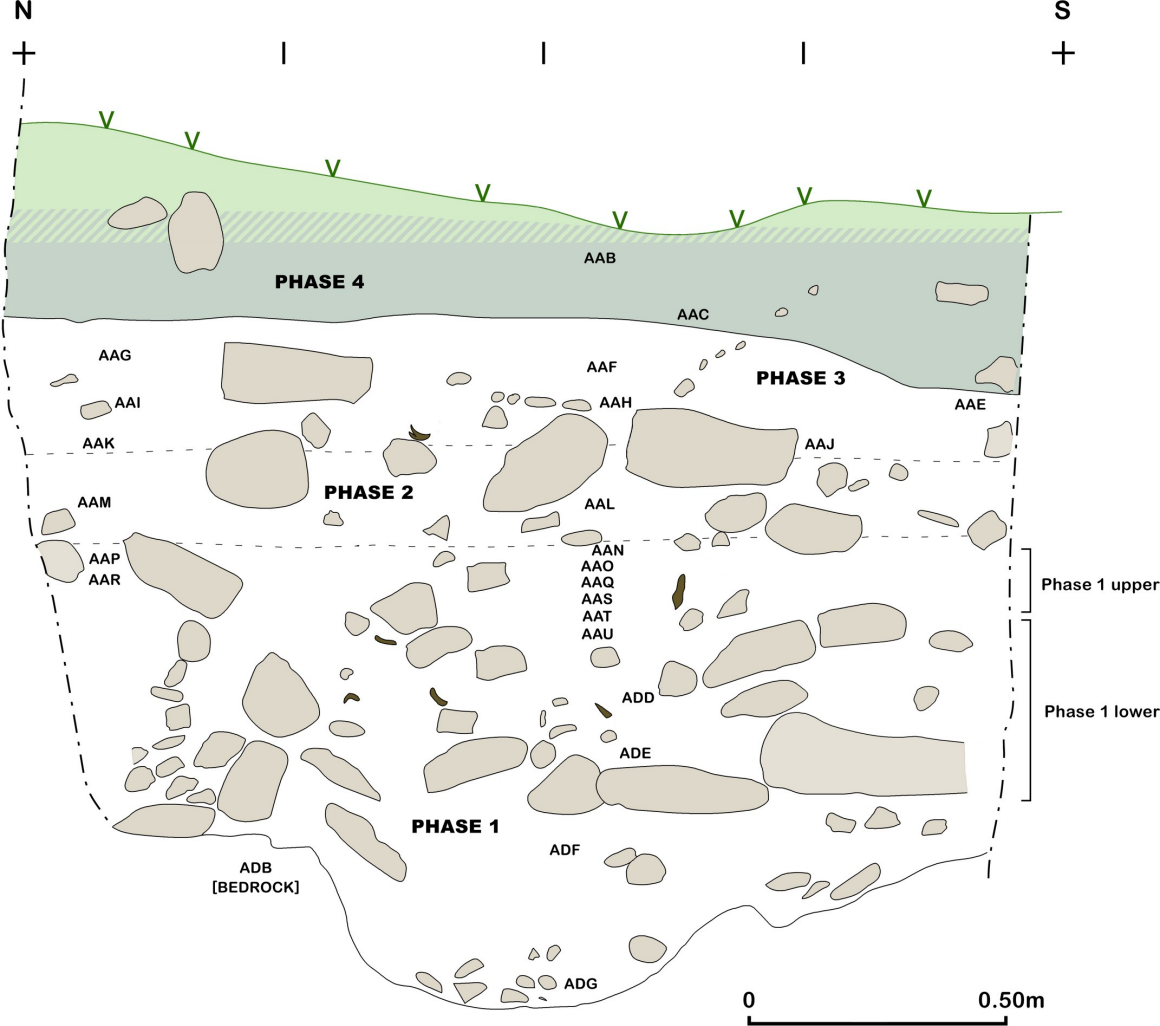
Trench A east section at the end of the 2017 season.

**Fig 12:**
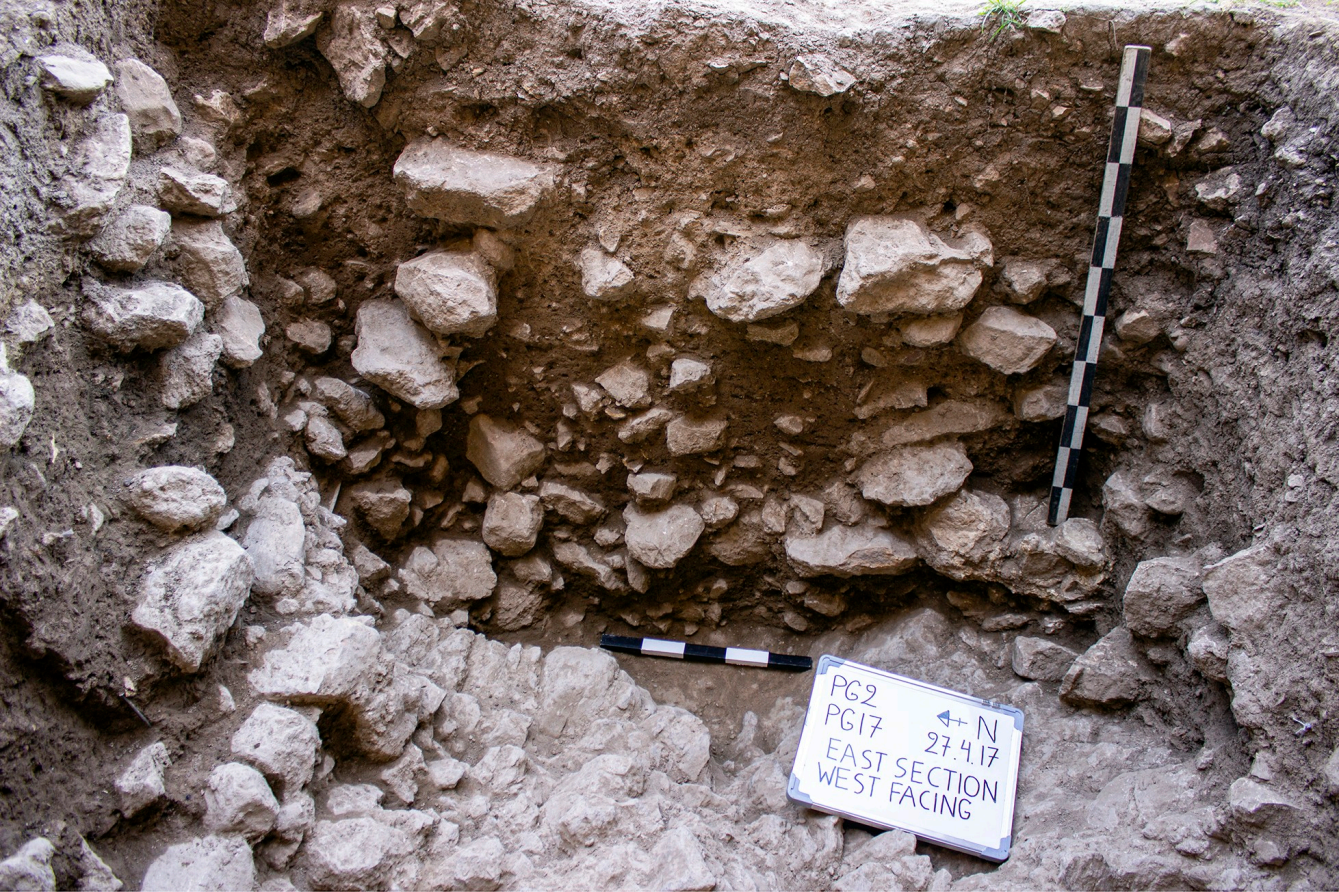
View of the Trench A east section at the end of the 2017 season (photo by E Asouti).

**Fig 13:**
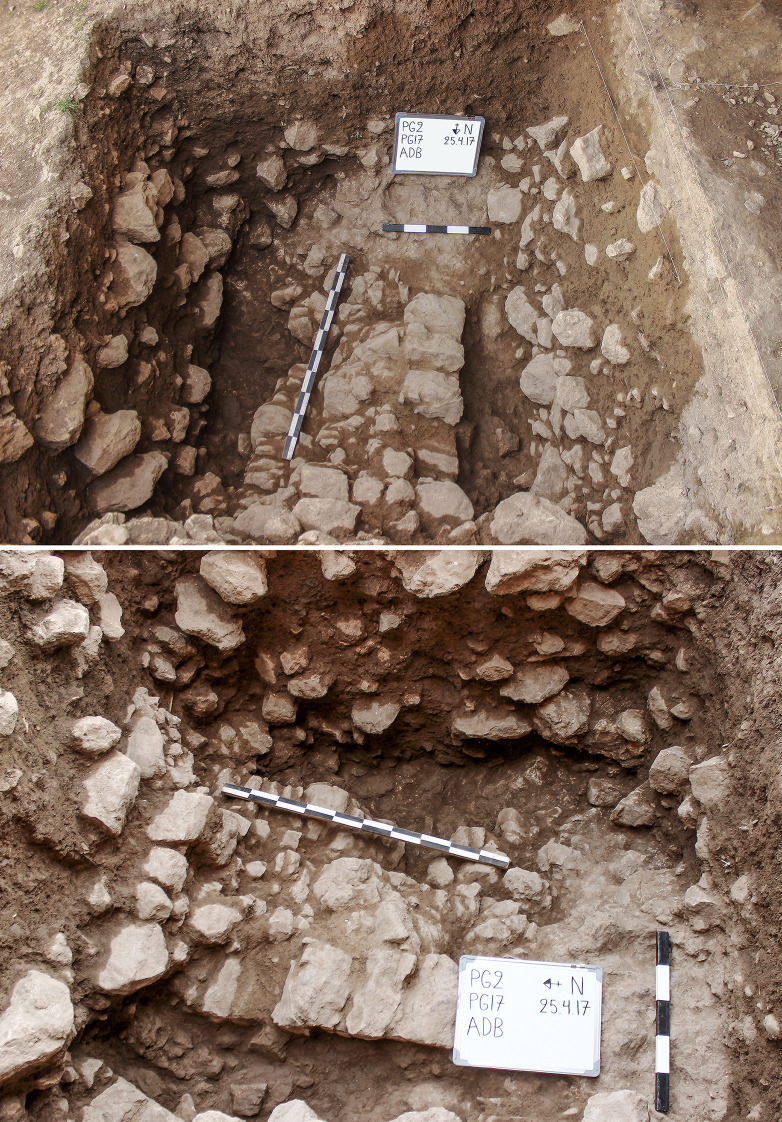
Views of the bedrock (ADB) in Trench A (photos by E Asouti).

The Trench A late Pleistocene sequence was divided into two sets of deposits. The stratigraphically earlier part contained dense concentrations of rockfall including both substantial stones and smaller debris. We designated this segment comprising the lower part of the dense rockfall overlying the bedrock as **Phase 1** (~0.50–0.65m, occasionally thicker) (Figs 9–12). The bedrock (ADB) was uneven and consisted of a SW to NE oriented limestone ridge, possibly related to the orientation of local folding in the Zagros range, with vertical edges leaving 2 declivities on either side also oriented SW to NE (Fig 13). The rockfall (~35–80% of the Phase 1 deposits) derived from the cave roof and walls, most likely due to water and ice action. The substantial quantity and size of some of the rock debris points to roof collapse. It thus appears likely that during its prehistoric occupation the PG roof extended further to the east (sheltering the whole of Area A). This reconstruction finds additional support in the absence of signs of a drip line within Trench A and in micromorphological analyses of Phase 1 upper deposits, which detected an increasing abundance of roofspall with depth (see S1 File and S2 Table).

Artefacts were found in sediment directly overlying the bedrock indicating that its surface was exposed when PG was first occupied. This mirrors the evidence available from the inner cave chamber reported by the Iraq-Jarmo project. If its entrance extended further out during the late Pleistocene the cave was probably larger and there would have been more space for activity in its outer chamber than suggested by its modern configuration. The bedrock declivities and depressions were probably filled first; they were packed with rockfall consisting of larger and smaller stones with moderate amounts of sediment between them, which contained concentrations of chipped stone, animal bone and charred plant debris. The chipped stone items were lying vertically and at all angles within these deposits, indicating their derivation from waste dumped and/or washed from the inner cave chamber, and from occasional *in situ* activities in the outer chamber. Overlying the bedrock ridge and these earlier fills were a series of deposits (designated as Phase 1 lower) with a maximum depth of ~0.35m containing dense amounts of rockfall (see Figs 9–11, and S4 and S5 Figs). Phase 1 upper comprised deposits with slightly less dense stone concentrations (~25–35% of the deposit) which we excavated in 4 arbitrary spits (0.03–0.05m each) (see Figs 9–11, and S4 Fig). The uppermost part of Phase 1 also contained a moderate quantity of small yellow and orange/red clay nodules alongside numerous artefacts lying at all angles. These included a proportion of horizontally bedded items, especially towards the southern end of the trench, suggesting a greater frequency of activities carried out in the PG outer chamber at this point in time.

**Fig 14:**
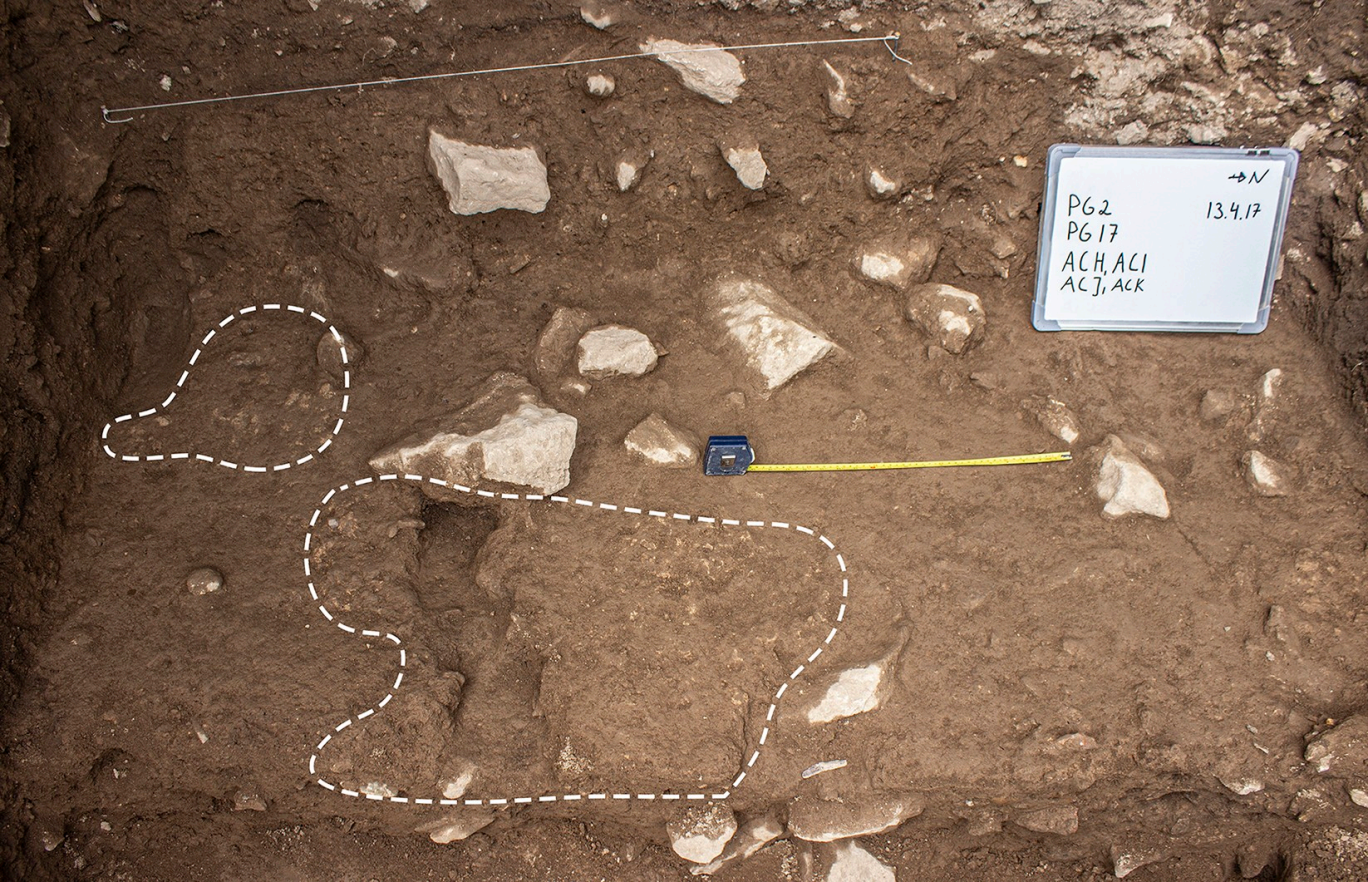
View of the discontinuous concreted yellowish surfaces (dotted line) marking the boundary between Phase 1 and Phase 2 deposits (scale: 0.50m) (photo by E Asouti).

The second set of late Pleistocene deposits excavated in Trench A was characterised by much lower concentrations of rockfall. The start of **Phase 2** (~0.15–0.20m deep) was marked by the presence of patches of a concreted yellowish clay surface (0.01–0.05m thick) containing small pebbles, artefacts and bones that overlay Phase 1 deposits in the southern part of the trench. There were 2 discontinuous patches of this surface that extended horizontally across the southern part of the trench, covering an area ~0.75m N-S and 0.25m E-W, bordered to the SW by a thicker patch of greyish clay (Fig 14). These deposits were relatively flat and smooth in their upper surfaces and were formed inside shallow depressions (0.01–0.05m deep). Both their base and the upper surface sloped to the east thus indicating that, at least for the duration of Phase 2, sedimentation generally sloped towards the mouth of the cave. They probably represent areas of pooled water formed within transient surfaces in which finer clay sediment was deposited and then concreted through a process of evaporation and are one of the few clear-cut stratigraphic boundaries found in the Area A late Pleistocene sequence. Micromorphological analyses of these yellowish silty clay aggregates (S1 File and S2 Table) indicated that their composition is relatively consistent including a moderate calcareous component. Although they contained some unsorted charred fragments of angular bone and micro-charcoal, these were found embedded in their groundmass thus replicating macroscopic observations in the field for the inclusion of similar materials in their matrix. No evidence was detected in the micromorphological thin sections for the presence of living surfaces, although clay patches with compacted smooth surfaces were dug in other parts of the Area A sequence.

**Fig 15:**
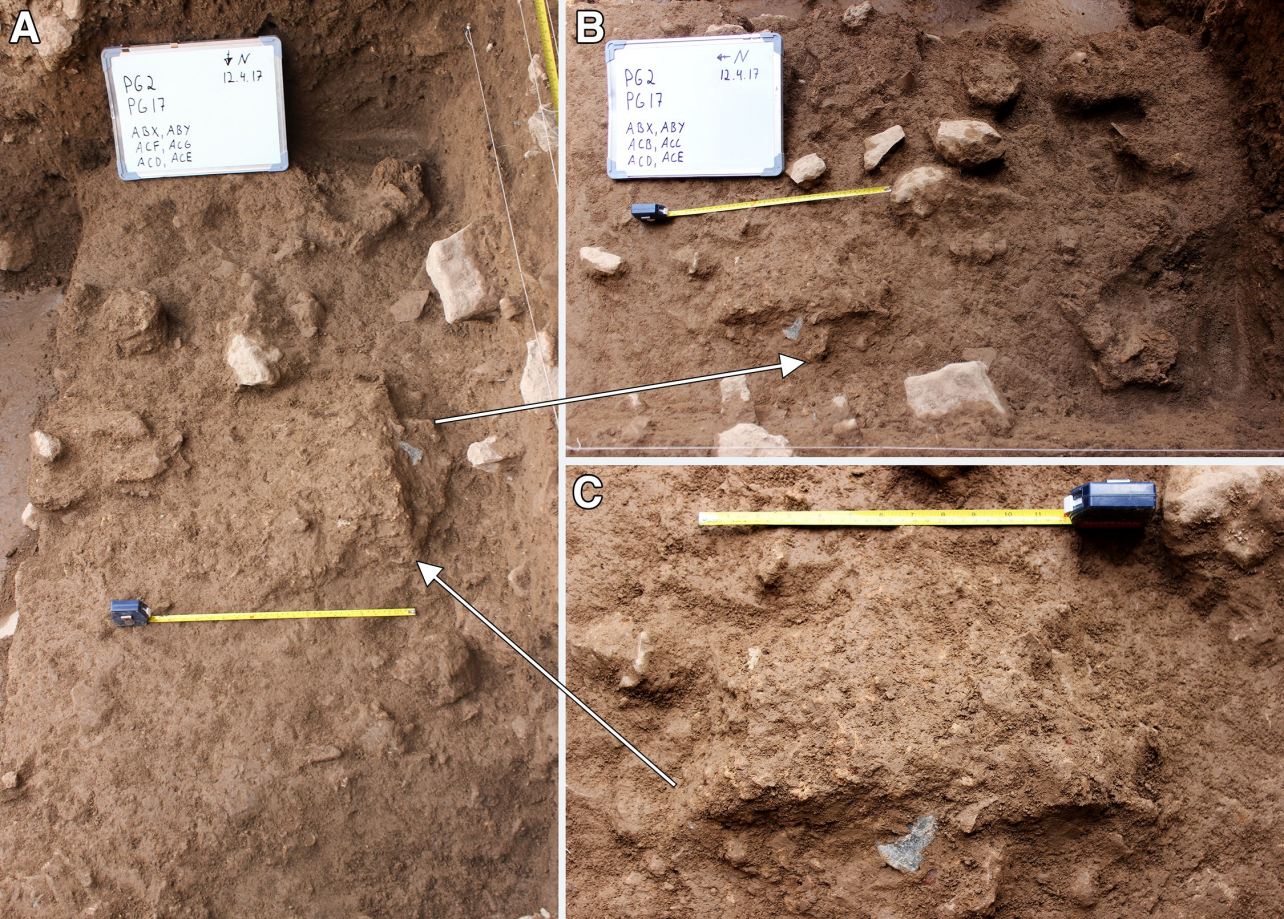
View of ABY comprising dispersed clay nodules and patches with artefacts and animal bone inclusions (photos by E Asouti). (A-B): scale 0.50m; (C): scale 0.30m.

The bulk of **Phase 2** deposits comprised ~0.10–0.15m of a light-yellow brown clayey loam that contained modest quantities of stone (5–25%) with fewer stones found in the southern quarter of the trench, and only sporadic inclusions of larger rockfall. Chipped stone lay at various different angles also including some horizontally lying artefacts. At various points, small horizontally bedded concentrations of retouched chipped stone tools were found suggesting the sporadic occurrence of *in situ* activities. Red ochre fragments were found scattered through the lower part of the Phase 2 deposits, although red ochre fragments occurred throughout the Area A late Pleistocene sequence. In the middle of Phase 2 deposits lay a series of clay lumps, slightly greyer and more clayey than the surrounding sediment. These were sub-rectangular or oval, ~0.10m in maximum size and contained small nodules of red/orange-brown clay. In the middle of the trench there was a more extensive concentration of small yellow, red and orange clay nodules (ABY ~0.7 x 0.36m) (Fig 15). The base and top of ABY sloped to the east following the general bedding of the late Pleistocene sediments in this part of the Area A sequence.

**Fig 16:**
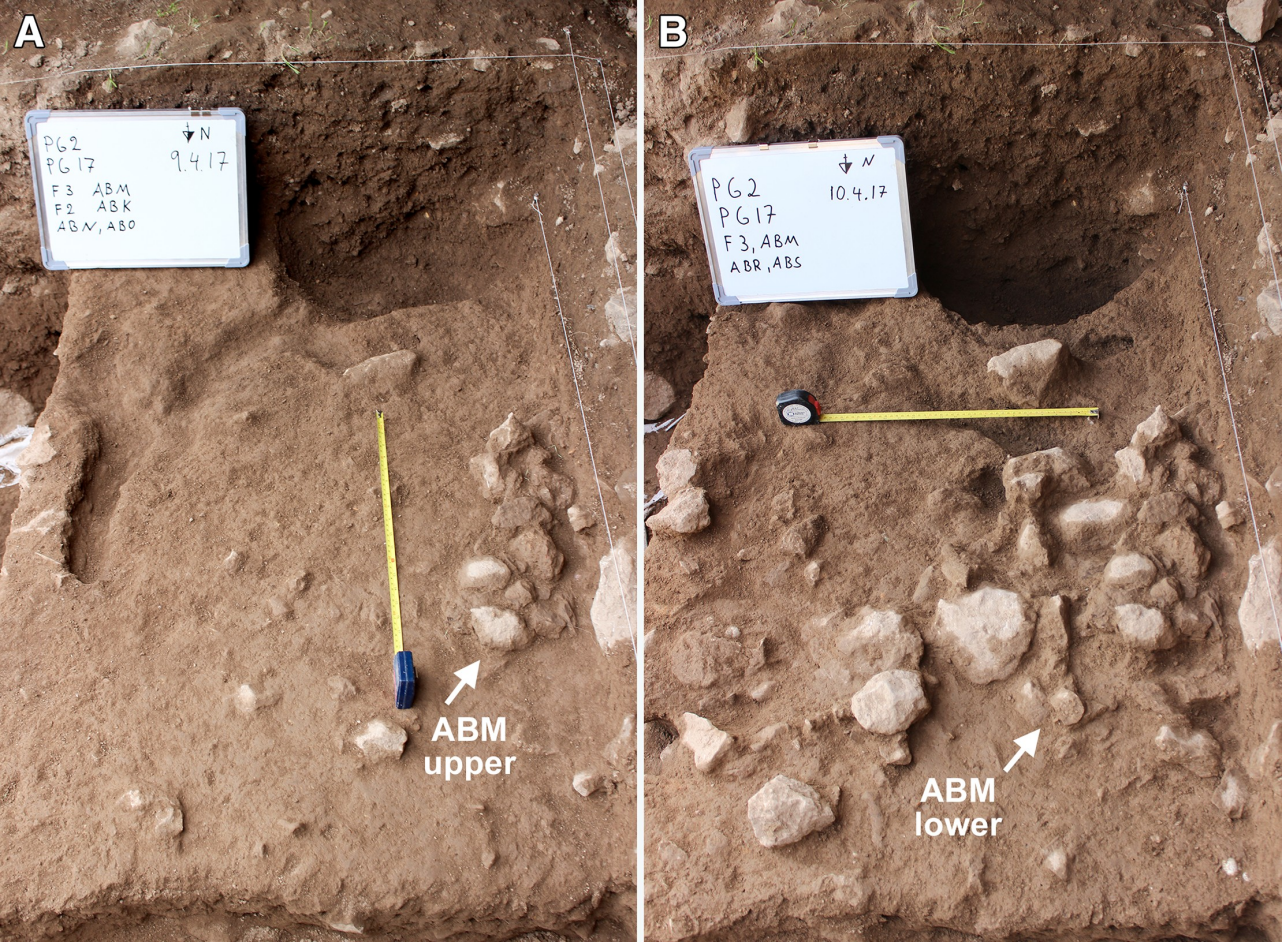
View of the hearth-like Feature 3 (ABM) (photos by E Asouti). (A): ABM upper; (B): ABM lower (scale: 0.50m).

**Phase 3** deposits were ~0.25–0.35m deep. They consisted of a series of light-yellow brown clay sediments with sporadic presence of larger and smaller stones. The animal bone and chipped stone lay at various angles, with some being horizontally bedded, likely representing the accumulation of waste material dumped or tossed from the inner chamber alongside occasional *in situ* activities. The basal boundary of Phase 3 was distinguished from the underlying Phase 2 deposits by the presence of a slightly disturbed, hearth-like feature (Feature 3/ABM) (Fig 16). F3 consisted of a sub-rectangular perimeter (~0.80m in length) of medium-size stones (ABM lower) overlain by a layer of smaller stones (ABM upper) that extended into the interior of F3. In both layers a high proportion of the stones were burnt. There were also occasional charcoal flecks in the fill of F3, although there was no evidence of an *in situ* fuel bed. F3 extended 0.60m SW to NE and projected 0.40m from the western section of Trench A, the remaining half or two-thirds of it being located inside the baulk (it is quite possible that they have been removed by Howe’s trench). The sediments around F3 contained higher concentrations of small light-yellow and orange clay nodules, which might represent burnt clay associated with hearth use. Animal bone and chipped stone found within these deposits were lying at all angles indicating their origin as waste material dumped into this area.

**Phase 4** deposits (~0.15–0.30m deep excluding the fill of the deeper cut features) were quite distinct from the underlying late Pleistocene sequence. They principally consisted of dark grey loose soft sediments, including both the material directly overlying Lateglacial deposits and the fill of the features that were cut into the Lateglacial sediments. The latter comprised the passage (F1/AAD) dug by Howe to provide a step into his trench (detected in the NW corner of Trench A) and a small 20^th^ century pit (F2/ABK) that cut the SW corner of Trench A (Figs 8 and 9).

#### Area B

**Fig 17:**
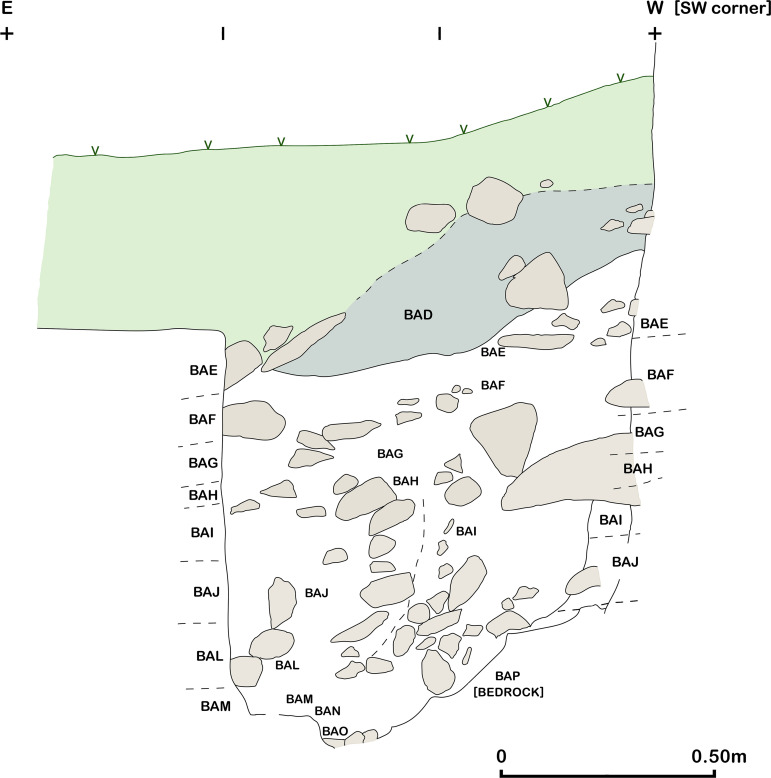
Trench B south section at the end of the 2017 season.

**Fig 18:**
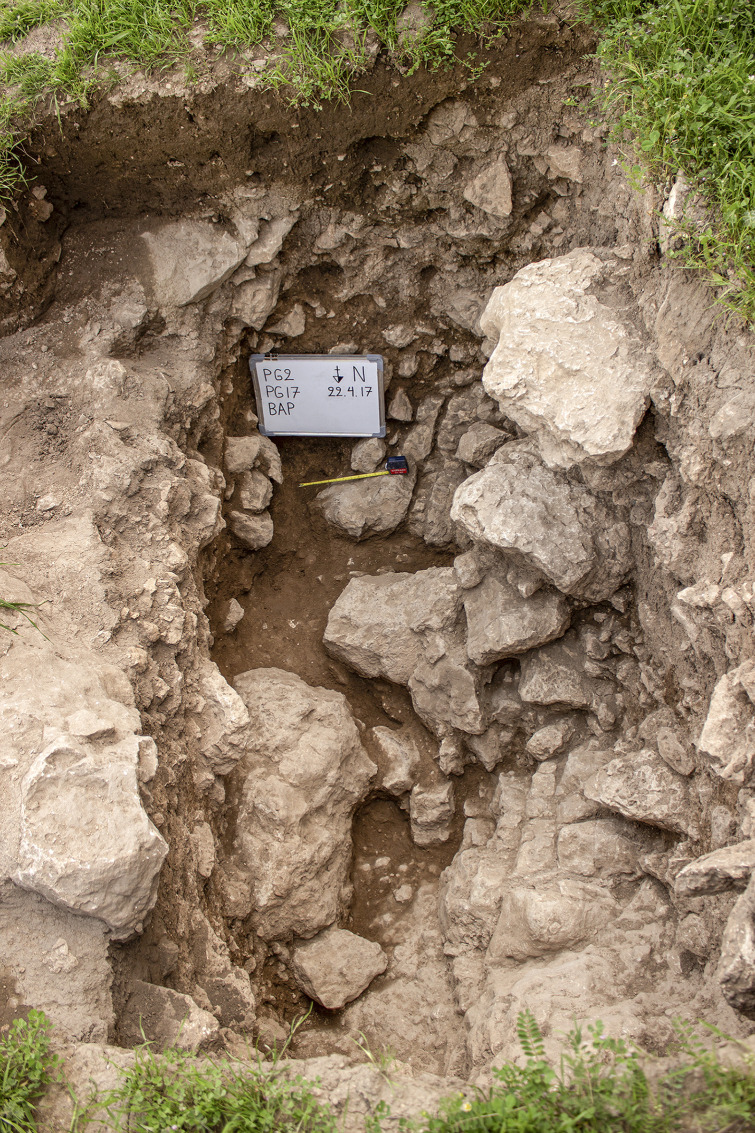
View of the bedrock (BAP) in Trench B (scale: 0.30m) (photo by E Asouti).

Excavations in Trench B (Fig 7) revealed a maximum of 0.85m of late Pleistocene sediment very similar in nature to that encountered in the deposits excavated in Area A: a light grey brown loamy clay, with occasional patches of darker brown and beige clay and a slightly siltier texture than the Trench A sediments, notably on the downslope side of B. This was removed in eight ~0.10m spits (Fig 17), which were excavated sloping to the east in conformity with the present-day slope, the bedding of some of the stones found within these deposits, and the underlying limestone bedrock (Fig 18). There was on average a higher density of stones in Trench B, with the exception of the deepest zone (Phase 1 lower) dug in Trench A. These Pleistocene deposits were overlain by ~0.50m of darker Holocene colluvium mostly deposited in the 20^th^ century mixed with Neolithic, Chalcolithic and Bronze Age artefacts.

The Trench B Pleistocene sediments contained materials washed out of the cave mixed with general colluvial deposits and rockfall. As in Trench A, cultural material was encountered in sediments immediately overlying the bedrock. Artefacts and animal bone were found bedded at all angles throughout the Pleistocene deposits, possibly representing materials washed down and/or tossed from the cave. There was no evidence to suggest that the terrace was used for recurrent *in situ* activities. It seems more likely that during the prehistoric habitation of PG a slope built up gradually in front of the cave entrance. Our excavation thus suggests that, unlike Zarzi where late Pleistocene habitation was focused on the terrace outside the cave, or Natufian sites in the Levant, at PG there is very little evidence for the regular use and/or occupation of the terrace area.

### Radiocarbon dating the PG sequence

Radiocarbon dates previously obtained by the Iraq-Jarmo project which could be reliably sourced in the literature include 2 faunal bone collagen samples (UCLA-1703A, UCLA-1714D) and 3 (botanically non-identified) charcoal samples (GrN-6415, GrN-6356, GrN-6357) (see also Fig 3). The UCLA dates were characterised by very wide error margins (>±200) and were thus deemed unreliable. The published information on the Groningen charcoal samples does not indicate if they represent wood charcoal and/or other charred plant parts, and if they were dated as single entities or bulk samples. GrN-6357 and GrN-6356 are in stratigraphic order and derived from superimposed spits, while GrN-6415 is denoted as originating from a depth <60cm.

A further set of AMS radiocarbon dates were obtained in the 2000s from animal bone. These dates are not in stratigraphic order: 3 of the dated collagen samples produced radiocarbon age determinations considerably younger than the Groningen charcoal samples originating from the same spits in Howe’s trench (in Fig 3 compare B-159545 to GrN-6356 from the 60-80cm spit, and B-159542 and B-159544 to GrN-6357 from the 80-100cm spit). It is worth noting that the UCLA collagen dates also appear systematically younger than the Groningen charcoal ages (compare GrN-6357 to UCLA-1714D from the 80-100cm spit and to UCLA-1703A from 120cm near the base of Howe’s sequence). This suggests that the mixing and movement of material between spits and/or mistakes in sample labelling in the field are unlikely sources of error. Another potential source of error with Palaeolithic bone protein collagen samples dated before the advent of ultrafiltration pre-treatment methods, is that the collagen may contain low molecular weight contaminants of modern origin which will invariably result in systematically younger radiocarbon age determinations [45–47]. The possibility of modern contamination (from dirty excavation tools, cigarette ash or smoke, and packing materials such as cotton wool or paper) is high for samples excavated in the 1950s when appropriate field sampling protocols had not been developed yet. Whether these 3 younger AMS collagen age determinations represent the result of bioturbation and/or inadequate field sampling protocols alongside insufficient pre-treatment, could be resolved by re-dating collagen samples from secure Zarzian contexts in Howe’s sequence with the addition of an ultrafiltration step in sample pre-treatment.

After excluding the 3 young collagen AMS dates and the UCLA dates the summed probability distribution of the remaining radiocarbon determinations from Howe’s trench indicates a range ~17,700–15,600 cal BP for the bulk of the Iraq-Jarmo project Zarzian sequence (60-100cm). The 2 dates obtained from the more disturbed upper part of Howe’s stratigraphy (B-159543 from the 20-40cm spit and GrN-6415 potentially from as deep as just above 60cm) extend this timespan to ~13,400 cal BP, while its uppermost layer (10-20cm) appears to date as late as the mid-Holocene (~5800 cal BP) or later (Figs 3–19, and S6 Fig).

**Fig 19:**
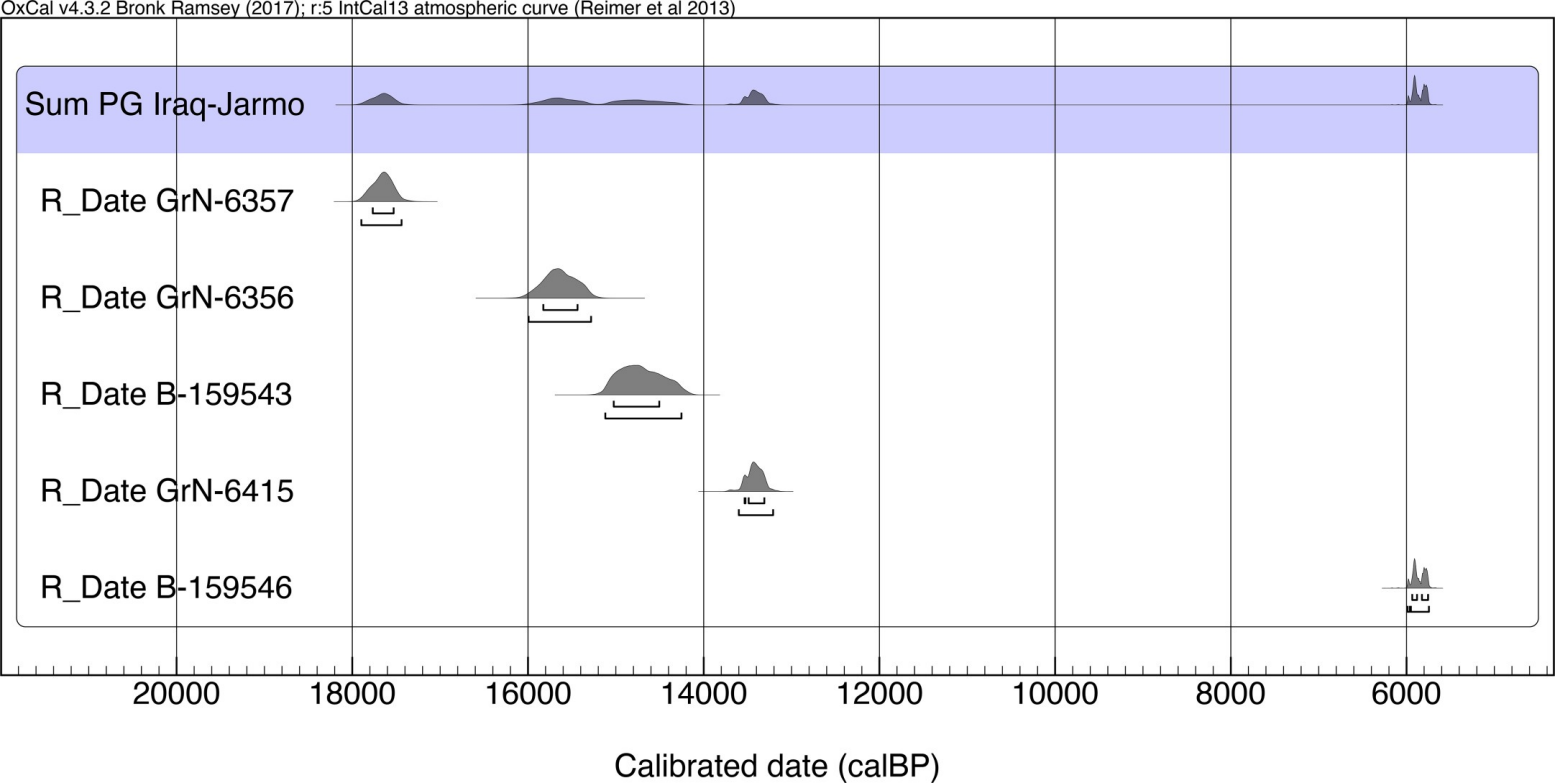
Summed probability distribution plot of Iraq-Jarmo project radiocarbon dates.

Obtaining a new sequence of AMS radiocarbon dates was a key objective of the EFEC project excavations at PG, given also the continuing absence of radiometric chronologies from Zarzian sites in the northern and central Zagros. During the 2016 and 2017 seasons we collected 138 charcoal samples in the field. Charcoal was prioritised for analysis due to the highly fragmented nature of animal bone found at PG and the anticipated low levels of collagen preservation, subsequently confirmed through collagen preservation tests run by Jessica Pearson at Liverpool (tested bones are listed in S3 Table; they were all heavily mineralised and collagen was absent or highly degraded). All charcoal samples were collected with clean tools, their 3D coordinates were recorded, and were individually labelled and packaged in aluminium foil. These samples were analysed in the Archaeobotany laboratory of the University of Liverpool in order to evaluate their suitability for radiocarbon dating. The laboratory protocol for sample selection involved the weighing and microscopic examination of each charcoal specimen, in order to produce botanical taxon identifications and assess their preservation status. As first priorities were targeted short-lived single entity samples (i.e., individual wood charcoal fragments) botanically identified as *Amydgalus* (almond). The natural lifespan of *Amygdalus* species is ~40–50 years in unmanaged form, extending to usually no more than 70–80 years for mature managed trees. The anthracological results (see below, **Archaeobotany** section) indicated the collection of fuel wood predominantly from *Amygdalus* shrubs, which suggests a potentially even shorter natural lifespan for the *Amydgalus* wood charcoal samples selected for radiocarbon dating. Exceptionally, 2 dates were obtained from short-lived charred plant materials retrieved from archaeobotanical flot fractions: B499843-ADG (*Amygdalus* charred nutshell) and B499844-ABR (*Amydgalus* wood charcoal). Apart from its obvious short-lived nature, the ADG nutshell fragment was also prioritised because the wood charcoal samples collected from the same context in the field appeared upon microscopic examination to be heavily degraded. They were covered by a continuous film of mineral precipitates that had penetrated their anatomical features; we could not thus exclude the possibility that they represent residual ‘old’ charcoal deposited in the sediment. Most of the charcoal samples collected in the field from Phase 3 contexts (including ABR) did not meet our sample selection criteria: they were too small to be dated as single entities, botanically indeterminate or too degraded and/or mineralised. We thus prioritised better-preserved single entity charcoal specimens (minimum weight before pre-treatment 8-10mg) originating from flot fractions.

**Fig 20:**
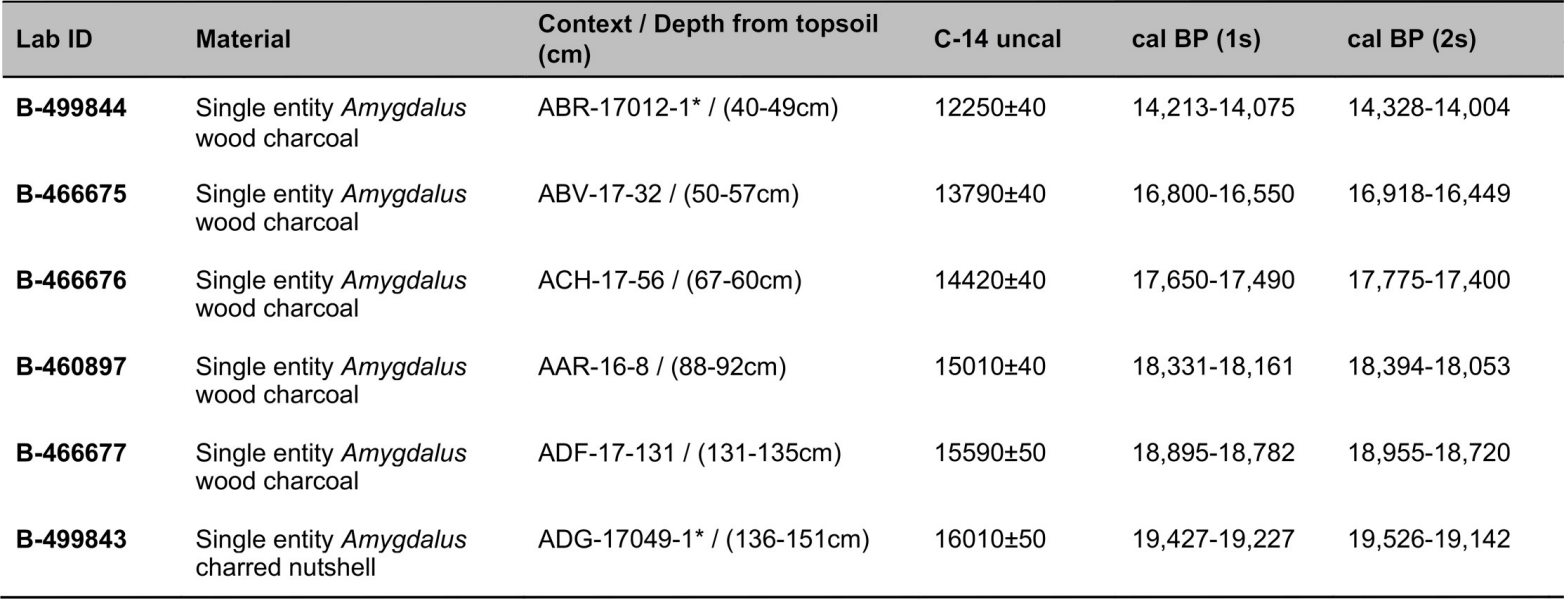
Radiocarbon dates (unmodelled) from the EFEC project sequence in Area A. *Flotation sample specimens; dates were calibrated using OxCal v4.3.2 [18]; IntCal13 atmospheric curve [19].

Fig 20 presents a list of all the currently available EFEC project AMS dates (full details of the attributes of the dated samples are presented in S4 Table). We prioritised for radiometric dating the Area A late Pleistocene sequence due to its archaeological characteristics: evidence for at least some *in situ* prehistoric activity and feasibility of stratigraphic phasing permitting Bayesian modelling [48]. The Area A dates are in stratigraphic order from the oldest to the youngest and have acceptably narrow standard errors.

**Fig 21:**
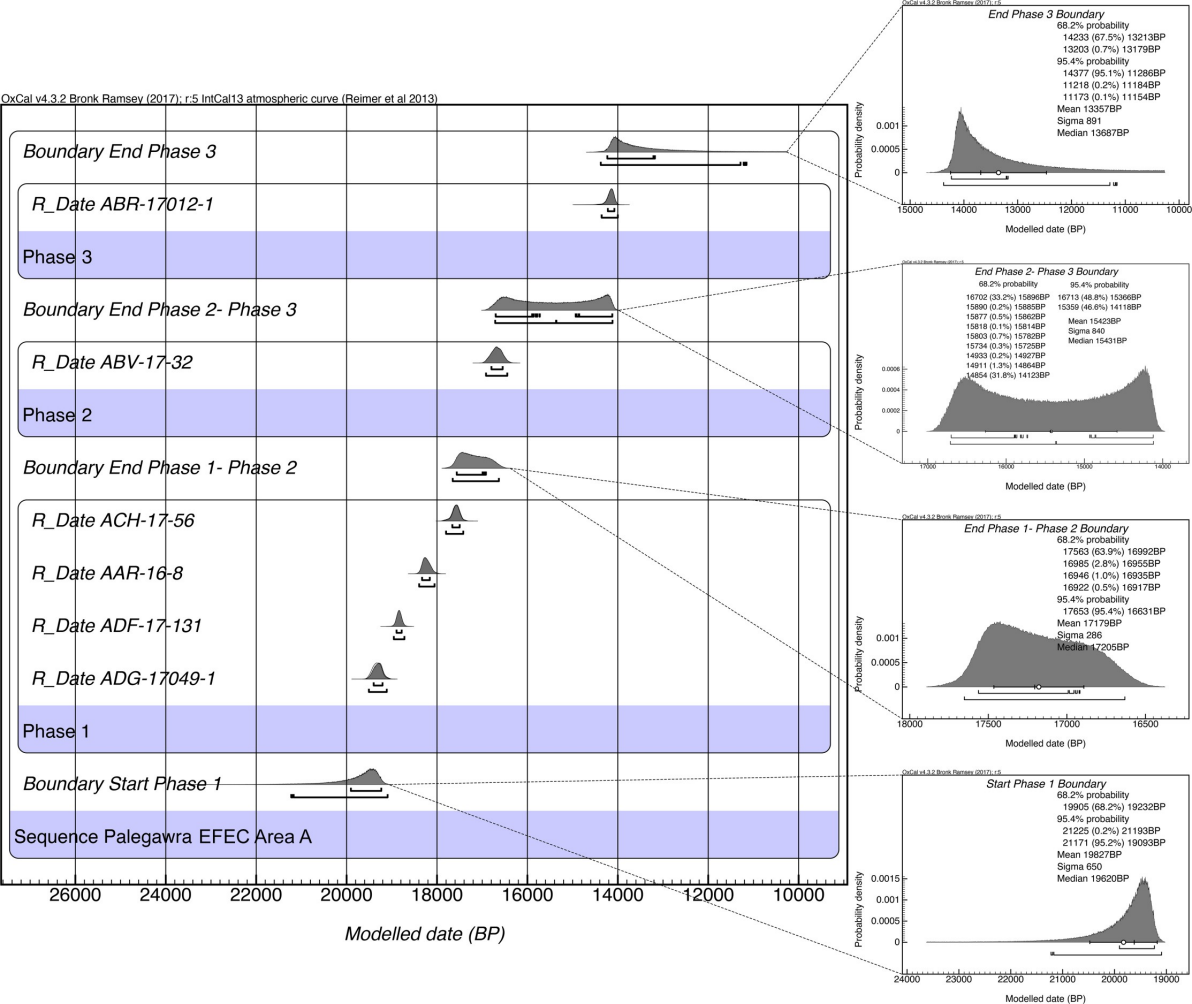
Bayesian age model of the late Pleistocene sequence excavated in Trench A (age model produced by C Kabukcu).

The preliminary age model of the Area A sequence (Fig 21 and S7 Fig, S5 Table) indicates that PG was first occupied between ~19,900–19,200 cal BP (1σ) or ~21,200–19,100 (2σ). Phase 1 is dated by 4 AMS determinations: 2 from the stratigraphically earliest contexts ADG and ADF, 1 from AAR, and 1 from ACH which brackets the upper boundary of Phase 1. The end of Phase 1/Phase 2 boundary is modelled at ~17,600–16,900 cal BP (1σ) or 17,600–16,600 (2σ). The duration of Phase 1 is thus modelled between 2–3 kyr. No appreciable gap in cave occupation is evidenced with the subsequent Phase 2, which has produced a single date from ABV of ~16,800–16,600 cal BP (1σ) near the upper end of the Phase 2 deposits. The age model points to a possible gap at the end of Phase 2: a single date of ~14,200–14,100 (1σ) was obtained from ABR near the base of Phase 3 deposits. The Phase 2/Phase 3 boundary is thus modelled between ~16,700–14,100 cal BP (with a median of ~15,400 cal BP). The upper end of the Area A Zarzian sequence is modelled to ~13,200 cal BP (1σ). Considering that there are ~0.25m of currently undated Phase 3 deposits overlying ABR the end of the Area A Zarzian sequence may date as late as ~13,000 cal BP.

Overall, based on the available evidence, the Area A Phases 1 and 2 appear to overlap with the late Pleniglacial (GS-2.1) while the start of Phase 3 corresponds to the beginning of the Lateglacial (GI-1e warm period). The absence of dates from the bulk of Phase 3 deposits currently precludes a more precise estimation of its duration. Provisionally, it appears probable that the PG Zarzian occupation terminated sometime before the end of GI-1 and the start of GS-1 (Younger Dryas) at ~12,900 cal BP (see also Fig 4).

### The chipped stone

In this paper we present the first results of the analysis of select contexts focusing on the late Pleistocene sequence excavated in Area A alongside a lithic sample from Area B for exploring spatial variation between the cave entrance and terrace areas. To date, 5181 chipped stone items have been recorded (4580 from Area A and 601 from Area B) including small finds excavated and 3D recorded in the field and artefacts recovered from flotation heavy residues and a few dry sieved contexts. More material awaits study in the Sulaymaniyah Directorate of Antiquities and Heritage. The chipped stone assemblage presented here represents ~40% of the overall PG lithic sample retrieved from undisturbed contexts, and >50% of the lithic assemblage recovered from Area A. The contexts studied to date from Area A are spread evenly across Phases 1–3 thus providing a representative picture of the overall characteristics of the lithics retrieved from each Area A phase. It is possible that, once a larger lithic sample from Area B is analysed, we might be able to investigate with a higher degree of precision variability through the Area B sequence and uncover more nuanced similarities and differences with the Area A lithic assemblage. In the following sections we present a first stage analysis of the main lithic categories found thus far at PG. Preliminary sorting has identified basic debitage and tool categories, also including more specific microlith categories (see also S6 Table). Detailed attribute data were also collected on retouch type and location, and detailed technological information on platform and bulb characteristics using a slightly modified version of the Wembach module [49].

#### Raw materials

**Fig 22:**
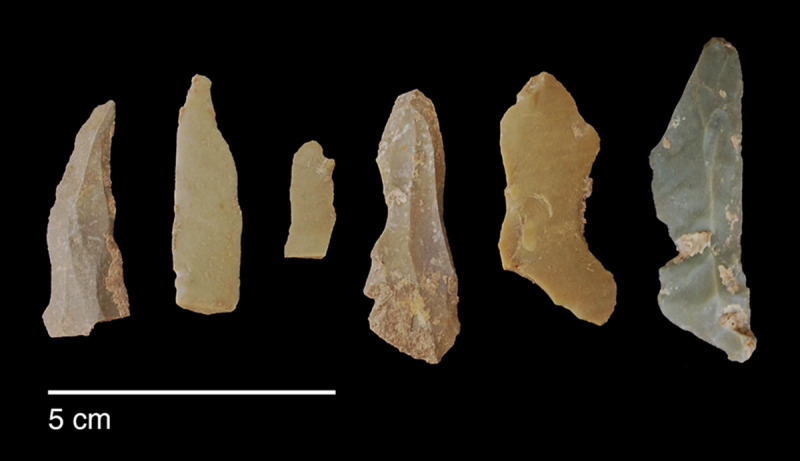
Category 1 raw materials (photo by D Baird).

**Fig 23:**
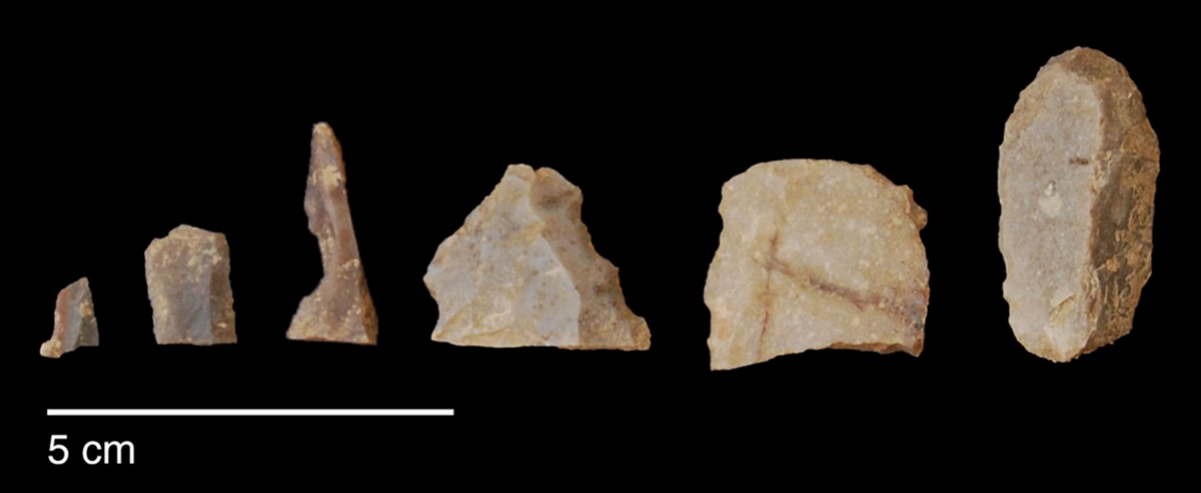
Category 2 raw materials (photo by D Baird).

The PG lithic assemblage comprises two main categories of raw materials. **Category 1** is represented by a light-dark green homogeneous opaque-clear coloured chert (Fig 22 and S8B and S8C Fig) with thick banding and smooth cortex bearing no signs of prolonged water transport, and a red brown opaque clear coloured homogeneous chert of similar texture (S9B Fig). These cherts were preferentially used for the production of blade tools and microliths. The presence of the whole reduction sequence including some primary flakes indicates that they were brought to PG in the form of complete or virtually complete chert nodules. Sources of raw material visually identical to the Category 1 cherts have been located ~35km to the west of PG, on the western side of the Chamchamal valley. They may have been collected from source or from wadi beds. **Category 2** is represented by cherts exhibiting much more varied texture, colour and cortex including glossy greys and pinks with rolled cortex, often banded (Fig 23). Similar raw material is located ~4km south of PG, in the bed of the river draining the Bazian valley. No raw materials suitable for knapping have been located in the bed of the wadi adjacent to the cave.

**Fig 24:**
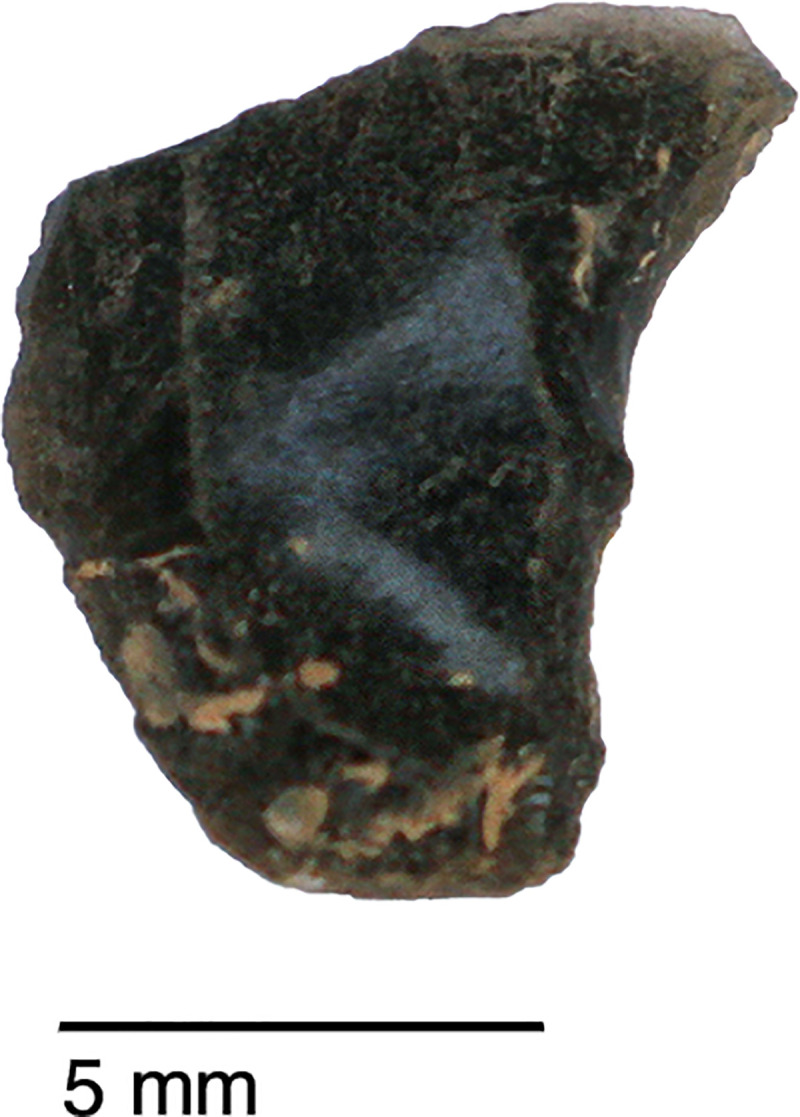
Obsidian retouched bladelet from context AAH (photo by D Baird).

The sole non-local raw material is obsidian. A single obsidian piece, a retouched bladelet, was found in context AAH (Phase 3). Its Zarzian attribution is confirmed by the bladelet oriented technology and retouch characteristics (Fig 24). Its greenish colour matches the descriptions of peralkaline materials known from the Nemrut Dağ/Lake Van sources in eastern Anatolia >400km to the NW of PG. The Nemrut Dağ attribution also matches previously reported characterisations of the 2 obsidian pieces discovered by Garrod at Zarzi [50]. Obsidian bladelets (~20 microliths, including a lunate and microburins) were also found by the Iraq-Jarmo project excavations in Zarzian deposits dug between ~0.60–1.20m. Their presence and technological characteristics suggest that at least some of the obsidian artefacts found in Howe’s trench are of Zarzian origin rather than being intrusive from mixed Holocene layers closer to the surface ([2]: p.58, [9]: p.141).

#### Reduction strategies

**Fig 25:**
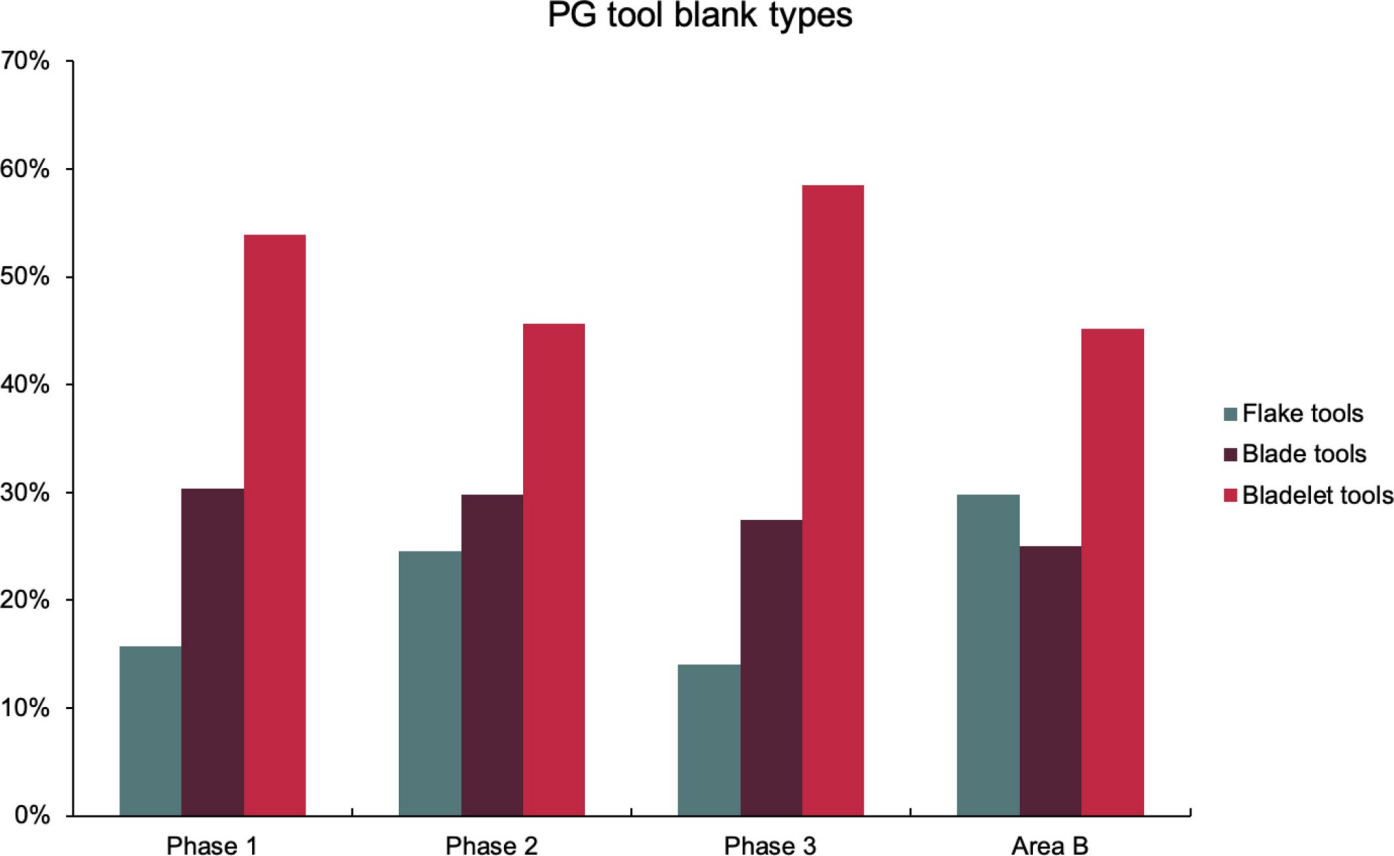
Relative proportions of blank categories for retouched tools (Area A: Phases 1–3 & Area B).

While flakes predominate in the debitage (53–63%) across all late Pleistocene layers excavated in Areas A and B, bladelets were the primary goal of lithic production, as blanks for retouched tools including both microliths and other bladelet tools (45–58% of blanks) followed by blades (25–30%) and flakes (14–30%) (Fig 25). Many flakes therefore represent the by-product of on-site core shaping. The presence of ‘change of orientation’ and multiple platform flake cores (together representing ~37% of the core assemblage) also attests to the existence of a dedicated flake-oriented production sequence. Both single and opposed platform bladelet cores (~40% of the core assemblage) also attest to the importance of bladelet production. Significant proportions of single platform sub-pyramidal cores (~24%) indicate the importance of single platform production strategies. Blade and bladelet production were probably continuous and sequential from the same cores.

Primary and secondary flakes, cortical blades and bladelets, frequent and varied core types, crests and rejuvenation elements attest to all stages of on-site knapping, from raw nodule to core, of both principal categories of raw material utilising all the main reduction sequences. The western Chamchamal valley raw material group (Category 1) was used for most tool types but was particularly favoured for the production of blade tools (including denticulates, burins and scrapers), flake scrapers (some of which may represent further reduced blade scrapers) and microliths (S8B and S8C Fig).

#### Retouched tools

**Fig 26:**
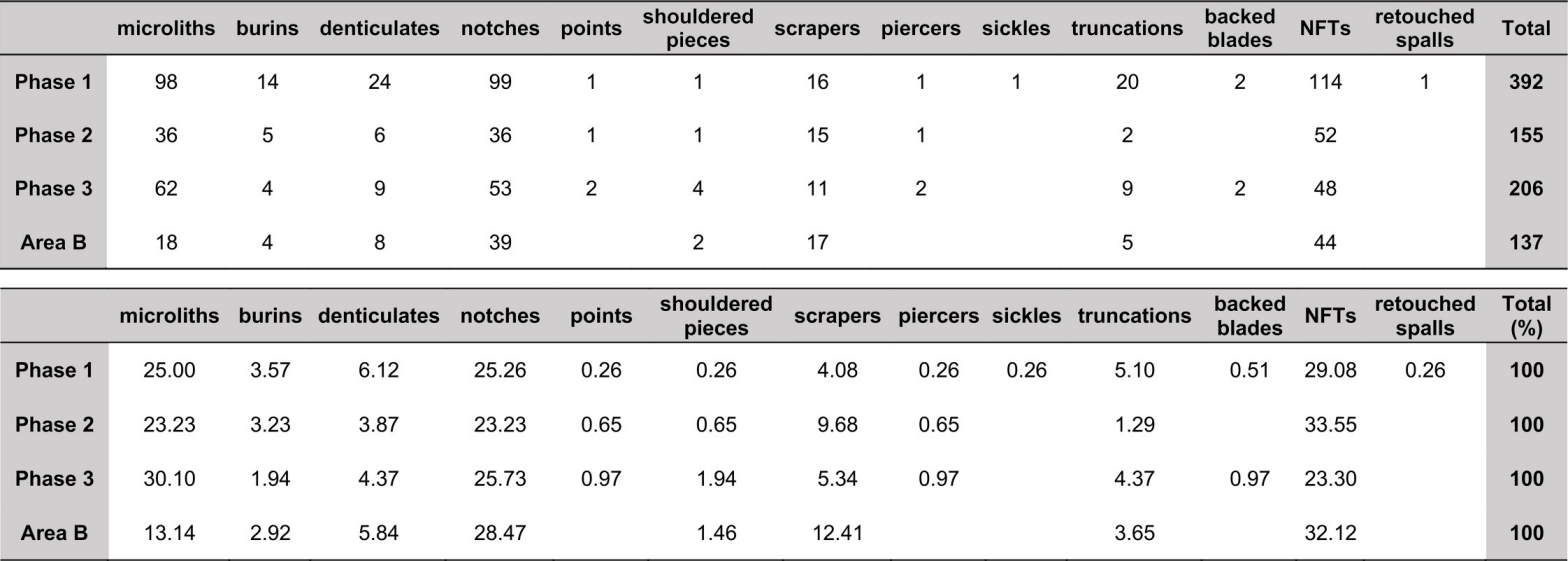
Counts and relative proportions (%) of retouched tools from Area A (Phases 1–3) and Area B.

**Fig 27:**
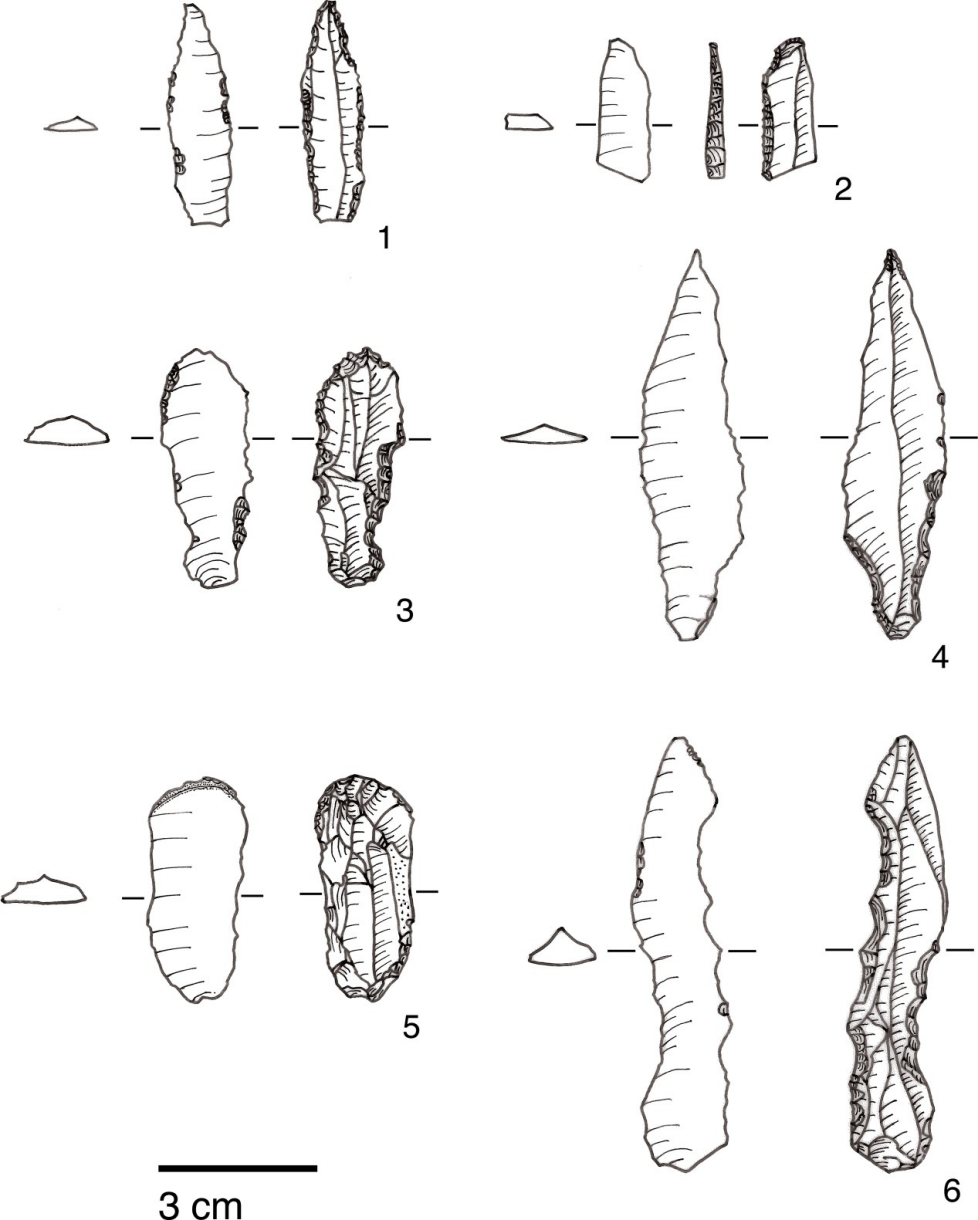
PG tools (drawings by D Baird). 1. Piercer (context AAJ); 2. Backed blade fragment (context ACC/SF no. 39); 3. Notched blade (ACM/SF78); 4. Zarzian point (ABI); 5. End scraper (AAL); 6. Denticulate (ACR/SF106).

**Fig 28:**
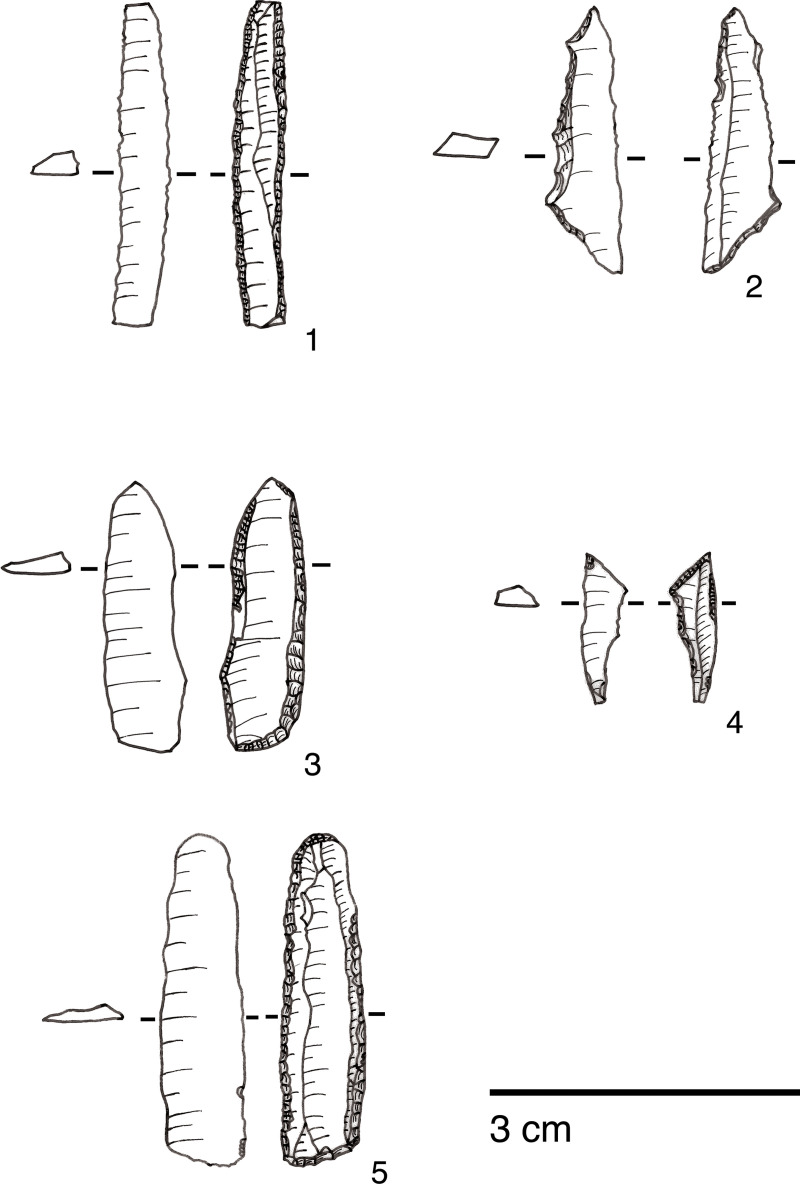
PG tools (drawings by D Baird). 1. Backed bladelet fragment (ADG/SF180); 2. Scalene bladelet (ACF/SF53); 3. Backed and obliquely truncated bladelet (ACV/SF136); 4. Scalene bladelet (ACH/SF73); 5. Backed and obliquely truncated bladelet (ACT/SF129).

The single largest category of tools in both Areas A and B, and across the Area A Phases 1–3 are non-formal tools (Fig 26) including blanks with highly varied areas of mostly marginal retouch (unilateral, direct and inverse but also bilateral, alternating or alternant). Non-formal tools comprise mainly retouched bladelets. The two other major tool categories are notched tools (Fig 27 no. 3) (often notched bladelets) and microliths (Figs 26–28). Other distinctive tool types, which are ubiquitous although found in more modest proportions, are denticulates (Fig 27 no. 6), scrapers, truncations and burins. End-scrapers are the most frequent scraper type (Fig 27 no. 5). Burins include mostly angle burins some of which were made on truncations. Characteristic Zarzian shouldered points (made on blades) (Fig 27 no. 4), backed blades (Fig 27 no. 2), shouldered pieces, piercing tools (Fig 27 no. 1) and sickle blades are also sporadically present (Fig 26).

Microliths, defined as bladelets with backing and truncations (Fig 28), are one of the most common tool types in the PG assemblage (Fig 26; see also S8–S13 Figs). They were produced on-site with the microburin technique for bladelet segmentation. Microburins form 4.03% of microliths, calculated on the basis of the restricted microburin index (rlMbt) in its broadest definition [51]. Scalene bladelets (Fig 28 nos. 2, 4) and obliquely truncated backed bladelets (Fig 28 nos. 3, 5) are ubiquitous in the assemblage, often made on *piquant trièdres* as indicated by their truncations. It thus appears probable that the microburin technique was frequently used. The application of the even more restricted index, linking microburins to the microlith types definitely manufactured by this technique (i.e., scalene bladelets, and backed and obliquely truncated bladelets) produces a maximum rlMbt of 13.04%. Since backed bladelets are unlikely to have been produced by the microburin technique, backed bladelet fragments were excluded from these calculations. If they represented fragments from backed and truncated bladelets then the matching fragments with the truncations would have been found in the relevant type categories.

Before describing the microlith characteristics in greater detail, it is necessary to clarify the relevant typological terminology in order to facilitate comparisons between different lithic assemblages, also taking into account the fact that there has been some variation in the terminology used by researchers working in different regions of SW Asia. This is especially pertinent to the application of the labels ‘geometric’ and ‘non-geometric’, and for clarifying the status of scalene bladelets. Apart from ensuring effective comparative analysis, a detailed consideration of these issues is particularly useful for evaluating the perceived increase in geometric microliths in the later part of the Zarzian previously reported in the literature [33, 34]. In the Levantine Epipalaeolithic terminology, scalene bladelets are elongated microliths where the back is created at an angle to the axis of debitage and arises on the bladelet to create a long oblique truncation along most of the tool, while a short oblique truncation completes the tool at the opposite end (Fig 28 nos. 2, 4). These tools relate closely to the backed and obliquely truncated bladelets, both important components of the PG lithic assemblage, which in the Levantine typologies are not normally classified as geometric microliths ([52]: pp.456-458, [53]: pp.86-88 & Figures 4.2–4.4, [54]: p.301). We have followed the same approach in our classification of the PG microliths. In the present study the term ‘geometric microlith’ is used *sensu stricto* to denote lunates, rectangles, trapezes, isosceles and short asymmetric triangles, excluding elongated scalene bladelets. Other studies of Zarzian lithic industries have adopted somewhat different classification schemes. Both Wahida [9–11] and Olszewski [33, 34] classify scalene bladelets as geometric microliths although Olszewski usefully separates elongated scalenes, and Wahida has separated out scalenes as well. Judging by the drawings included in the published report of the TB75 assemblage Ohnuma [55] appears to implicitly classify scalene bladelets as non-geometric microliths.

**Fig 29:**
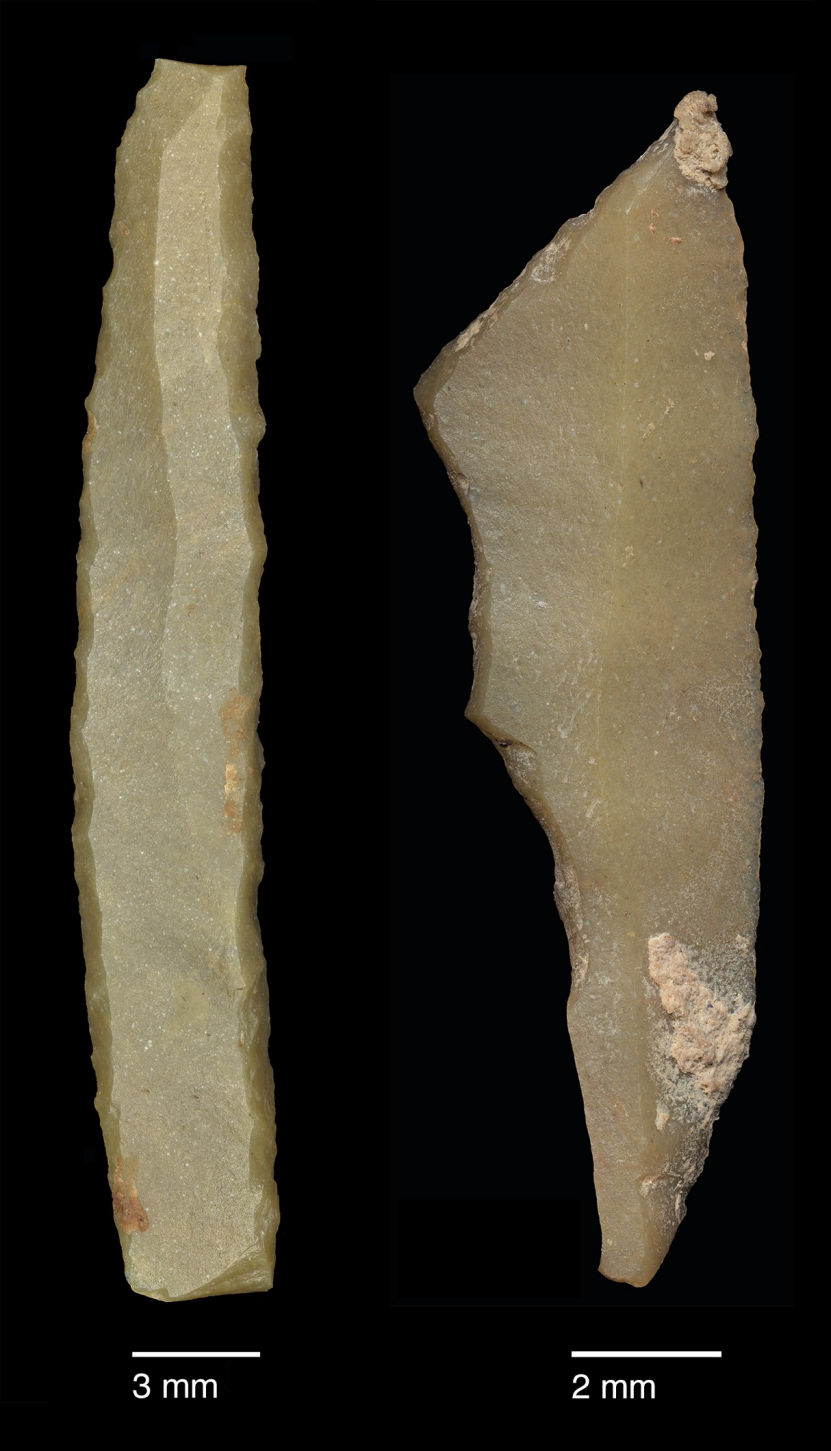
Backed bladelet fragment (left: ADG/SF180) and scalene bladelet (right: ACH/SF73) (photos by E Asouti).

**Fig 30:**
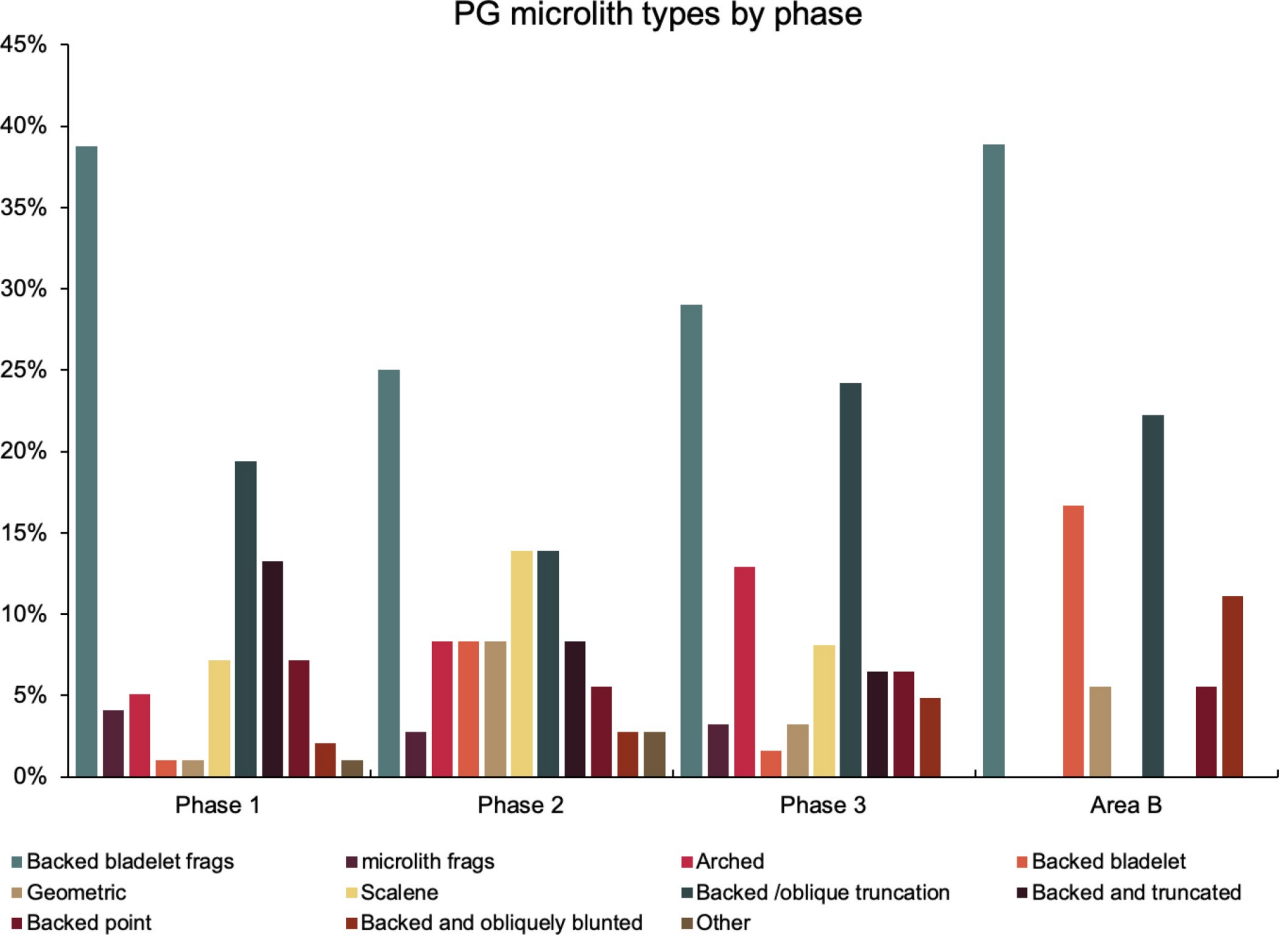
Relative proportions of microlith types in Area A (Phases 1–3) and Area B.

**Fig 31:**
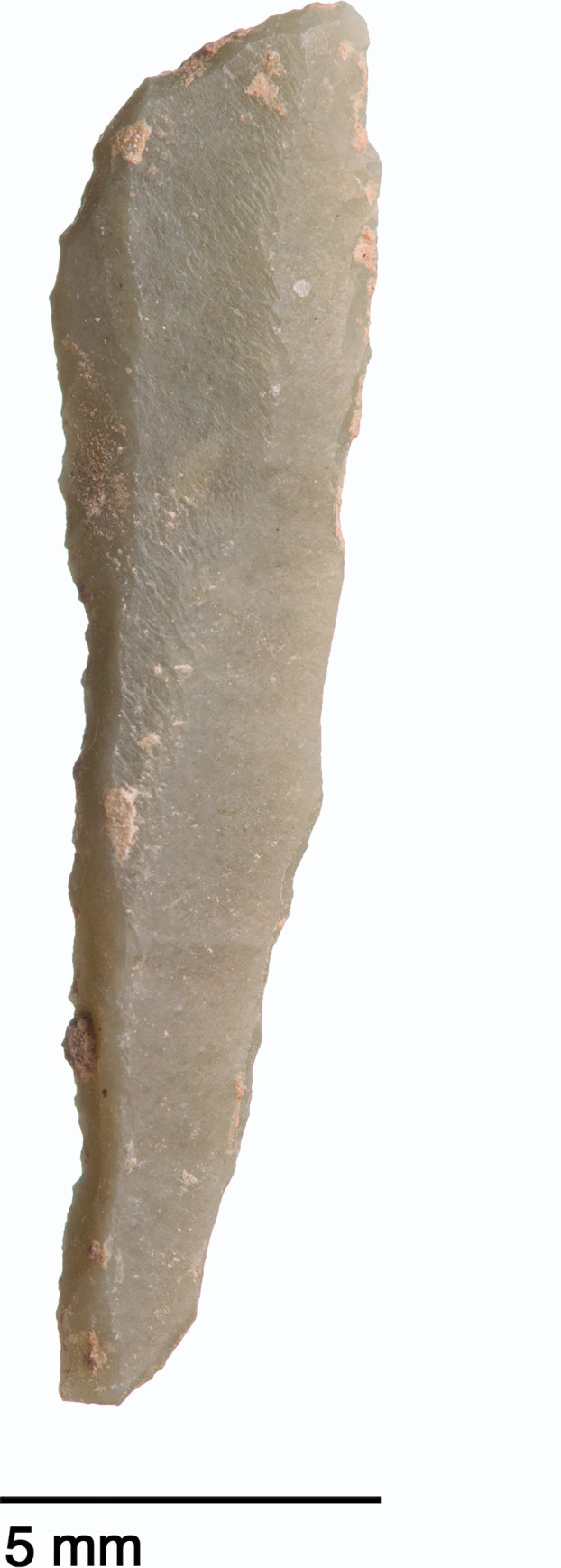
Backed and obliquely truncated bladelet from context AAM (photo by D Baird).

**Fig 32:**
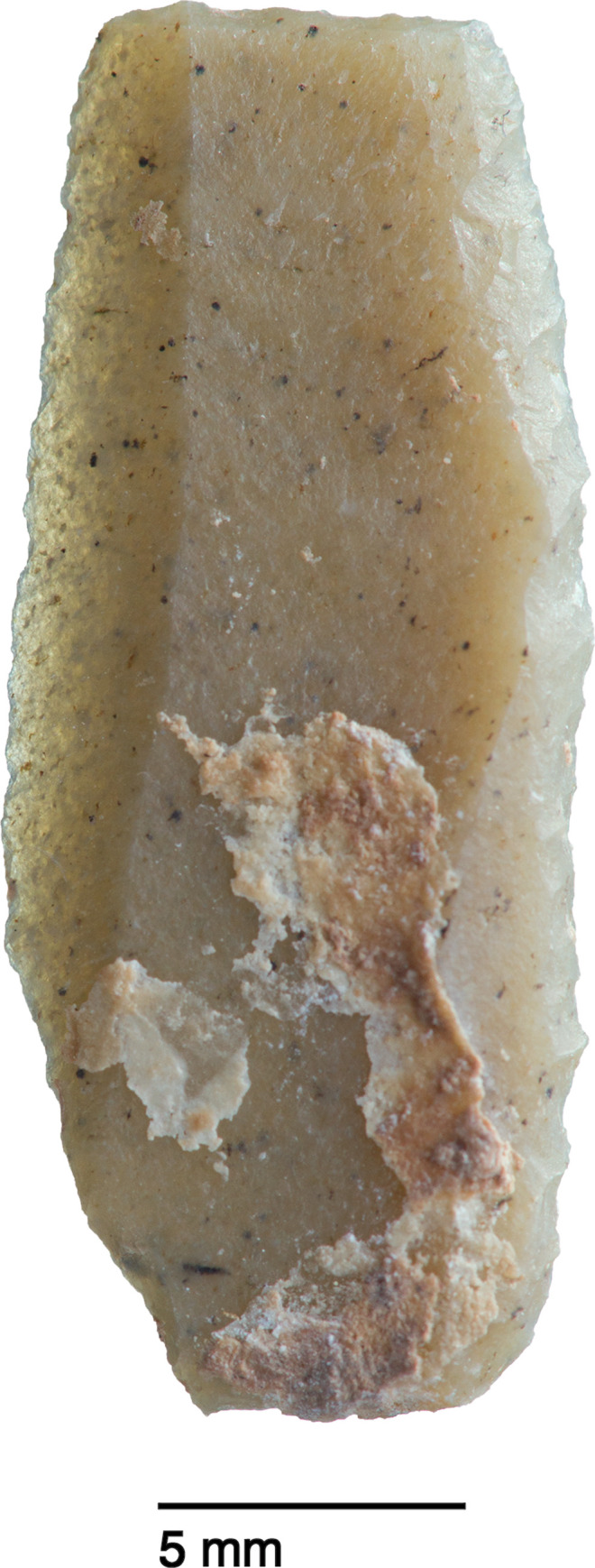
Backed and truncated bladelet from context AAL (photo by D Baird).

The single most common microlithic item found at PG are backed bladelet fragments (Fig 29) representing 25–39% of microliths (Fig 30). These were often derived from microliths with a range of truncation types, although some probably originated from complete backed bladelet tools without truncations. Complete examples of this tool type are ubiquitous amongst microliths, with frequencies ranging from 1–17% (Fig 30). Therefore, it seems likely that backed bladelets without truncations were much more common than suggested by the frequencies of complete examples. The microlith repertoire is dominated by various types of backed and truncated bladelets. Backed and obliquely truncated bladelets (Fig 31) are the most common (~14–24%) followed by backed and truncated (Fig 32) and scalene bladelets (each ~6–14%) (Figs 29 and 30 and S11 Fig). Geometrics *sensu stricto* represent only ~1–8% of the microlith assemblage. Arch-backed (S12A Fig) and backed microliths with arched truncations (S8A Fig) are also regularly present (5–13%). Backed and obliquely blunted items, where the oblique truncation forms an acute angle to the backed edge (S9B Fig) and microlithic backed points comprise ~2–11% and 5–7% of the microlith assemblage respectively (Fig 30).

#### Chronological and spatial variability

The overall technology and representation of the main debitage types demonstrate little change through time, without clear trends in evidence through the Area A Phases 1–3. Retouched tool types also occur in very similar proportions across phases. The only potentially significant, if minor, diachronic shift is attested in the modest increase in the proportions of microliths (from ~23–25% in Phases 1–2 to 30% in Phase 3) (Fig 26). However, the frequencies of most specific microlithic types do not change through time with the sole exception of backed and truncated bladelets, which decrease from ~13% in Phase 1 to 6.5% in Phase 3 (Fig 30). The frequencies of geometrics *sensu stricto*, which could be reasonably expected to increase through time according to the classic accounts of the evolution of the Zarzian industries in the literature (see [34] and references therein) fluctuate without evidence of a clear temporal trend. Although they are slightly higher in Phase 3 compared to Phase 1, they register their highest values in Phase 2 (Fig 30). On the whole, the PG Area A lithic assemblage (Figs 26–30) points to a remarkable degree of continuity in the presence of most tool types, including the main microlith types, over a period of ~6000 years.

**Fig 33:**
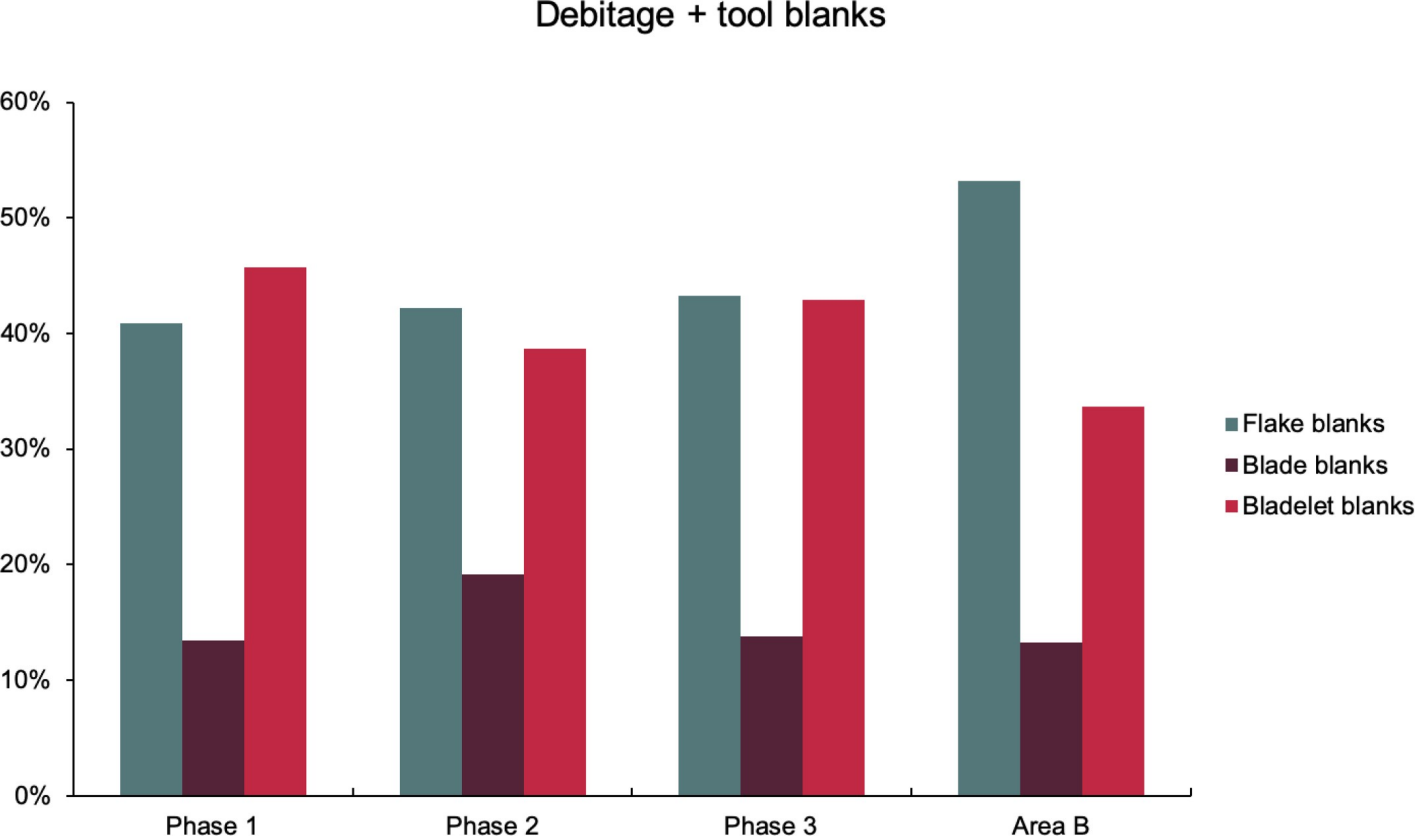
Relative proportions of combined debitage and tool blank categories from Area A (Phases 1–3) and Area B.

The clearest indicators of spatial variability in the PG chipped stone assemblage emerge when considering the lithic sample retrieved from Area B, which contains higher proportions of flakes and much fewer bladelets (unretouched debitage and tool blanks combined) (Fig 33). This is also reflected in the higher proportion of flake tools and the lower proportions of bladelet tools (Fig 25). Furthermore, microliths are notably less frequent in Area B (~13% of the retouched tools) while scrapers are more frequent (12%) (Fig 26). These differences between the Area A and B assemblages are significant. Theoretically, lithic material could have washed down from the cave onto the terrace immediately adjacent to it, in which case one could anticipate the accumulation of fairly similar lithic assemblages in both areas. However, while this might represent one mechanism for lithic deposition in Area B, the differences observed in the composition of the two assemblages suggest that it is unlikely to have been the main contributing factor. Instead, it seems possible that lithic accumulation on the terrace resulted primarily from the tossing of material from the cave entrance as part of specific activities, and/or the performance of specific (and to a degree distinct) tasks involving knapped stone use on the terrace. For example, scrapers might have been used more frequently on the terrace area. These are classic end, side and round scrapers with a semi-abrupt, often Aurignacian-style retouch penetrating quite highly on their thick edges. Although it is not possible to directly translate typological categories into functional categories, the PG scrapers would have made excellent skin/hide, wood and reed processing implements. In turn, it is conceivable that such activities were performed more regularly on the terrace further away from habitation areas. Similar patterning in the spatial organisation of activities may also explain the presence in Area B of a higher proportion of non-formal flake tools. The proportions of primary and secondary flakes are not much higher in Area B contexts, therefore greater degrees of initial core shaping and reduction are unlikely to explain this disparity. Alternative explanations include the introduction of flake blanks to the terrace for the production of flake tools, and/or the more frequent use of unretouched flakes possibly linked to the use of a higher proportion of non-formal flake tools. Future microwear analyses will help resolving these issues. Lastly, the presence of microburins and bladelets in Area B suggests that the lower proportions of microliths in this area might reflect less production and/or refurbishment of microlithic hafted tools (e.g., projectile points and/or cutting tools), less use of cutting tools, or possibly less butchery and removal of projectiles from carcasses on the terrace in front of the cave.

#### Comparison with the Iraq-Jarmo project assemblage

Although detailed comparisons are not feasible due to the absence of a full report, the Iraq-Jarmo project assemblage as described briefly by Braidwood and Howe [2] appears on the whole very comparable with the range of core and tool types present in the EFEC project chipped stone assemblage. Backed blades and microlithic bladelets, various scrapers and burin types, notched blades and flakes were especially common. The predominance of sub-pyramidal bladelet and flake cores was also noted while, as discussed already, the presence of obsidian geometric microliths and microburins point to Zarzian obsidian use and the production of tools locally from imported obsidian bladelets. An obvious point of contrast is the relatively high frequency of geometric microliths informally reported by Braidwood and Howe ([2]: p.58) especially triangles and trapezes, which was a key argument behind their attribution of the PG habitation to a ‘late Zarzian’ facies. It is clear that at least some of the Iraq-Jarmo project geometric microliths would be re-classified by our analysis as small scalene bladelets (one of the most common tool types encountered in the EFEC project assemblage) and arched backed/truncated bladelets ([2]: Plate 24). This discrepancy is thus likely to be explained, at least in part, by differences in the classification methodology used, namely the grouping of scalene bladelets with geometrics.

**Fig 34:**
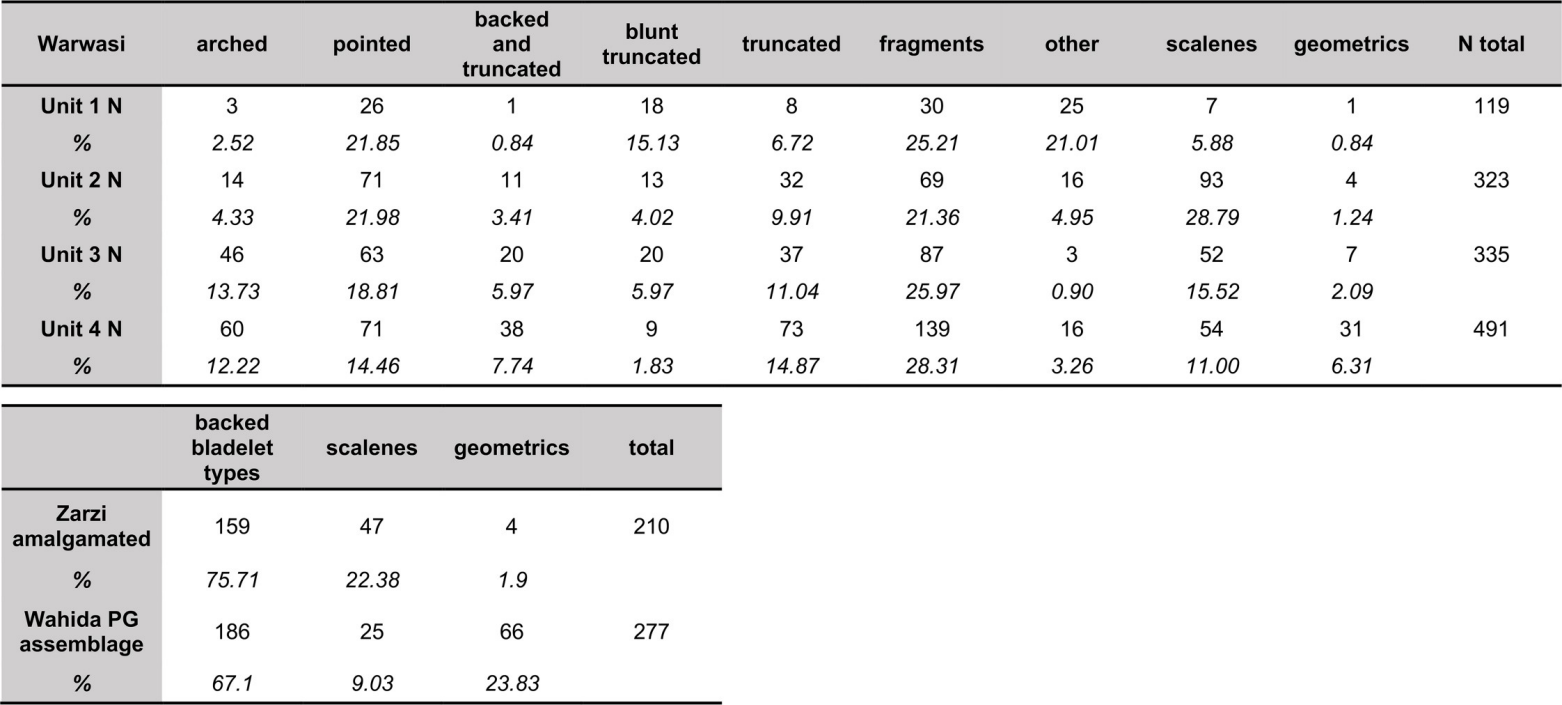
Representation of microlith categories in the Warwasi, Zarzi and PG Wahida assemblages adjusted to the EFEC project PG microlith categories. Data sourced from [9, 33].

Wahida [9, 11] re-analysed a sample of Howe’s lithics (~1500 items) stored in the Iraq Museum and in Chicago. This sample (Wahida assemblage = WA) represents a small proportion of the Iraq-Jarmo project lithics numbering >4000 chipped stone items ([2]: p.58). Its representativeness is limited, as indicated by the absence of a category equivalent to our non-formal tools and the under-representation of debitage. However, assuming that formal tools were preferentially selected for museum storage and curation, it is still possible to compare the relative proportions of formal tool categories between the WA and the EFEC project assemblage. Notches and microliths are the two most frequent formal tool types in both assemblages, with microliths being somewhat more frequent in the WA. Scrapers are nearly as ubiquitous as notches in the WA, contrasting with the EFEC project assemblage in which notches are much more common. End-scrapers are frequently found in both assemblages, while shouldered points are slightly more common in the EFEC assemblage. Burins are found in more modest quantities in both assemblages, although in the WA they are less ubiquitous compared to scrapers, which lends additional support to the impression of a higher frequency of scrapers in the WA. Another contrast between the two assemblages is the relatively high frequency of drills in the WA, although some of these drills would have been classified as backed bladelets or microlithic points by our analysis. Wahida ([9]: p.136) also reported the presence of a total of 66 geometric microliths *sensu stricto* (although possibly including some made on blades). As he reported these together with scalene bladelets, it is not possible to distinguish the depths from which the geometrics *sensu stricto* were recovered. Based on the published illustrations some of the WA backed bladelets appear to include what we would have classified as scalene bladelets; thus, the proportions of scalene bladelets are likely to represent an underestimation of this tool type. The WA backed bladelet category comprises arched backed bladelets, backed and obliquely truncated bladelets, and other backed and truncated bladelets ([9]: pp.131-143). When compared to the combined proportions of similar categories in the EFEC assemblage, the WA appears to contain slightly lower proportions of these microlith types (Fig 34) while scalenes appear in similar proportions in both assemblages. A notable contrast lies in the much higher proportions of geometrics *sensu stricto* (excluding scalenes) amongst the microliths of the WA (Fig 34) especially trapezes and lunates ([9]: pp.131-143).

The WA is not presented in granular detail by context/depth. It is also unclear if it includes material from the upper 40cm of Howe’s trench, wittingly or otherwise, although this is likely as the 0-60cm depth zone was included in the WA sample. Olszewski has previously suggested an increase in geometric microliths at Zarzi and Warwasi as indicative of a ‘late Zarzian’ facies [33] (see below, **Comparisons with other Epipalaeolithic assemblages** section). The Zawi Chemi B sequence is the best representative of a ‘late Zarzian’ type assemblage due to its (contested) radiometric dating and particularly the increasing proportions of ground stone axes in its upper levels, in conjunction with the higher frequency of geometric microliths especially of geometric *sensu stricto* lunates (~18% of microliths) ([6]: p.9 & Table 1). In this light it remains possible that the probably genuinely higher proportions of geometric microlith forms *sensu stricto* found in Howe’s trench, given also the presence of lunates, could have derived from chronologically later phases of Zarzian compared to the undisturbed sequence excavated in Area A. The 0-60cm WA microliths could have derived from undisturbed or mixed terminal Pleistocene deposits or from some disturbed early Holocene occupation. However, this is also unlikely to explain the whole of the disparity observed between the two assemblages, given that the overall WA sample came from the full depth of Howe’s stratigraphy. It is therefore also possible, if on presently available evidence unprovable, that this disparity reflects at least in part finer degrees of spatial variation in the distribution of the microlith types retrieved from Howe’s trench and the EFEC project Area A. Furthermore, although there is no evidence from stratigraphically early phases of other sites (e.g., the long if undated Warwasi and Zarzi sequences) for a significant presence of geometrics *sensu stricto* or for contemporaneous intra-site spatial variation between geometrics *sensu stricto* and non-geometrics, we also cannot exclude the possibility that such variations as presently detected at PG may reflect differences in site use.

#### Comparisons with other Epipalaeolithic assemblages

The PG assemblage fits well published descriptions of Zarzian industries, being characterised by a technology directed at predominantly single platform bladelet production with flakes as an additional production goal. There are significant proportions of microliths, at least some of which were produced with the microburin technique, bladelet tools, and notched blades and bladelets, alongside lower and more variable proportions of scrapers, burins and denticulates, and the occasional presence of shouldered points on blades.

The most obvious points of detailed comparison are the assemblages previously studied and published from Zarzi and Warwasi. Olszewski [34] has usefully compared the relative proportions of the tools found in Warwasi and Zarzi, the latter including materials from both Garrod’s and Wahida’s excavations (Fig 34). She has incorporated Dufour bladelets in her microlith category; our analysis would have re-classified at least some of them as retouched bladelets since they are not necessarily backed forms. After excluding Dufour bladelets, microliths make up 15–30% of the Warwasi and Zarzi tool assemblages, their proportions thus being directly comparable to those of PG (13–30%) (Fig 34). Scrapers, ranging from 2–13% at Warwasi and in Wahida’s Zarzi assemblage, also match the PG scraper frequencies (4–12%). Burins form 0.2–6% of the Warwasi and Zarzi assemblages, compared to 1.9–3.5% in the PG assemblage. Notches and denticulates form the most important, if broad, tool category at Warwasi and Zarzi (25–32.5% except for Warwasi Unit 2 where they represent 17% of the tool assemblage). Their proportions are again comparable to their frequencies at PG (27–34%). Backed blades are uncommon at Warwasi and Zarzi, and also rare at PG. The proportions of piercing tools found at PG are comparable to those from Zarzi, but they are slightly more frequent at Warwasi. Shouldered points are rare at Zarzi and PG, and they appear to be absent from the Warwasi assemblage [33]. Across these broad tool indices PG, Warwasi and Zarzi appear to be closely comparable assemblages.

Comparisons of the variability observed within the microlith tool category can be explored further, not least because Garrod, Olszewski, Braidwood and Howe, and Wahida have all treated the increasing frequency of geometric microliths as indicative of a ‘late Zarzian’ facies. As suggested by her illustrations, Olszewski [34] grouped elongated scalenes (our ‘scalene bladelets’ category) with geometric microliths, the latter also including Dufour bladelets. Her blunted non-geometric microliths seem to include backed bladelets (as defined by us) but also some non-backed bladelet tools. Her non-geometric point type includes backed microlithic points grouped together with other types ([33]: Figure 8.1) thus resulting in a higher proportion of this tool type compared to our microlithic backed point type. A re-adjustment of Olszewski’s categories evens out the differences observed between Warwasi and PG with regard to the proportions of arch backed bladelets, backed and truncated types, scalene bladelets and geometrics *sensu stricto* (see Figs 26–34). Scalene bladelets are the most common type amongst the Warwasi microliths, increasing from ~6% in Unit 1 to ~29% in Unit 2 and then decreasing to 11% in the stratigraphically latest Unit 4 (Fig 34). In the PG Area A assemblage, the proportions of scalene bladelets are fairly comparable (~7–14% without indications of a directional trend). Their probably low proportions in Area B are thus even more notable. Arched backed bladelets range from 5–13% in Area A Phases 1–3, and 2.5–13.7% at Warwasi (with higher proportions observed in Units 3–4). At both Warwasi and PG this might reflect a diachronic trend. The most notable contrast between the two sites lies in the proportions of backed and truncated bladelets, which at Warwasi represent only ~1–8% (increasing from Unit 1 to 4) (Fig 34) while at PG they range between 22–33% across the Area A sequence and in Area B without evidence of a clear diachronic trend (see Fig 26). While at Warwasi some equivalent items might be amalgamated in the blunt ended category, even adjusting their frequencies for this would not approximate their proportions at PG. The available data thus suggest a significant divergence in the abundance and proportions of backed and truncated bladelets between the two sites. At PG geometrics *sensu stricto* range between 1–8% without evidence of a diachronic trend. At Warwasi their frequencies are similar (~1–6.5%) (Fig 34) but, unlike PG, they increase through time with their highest proportions recorded in Unit 4. However, considering the much higher frequency of geometrics (8.33%) in Area A Phase 2 compared to the (undated) Unit 4 of Warwasi, it seems rather improbable that there is a temporal trend towards increasing proportions of geometrics *sensu stricto* for much of the Zarzian. Moreover, were scalene bladelets to be grouped with geometrics *sensu stricto* there would have been little evidence of a diachronic trend in their proportions at either site. It remains the case that a chronologically late Zarzian facies (i.e., after ~13,000 cal BP based on the Area A radiocarbon dates) may be characterised by significantly higher proportions of geometrics *sensu stricto*, specifically higher proportions of lunates, assuming that the Zawi Chemi B evidence is representative as discussed in the previous section. If upheld, this evidence overall represents significant differences with the Levantine Epipalaeolithic industries in which geometrics *sensu stricto* become important elements of microlith assemblages after ~16,000 cal BP and are dominated by lunates from ~14,500 cal BP.

The Zarzi assemblage as presented by Wahida [10, 11] comprises very generic microlith categories, essentially a backed bladelet category presumably including a wide range of backed types, scalenes, largely scalene bladelets, and geometric microliths designated as lunates. After amalgamating the Wahida and Garrod lithic samples (Fig 34) the Zarzi assemblage appears to be dominated by various forms of backed, and backed and truncated bladelets, also including a significant proportion of scalene bladelets (~22%) and very modest proportions of geometrics *sensu stricto* (~2%). It is conceivable that scalenes and geometrics *sensu stricto* increase through the Zarzi sequence, but this is far from conclusive given Wahida’s modest sample size and the limited stratigraphic control of Garrod’s excavation.

To summarise, PG displays significant affinities to the best published Zarzian chipped stone assemblages from Warwasi and Zarzi both technologically and in the types, diversity and range of the tools present. The microburin technique is a shared feature of microlithic production. A similar range of microlith types is found at all three sites, while scalene bladelets form a regular component of their assemblages through time. Olszewski [33] had speculated that the scalene bladelets and the single geometric microlith *sensu stricto* found at Warwasi Unit 1 might be intrusive, but there is no reason to hypothesize that this is the case for all the microliths recovered from Unit 1. Both types are also clearly attested in PG Area A from its earliest phases. Thus, it is likely that geometric microliths *sensu stricto* occur in small proportions (~1–8%) at all three sites.

Quantitative data are lacking for other Zarzian assemblages, therefore only broad comparisons can be drawn with the EFEC project PG lithic assemblage. At Pa Sangar, Hole and Flannery ([31]: p.159) published illustrations indicating the presence of the same range of tools and microliths as at PG, with a notable presence of scalene bladelets. At Shanidar B the Zarzian samples comprise larger tools similar to PG, including shouldered points, alongside a comparable range of microliths including scalenes, backed bladelets, backed pointed types and some geometrics *sensu stricto* ([9]: p.128). At Ghar-e Khar, Shidrang et al. [56] reported a small Zarzian assemblage dominated by blades and bladelets. These were produced on-site mainly from single-platform semi-pyramidal cores for the manufacture of notches and denticulates and, amongst microliths, backed bladelets and low numbers of geometrics, especially triangles/trapezoids. TB75 contains two Zarzian phases, Layers 6 and 5. Layer 6 (~20,000–19,700 cal BP) predates Phase 1 at PG, while Layer 5 (~18,000–14,000 cal BP) partly overlaps with the PG Area A sequence [35]. Their lithic samples are modest in size [55]. Both the debitage and the cores point to the importance of bladelet production at TB75, which is replicated at PG. Although the frequencies of retouched tools are modest, their illustrated types display similarities with tool types found at PG, with scrapers (especially end-scrapers) being an important component. TB75 microliths comprise non-geometric backed bladelet types ([55]: pp.117-119) matching the importance of these types at PG including backed bladelets, backed with oblique truncation and probably scalene bladelets ([55]: Figures 6.1 & 6.2). A notable difference with PG is the absence of geometrics *sensu stricto* even from the (chronologically) late Zarzian deposits of TB75.

The now much better-defined PG lithic assemblage combined with the new PG radiometric chronology permit identifying with greater precision the salient features of the Zarzian and its development through time. It is now clear that classic Zarzian industries were present in the NW Zagros from as early as ~19,600 cal BP (i.e., within the LGM). Bladelet production was a major technological goal, supplemented by purposive flake and, to a lesser extent, blade production. Flake production seems to have been part of separate reduction sequences as well as representing a useful by-product of blade/bladelet core shaping. Blade and bladelet production were probably continuous from the same cores, as observed at TB75 (with similarly early dates). Microliths and notched blade/bladelets are the main broad tool categories, supplemented by the regular, albeit more modest, presence of scrapers (notably end-scrapers), burins and denticulates, with a less frequent occurrence of backed blades and rare, yet distinctive, shouldered points on blades. Microliths are dominated by non-geometric types, backed and truncated bladelets and backed bladelets of various types with a regular presence of scalene bladelets (many probably manufactured using the microburin technique). Geometrics *sensu stricto* are present at PG from ~19,600 cal BP as they are in the stratigraphically early (albeit undated) deposits of the Warwasi sequence. The microburin technique was used for some microlith manufacture, although restricted microburin indices appear modest (probably between 4–13%) which seems to be a general characteristic of the Zarzian. Overall, the PG assemblage indicates that the microburin technique was in regular if modest use from the LGM in the Zagros, paralleling the situation observed in the southern Levant where it is attested from the Initial Epipalaeolithic (~24,000–21,000 cal BP) ([57]: pp.342-344). Microburin indices indicate however significant contrasts between the Levantine assemblages to which they have been applied, and the Zarzian. In the southern Levant, restricted microburin indices fall in the range of 23–84% for Mushabian and Madamaghan assemblages ([53]: Table 4.4) and 10–75% in the Azraq basin ([57]: Table 8.2).

Between ~19,600–13,000 cal BP these fundamental characteristics of the PG chipped stone assemblage remained largely unaltered. If, as it seems likely, habitation at PG continued after 13,000 BP there is much to suggest that these characteristics remained stable beyond this point in time, although there is also the possibility that geometric microliths increased somewhat as a proportion of the total microlith component. However, on presently available evidence, it is far from conclusively demonstrated that a ‘late Zarzian’ facies can be distinguished on the basis of the increased frequency of geometrics (either *sensu stricto* or including scalene bladelets). Neither Warwasi nor Zarzi suggest that this is the case, also considering the modest proportions of geometric microliths found even in Warwasi Unit 4 and in the upper Zarzi strata. The sole microlith type that seems to increase through time at both PG and Warwasi, albeit only by a few percentage points, are the arched backed bladelet forms. This resonates with Olszewski’s [34] suggestion that curved microlith forms increase from earlier to later Zarzian phases.

#### Relationships with Baradostian/Zagros Aurignacian assemblages

At present, the radiocarbon determinations for Phase 1 at PG are the earliest for the Zarzian of the northern and central Zagros. Therefore, it is worth considering questions of degrees of similarity and difference and possible continuity with the Baradostian/Zagros Aurignacian lithic assemblages (henceforth termed as Baradostian) especially with evidence of possible later Baradostian phases. Hole and Flannery [31], Wahida [11] and Olszewski [58] have all proposed that some continuity existed between the Baradostian and the Zarzian. The best examples of Baradostian assemblages come from Shanidar C [28] and Yafteh [59] where recent radiocarbon dates place their main Baradostian phases before 35,000 BP. The best candidate for a later Baradostian phase comes from the upper part of the (undated) long Baradostian sequence excavated at Warwasi (Levels P-Z, excluding R) [58]. Since Warwasi has not been radiocarbon dated its Baradostian phases may still predate its earliest Zarzian layers by several millennia.

Generally, Baradostian assemblages seem to show the same tool elements albeit with varying proportions distributed along a spectrum. Key distinctive elements include Font Yves/El Wad/Arjineh points, carinated scrapers, polyhedric/carinated burins and Dufour bladelets, the latter sometimes classified as microliths [60]. Some assemblages (e.g., Shanidar C) display a greater emphasis on flake production represented by both debitage and blanks, while others (e.g., Pa Sangar, Warwasi P-Z) include more evidence for blade/bladelet production and blanks. Higher proportions of Font Yves/El Wad/Arjineh points, carinated scrapers and polyhedric/carinated burins are associated with more flake-oriented assemblages while more backed blades, Dufour bladelets and other retouched bladelets (sometimes called microliths) are found in the bladelet-oriented assemblages [11, 28, 58, 60]. In some ways this mirrors the distinctions identified between the Levantine Aurignacian and Ahmarian industries. There may also be a chronological trend: Shanidar C has early dates, while at Warwasi the more flake-oriented assemblage is stratigraphically earlier than the more bladelet-oriented assemblage [60]. Still, the small number of known Baradostian sites cautions against drawing definitive conclusions, a point further underscored by the fact that chronologically overlapping Levantine Ahmarian and Aurignacian assemblages also demonstrate similar variability [60].

Assuming that Warwasi P-Z represents a chronologically late Baradostian assemblage, typologically more similar to the Zarzian with its significant component of bladelets and bladelet tools, it is useful to compare it with the assemblage from PG Area A Phases 1–2. Scrapers, especially end-scrapers, burins, notches and denticulates, backed blades and retouched bladelets are all important tool components in Phases 1–2. However, these represent rather generic similarities, which also occur between many Upper Palaeolithic assemblages across SW Asia and further afield. There is little evidence for more specific similarities. Carinated scrapers, polyhedric burins, Font Yves/El Wad/Arjineh points, and significant proportions of Dufour bladelets are not documented at PG. The most notable contrast between the 2 assemblages is the appearance in the early phases of Area A of a suite of microliths (backed, and backed and truncated bladelets of various forms) in relatively high proportions. The chronologically early PG Zarzian lithic assemblage includes rare shouldered points and a substantial representation of backed, backed and truncated, and scalene bladelets, which are not found in Baradostian assemblages, with a small presence of geometrics *sensu stricto*. Therefore, we conclude that, although continuity between later Baradostian and Zarzian lithic assemblages is possible, the PG chipped stone assemblage does not provide strong indications for it (see also Olszewski’s [61] recent re-evaluation of the Warwasi assemblages).

### Shell beads, ground stone and ochre

**Fig 35:**
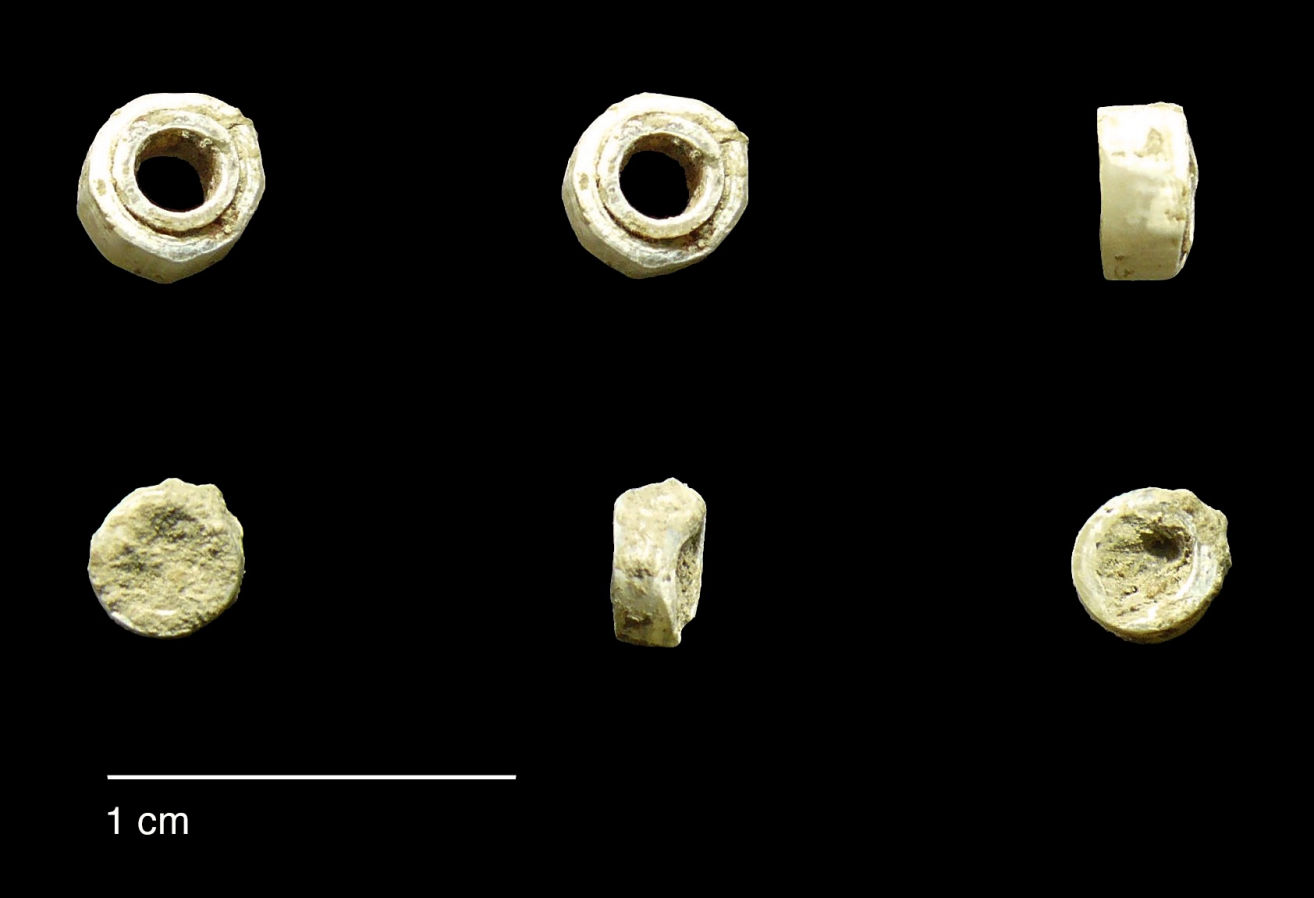
Different views of scaphopod short sections from context AAJ (photos by D Baird).

**Fig 36:**
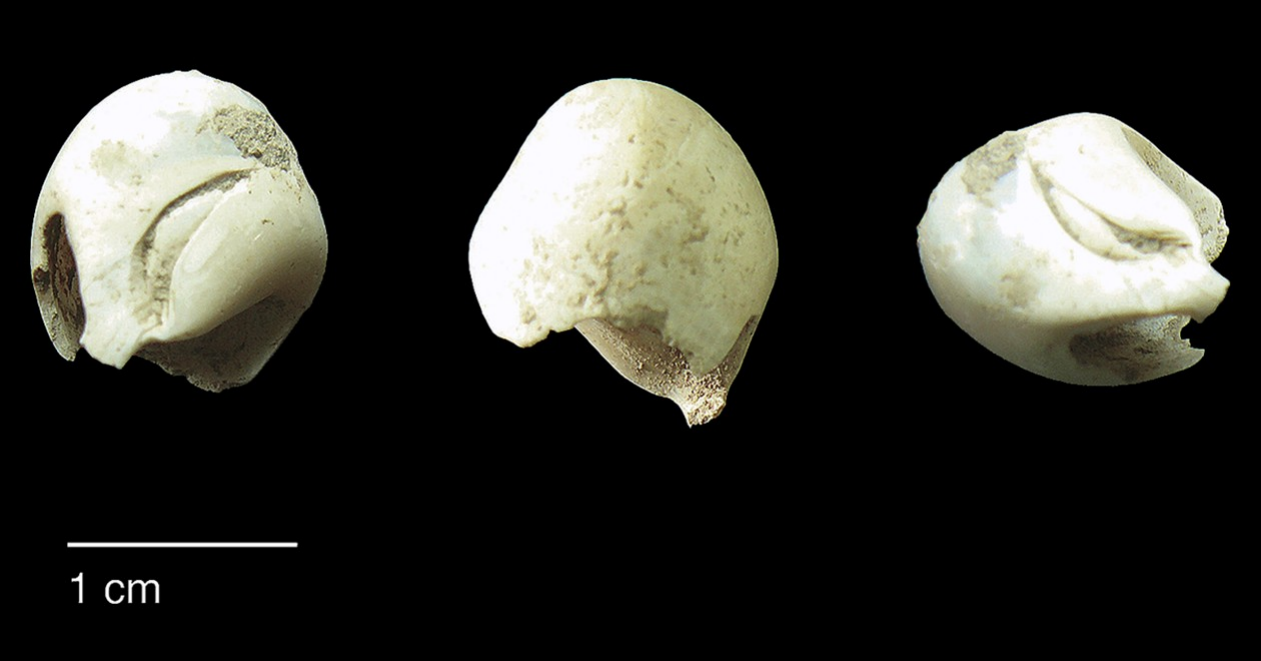
Different views of a possible *Theodoxus* sp. pierced shell from context AAJ (photos by D Baird).

The beads retrieved from the PG late Pleistocene deposits of Area A include 4 short cut segments and 2 complete/almost complete scaphopod (*Dentalium* or *Antalis*) shells: 4 short sections from context AAJ (Phase 3) one of which preserved traces of red ochre on its cut end, and 2 more complete shells from ABV (Phase 2) (see Fig 35). Another larger and possibly pierced shell (provisionally identified by Daniella Bar-Yosef as *Theodoxus* sp., a riverine taxon) was also retrieved from context AAJ (Fig 36). Detailed analysis to determine whether the scaphopod segments represent fossil shells (quite possibly distant sources) or they were instead transported to PG from coastal areas at a considerable distance (>600km also taking into account the lower sea levels during this period) is pending. The very small size of the cut segments suggests intensive curation of the scaphopod shells for maximizing the number of beads that could be produced from a limited supply of shells available for this purpose ([62]: p.618). The occurrence of scaphopod beads in secure archaeological contexts in Area A confirms the previously reported presence of marine shell ornaments in Howe’s trench ([2]: p.58). All shells were retrieved from the sorting of flotation heavy residues which is as yet incomplete, therefore it is highly likely that more such items will be retrieved. Scaphopods and other marine shells have also been previously reported from Pa Sangar in the central Zagros ([31]: p.160).

**Fig 37:**
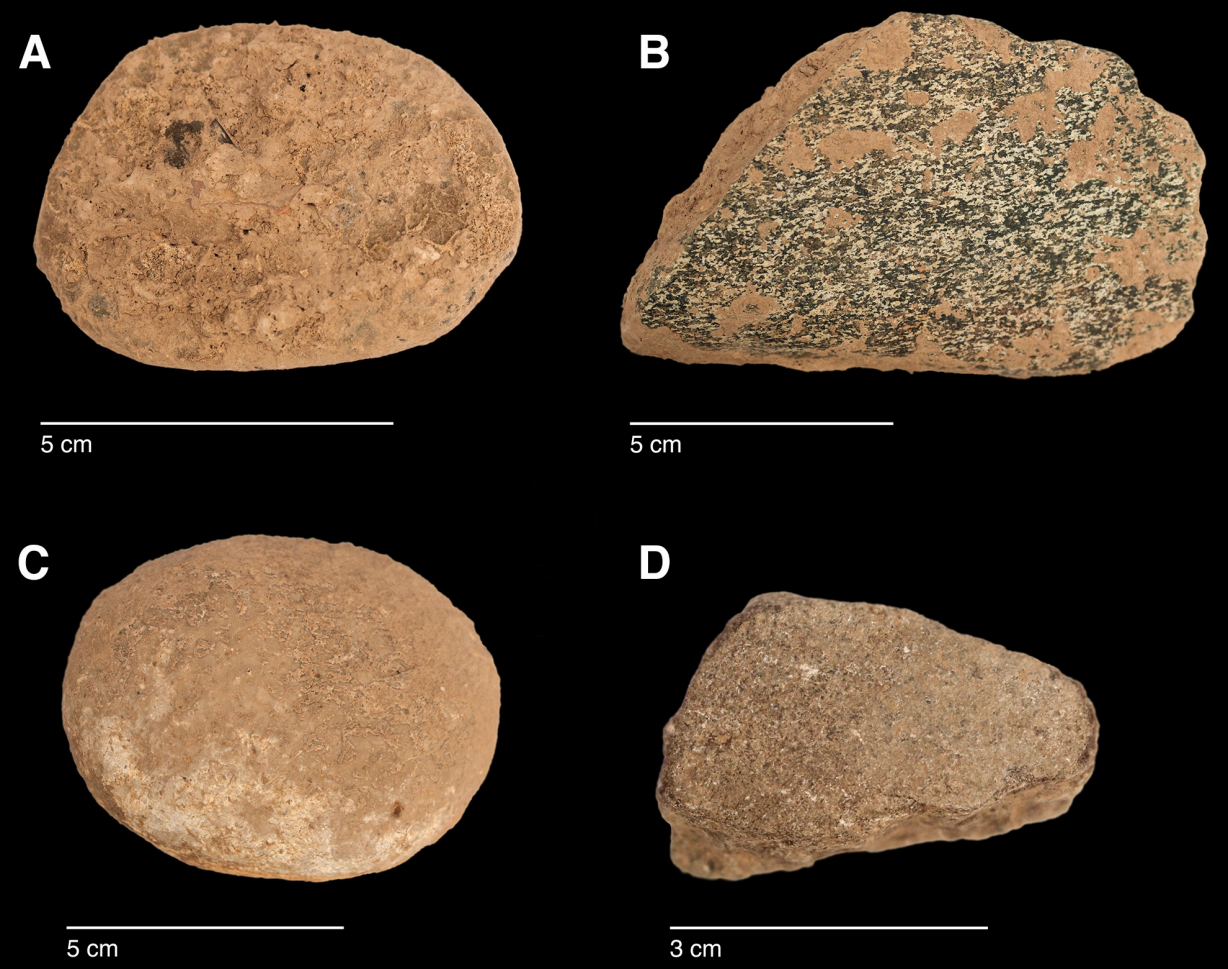
PG ground stone items (photos by D Baird). (A) Hand stone (GS5/AAO); (B) Hand stone or grinding slab fragment (GS4/AAN); (C) Hand stone (GS2/AAH); (D) Grinding slab fragment (GS8/ACX).

A modest number of ground stone implements were retrieved in the field from secure Area A contexts, including 8 artefacts and 12 possible ground stone fragments. These were manufactured from igneous and limestone raw materials, with some of the igneous rock artefacts displaying more extensive shaping through working and use (Fig 37). No sources of igneous rocks have been identified near PG. Following the typology established by Wright [63] the identifiable artefacts include 1 hand stone (Ground Stone 2 = GS2) and 1 relatively thin grinding slab fragment (GS8) made of igneous material, possibly representing a grinding kit. Additional fragments may derive from either hand stones or grinding slabs. Other items include 2 pounders and 1 edge-damaged/flaked limestone pebble alongside a number of indeterminate fragments. Both complete artefacts and the fragments that were large enough to permit reconstructing the original shape of the implements ranged in size between ~7–10 x 5-8cm thus being eminently portable. Only 2 fragments (hand stones or grinding slabs) (GS4, GS6) could have derived from somewhat larger items. Similar implements could have been used for the processing of plants, meat and minerals such as ochre. A pierced stone pendant (SF63) was also retrieved from context ABN (Phase 2).

The PG ground stone items appear in comparable frequencies and types to the materials reported from other Zarzian sites [64]. Sandstone abraders have been reported from Pa Sangar ([31]: p.160) which, although seemingly of a different raw material, could have served a similar function to some of the PG ground stone items. Grooved stones were present at Zarzi [1] but have not yet been found at PG. Similar types to those found at PG are predominant at Zawi Chemi B although its upper deposits have in addition axes, mortars and pestles, which are absent from PG, presumably an indication of the ‘final’ Zarzian nature of the Zawi Chemi B assemblage (see above and [6]).

Small fragments of red ochre were found in several contexts excavated at PG, ochre also coating some faunal elements (see below, **Zooarchaeology** section). This suggests the regular processing and use of ochre on-site both as a raw material and for coating personal ornaments such as beads, in common with behaviours widely attested among late Palaeolithic communities across SW Asia. The use of red ochre has a long history in the Zagros dating back to the Upper Palaeolithic as indicated by its presence in Baradostian assemblages in raw form and in association with grinding stones and personal ornaments [31].

### Zooarchaeology

The Iraq-Jarmo project faunal assemblage was studied and published in detail by Turnbull and Reed [65]. They reported a broad array of taxa including large and small mammals, birds and tortoise, with onager dominating the assemblage. In addition, their analysis of the faunal remains addressed questions relating to hunting practices and whether PG functioned as a short-term hunting camp or longer-term base camp. They also determined from taxon representation that the local environment was likely to have been a sparsely wooded steppe. Although comprehensive for the time, the zooarchaeological recovery methods used by the Iraq-Jarmo project do not compare to the standards common to modern excavations. In addition, only large mammalian taxa were quantified, which means that the contribution made by other faunal subsistence sources (e.g. birds and tortoise) remains relatively unknown. Taphonomy and butchery practices were also not explored in any detail.

To date, the EFEC project zooarchaeological analysis at PG has focused on the late Pleistocene sequence excavated in Area A. Building on previous work, our key objectives are to provide quantification of the taxa recovered, assess the taphonomy of the faunal assemblage and establish formation processes, and consider carcass treatment. We briefly address hunting strategies and seasonality, and discuss what taxon representation can tell us about the local environment at the time of occupation and whether there were any diachronic shifts in faunal exploitation. Lastly, we compare the PG faunal assemblage to those known from other Epipalaeolithic sites in the Zagros and provide preliminary information on culturally modified faunal elements involving worked bone and the use of ochre on bone.

The faunal assemblage studied thus far from Area A Phases 1–3 includes a NISP (Number of Identified Specimens) of 639, with a total of 18,455 bones recorded. This is a much smaller diagnostic assemblage compared to that analysed by Turnbull and Reed (NISP = 2596). The assemblage reported here derives solely from the >4mm fraction of the sieved assemblage (from both flotation heavy residues and dry sieving in the field) and represents ~80% of the contexts that were excavated. The >4mm fraction was prioritised for analysis due to the higher probability of finding diagnostic bones. Some 15 bags of it remain at the Sulaymaniyah Directorate of Antiquities and Heritage awaiting analysis. The 2-4mm fraction of the flotation heavy residues has not yet been analysed. From an initial inspection, this subset of the assemblage is likely to contribute substantially to the numbers of fish and microfauna recovered. In this paper we focus on the reporting of the macrofauna (hare size and above). More detailed analyses of the birds, fish and microfauna are planned.

#### Methods

Laboratory analysis was conducted at the Sulaymaniyah Directorate of Antiquities and Heritage and at the UCL Institute of Archaeology (IoA). The bones were initially sorted into ‘diagnostics’, ‘undiagnostics’ and ‘indeterminate’. Diagnostics are bone fragments that can be identified to taxon or element. Undiagnostic bones are those that can be identified to element group (e.g., long bone, flat bone, vertebra, rib, skull, tooth fragment) and mammal size-class, but not to taxon or element. Indeterminate bone fragments are those that cannot be identified to any degree. Due to time and export constraints, we concentrated primarily on diagnostic bones. Undiagnostic and indeterminate bones were only recorded for 16 contexts (representing approximately one third of the analysed Area A contexts).

**Fig 38:**
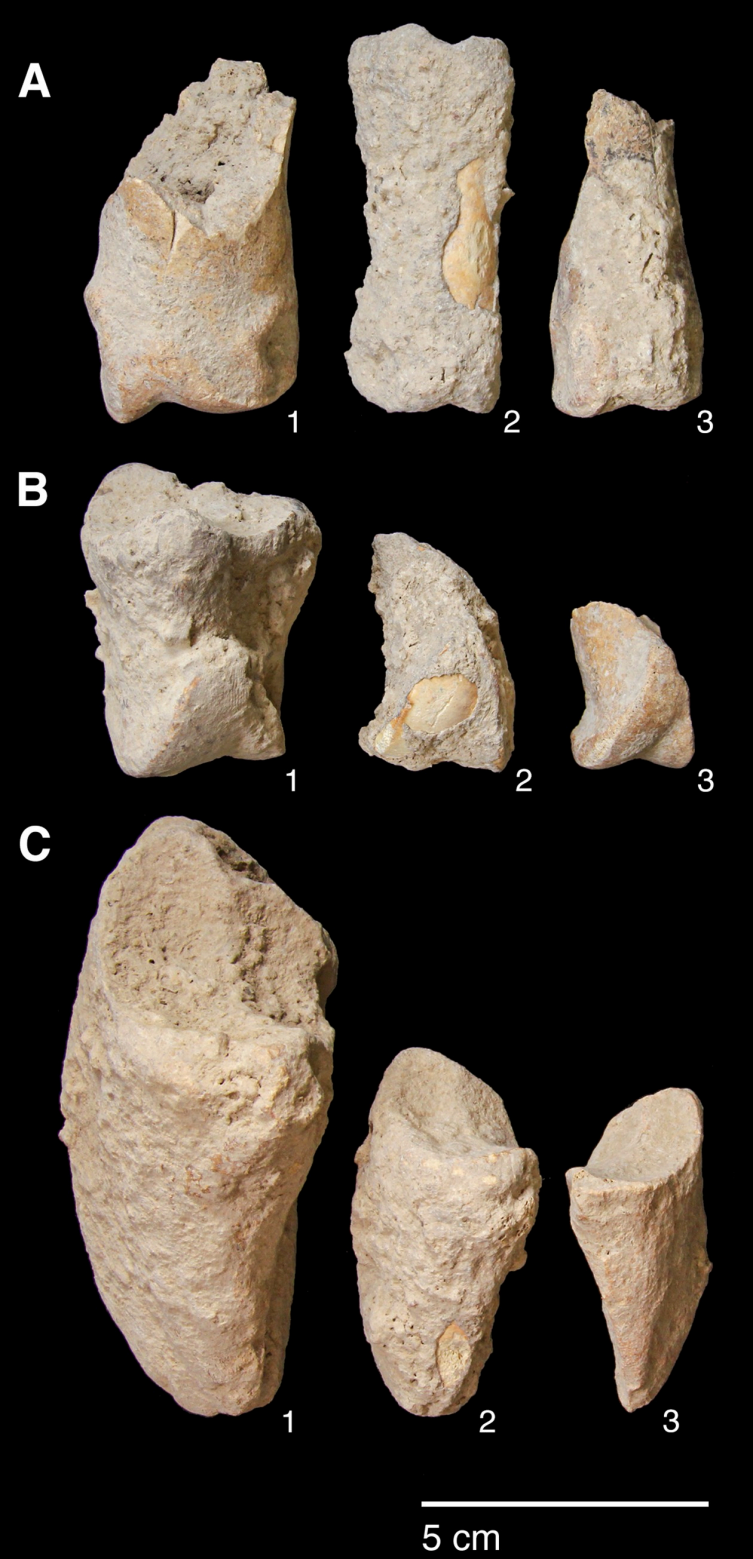
Size comparison of PG aurochs and red deer (photo by K Swinson). Aurochs and red deer phalanges demonstrating the large size difference between the two taxa: (A) Phalanx 1 (1: aurochs, 2–3: red deer); (B) Phalanx 2 (1: aurochs, 2–3: red deer); (C) Phalanx 3 (1: aurochs, 2–3: red deer).

Identification of diagnostics was primarily undertaken using the IoA zooarchaeological reference collection. Aurochs and red deer were primarily distinguished using morphological criteria. Where diagnostic features were not present, identification was based on size as there is a clear difference between aurochs and red deer at PG (see Fig 38). The equid bones have been attributed to onager (*Equus hemionus*) on the basis of comparative biometrical analyses with modern Iranian onager (S2 File and S7 Table). Red and fallow deer were separated using the criteria reported in Lister [66] and the size difference between the two species. The PG fallow deer may belong to the subspecies *Dama dama mesopotamica*; however, without appropriate diagnostics we have been unable to give a definitive identification. Sheep and goat dated to this period are certainly wild and were identified where possible [67–70]. Gazelle bones are attributed to *Gazella subgutturosa* based on knowledge of their geographical distribution and their large size rather than morphological criteria. Hare are reported here as *Lepus sp*. since northern Iraq lies in the overlap zone for both *Lepus europaeus* and *Lepus capensis*. Tortoise is assumed to represent *Testudo graeca* based on known distribution ranges.

**Fig 39:**
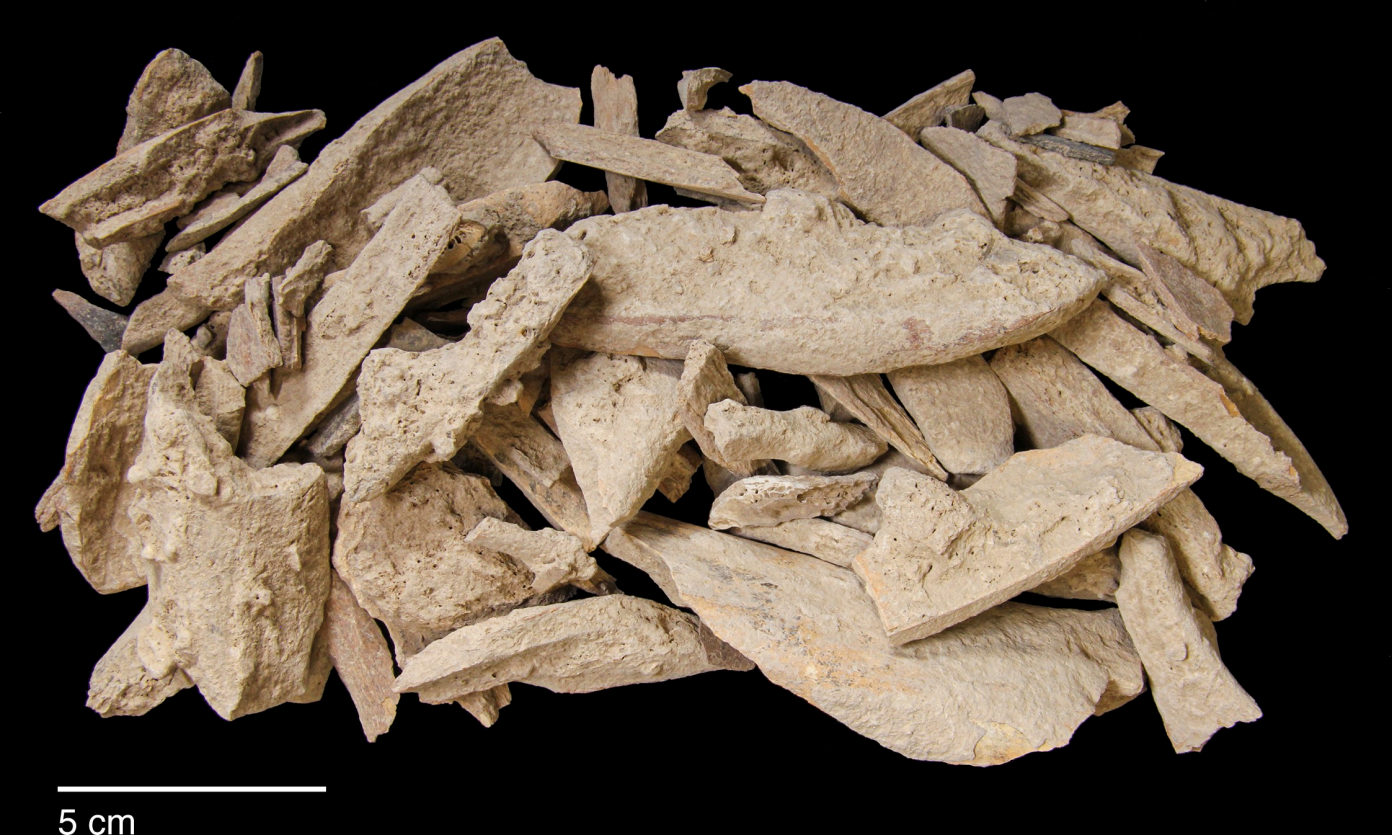
Examples of PG animal bones showing the thick lime concretions covering their surface (photo by K Swinson).

In addition to taxon recording, diagnostics were identified to skeletal element and portion. Anatomical side, burning, butchery, epiphyseal fusion, fragment size, tooth wear, weathering, digestion, working, gnawing, root etching, biometrics [71] and pathologies were also recorded. Where possible undiagnostic bones were identified to mammal size category (*Bos/Cervus/Equus*, *Dama/Sus/Ovis/Capra/Gazella* and *Vulpes/Lepus*). Limited taphonomic information was also recorded for long bones (nature of fragmentation, fragment length and burning) in order to better understand assemblage formation processes. Both taxonomic identification and the recording of bone surface taphonomy were complicated by the lime travertine concretions encasing the majority of the animal bone recovered from PG (Fig 39). The decision was made not to treat the bones with acid to remove these concretions as this can damage surface markings. This was a concern given the presence of ochre on some of the bones (see below, **Worked bone and ochre use** section).

#### Assemblage formation

**Fig 40:**
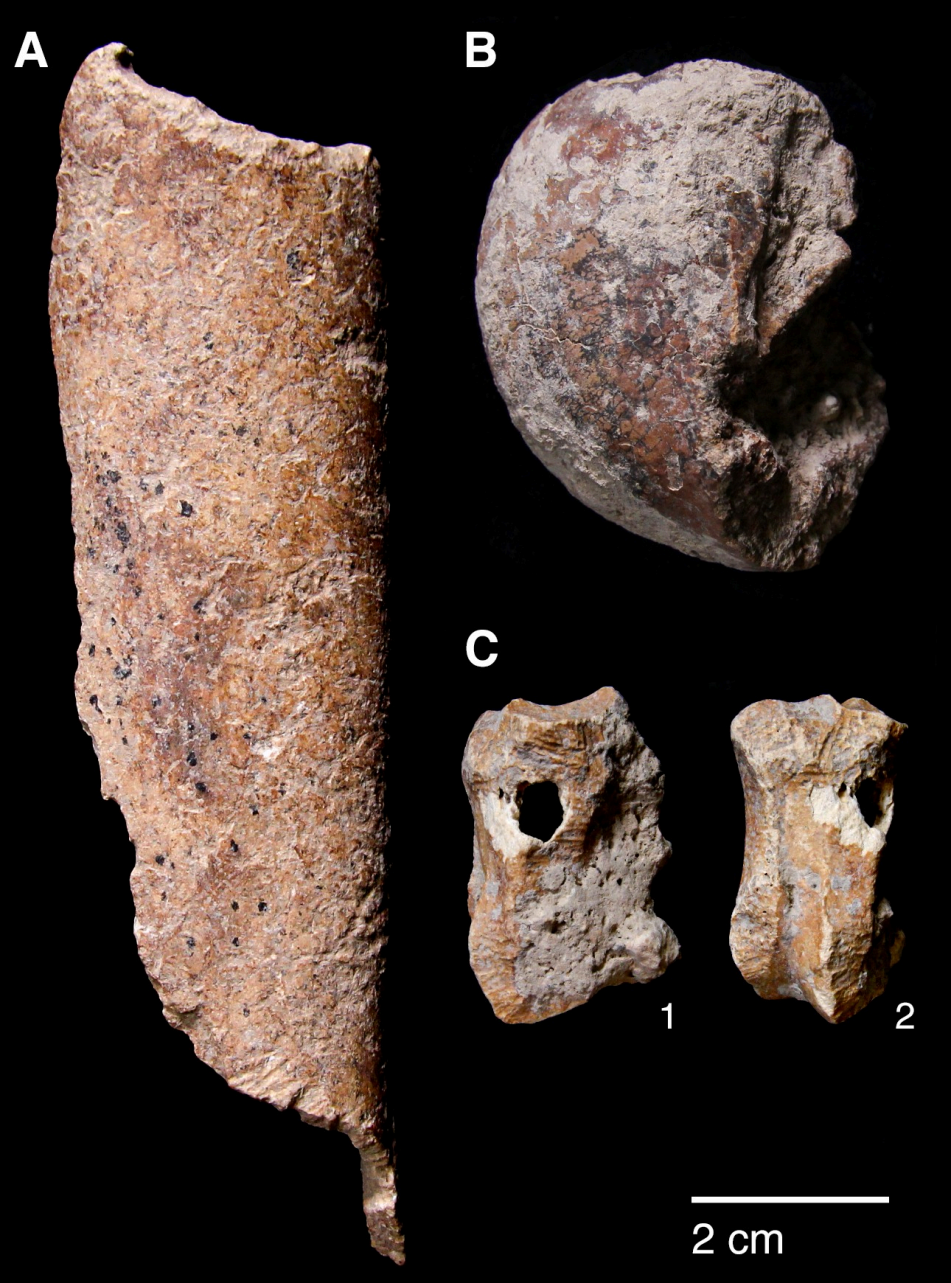
Taphonomic indicators in the PG mammal bone assemblage (photos by K Swinson). (A) Large mammal long bone fragment from AAJ exhibiting the network of small grooved lines characteristic of root etching on the surface, and rodent gnawing marks on both broken ends. (possibly from porcupine); (B) Femoral head of a *Bos/Cervus/Equus* size mammal from ABG with red staining (unlike the brown patina commonly observed on the PG bones) (C) *Capra* phalanx 2 from ACH (1: medial view, 2: posterior view) heavily gnawed by rodents.

The PG animal bone is highly fragmented, with diagnostics making up only ~1.4% of the assemblage (S8 Table). Diagnostics are generally fragmentary and include very few complete elements. High levels of fragmentation appear consistent across contexts and phases as indicated by long bone fragment sizes (S9 Table). Despite the high level of fragmentation, the bone itself is generally well preserved, with only moderate weathering present. Taphonomic indicators appear to be similar throughout Area A contexts, suggesting homogenous burial conditions across the stratigraphy. Thus far we have found only one bone fragment in context ABG exhibiting signs of a different burial environment. It was stained dark red, had an unusual texture, and was also much heavier than other fragments of similar size (see also Fig 40B). These attributes may signify exposure to water, either *in situ* or via transportation. However, the absence of round-edged water-worn bone suggests that fluvial action is unlikely to be a factor in assemblage formation.

The frequency of gnawing is very low (n = 12, <0.001%. Fig 40A–40C) even after accounting for the fact that mineral concretions on the bones may have hindered the identification of gnawing marks. The identified marks consist entirely of rodent gnawing; some appear to be relatively large and have the parallel flat-bottomed grooves and ‘windows’ in shafts characteristic of porcupine (Fig 40A) [72]. No carnivore gnawing was recorded which fits well with the complete absence of digested bones. The lack of carnivore gnawing indicates that the PG bone assemblage is anthropogenic in nature, without any sizeable contributions by carnivores which may have visited the cave. It is possible that the intensive processing of the bones for marrow extraction (see below, **Carcass Processing** section) might have rendered them less attractive to carnivores [73]. The low levels of gnawing also suggest limited surface exposure of the bones prior to burial. This is supported by other lines of evidence, including the absence of root etching (only 4 fragments, from contexts AAE, AAH and AAJ, exhibited root etching indicative of burial in topsoil; see Fig 40A) and the low level of weathering. Altogether these factors indicate limited disturbance and/or movement of bone within the Area A stratigraphy. Thus, the faunal evidence supports the stratigraphic integrity of the late Pleistocene contexts excavated in Area A.

#### Taxon representation

**Fig 41:**
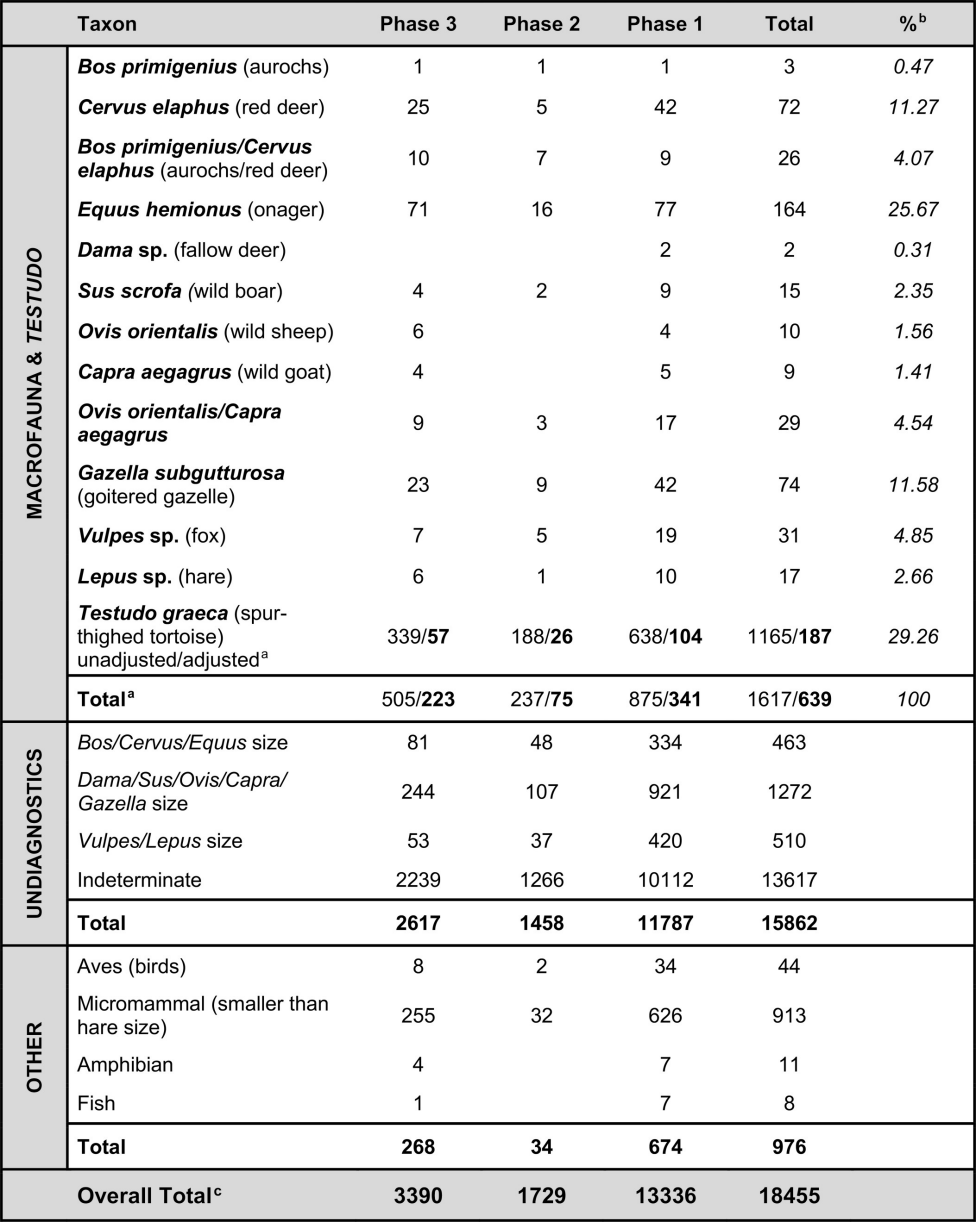
PG faunal NISP counts listed by Area A phase (Phases 1–3). ^a^Unadjusted/**adjusted** *T*. *graeca* NISP counts are listed to account for tortoise carapace and plastron fragmentation. **Adjusted** counts were calculated by dividing the total number of plastron and carapace fragments found in the given assemblage by 60 and adding limb/girdle elements following Martin et al. [74] (p.656); ^b^Taxon relative abundances (*%*) were calculated on the basis of **adjusted** *T*. *graeca* counts; ^c^Overall Total counts were calculated on the basis of unadjusted *T*. *graeca* counts.

A broad range of taxa were exploited at PG ranging from large ungulates such as aurochs and red deer, through to smaller mammals such as hare and fox (Fig 41). Onager represents the largest proportion of the assemblage with red deer, sheep/goat and gazelle making up smaller but sizeable components. Fox, hare and wild boar also contribute small but meaningful proportions, while aurochs and fallow deer are present in very low numbers. Sheep and goat are evenly represented (10 sheep and 9 goats). Of the non-mammalian taxa, tortoise is present in very large numbers with the unadjusted fragment count making up >70% of the combined large mammalian and tortoise assemblage. Even after adjustment the tortoise count is sizeable (~29%; Fig 41). The condition, patination and taphonomy of tortoise bone are similar to mammal bone. For these reasons we conclude that tortoise bone very likely represents human food waste and, consequently, that tortoise played an important role in faunal subsistence at PG. Microfauna bone fragments are also present in large numbers and, although they have been counted, they have not yet been fully analysed. There are just over 100 mandibles and maxillae which are identifiable to species and will contribute, once analysed, important information on the local environment. Of the remaining taxon groups, birds and amphibians are relatively rare. Fish bone counts are also very low, although a preliminary inspection of the 2-4mm fraction suggests that more may be present. Although not included in Fig 41, a small number of crustacean and mollusc fragments have also been recovered.

#### Ageing data, hunting strategies and seasonality

Ageing data are scarce due to the small size of the assemblage and its high degree of fragmentation. Tooth wear analysis was not feasible due to the lack of preserved mandibles. However, in terms of epiphyseal fusion there is a high proportion of fused to unfused skeletal elements (S10 Table) suggesting that the majority of hunted mammals were adults.

We can determine a little more about onager. In terms of hunting strategies, onagers were hunted primarily as adults (S10 Table) with just one element belonging to an individual under one year old. The equid crown heights too (S11 Table) show the culling of prime aged adults of ~7–9 yrs old, but also older adults of 10+ yrs. A re-analysis of equid crown heights from Turnbull and Reed’s assemblage by Bakken [75] also found hunted equids to be of prime age, a strategy interpreted as targeted selection rather than ‘whole-herd’ culling which would predict more juvenile deaths. Bakken argues for the hunting of primarily female groups with occasional juveniles in the herds and the Area A onager assemblage would fit this interpretation too. Onagers generally exhibit ‘type 2’ equid behaviour [76] whereby adult males hold seasonal territories in rich grasslands during the mating season which adult female groups range across, while younger male ‘bachelor’ groups herd elsewhere. Group size fluctuates seasonally with higher densities forming on grassy plains in early-mid summer. Modern onager herds in Iran inhabit grassy plains for much of the year, but large groups move into the more sheltered hill-valleys during harsh winters and spring to avoid strong winds and rain [77]. While we lack any seasonal information for the PG culls, a likely hunting strategy would have been to await onager herds entering the Bazian valley in winter/spring, after the mating season and before summer foaling. Trapping these fleet-footed animals may have been easier in an enclosed valley than on the open plains, and it would be closer for taking whole carcasses back to the cave for processing (see below **Skeletal part representation** section).

Firm seasonality evidence is lacking, so suggestions here are highly speculative. But while gazelle fusion data lack resolution (S10 Table) the presence of infants (<7 months old) and possibly yearling animals (<18 months old) amidst an otherwise adult cull may be found in winter/spring hunting too. Also of interest to the question of the seasonality of resource procurement and hence occupation at PG, is the high number of tortoise bones found in the assemblage. In areas with cold winters *Testudo graeca* hibernates in below-ground burrows to inhibit energy and heat loss [78]. Sadeghi and Torki’s study of present-day *T*. *graec*a in the Zagros mountains [79] reports tortoises to hibernate from mid/late autumn through winter, emerging for activity in spring. Tentatively, this points to spring/summer collection of tortoises. Considered together the onager and tortoise evidence narrows down the possible seasons of occupation at PG to the spring and summer months.

#### Skeletal part representation

**Fig 42:**
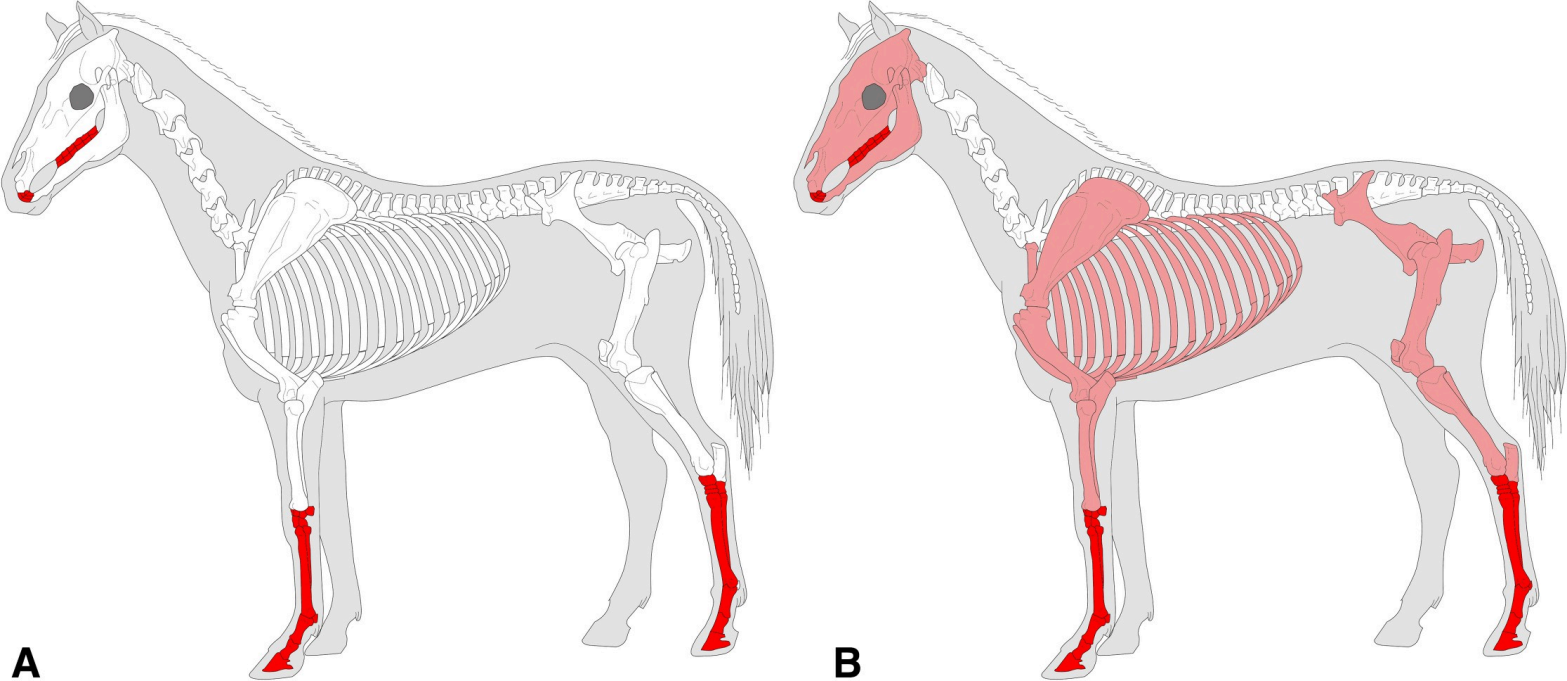
Skeletal part representation at PG. (A) Elements (teeth, metapodia and phalanges; marked in dark red) that are very well represented by ungulate diagnostic bones (aurochs, red deer, onager, fallow deer, wild boar, sheep, goat and gazelle) (see also S11 Table). The white areas signify comparatively low or absent element representation; (B) Elements represented by ungulate diagnostic bones (dark red) and undiagnostic bones (light red) of large and medium sized mammals (see also S12 Table) (image adapted from [80]: p.21).

Ungulate diagnostics (aurochs, red deer, onager, fallow deer, wild boar, sheep, goat and gazelle) are dominated by loose teeth, metapodia and phalanges (see Fig 42A and S12 Table) leaving much of the carcass comparatively unrepresented. On the basis of diagnostic bone representation alone PG could be interpreted as a kill or primary butchery site, with the meat-rich bones of the limbs and trunk removed and consumed off-site. However, this interpretation is refuted once the undiagnostic bones are considered; long bones, ribs, tooth fragments, flat bones and cranial fragments are all well represented (see Fig 42B and S13 Table). Although vertebrae are very infrequent (numbering only 7 out of the 2104 undiagnostic bones recorded at PG) it is highly unlikely that their absence reflects a ‘true’ pattern. Instead, it probably represents the combined result of the poor preservation of low-density vertebrae and the inherent difficulty of identifying vertebrae fragments in highly fragmented assemblages. Vertebrae fragments are very likely to be amongst the 13,617 indeterminate bones that could not be identified to any specific element group (S7 Table).

The representation of all body elements at PG indicates that complete carcasses were brought to the site. Element representation is similar across all mammal size categories, suggesting that all carcasses (even of large ungulates such as red deer and onager) were transported whole and were processed and consumed on-site (see also S13 Table). Given the weight of these larger animals, it is highly likely they were hunted in close proximity to the cave. The proportions of different element types within the undiagnostic bone also remain relatively stable through the Area A sequence, suggesting that whole carcasses were brought back to the cave for processing throughout its late Pleistocene habitation.

#### Carcass processing

Evidence of butchery is common throughout the PG assemblage. The sole processing activity identified is marrow extraction. There are no cut marks clearly representing the dismemberment, filleting or skinning of carcasses, although it is possible that the concretions covering the bone surfaces have hindered the identification of such activities. In contrast, it is easy to recognise bones that have been broken open for marrow extraction even with the presence of concretions.

**Fig 43:**
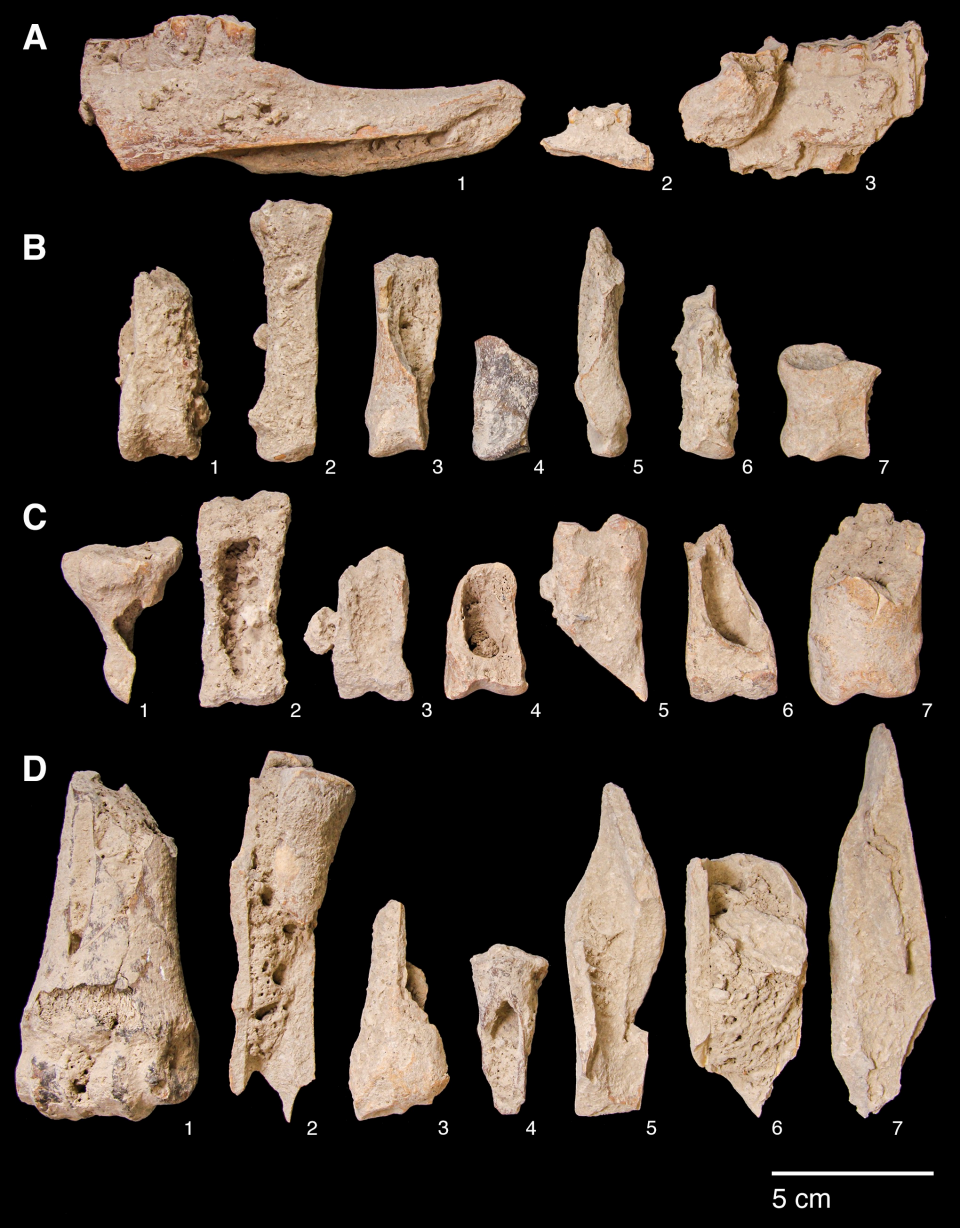
Common butchery patterns in the PG faunal assemblage indicative of marrow extraction (photo by K Swinson). (A) Chops along the horizontal ramus of the mandible (1: red deer, 2: gazelle, 3: onager); (B) Longitudinal chops along the medial line of onager lower limb bones (1: metapodial, 2–6: phalanx 1, 7: phalanx 2); (C) Aurochs and red deer phalanges variously chopped (1, 4: red deer phalanx 2 diagonal longitudinal chop, 2: red deer phalanx 2 lateral longitudinal chop, 3: red deer phalanx 1 lateral longitudinal chop, 5: red deer phalanx 1 diagonal chop, 6: red deer phalanx 1 spiral fracture, 5: aurochs phalanx 1 spiral fracture); (D) Spiral fractures and fresh breaks in long bones (1: red deer distal metapodial, 2: onager proximal metapodial, 3: sheep/goat distal radius, 4: boar proximal radius, 5–7: *Bos/Cervus/Equus* size long bone fragments).

The clearest evidence of marrow extraction occurs on the bones most commonly found in the assemblage: mandibles, metapodia and first and second phalanges. The majority of red deer and onager first phalanges display evidence of marrow extraction. Many of these elements have been broken open longitudinally, often splitting the bone in half along the shaft (see Fig 43 for examples). Long bone shaft fragments are also abundant, despite the relatively low number of long bone diagnostics recorded (S9 Table). The fracture surfaces of the vast majority of bones indicate that they were broken while fresh, with many also exhibiting the classic ‘spiral fracture’ of marrow extraction [81] (see Fig 43D for examples). This suggests that not only the diagnostic elements of the mandible, feet and toes were processed but also the marrow-rich long bones. The high levels of long bone fragmentation and the absence of diagnostics for these elements probably resulted from the high intensity of long bone marrow processing, in a pattern very similar to that observed at the contemporaneous site of Kharaneh IV in the Azraq basin in Jordan [82–84]. The PG evidence for the intensive processing of carcasses for marrow extraction is in concordance with taphonomic studies of several southern Levantine Epipalaeolithic assemblages [82–84]. The relatively consistent fragment sizes of the long bone shafts found within and between different contexts at PG suggest that marrow extraction was routinely practiced and was systematic in its application (S9 Table) [85].

**Fig 44:**
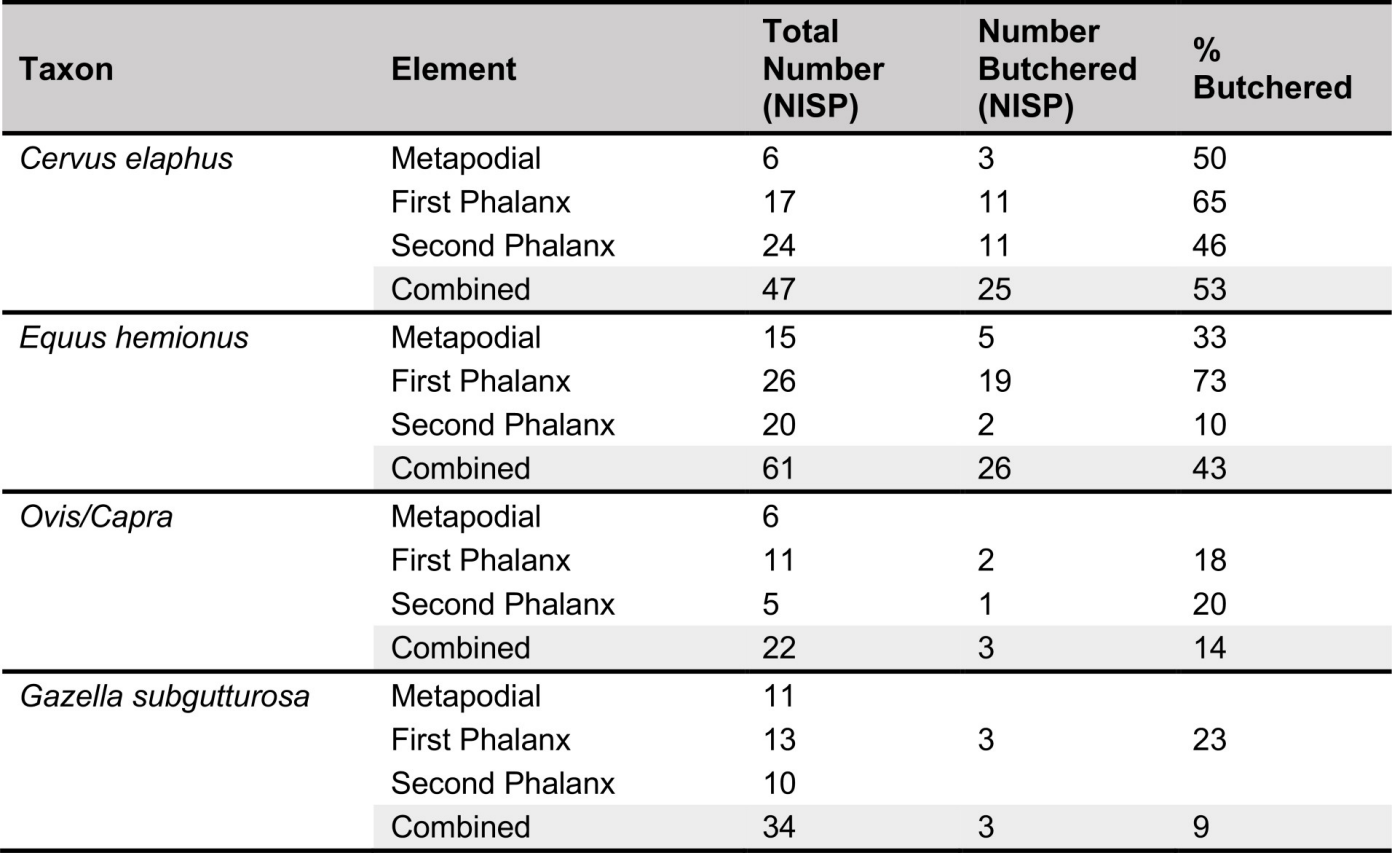
Frequency of butchery (marrow extraction) of the metapodia, first phalanges and second phalanges of the 4 main ungulate taxa.

The intensity of marrow processing at PG varies between taxa and skeletal elements (see also Fig 44). The metapodia and phalanges of the larger taxa–onager and red deer–were much more commonly processed for marrow (43% and 53% respectively) compared to the smaller sheep/goat (14%) and gazelle (9%). In terms of elements, the first phalanx was most commonly butchered, followed by the metapodia and the second phalanx. Bone marrow content varies by taxon and element [73, 84, 86]. Unsurprisingly, the bones of larger taxa contain a greater amount. Similarly, the larger long bone elements contain more marrow compared to the smaller metapodia and phalanges. Using this framework, the differential rates of marrow extraction between taxa and elements can be interpreted through the lens of rate of return. That the PG hunter-gatherers preferentially targeted bones with higher marrow yields is demonstrated by three lines of evidence: (1) the high levels of fragmentation of the marrow-rich long bones, (2) the focus on processing the bones of the larger taxa, and (3) the preference for breaking open the first phalanx over the second phalanx (see also Fig 44). Low-yield elements (i.e., the second phalanx of smaller ungulates) were almost completely ignored. This pattern contrasts with that observed in several Epipalaeolithic southern Levantine sites where the second phalanx of gazelle was broken open for marrow, despite its low yield [83]. However, the comparatively low level of breakage of the relatively high-yield metapodia observed at PG does not fit with the general pattern of ‘optimal’ marrow extraction [73, 84, 86]. This deviation can be explained once processing effort is taken into account. Due to the relative ease in which marrow can be extracted from phalanges, they can be interpreted as having a higher rate of return compared to the metapodia [73, 87].

Intensive marrow processing such as observed at PG is typically interpreted as a signal of resource stress due to the effort expenditure necessary to break bones open [86, 88]. However, marrow extraction is near ubiquitous amongst modern and historical hunter-gatherers and has been demonstrated to represent a cost-effective and important source of subsistence [83, 86, 89]. The high fat content of marrow makes it highly palatable. Marrow has more than twice the calorific value of carbohydrate and protein food sources [90] while it is also relatively easy to store and can be used to preserve other meat and plant foodstuffs extending their shelf life by months [91, 92]. Marrow extraction from low-yield phalanges has also been interpreted as a strong indicator of resource stress [73, 84, 86, 88]. However, several experimental studies have shown that phalanges are relatively easy to open and can therefore result in a higher rate of return compared to more typically marrow-rich long bones [73, 87]. In addition, the marrow of phalanges and metapodia has a higher oleic acid content [93] and is thus considered “more nutritious, storable and reportedly even tastier” ([86]: p.24). With these factors in mind, researchers have pushed back against the notion that intensive marrow extraction should be viewed as an indicator of resource stress [87]. The PG hunter-gatherers did not process every single element that contained marrow. Instead they appear to have struck a balance between processing effort and nutritional return. The absence of resource stress is supported by other lines of evidence. Hunted animals were primarily mature adults, with no sign of the shift to juveniles expected during periods of resource stress [94]. In addition, there is no evidence of a change in prey species choice that could have been induced by overhunting (see below, **Diachronic trends in taxonomic representation** section).

#### Habitat inferences

**Fig 45:**
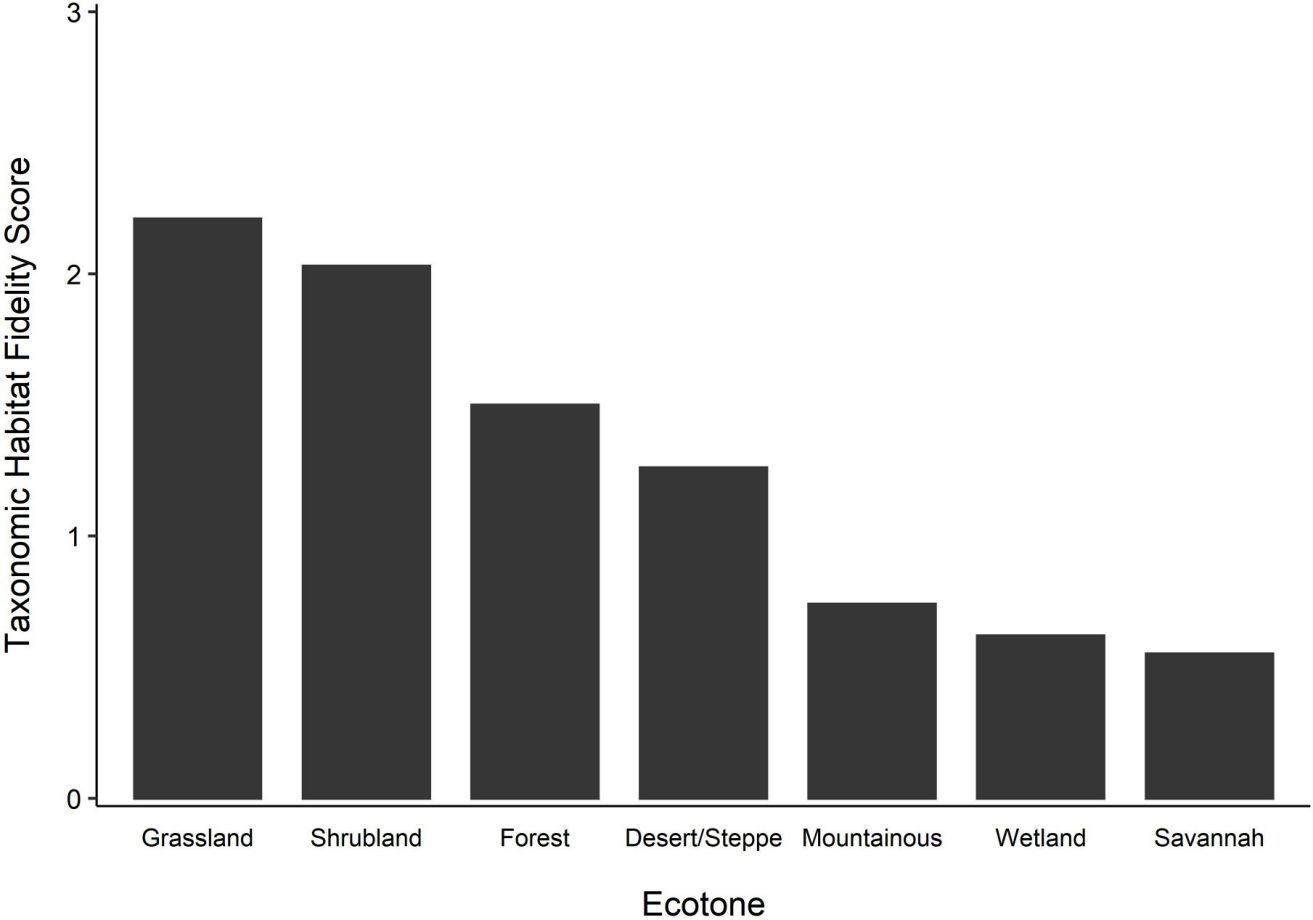
Taxonomic habitat fidelity scores of the PG faunal assemblage.

Using the large mammalian species present in the PG assemblage it is possible to reconstruct the range of ecotones that were present within hunting distance from the cave. All identified mammalian taxa were assigned a ‘habitat fidelity score’ [95] representing their current dependence on various habitat types. Each taxon was given a score of 1, which was split across the different ecotones (grassland, wetland, etc.) based on its ecological preferences. These scores (S14 Table) were assigned based on the faunal habitat types reported for each species on the ‘IUCN Red List of Threatened Species’ website [96]. Taxon scores were then added together for each ecotone in order to generate overall habitat fidelity scores for the entire assemblage. The results of this analysis demonstrate that the PG faunal assemblage is associated with a broad range of habitats (Fig 45). The late Pleistocene hunting range was ecologically diverse with ecotones ranging from forest and grassland through to wetland and desert/steppe habitats. This suggests that the PG hunters were adept at exploiting fauna encountered in different environments with a particular emphasis on grasslands and shrublands which were likely proximate to the site.

#### Diachronic trends in taxonomic representation

**Fig 46:**
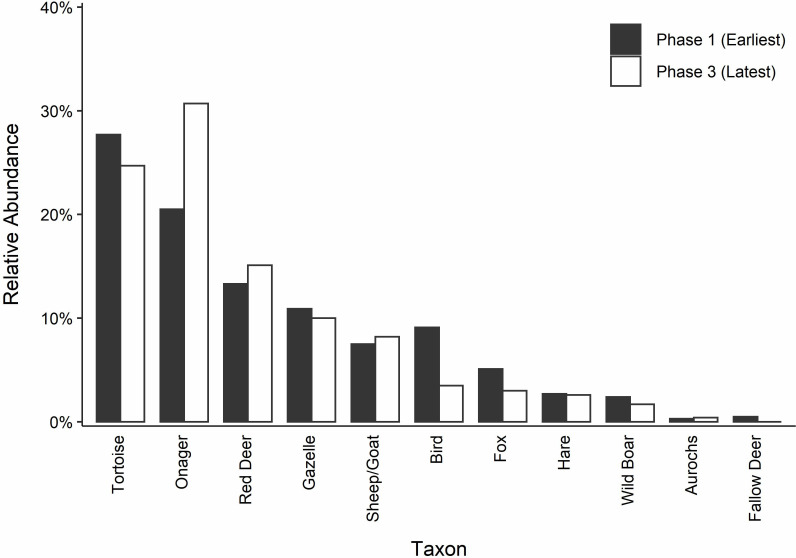
Relative abundances of taxa in Area A Phase 1 (NISP = 375) and Phase 3 (NISP = 231). Relative taxon abundances were calculated from the NISP counts of the Area A Phases 1 and 3 faunal samples on the basis of adjusted tortoise counts (see also Fig 41). The aurochs/red deer category was combined with red deer. Phase 2 NISP counts were excluded from this analysis due to the small size of the Phase 2 faunal sample.

The diagnostic assemblage of Phase 2 (n = 77) is much smaller than those of Phase 1 (n = 375) and Phase 3 (n = 231). For this reason, we have focused on comparing the stratigraphically and chronologically earliest Phase 1 to the latest Phase 3 in order to explore temporal shifts in faunal exploitation. For the majority of taxa there is very little change in abundance between the two phases (see Fig 46). Clear exceptions are the increase in onager (by ~10%) and the decrease in birds (by ~5%). These shifts contrast with the trends identified at several Epipalaeolithic southern Levantine sites where the proportions of ungulates decrease through time as birds increase [97, 98]. Following the precepts of the Broad Spectrum Revolution (BSR) [98, 99] such trends have been linked to resource stress whereby low-return birds are increasingly incorporated to the diet due to the progressive overhunting of high-return ungulates. At PG the reverse trend is observed: large-bodied onagers *increase* through time as birds decline. This suggests that the classic BSR framework does not apply; there is no evidence that the Zarzian hunter-gatherers occupying PG overhunted the locally available mammalian fauna. One possible explanation for the observed increase in onager during Phase 3 is that it may reflect climatic impacts on onager availability in the landscape. Phase 3 is dated to the warmer and more humid GI-1 (Lateglacial). Equids, including onagers, are large herbivores characterised by high water requirements and a preference for grassland habitats [100]. The more humid conditions of the Lateglacial are thus likely to have favoured onager population expansion, which could have prompted a shift away from fowling towards a greater focus on high meat-yield onager hunting.

#### Comparison with the Iraq-Jarmo project PG faunal assemblage

**Fig 47:**
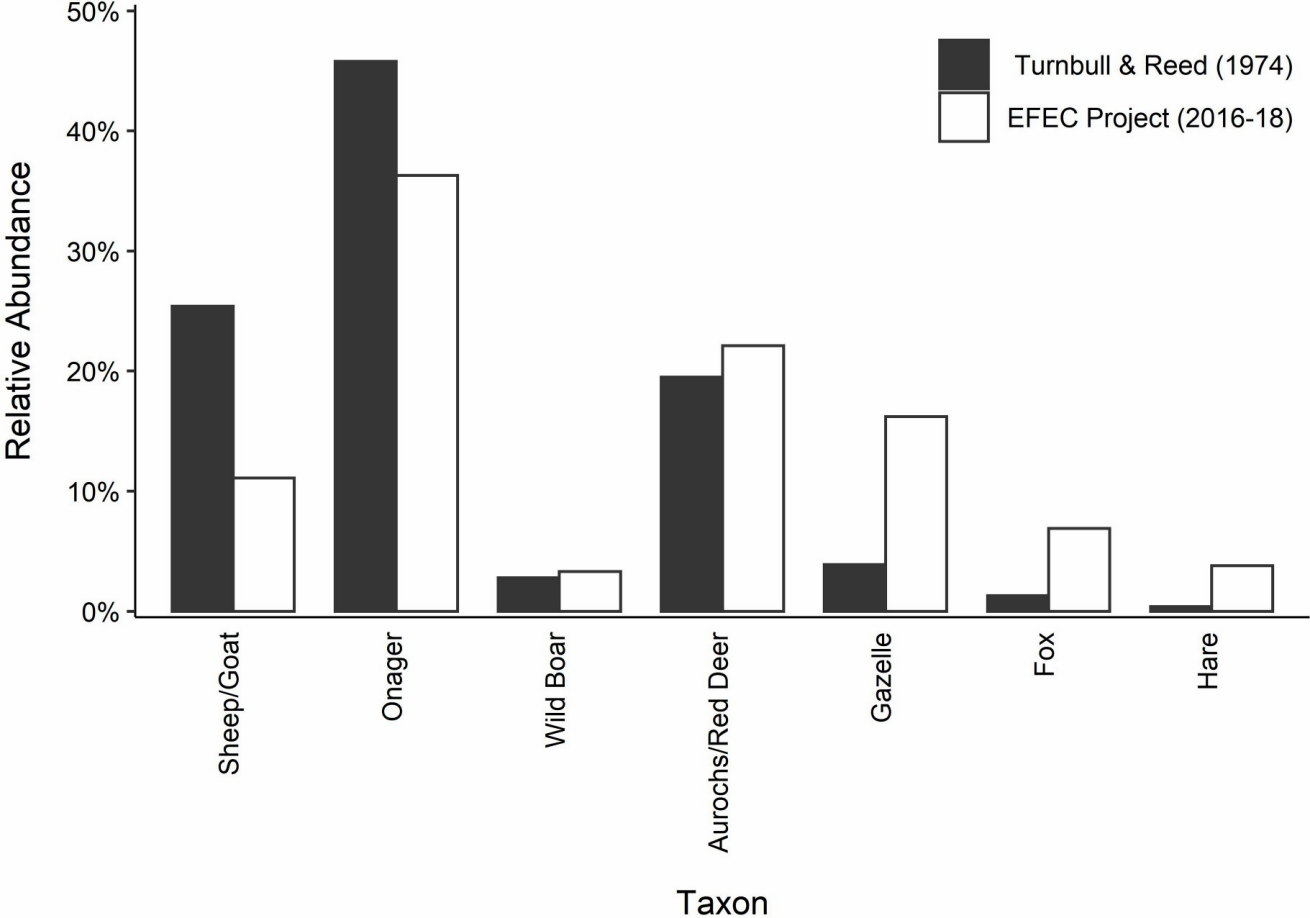
Comparison of the relative abundances of taxa recorded in the EFEC project assemblage (NISP = 452) and in the Iraq-Jarmo project assemblage studied by Turnbull and Reed (NISP = 2445). Relative taxon abundances were calculated from the diagnostic mammalian NISP counts presented in Fig 41 and those listed in Turnbull and Reed ([65]: Table 1 & pp.94-95) (including all mammalian taxa from hare size and above). Aurochs and red deer were combined into a single category. Only taxa present in both assemblages are shown in this graph (taxa re-ordered from Fig 46 to reflect taxon over- and under-representation in Turnbull and Reed’s study compared to the EFEC project study).

**Fig 48:**
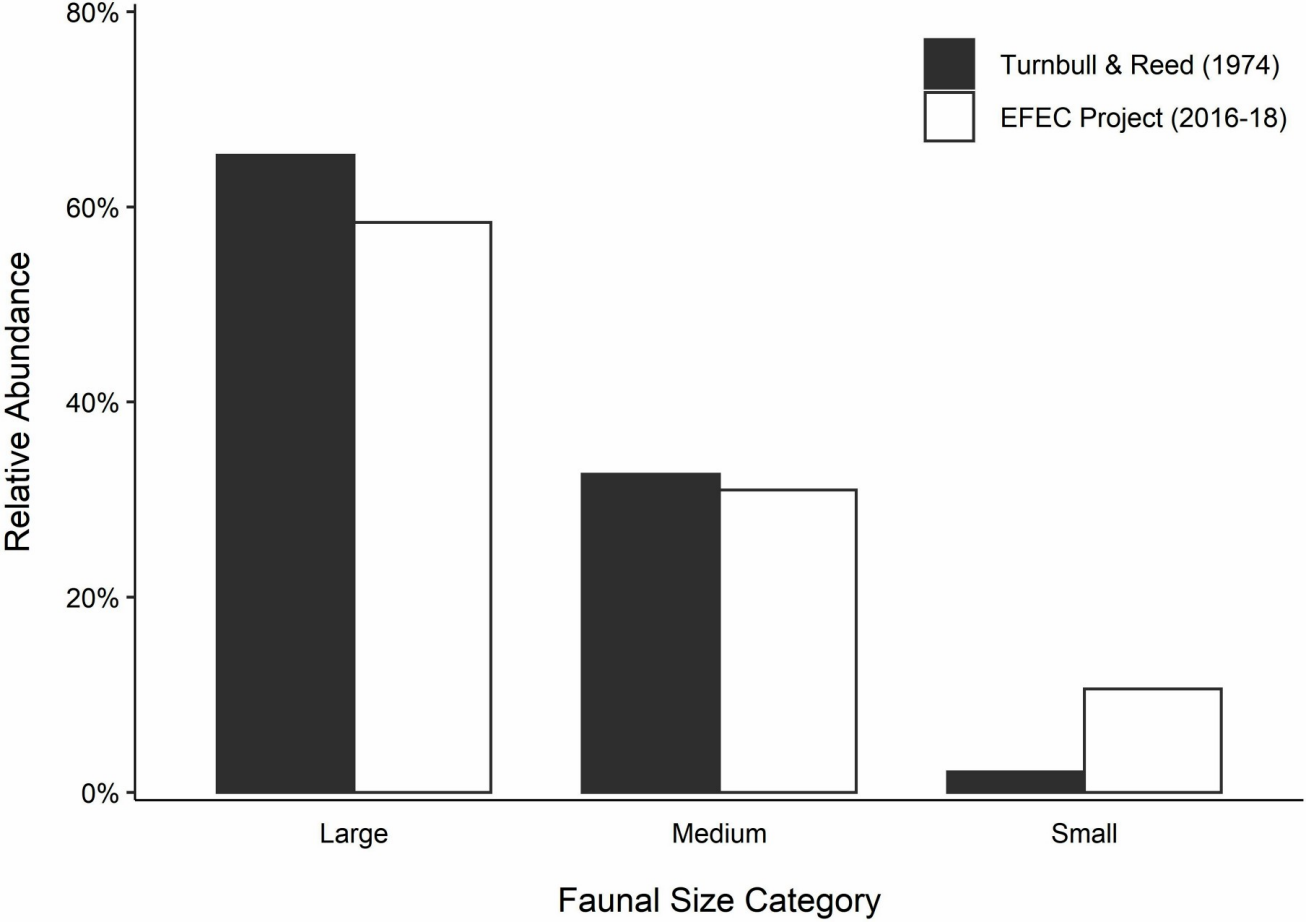
Comparison of the relative abundances of faunal size categories recorded in the EFEC project assemblage (NISP = 452) and in the Iraq-Jarmo project assemblage studied by Turnbull and Reed (NISP = 2445). ‘**Large**’: *Bos/Cervus/Equus***; ‘Medium’:** *Dama/Sus/Ovis/Capra/Gazella***; ‘Small’:** *Vulpes/Lepus***. Relative taxon abundances were calculated from the diagnostic mammalian NISP values presented in Fig 41 and those listed by Turnbull and Reed ([65]: Table 1 & pp.94-95)**.

The relative taxon abundances calculated from the Area A NISP counts in the present study (Fig 41) differ substantially from those published by Turnbull and Reed [65] (see also Fig 47). This is unsurprising given the differences in the recovery methods employed by the two excavations. It is well established that the recovery methods used in Howe’s excavations (collection by hand in the trench and dry sieving in the field with a large mesh) are highly likely to result in a recovery bias against smaller taxa. This bias is clearly reflected in the differences observed in the relative taxon abundances between the two assemblages (Fig 47). Larger taxa such as onager are more abundant in the Iraq-Jarmo project assemblage, while smaller taxa such as gazelle, fox and hare are more frequent in the EFEC project assemblage. When taxa are combined into size categories, the scale of the potential recovery bias becomes even clearer: the abundance of small taxa in the EFEC project assemblage is more than treble that of Turnbull and Reed’s study (Fig 48).

However, recovery bias cannot explain the higher proportions of sheep/goat recorded in the Iraq-Jarmo project assemblage (with an overall abundance of sheep/goat more than double that recorded by our analysis) and the far lower proportions of gazelle recorded by Turnbull and Reed. One possible explanation for the discrepancies observed in the representation of these taxa may be spatial variation in animal processing activities and/or bone waste disposal (i.e., that sheep/goat processing occurred more frequently in the cave chamber, while gazelle processing took place closer to the cave entrance). Another explanation might be identification bias. Without more information on the reference materials used for the identification of these taxa, and the morphological criteria applied by Turnbull and Reed, it is difficult to speculate on its likelihood and potential impact. The present study had access to gazelle reference specimens alongside sheep and goat; we are thus confident in the reliability of our identification of these taxa.

Another difference between the two assemblages is that Turnbull and Reed recorded several taxa which, to date, have not been identified in our Area A sample including wolf/dog (*Canis* sp.), jungle cat (*Felis* cf. *chaus*), lynx (*Felis lynx)* and badger (*Meles meles*). These taxa were very infrequent in the Iraq-Jarmo project assemblage (0.1–0.6%). Their absence from the EFEC project assemblage almost certainly reflects the higher number of diagnostic mammalian bones recorded by Turnbull and Reed (n = 2445, compared to n = 452 for the current study).

#### Comparisons with other Zarzian faunal assemblages

There are very few Zarzian sites with published animal bone assemblages. Those that have been reported are small and fragmentary (the highest in number, Ghar-e Khar, includes only 32 large mammalian diagnostics). The taxa found at Warwasi [101], TB75 [102], Ghar-e Khar [103] and Zarzi ([1]: p.23, [10]: pp.36-37) largely overlap with those recorded at PG and include aurochs, onager, deer, wild boar, sheep, goat, gazelle, fox, hare and tortoise. Similar to PG, the Warwasi diagnostics are dominated by onager. Equids are also present at TB75, but as a smaller proportion of the assemblage. By contrast, no onager was recorded by Hesse in his study of part of the Ghar-e Khar faunal assemblage. This probably reflects the division of the assemblage for analysis, with some of it having been removed by Dexter Perkins post-excavation. Hesse reports that preliminary work by Perkins had noted abundant quantities of equid bone ([103]: p.39). Unfortunately, to date, the assemblage studied by Perkins remains unpublished. No onager was identified at Zarzi, although this may reflect the very low number of diagnostic bones retrieved from this site. Given the paucity of the available evidence it is difficult to draw meaningful comparisons between PG and the above-mentioned sites other than noting that equid hunting might have been common among the Zagros Epipalaeolithic groups.

#### Worked bone and ochre use

**Fig 49:**
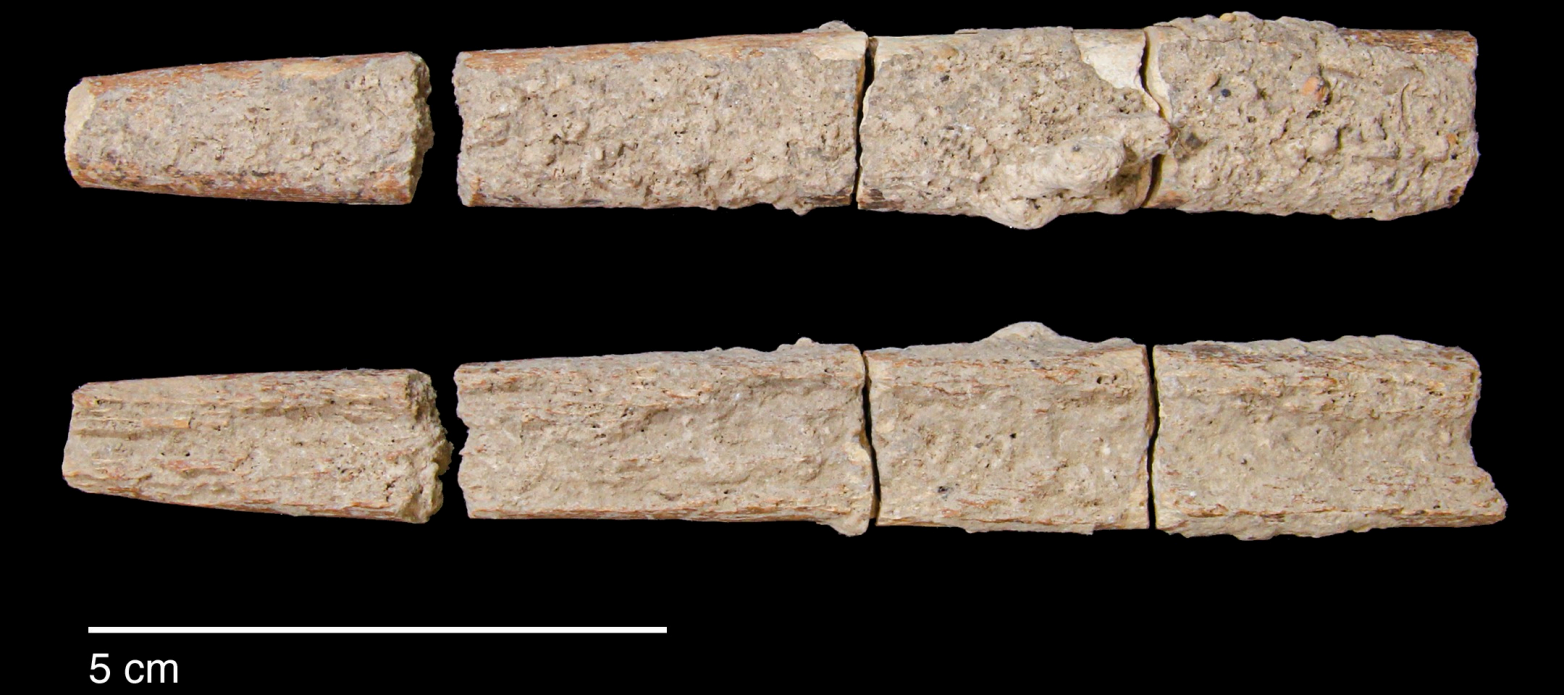
Bone point from context AAN (photo by K Swinson).

A single worked bone (bone point) was recovered from context AAN/Phase 1 (Fig 49), although another polished metapodial (found in context ABZ/Phase 2) might have functioned as a less formal tool. The bone point was crafted from a large (*Bos/Equus/Cervus* size) metapodial shaft that had been split longitudinally and ground to a taper (as far as can be observed given that both the ‘handle’ and the ‘tip’ ends were broken). The even tapered form is characteristic of the Zagros prehistoric bone tools and seems to have been achieved by grinding and turning bone against stone, unlike Levantine Natufian bone points which tended to be sharpened with chipped stone [104, 105]. Bone points could have been used for many tasks including leather-working, piercing and basketry [106].

**Fig 50:**
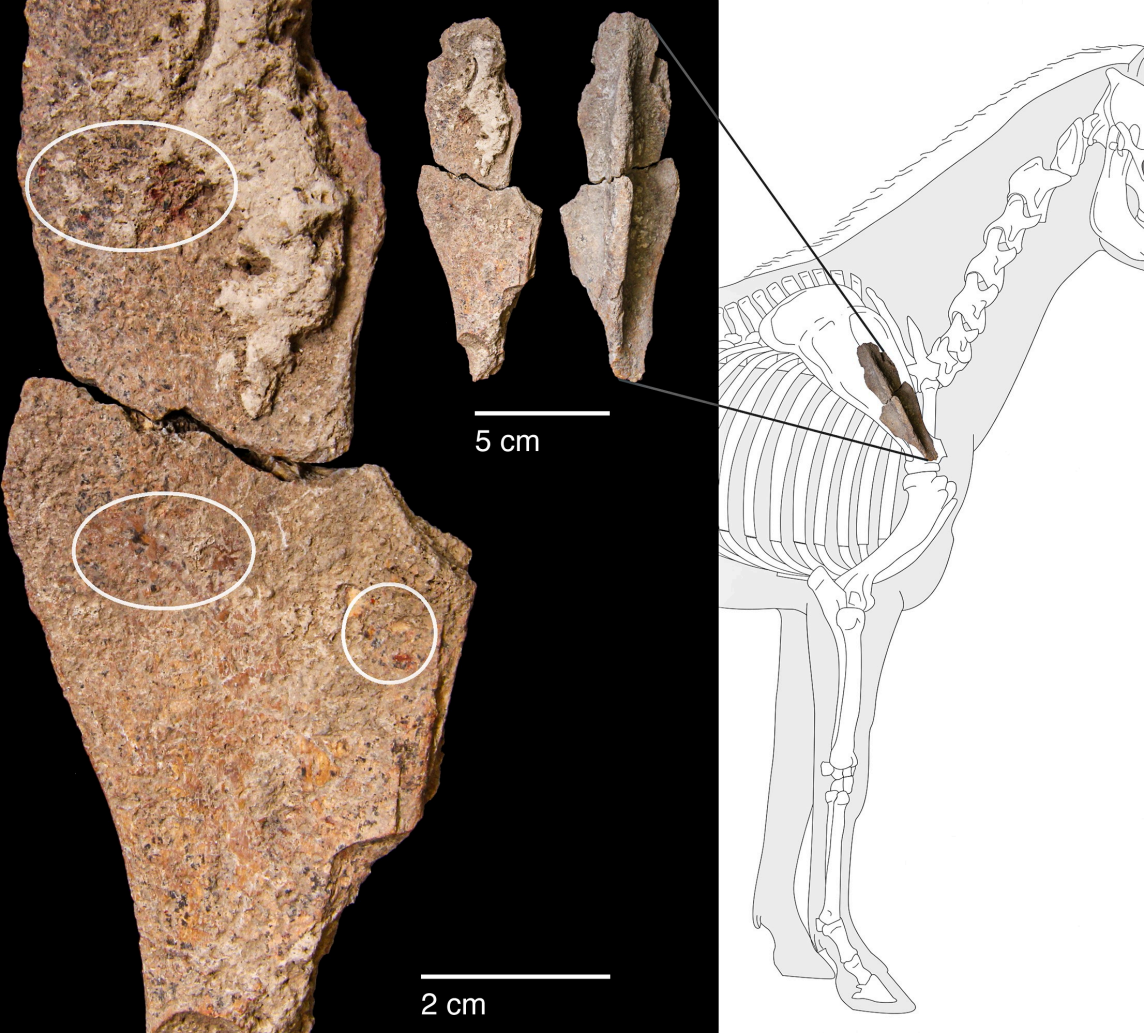
Onager scapula from context AAE with red ochre traces (photo by K Swinson). Onager drawing adapted from [80] (p.21).

Red ochre was identified on the surface of 4 bone fragments: 1 onager bone and 3 tortoise carapace fragments. The onager fragment (context AAE/Phase 3) is a neck and part of the blade of a scapula (Fig 50). The neck shows signs of impact breakage, possibly the result of the removal of the glenoid cavity and articular end. The ventral (flat) side of the scapula blade is coated with deposits of ochre, suggesting it could have been used as a palette. The symbolic and functional use of large mammal scapulae has been noted from the Eastern Mediterranean Upper Palaeolithic throughout prehistory [107] and includes their use as palettes in the Neolithic (e.g., in Anatolia [106] and Armenia [108]: p.200). In the Zagros, a notched and polished goat scapula has been reported from Zarzian layers at Ghar-e Khar [103] indicating a long-lived tradition of scapula working in this region.

**Fig 51:**
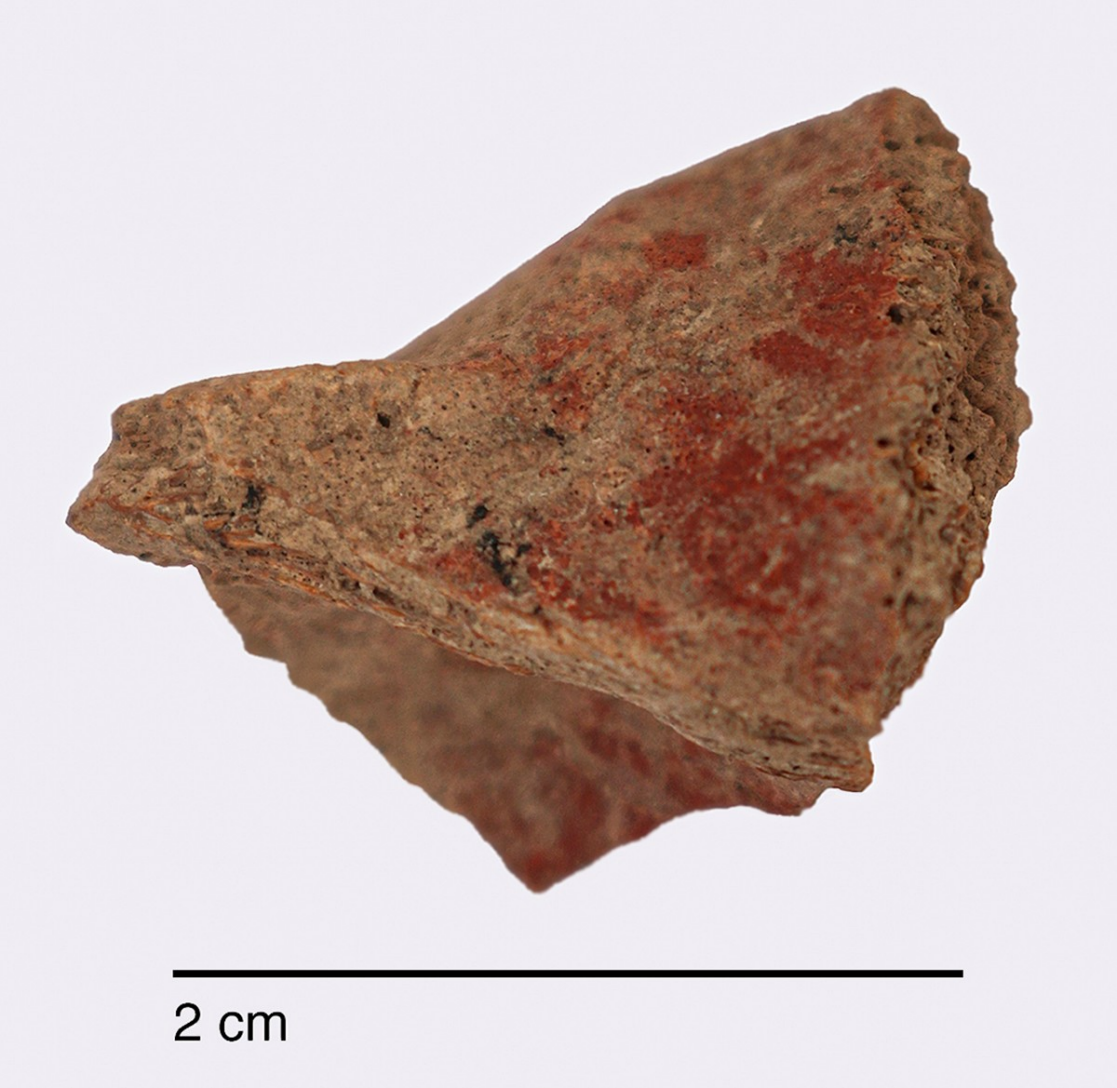
Tortoise carapace (marginal scute) with ochre staining from context AAI (photo by D Baird).

One of the 3 ochre-stained tortoise fragments is a marginal scute of the carapace (from AAI/Phase 3) with red ochre and charcoal fragments clearly visible on its surface (Fig 51). The ochre is located on the inside lip of the domed carapace and presumably was directly in contact with the bone. The association of ochre with the tortoise fragment suggests that the tortoise shell might have served as a container for ochre, or as a palette for its mixing. 2 more tortoise scutes (from AAI/Phase 3 and ACR/Phase 1) have linear traces of ochre on their surfaces hinting at ochre decoration of a possible vessel. Surface polish observed on 2 other tortoise scute fragments (from AAP/Phase 1) supports the interpretation of the tortoise carapaces being used as bowls. Scraping marks identified on a single tortoise scute (from AAO/Phase 1) may reflect vessel use but might also have resulted from tortoise meat consumption.

Ochre use is well known from the Middle Palaeolithic onwards and is particularly widespread in the Upper Palaeolithic and Epipalaeolithic of Europe and SW Asia. Occurring in pieces by itself and on objects such as shell and ground stone, the pigment is also found in association with burials (for SW Asian examples see [109–111]). Speth and Tchernov [112] have suggested that tortoise carapaces were used as vessels from as early as the Middle Palaeolithic, hence their use as containers at PG, where tortoises were heavily exploited, would not be unexpected.

##### Archaeobotany

The perception of the absence of plant food use in the Zagros Epipalaeolithic is widespread in the literature. In large part this stems from the absence of the traditional indicators of plant harvesting and processing familiar from Levantine sites of this period. Some authors have noted, for example, the lack of evidence for the use of ground stone for seed processing as opposed to pigment preparation ([113]: p.52). Others have deduced, based on the limited pollen evidence available, that climate conditions were too cold and dry to permit sufficient grass growth [114]. Another issue is the apparent poor preservation of charred plant remains at the few sites in which archaeobotanical recovery has been attempted via manual (bucket) flotation [115]. Limited archaeobotanical preservation also explains the lack of charcoal finds suitable for radiocarbon dating [31, 116]. However, it remains unclear whether it genuinely reflects a low contribution of plants to Zarzian subsistence or it has been disproportionally affected by the lack of intensive archaeobotanical sampling at the few excavated sites. Addressing this issue has been a major objective of the EFEC project excavations at PG.

Previous archaeobotanical sampling at Iraqi Zarzian sites is limited to Zarzi and PG. While re-excavating the Zarzi terrace in 1971 Wahida dry sieved (2mm mesh) and wet sieved (1mm mesh) select deposits. Although the number and volume of processed sediment samples are unknown, they all derived from 2 excavation squares: 3B and 3C. The sole plant macrofossils reported by Wahida include 2 uncarbonized buckthorn seeds (identified as *Rhamnus cathartica*) from square 3C ([10]: p.36] which, by virtue of their preservation status, almost certainly represent historical/modern intrusions. The charred plant remains reported by the Iraq-Jarmo project excavations at PG are limited to 14 wood charcoal identifications: 11 fragments of oak, 1 of a “large” tamarisk tree, 1 of poplar and 1 of an unspecified conifer ([2]: p.59). Although Braidwood and Howe alluded to the recovery of non-wood plant remains as well (to be studied by Hans Helbaeck) no further report was forthcoming.

In this paper we present the results of the analysis of archaeobotanical flotation samples (including both wood charcoals and non-wood charred plant macrofossils) retrieved from the Area A late Pleistocene sequence (Phases 1–3) alongside the first results of the analysis of 10 phytolith samples from select Area A contexts. Laboratory work is ongoing on the samples retrieved from Area B and the Area A post-Pleistocene Phase 4. In the following sections we evaluate the taxonomic composition of the archaeobotanical assemblage, assess its preservation and formation processes, and interpret these data in the light of Zarzian plant exploitation and the vegetation resources available in the PG environs. We also discuss the insights they provide concerning the impact of climate change on the local and regional vegetation, and the potential they reveal for future archaeobotanical research at pre-agricultural sites in the Zagros region and beyond.

#### Methods

52 flotation samples have been studied from Phases 1–3, including all contexts that were sampled by machine-assisted flotation, corresponding to 2130.95 litres of excavated sediment. Laboratory analyses of charred plant macrofossils were undertaken at the University of Liverpool Archaeobotany laboratory. Each flot fraction was passed through a stack of 100mm diameter geological test sieves (meshes 4mm, 2mm, 1mm, 500μm and 250μm, plus retainer). The resulting fractions were sorted in their entirety under a Leica S8APO stereozoom microscope (magnifications x10-x80). Non-wood charred macrofossils were identified by comparison to modern specimens held in the Liverpool botanical reference collection and published reference works [117–119]. The reference collection of the UCL Institute of Archaeology was also consulted. Wood charcoals were hand- and/or pressure-fractured with a carbon steel razor blade to produce fresh sections of the three anatomical planes (TS = Transverse Section, RLS = Radial Longitudinal Section and TLS = Tangential Longitudinal Section). These were examined under a Meiji MT7500 darkfield/brightfield metallurgical microscope (magnifications x50, x100, x200, x400). Botanical identifications were made by comparison to published reference works [120, 121] and specimens held in the Liverpool wood charcoal reference collection. Select wood charcoal specimens were photographed for publication with a Keyence VX7100 4K ultra-high accuracy digital microscope at magnifications x100-x1000. SEM microphotography, requiring the gold coating of fragile charred specimens mounted on steel stubs with adhesive conductive carbon tape, was not undertaken due to the low numbers of complete non-wood specimens and the need to preserve both wood charcoals and non-wood charred macrofossils for future radiocarbon assays and other analyses. Phytolith analysis took place at the Department of Archaeology and Anthropology, Bournemouth University (a detailed description of the methods is provided in S3 File and S15 Table).

#### Botanical assemblage composition

**Fig 52:**
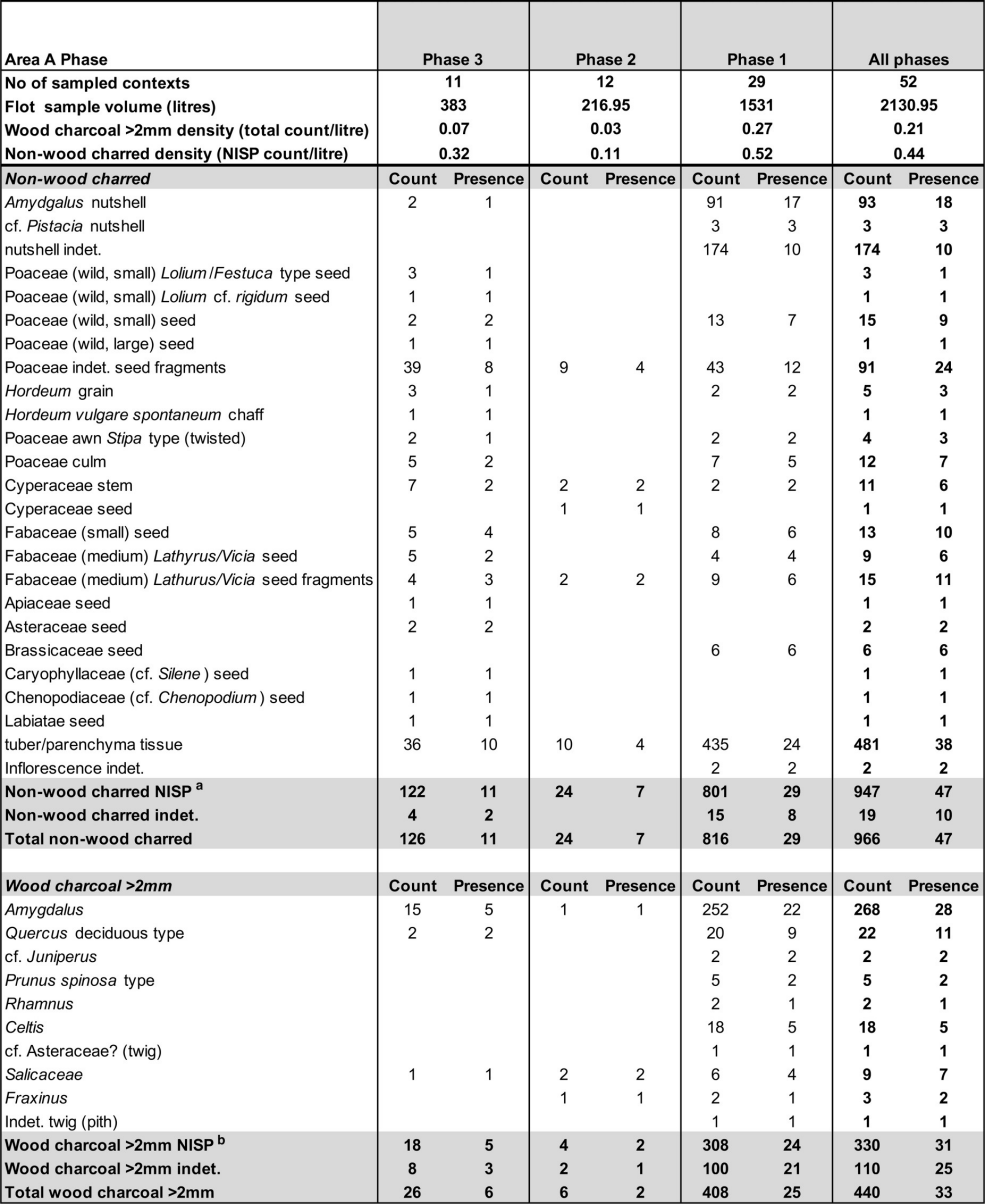
Per phase and summary densities, counts and ubiquity (sample presence) of all types of charred plant macro-remains retrieved from the Area A late Pleistocene sequence (Phases 1–3). ^a^The Non-wood charred NISP count includes all non-wood charred plant macrofossils that could be attributed to a specific taxonomic group (family, genus, species) or to a clearly defined generic plant part group (e.g., nutshell or tuber/parenchyma tissue). The latter attribution is based on the fact that, although from a strictly botanical point of view such groups do not provide insights into plant taxon selection and use, from an archaeological and palaeoecological one they represent useful indicators of the spectrum of plant resource choice and habitat diversity (e.g., in the case of PG relating to the collection of nuts and tubers, and the contribution of dryland and wetland habitats to late Pleistocene plant exploitation) (see also S16A and S16B Table); ^b^The Wood charcoal >2mm NISP count includes all botanically identified wood charcoal fragments.

Fig 52 and S16A and S16B Table present the results of the microscopic analysis of the non-wood plant macrofossils and the wood charcoals retrieved from the Area A late Pleistocene sequence. In total, 966 non-wood items and 440 wood charcoals were retrieved from 52 contexts sampled by machine-assisted flotation. Of these, 947 non-wood items and 330 wood charcoals were initially identified to species, genus or family level, with the exception of tubers/parenchyma tissues for which further SEM analysis is planned, and a proportion of nutshell fragments that were too small and/or eroded to be positively attributed to a particular taxon. The indeterminate non-wood items also contained some fragments of amorphous burnt plant tissues that may represent carbonised nutmeat or other types of plant food debris, again to be determined by SEM. At the time of writing, due to the disruption caused to non-clinical laboratory research by the COVID-19 lockdown, the analysis of these remains was still incomplete.

As Fig 52 and S16A Table demonstrate, the density and distribution of all types of charred plant macro-remains varies greatly by phase and context. While density values are overall low across the sampled sequence, the highest densities of charred plant macro-remains and the highest diversity of wood charcoal taxa occur in the stratigraphically and chronologically earliest Phase 1 deposits. Phase 2 samples display the complete opposite pattern, while Phase 3 contains a slightly higher diversity of non-wood taxa. These differences in charred plant macro-remain densities and taxonomic composition are best explained by variations in preservation conditions and (in the case of Phase 2 contexts) likely occupation intensity as well, rather than diachronic trends in the availability, distribution and use of the local vegetation resources (see below, **Assemblage formation processes** section).

**Fig 53:**
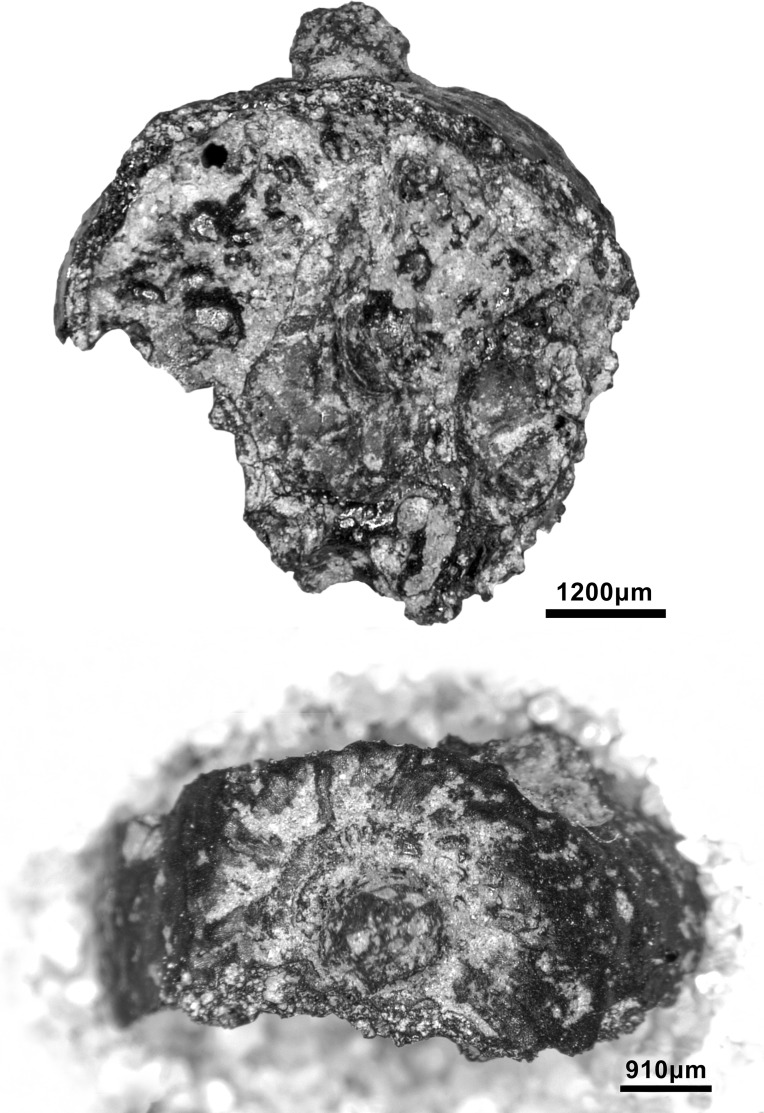
Tuber fragment from context AAJ (photos by C Kabukcu).

**Fig 54:**
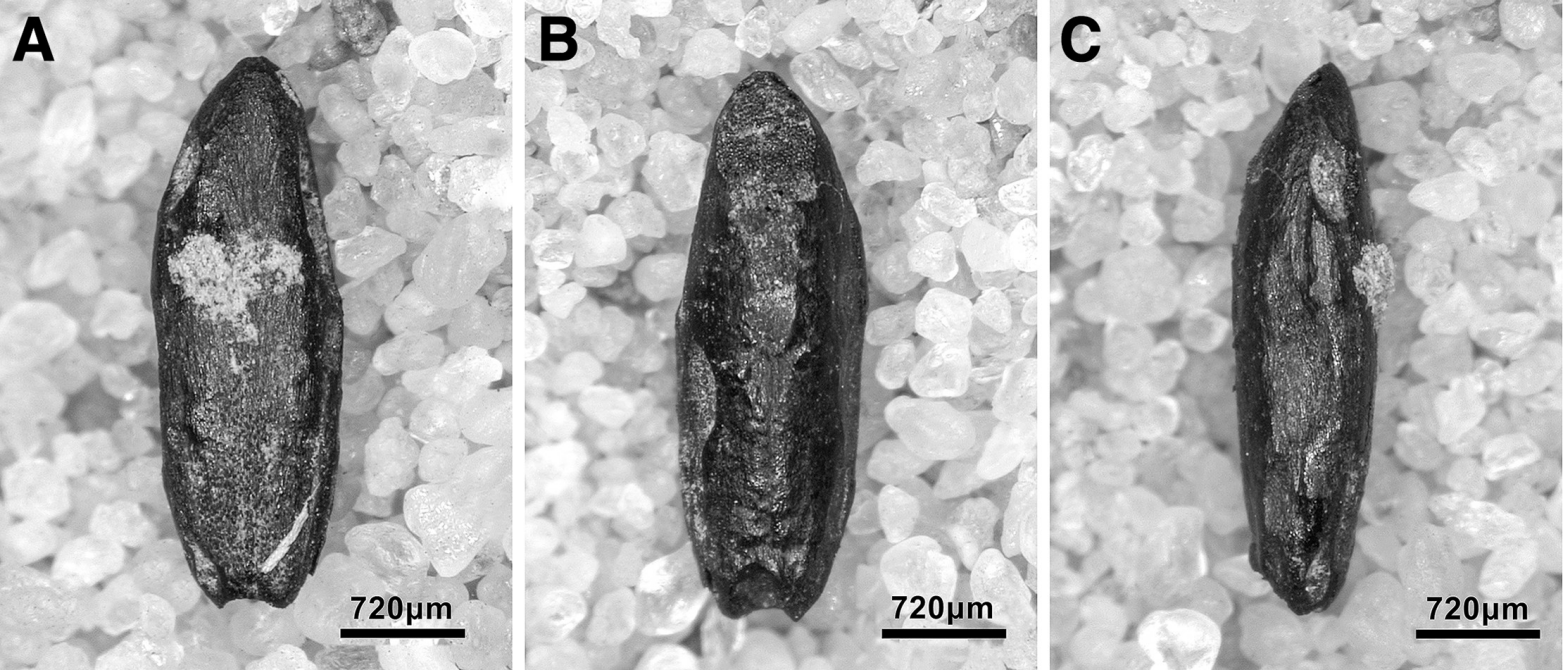
Different views of small Poaceae seed from context AAT (photos by C Kabukcu). (A) ventral; (B) dorsal; (C) lateral.

**Fig 55:**
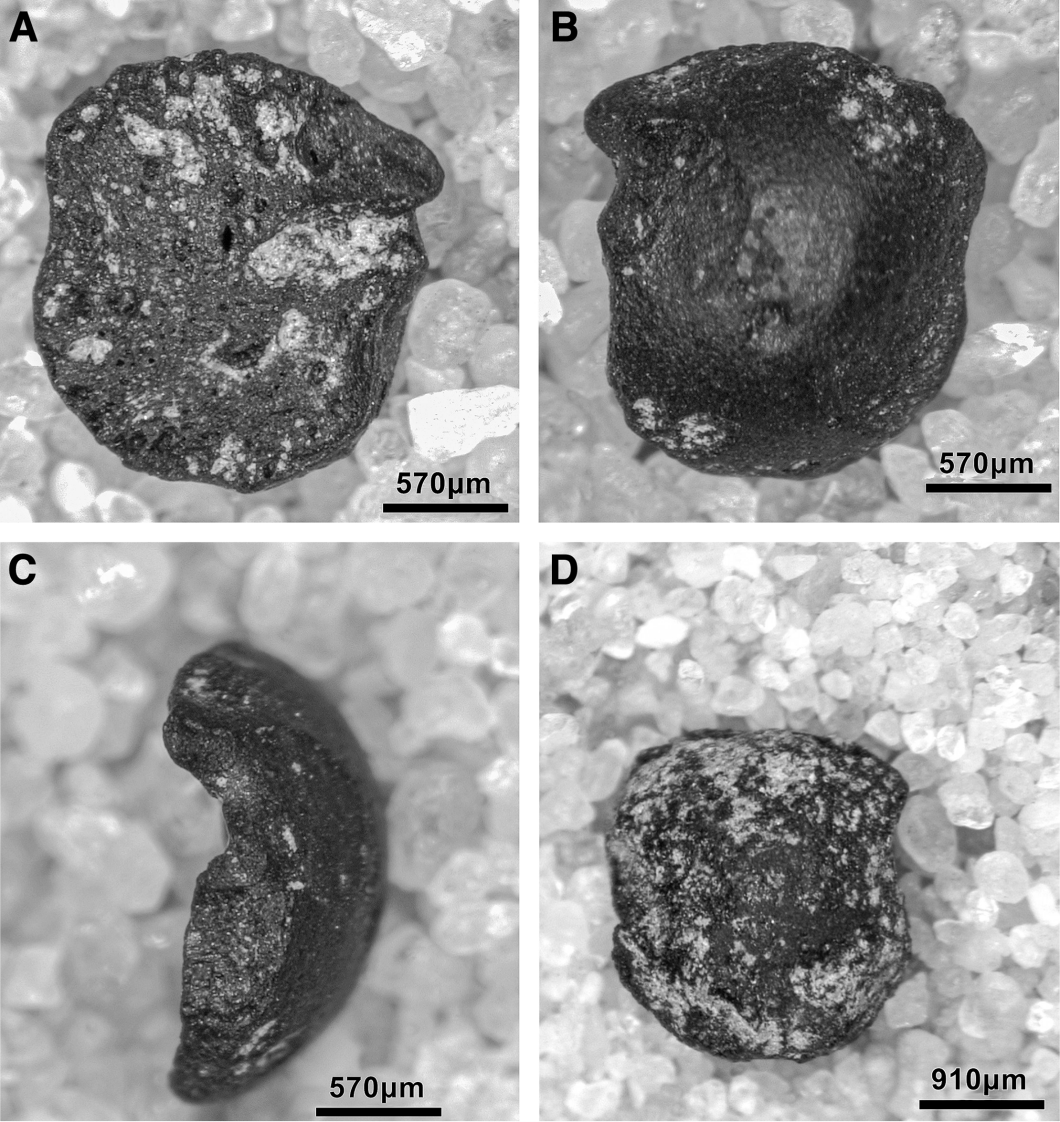
*Vicia/Lathyrus* legume seeds (photos by C Kabukcu). (A-C) seed from context ADC; (D) seed from context ACX.

Across all phases the most common non-wood remains by count and ubiquity (sample presence) are tubers/parenchyma, followed by grasses (small-seeded Poaceae and Poaceae seed fragments, which may represent both large- and small-seeded taxa) and medium-sized legumes (Figs 53–55). The latter comprise exclusively *Lathyrus*/*Vicia* preserved as whole seeds and seed fragments (Fig 55). Cyperaceae stem fragments also occur in samples from all phases. They often appear triangular in section, which indicates that they may have derived from *Cyperus* sp./spp. (sedges) abounding in wetland habitats.

**Fig 56:**
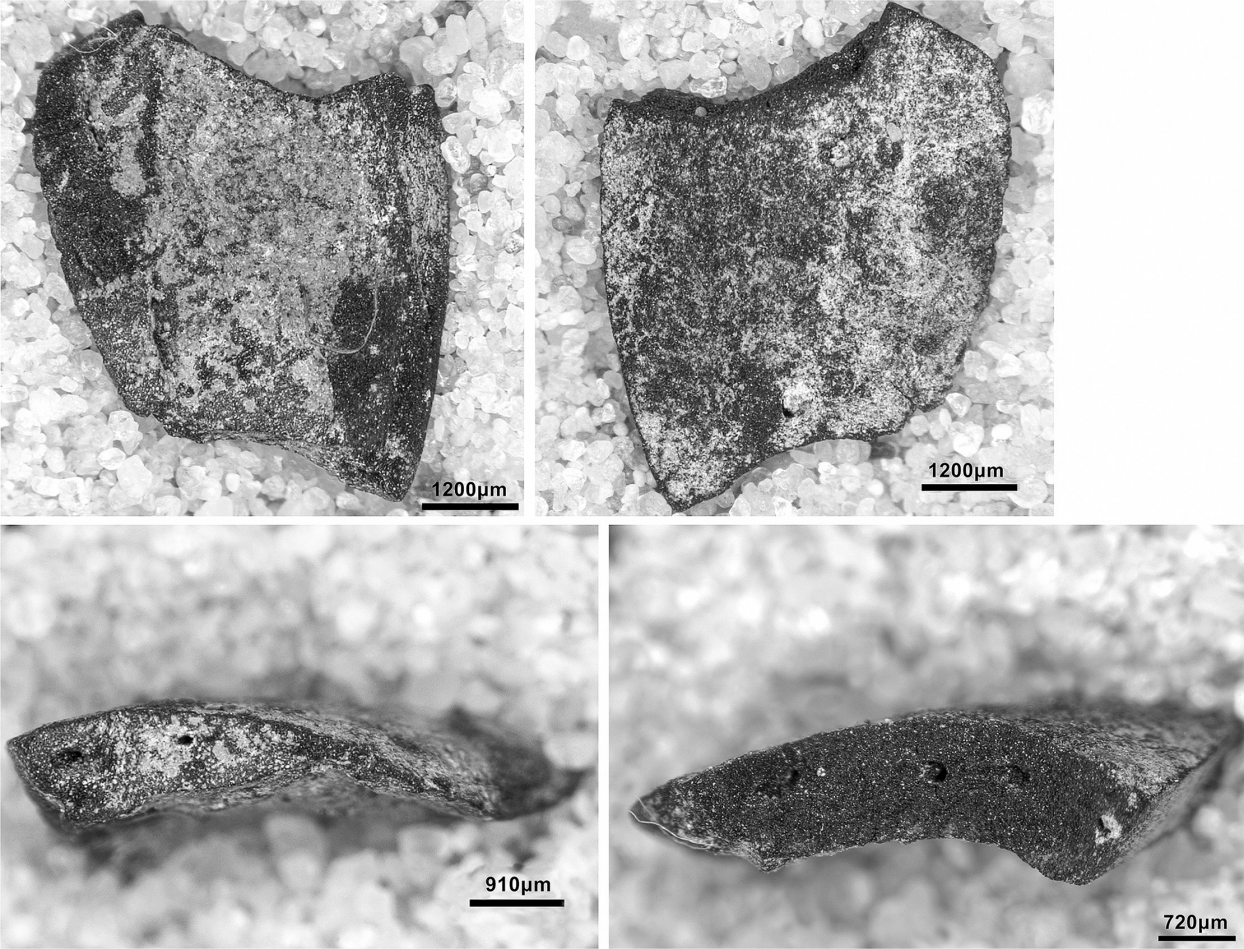
*Amygdalus* nutshell fragment from context ACN (photos by C Kabukcu).

**Fig 57:**
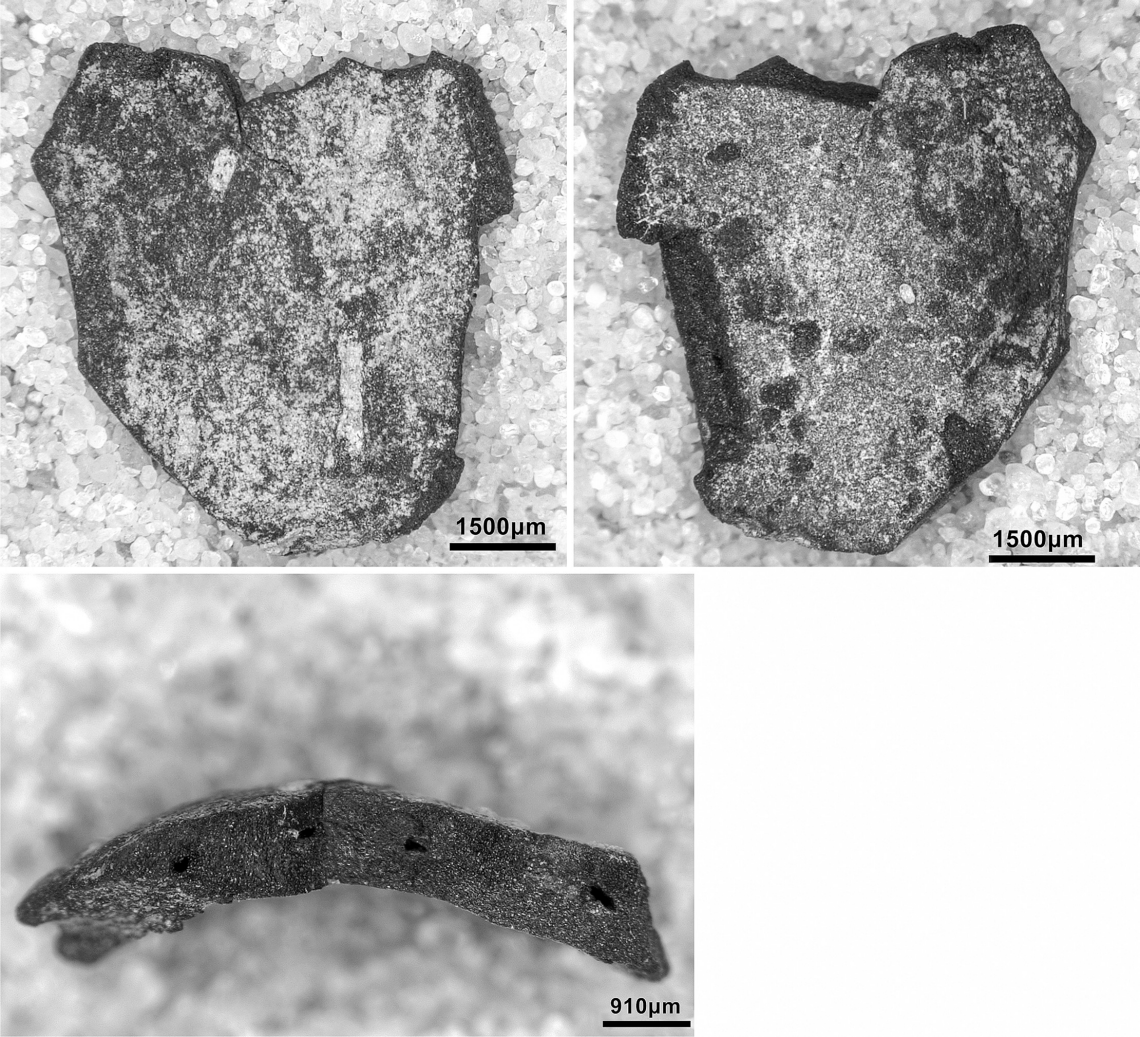
*Amygdalus* nutshell fragment from context ACN (photos by C Kabukcu).

The comparatively better-preserved non-wood assemblages of Phases 1 and 3 provide a more detailed picture of the diversity of plant exploitation at PG. Prominent in Phase 1 by count and ubiquity is nutshell, definitely of *Amygdalus* (wild almond) (Figs 56 and 57) and likely of *Pistacia* (terebinth) as well, although the identification of the latter remains tentative due to the very fragmentary nature of the remains. The high numbers and ubiquity of nutshell fragments in Phase 1 samples point to the regular contribution of gathered nuts to late Palaeolithic subsistence.

**Fig 58:**
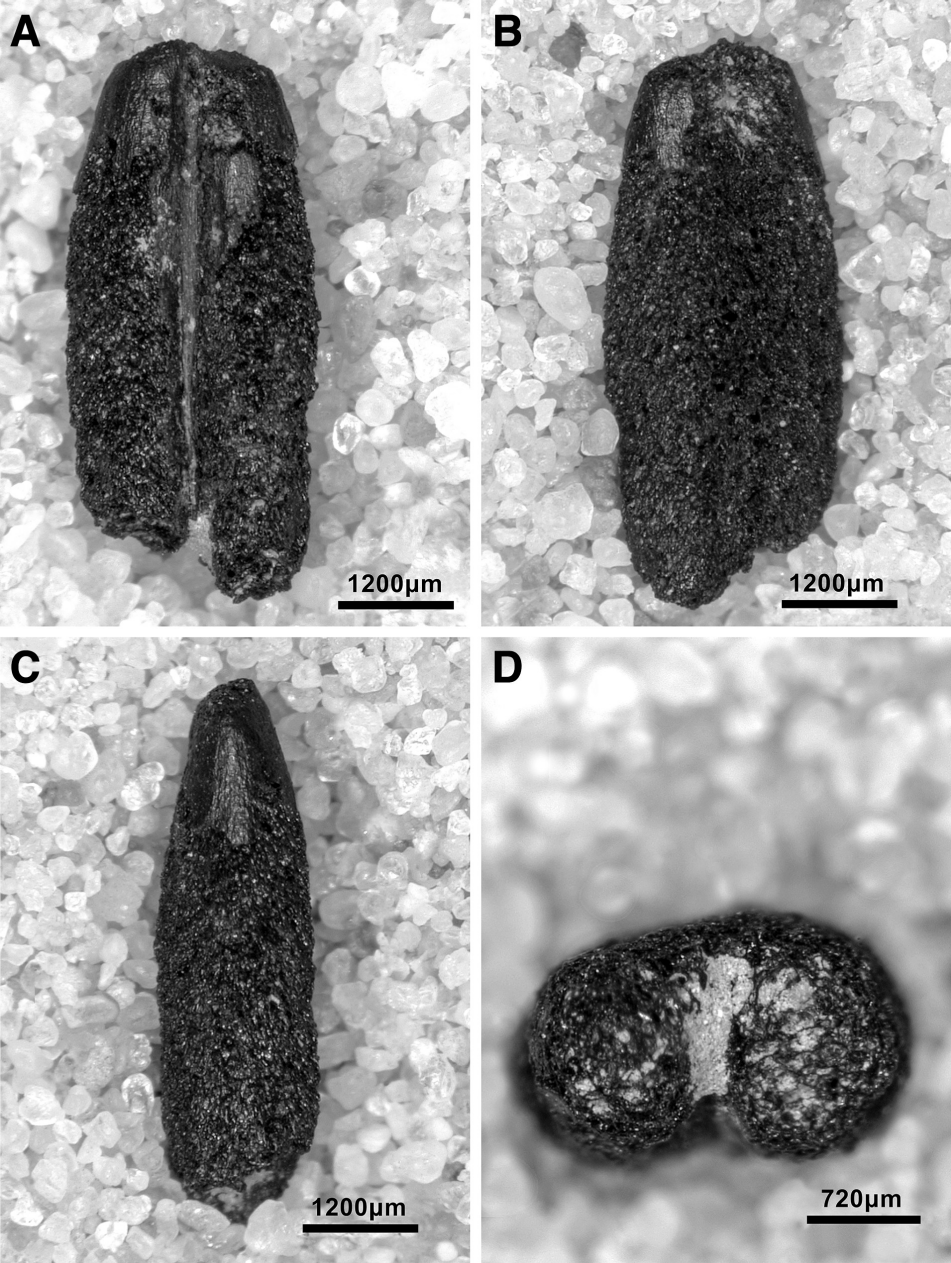
Different views of *Hordeum* grain from context ADG (photos by C Kabukcu). (A) ventral; (B) dorsal; (C) lateral; (D) cross section.

**Fig 59:**
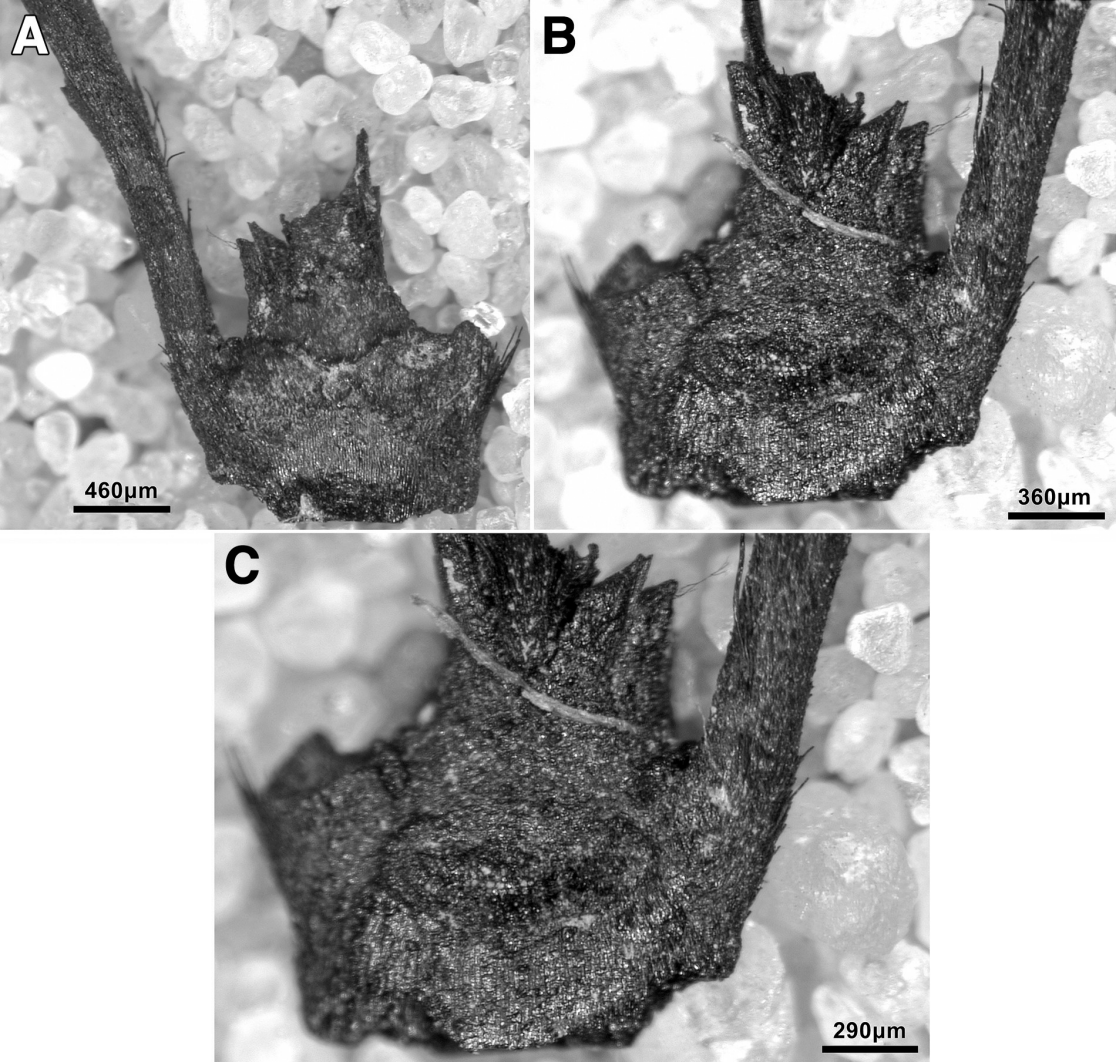
*Hordeum vulgare spontaneum* chaff from context ABC (photos by C Kabukcu). (A) Back view; (B-C) Front view showing the wild-type break-off scar.

**Fig 60:**
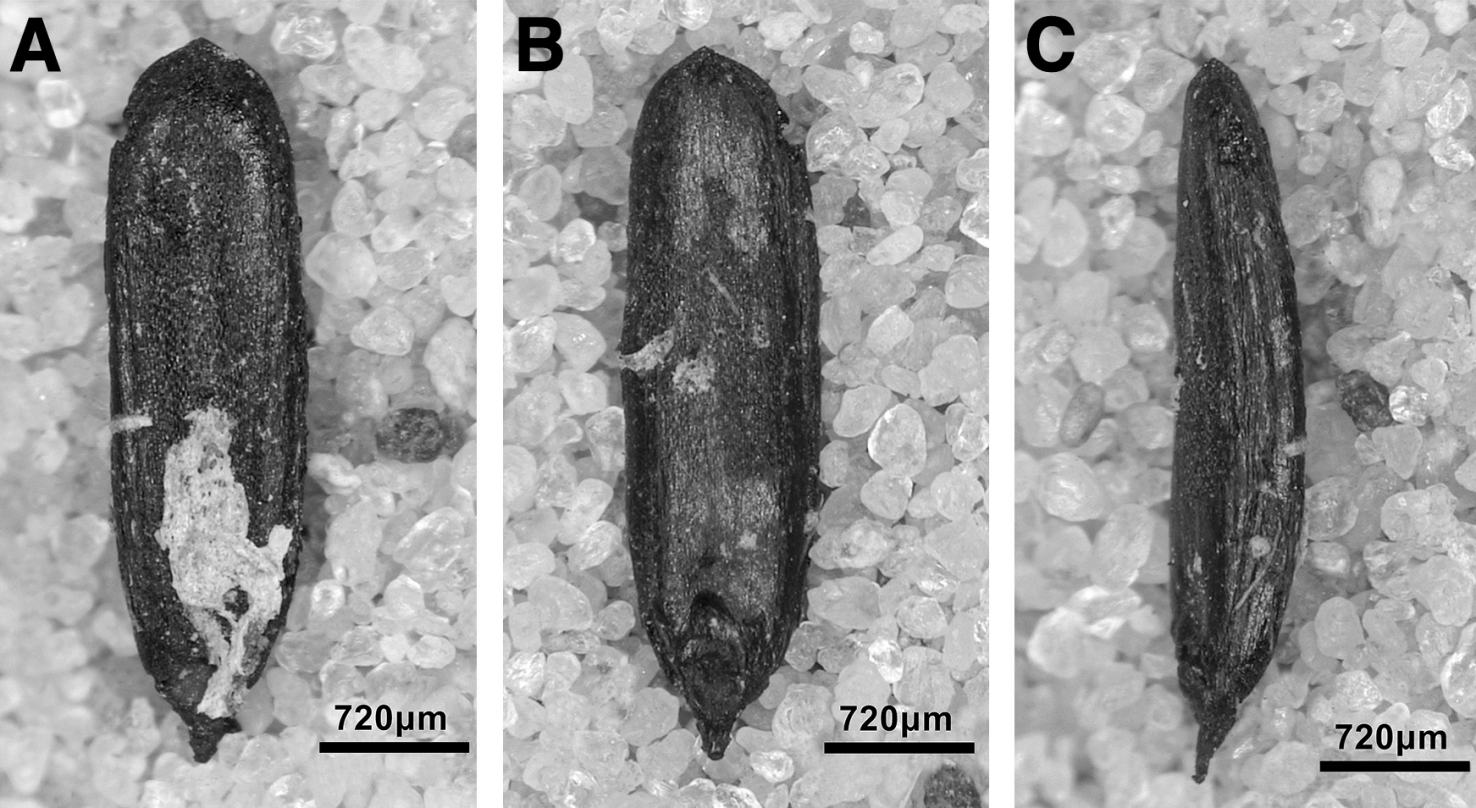
Different views of *Lolium* cf. *rigidum* seed from context ABE (photos by C Kabukcu). (A) ventral; (B) dorsal; (C) lateral.

**Fig 61:**
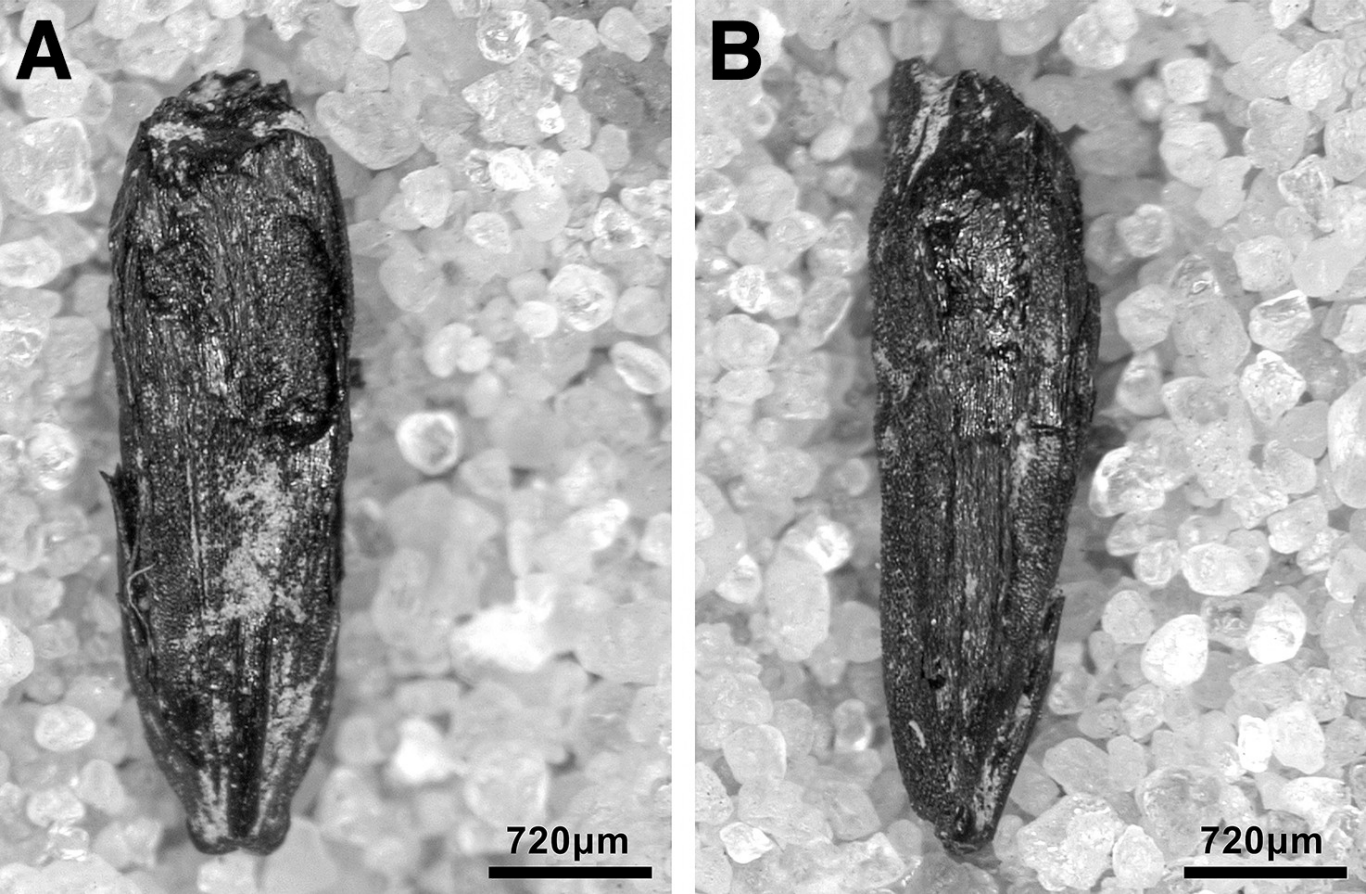
Different views of *Lolium*/*Festuca* seed from context ABC (photos by C Kabukcu). (A) ventral; (B) lateral.

Small- and large-seeded grasses (Poaceae) are also present in both Phase 1 and Phase 3 samples. Although their poor preservation prevents more precise identifications, snapshots of the spectrum of the grass taxa exploited occur sporadically in the assemblage. These include fragments of the characteristically twisted awns of feather-grass (*Stipa*) and the few *Hordeum* sp. grains retrieved from contexts AAF, ABC (Phase 3) and ACH, AAO, AAU, ADG (Phase 1) (Fig 58). The presence of *Hordeum vulgare spontaneum* chaff in ABC further indicates that wild barley was probably among the grasses gathered by the late Palaeolithic inhabitants of PG (Fig 59). Of the small-seeded Poaceae, we have tentatively identified ryegrass (*Lolium* cf. *rigidum*) and *Lolium*/*Festuca* in samples from ABE and ABC (Phase 3) (Figs 60 and 61). Small-seeded legumes (Fabaceae), impossible to identify beyond family level, are also relatively well represented in both Phase 1 and Phase 3 samples. By contrast some taxa are present only in single phases. Brassicaceae (mustard family) seeds occur exclusively in Phase 1 samples. Their presence in 6 different contexts (ACH, ACL, AAR, ACU, ADA, ADC) spread across the Phase 1 sequence suggests that their use might have been more widespread than their modest counts indicate. Very few seeds of Labiatae, Asteraceae, Chenopodiaceae (cf. *Chenopodium*), Apiaceae, and Caryophyllaceae (cf. *Silene*) were also occasionally present in Phase 3 samples. These, if not intrusive from post-Pleistocene layers, might represent background ‘noise’ as they are all identified with open ground and/or ruderal plant communities.

**Fig 62:**
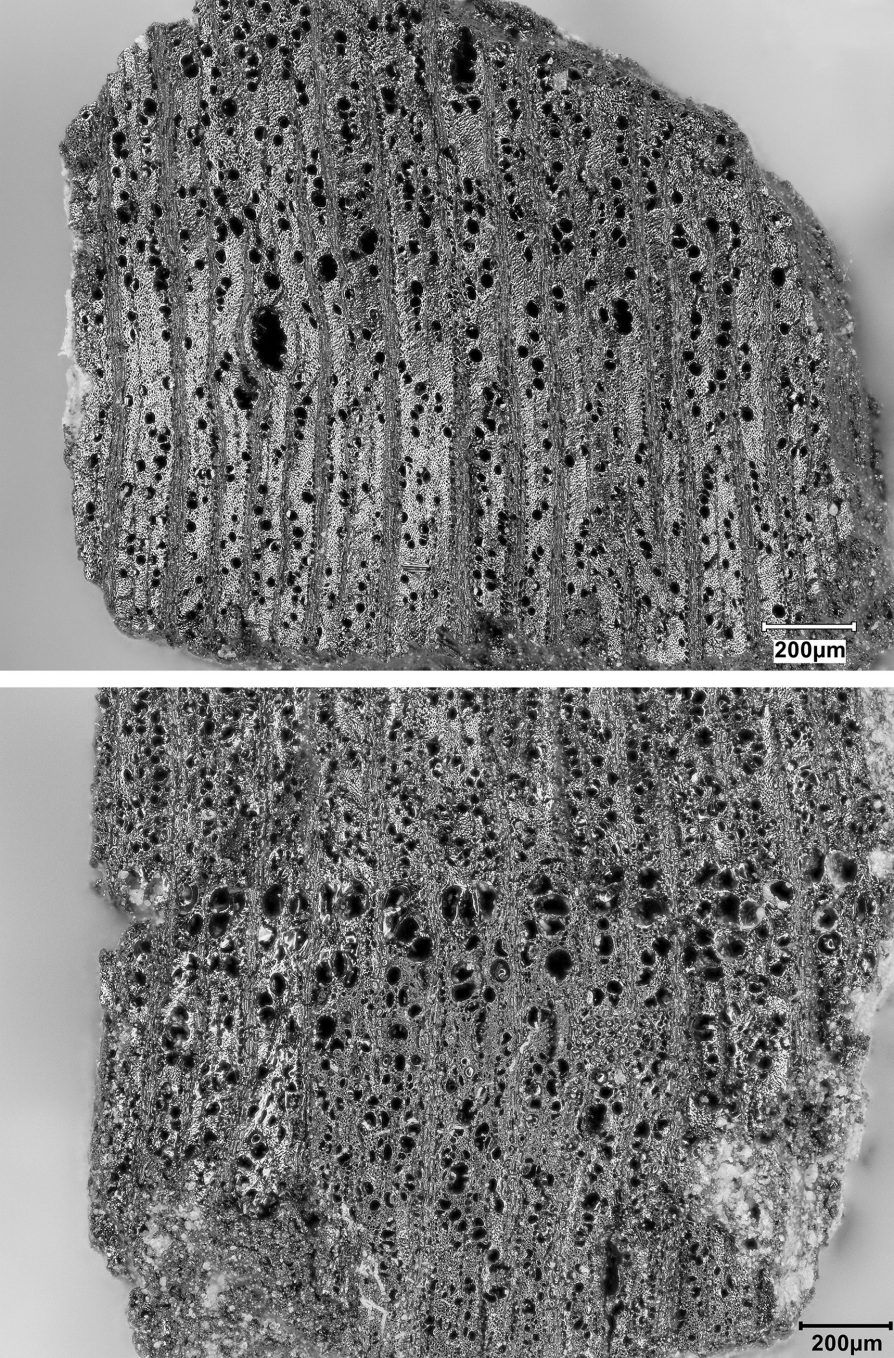
TS planes of *Amygdalus* charcoal fragments from context ADG (photos by E Asouti).

**Fig 63:**
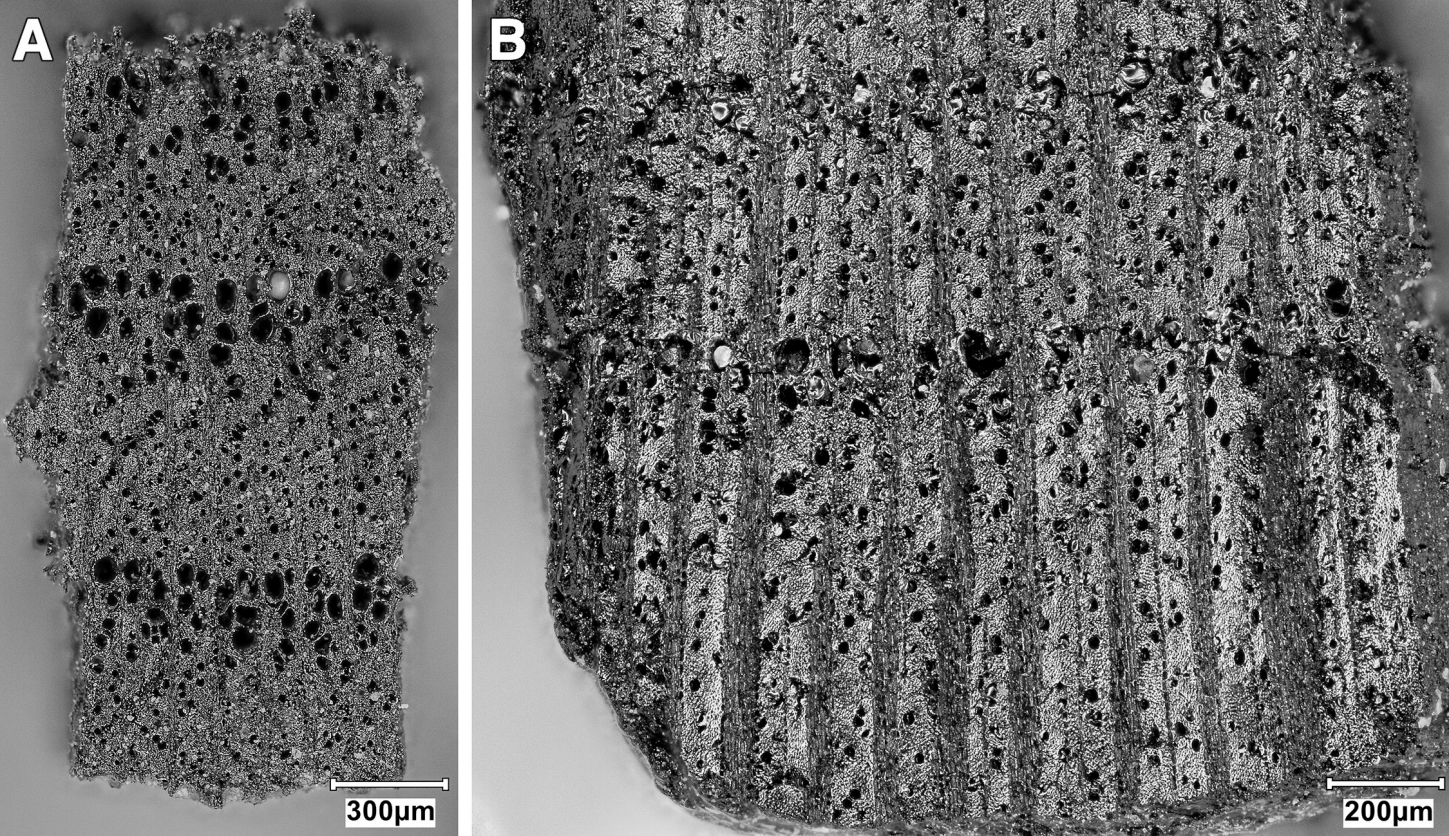
TS planes of *Amygdalus* charcoal fragments (photos by E Asouti). (A) context ACT; (B) context ACX.

**Fig 64:**
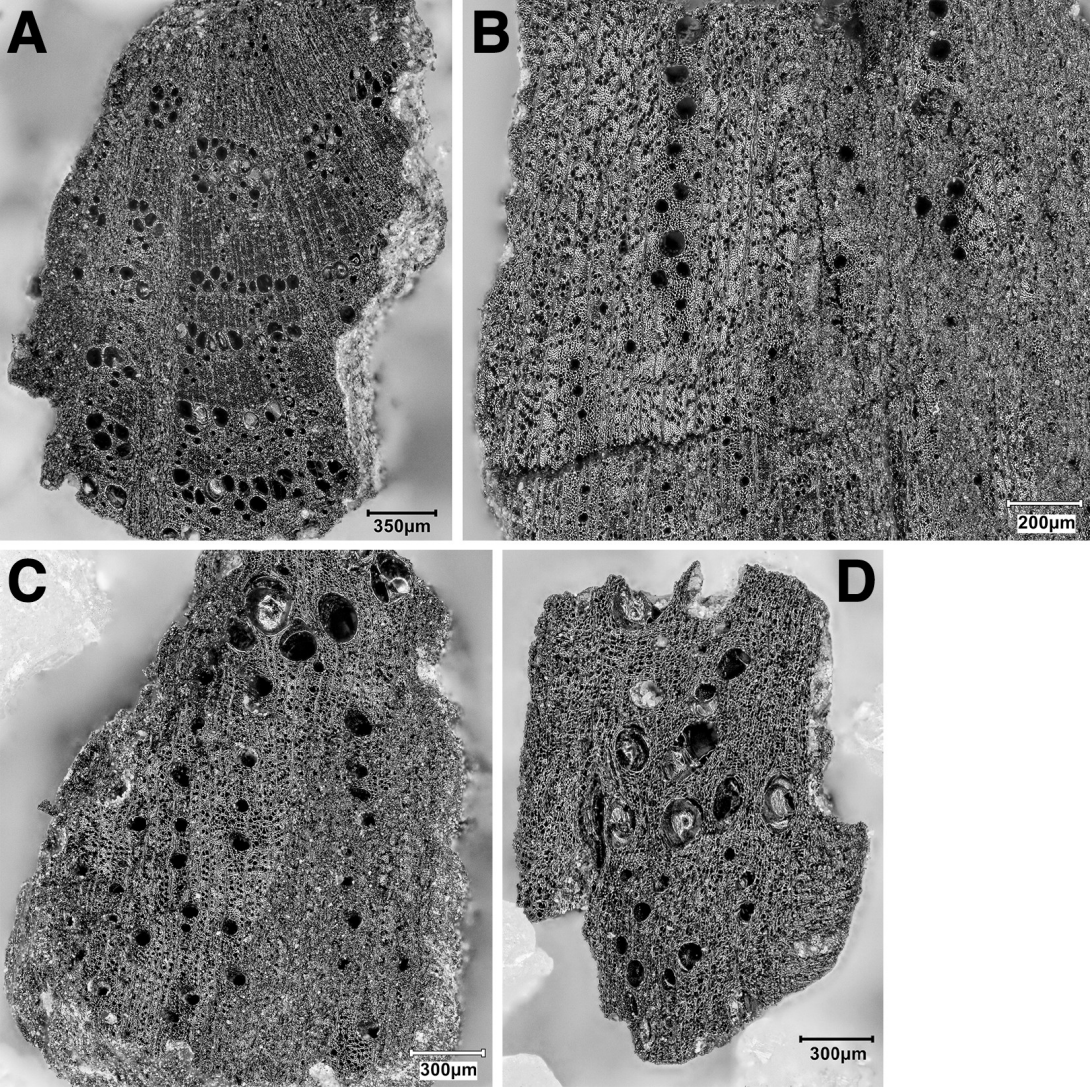
TS planes of deciduous *Quercus* charcoal fragments (photos by E Asouti). (A) context AAF; (B) context AAH; (C) context ACT; (D) context ADF.

**Fig 65:**
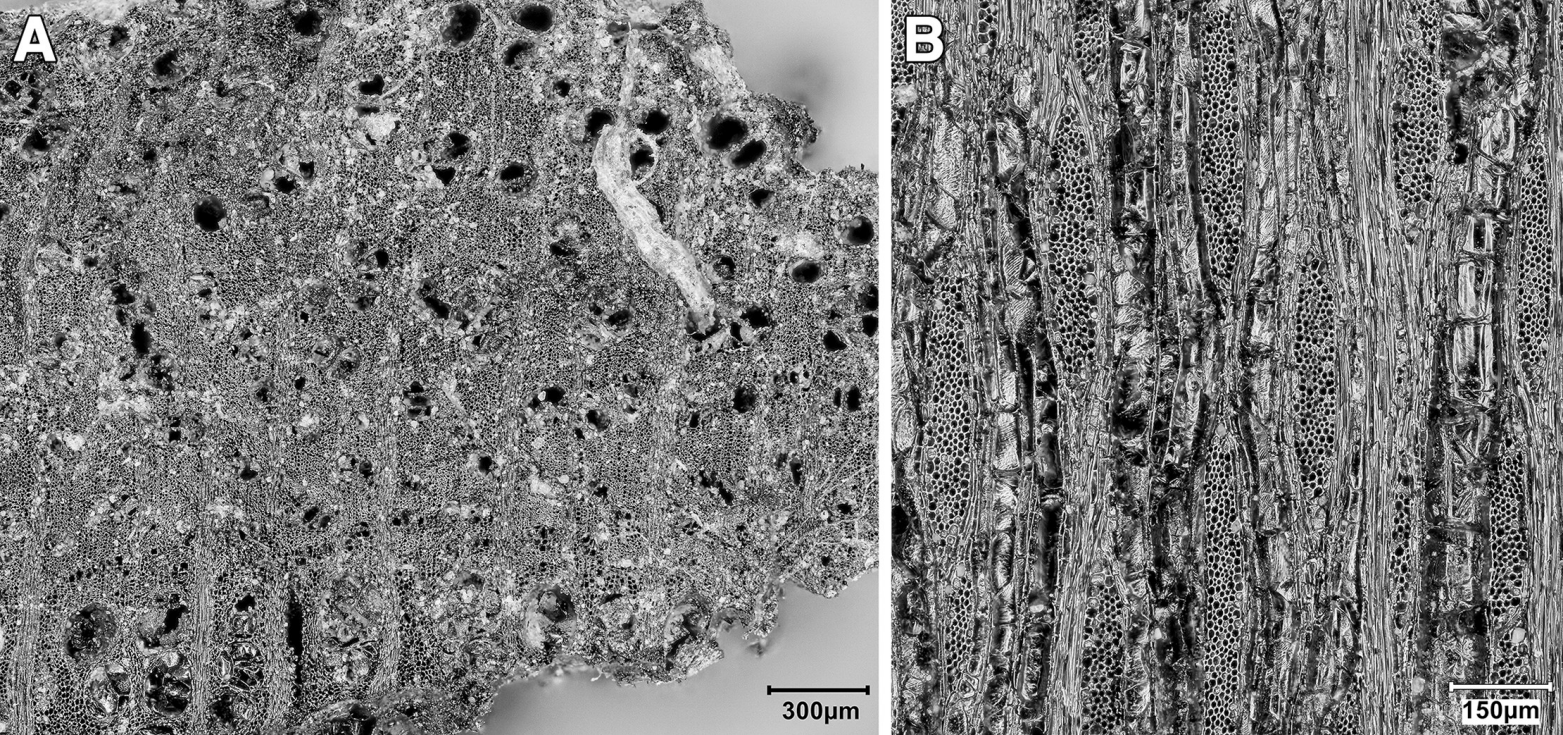
*Celtis* charcoal fragment from context AAS (photos by E Asouti). (A) TS plane; (B) TLS plane.

**Fig 66:**
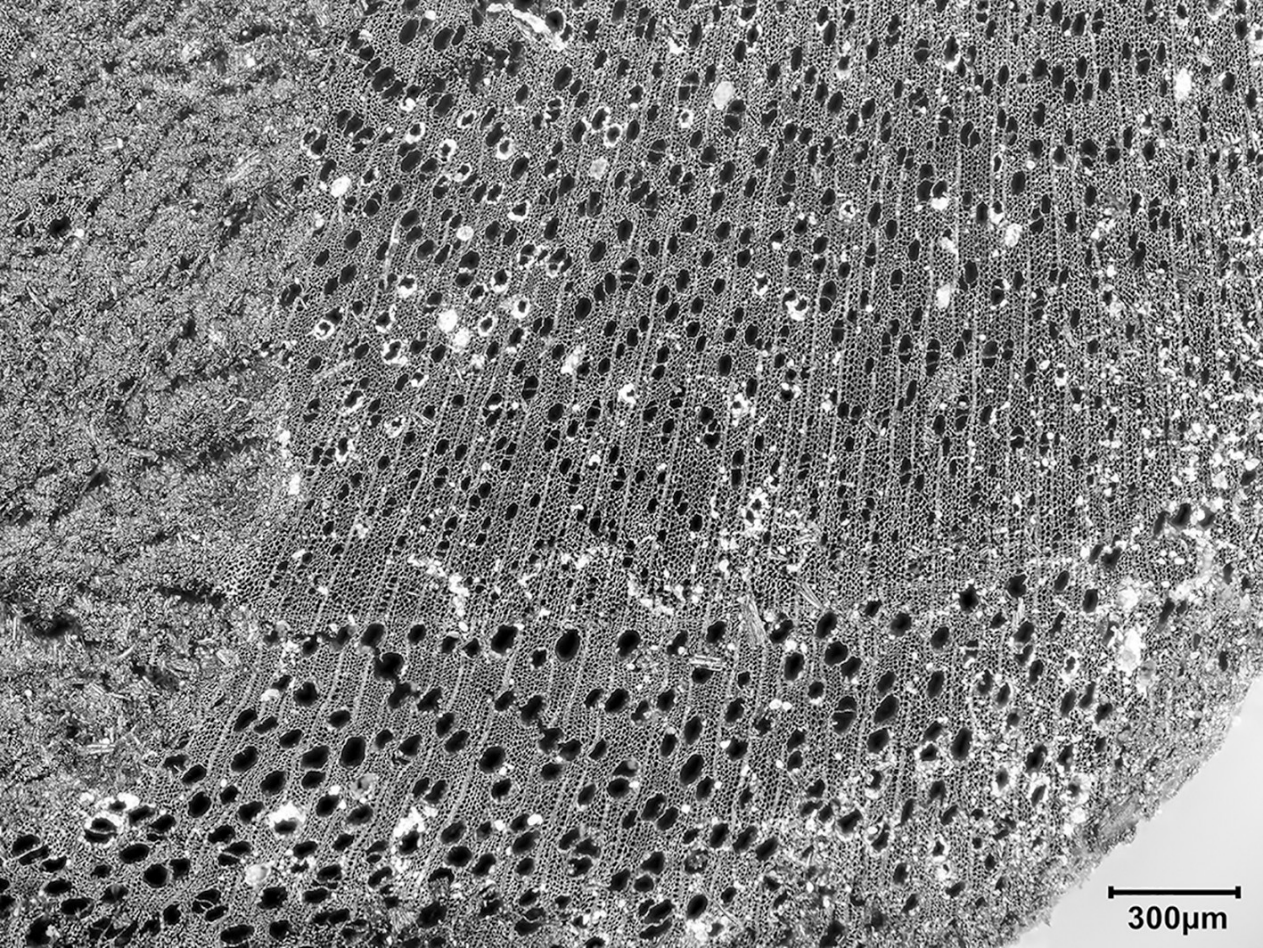
TS plane of Salicaceae charcoal fragment from context AAH (photos by E Asouti).

**Fig 67:**
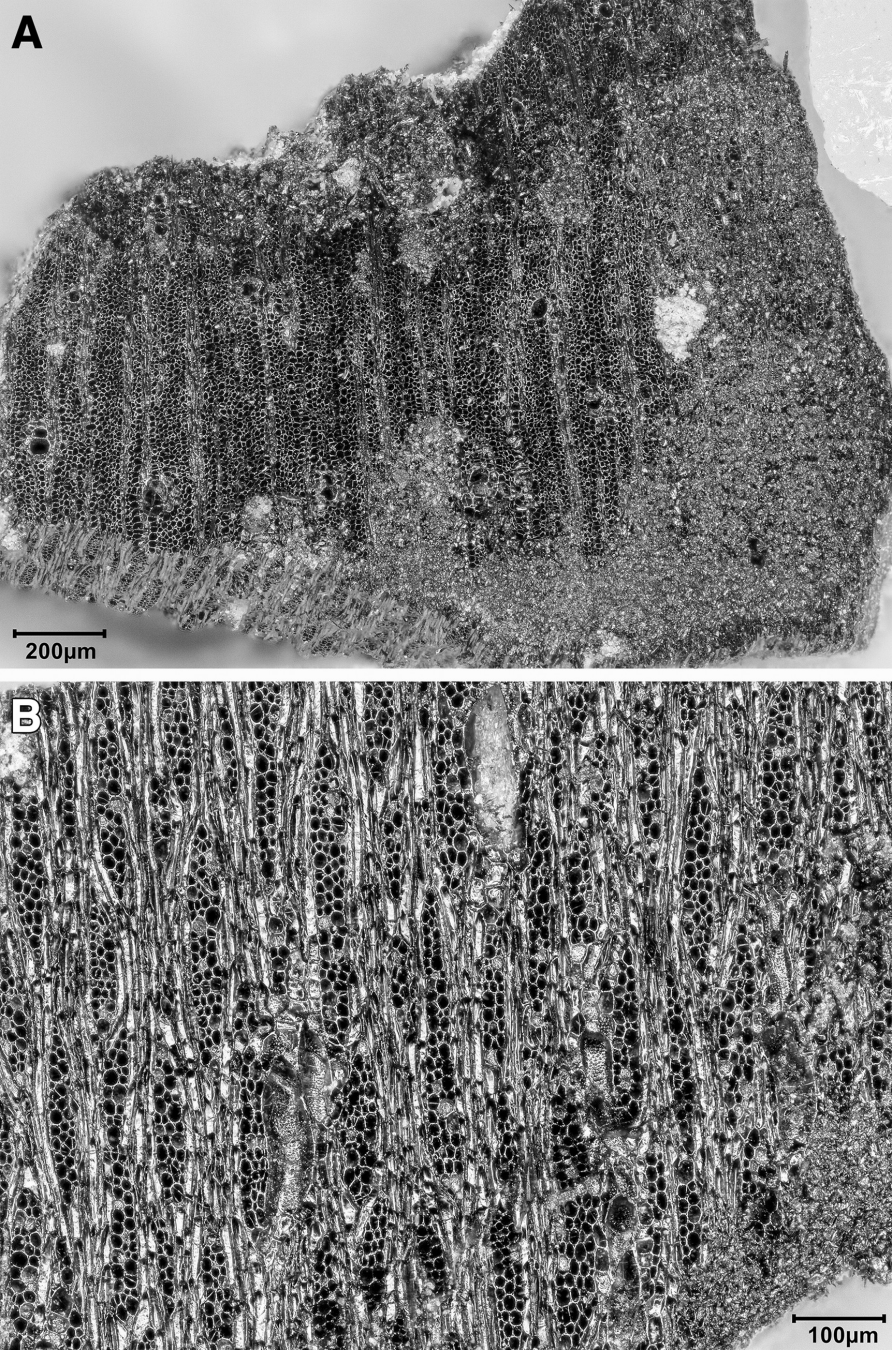
*Fraxinus* charcoal fragment from context AAS (photos by E Asouti). (A) TS plane; (B) TLS plane.

**Fig 68:**
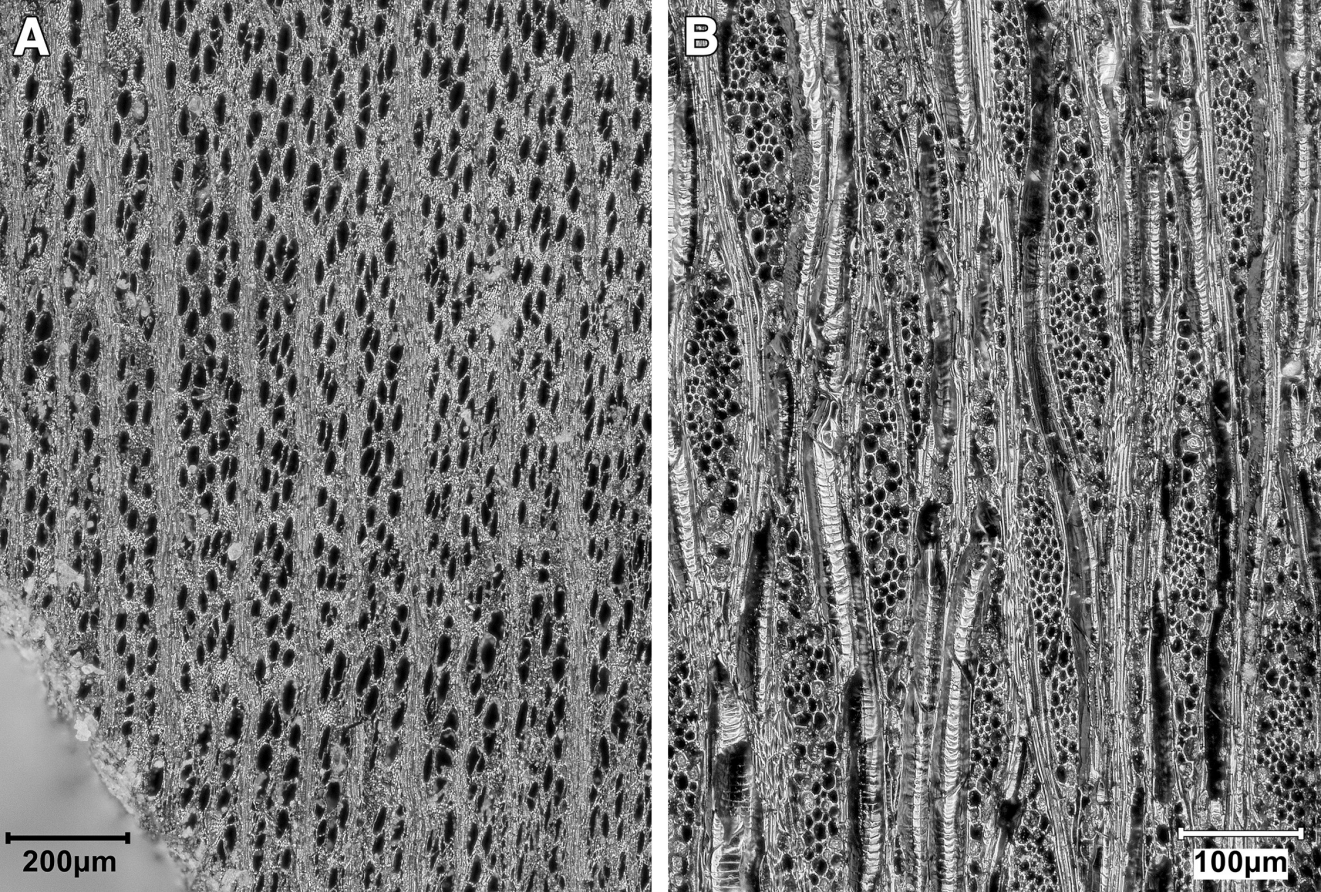
*Prunus spinosa* type charcoal fragment from context AAS (photos by E Asouti). (A) TS plane; (B) TLS plane.

**Fig 69:**
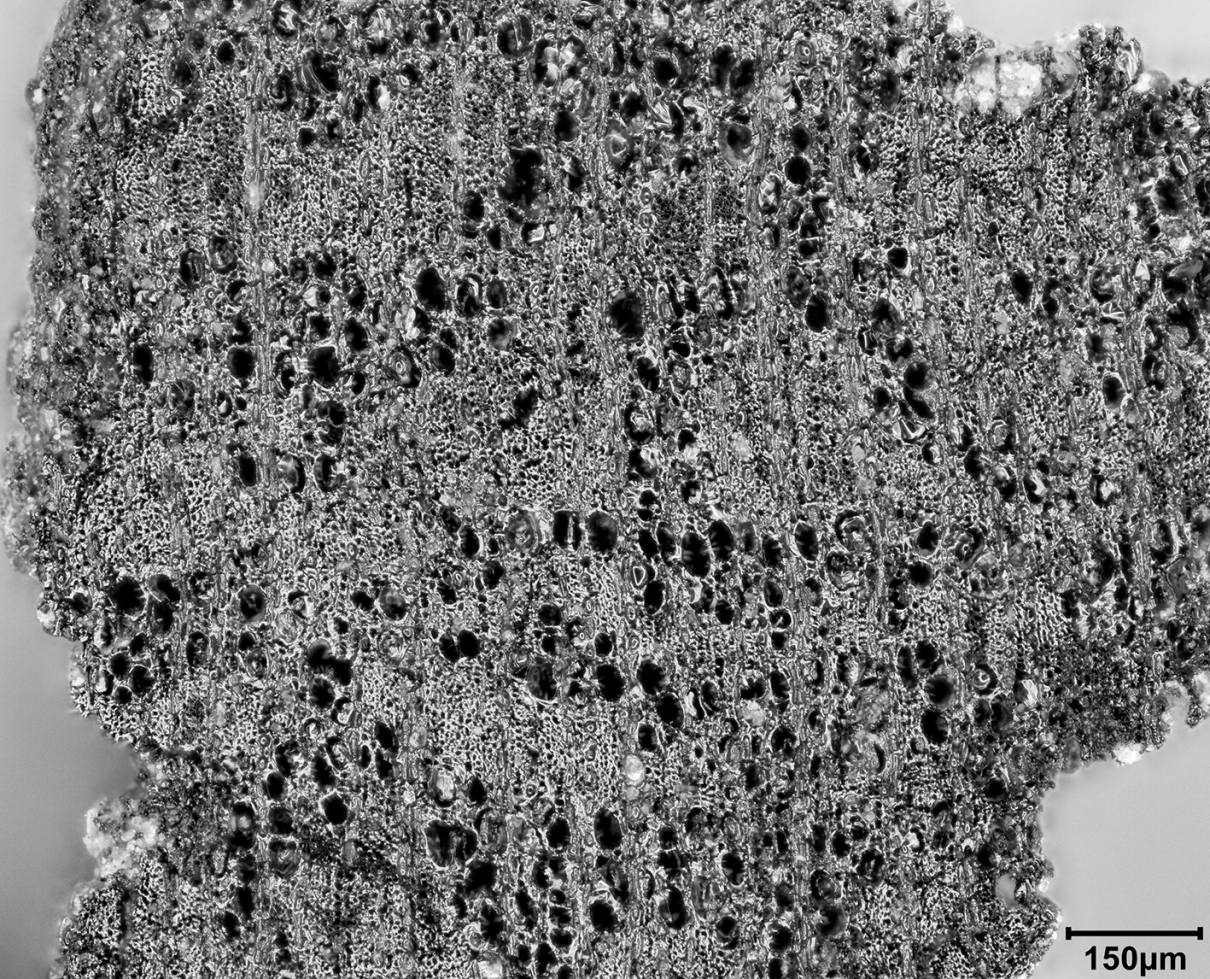
TS plane of *Rhamnus* charcoal fragment from context ACV (photo by E Asouti).

The impact of preservation conditions on charred macro-remain densities, sample composition and taxon representation, is even more acutely demonstrated in the Area A anthracological assemblage (Fig 52 and S16Α and S16Β Table). More than 90% of the wood charcoal NISP originated in Phase 1 samples, which also contained the highest densities of anthracological remains: 0.27 wood charcoals >2mm per litre of sediment, compared to 0.03 for Phase 2 and 0.07 for Phase 3. *Amygdalus* (Figs 62 and 63) is by far the most common charcoal taxon in Phase 1, being present in 22 out of 29 sampled contexts and amounting to 252 fragments out of a total NISP of 308. Although the majority of identified fragments were too small to permit wood calibre estimations [122, 123] most displayed the curved growth rings and/or wide rays typically associated with shrubby forms [124] (S14–S16 Figs). The second most common taxon is *Quercus* (deciduous oak) with 20 fragments found in 9/29 Phase 1 contexts (Fig 64). Other relatively ubiquitous taxa include *Celtis* (hackberry) and Salicaceae (willow/poplar) (Figs 65 and 66). A few taxa also appear sporadically including *Fraxinus* (ash), *Prunus spinosa* (blackthorn) and *Rhamnus* (buckthorn) (Figs 67–69). 2 conifer fragments may represent *Juniperus* (juniper) judging from the very short ray height (1–4 cells) visible on the TLS plane, although they were too small (~2mm) to permit a more secure identification. A single twig fragment possibly representing Asteraceae indet. (not of the *Artemisia* type) was also retrieved from context AAT.

The preliminary results of the analysis of phytolith samples (detailed in S3 File) are largely in agreement with the macrobotanical finds. They indicate that Cyperaceae were used at PG while there is also evidence for the collection of reeds (*Phragmites*). Algae and sponge spicules were found in all analysed contexts suggesting that wetland plants and perhaps even clay lumps were brought to the site from wet habitats nearby. Both grasses and dicots are well represented in the analysed phytolith assemblage, especially in Phases 1 and 3. Although the phytolith evidence for grass seed processing is limited, this may reflect the poor preservation of husk phytolith forms compared to the over-representation of leaf and stem morphotypes, and/or spatial variation in the deposition and taphonomic histories of seed debris (see below, **Assemblage formation processes** section). Burning is ubiquitous, particularly in grass leaves bulliforms and in some *Phragmites* bulliforms. Most analysed samples also contained starch granules. Starches are common in many plant parts especially in seeds, nuts, roots and tubers. Pending further taxonomic analyses their ubiquity in the studied Area A samples is another good indicator for the collection and processing of edible plant resources at PG during its late Pleistocene habitation.

#### Assemblage formation processes

**Fig 70:**
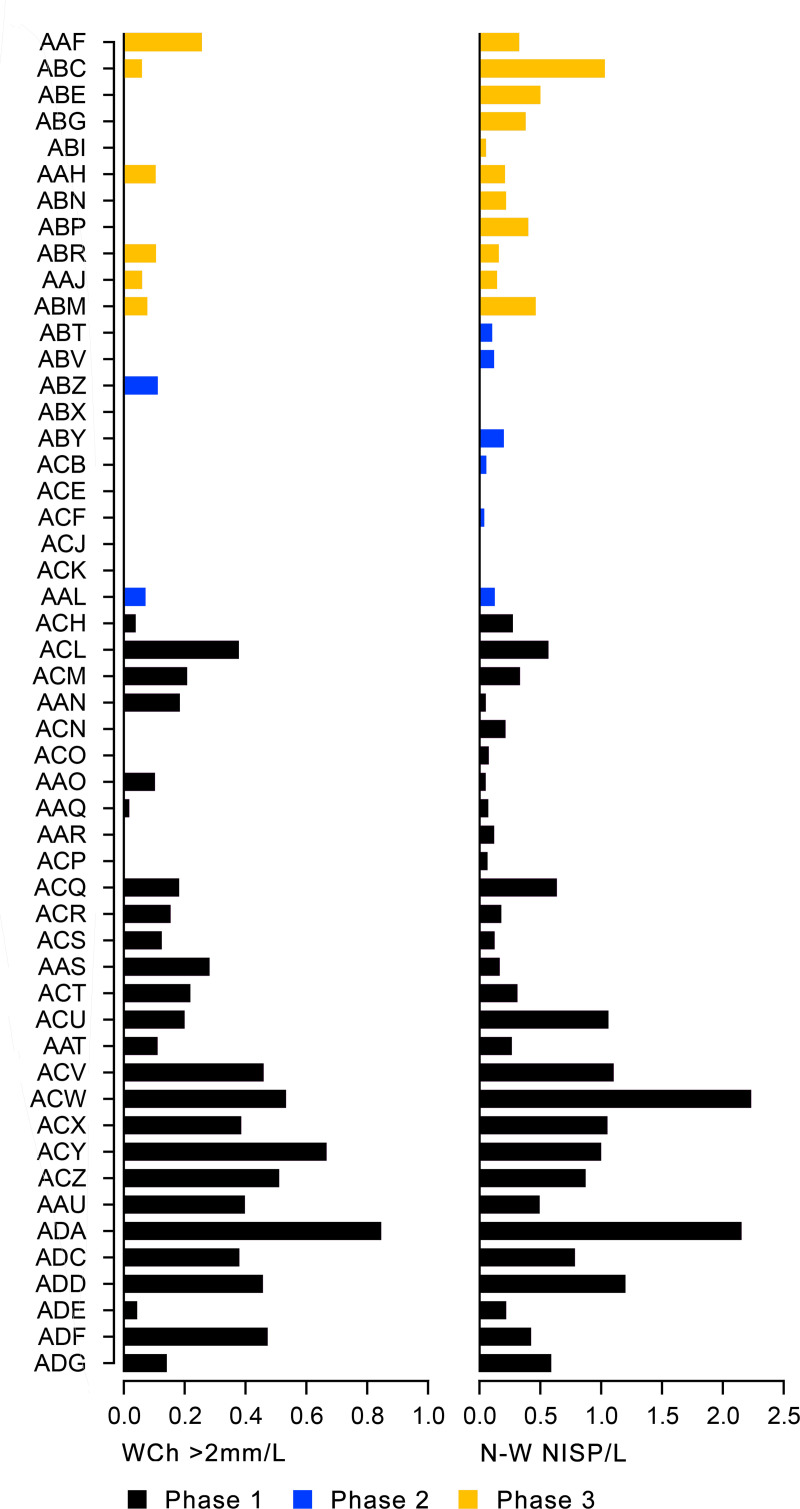
Botanical densities (number of items per litre of sediment) of all sampled Area A late Pleistocene contexts (excluding context ACD).

The Area A late Pleistocene archaeobotanical assemblage is generally characterised by low charcoal densities, expressed as the number of wood charcoals >2mm (WCh) and the now-wood NISP (N-W) per litre of sediment. Charcoal preservation varies significantly between Phases 1–3 (see Figs 52–70, and S16Α and S16Β Table). The highest densities occur in the deepest and chronologically earliest Phase 1 deposits also including the basal layers above the bedrock. It is apparent that with few exceptions (e.g., contexts AAO, AAQ, AAR, ACP) the concentrations of rockfall in Phase 1 contributed to the survival of charred macro-remains by limiting the negative impacts of seasonal ground moisture evaporation and sediment leaching on charcoal preservation. Out of 29 Phase 1 samples only 4 (ACN, ACO, AAR, ACP) did not contain wood charcoals, whilst every sample contained non-wood macrofossils. By comparison, the largely rockfall-free deposits of the stratigraphically latest Phase 3 contained much lower charcoal densities. 5 out of 11 Phase 3 samples (ABE, ABG, ABI, ABN, ABP) were devoid of wood charcoals >2mm.

A closer inspection of the per phase density ratios, including the ratio of non-wood NISP (with and without tuber/parenchyma items) to wood charcoal and the nutshell to wood charcoal ratio, demonstrates clearly the variation in the preservation of different groups of charred macro-remains with depth (see S16B Table). Wood charcoals, tubers and nutshell are better represented in Phase 1 deposits by comparison to charred seeds which appear to have survived better than wood charcoals in Phase 3 samples (S16A and S16B Table). This may reflect changes through time in the topography of the PG cave floor with sediment accumulation resulting in the greater exposure of larger and/or more angular charred debris to mechanical damage, e.g. via trampling, alongside seasonal wetting-drying cycles and sediment leaching.

Phase 2 samples contained the lowest charcoal densities of the entire sequence. From a total of 12 samples, 4 were completely devoid of charred macro-remains (ABZ, ABX, ACJ, ACK). Of the remaining 8 only 2 (ABZ and AAL, the latter at the base of Phase 2 deposits) contained a few wood charcoals alongside the *Amygdalus* C-14 wood charcoal sample that was collected in the field from ABV. 5 samples (ABT, ABV, ABY, ACB, ACF) contained sporadic non-wood remains with 3 or less NISP per flot fraction. The sole exception was ACD, a small (0.45 litre) context (not shown in Fig 70) which contained 5 charred tuber/parenchyma fragments and 1 legume seed (S16A Table). Neither the Area A radiocarbon age model nor the artefactual, micromorphological and stratigraphic evidence indicate a break in sediment deposition during the timespan represented by Phase 2. The low charcoal densities thus likely reflect minimal rates of charred plant debris accumulation in Area A during this period. They might also indicate the comminution and destruction of the fragile charred plant remains transported into Area A from the inner cave chamber through slopewash deposited in small water pools (the concreted clay surfaces abounding in Phase 2) whereby charcoal particles were obliterated by repeated cycles of wetting and drying. Significantly, Phase 2 has also generated much lower faunal NISPs compared to Phases 1 and 3. Overall, the combined macrobotanical and faunal records point to a low frequency of fire-related subsistence activities at PG in the timespan represented by Phase 2 (~17,000–16,600 cal BP). It is possible that the cave was more infrequently visited during this period, which overlaps with the onset of a renewed phase of climatic aridity lasting until ~15,200 cal BP as indicated by the lake Zeribar palaeoclimatic sequence (Fig 4).

The low wood charcoal densities reflect in part the nature of the excavated deposits, adjacent to the cave entrance where fuel wood waste was more susceptible to trampling, and in part the location of Area A further away from the inner cave chamber, excavated by the Iraq-Jarmo project, which probably received the bulk of charred plant debris accumulated from firewood use and associated food preparation and consumption activities. Overlying this spatial filter are the effects of the post-depositional decay of charred plant debris: the highest charcoal densities were retrieved from the stratigraphically deepest Phase 1 deposits, where plant remains were sealed under dense rockfall layers that mitigated the destructive impacts of sediment leaching and seasonal wetting-drying cycles on charcoal preservation. A similar positive correlation of charcoal density with increasing depth has been observed by Colledge [125] at the early Natufian site of Wadi Hammeh 27 in the Jordan valley. At PG, the effects of leaching on plant densities must have been particularly pronounced, which is also suggested by the low frequency and poor preservation of phytoliths in Area A contexts of all phases (see S3 File).

The low densities of non-wood macrofossils are also not unexpected. This is a phenomenon long recognised in pre-agricultural sites, and stems from the nature of Palaeolithic plant exploitation involving a great diversity of species used in various forms (raw, cured and/or cooked) for food, raw materials, medicinal and other purposes. By comparison, Neolithic sites usually have much higher charred plant densities, due to their focus on the intensive management of stored staple grain, and the higher volumes of charred plant debris generated by daily food processing and cooking activities [126]. Differences in preservation also arise from the morphology of the species involved. Gathered plants producing dense and inedible waste debris (e.g., the nutshell that is so ubiquitous at PG) are more likely to be deliberately discarded in fires, and thus have a greater chance of entering the archaeobotanical record compared to seeds and nutmeat that would only be accidentally burnt during food preparation [127]. Low charred macro-remain densities may also result from the transient nature of human habitation in caves and rock-shelters [128] although exceptions to this pattern are also known from Palaeolithic cave sites in the Eastern Mediterranean [124, 129].

Perhaps the most important conclusion arising from the evaluation of the PG archaeobotanical assemblage formation processes is that small-scale sampling strategies involving few samples and/or low sediment volumes processed by manual dry sieving or bucket flotation, would have resulted in the erroneous assumption of minimal (or no) plant gathering and use at PG during its late Pleistocene habitation. Archaeobotanical recovery at PG shows that intensive sampling of large sediment volumes processed with machine-assisted flotation can overcome to a significant degree poor organic preservation and provide a reasonably representative picture of botanical assemblage composition. This strategy is thus eminently appropriate for investigating Palaeolithic plant exploitation and vegetation ecologies in the Zagros and other Middle Eastern regions facing similar organic preservation challenges.

#### Late Pleistocene plant exploitation

As discussed above, the PG archaeobotanical assemblage has been transformed by various taphonomic filters and post-depositional processes that have severely impacted its preservation and therefore its representativeness for reconstructing the full spectrum of late Palaeolithic plant selection and use. In order to mitigate these largely anticipated impacts we sought to maximise the spatial coverage and volume of field sampling, and the intensity of laboratory analyses. Coupled with targeted phytolith sampling, this rigorous approach has provided an unprecedented window into Zarzian plant exploitation and the late Pleistocene vegetation of the NW Zagros piedmont zone.

Based on the available evidence, it can be reasonably concluded that plant subsistence was focused on dryland resources including small-seeded grasses and legumes alongside *Lathyrus/Vicia* and at least some large-seeded grasses such as *Stipa* and *Hordeum*. Wild almond nuts (*Amygdalus*) and, possibly, *Pistacia* too were another major plant subsistence component. The regular collection of tubers may also be surmised from the abundance and ubiquity of charred parenchyma tissues, although which taxa and habitats were targeted can only be clarified after the completion of SEM analyses. The occurrence of Cyperaceae charred stems and phytoliths in samples from all phases might represent waste discarded into the fire following the cleaning, preparation and cooking of sedge tubers as food. The gathering of Brassicaceae (mustard family) may also be hypothesised based on the occurrence of their seeds in 6 different Phase 1 contexts.

Overall, despite preservation limitations, the combined Area A macro- and micro-botanical assemblages point to the exploitation of a diverse spectrum of plant taxa, which were gathered during at least 4–6 months in the annual cycle (i.e., in the late spring, summer and early autumn) as suggested by the presence of grass and legume seeds and nutshell. It must be stressed here that this reconstruction of seasonality represents a conservative estimate based on the ubiquity of taxa that were preferentially preserved in the PG macrobotanical assemblage (see above, **Assemblage formation processes** section). Tubers, alongside plants that have not been preserved, could also have been collected in different seasons. The same holds for fuel wood, as wood charcoals did not preserve seasonality indicators such as terminal growth rings. Other seasons of habitation (i.e., winter and early spring) are thus entirely possible although they remain, by necessity, invisible in the PG archaeobotanical record. Overall, the available evidence suggests that plant exploitation was opportunistic, aimed at maximising the resources available within a relatively short radius around the cave including both dryland and wetland habitats in direct proportion to their distribution in the local landscape.

This picture of Zarzian plant subsistence at PG based on grasses, legumes, nuts and tubers is compatible with the evidence available from other Palaeolithic sites in the Eastern Mediterranean. *Lathyrus*/*Vicia* seeds, rich in protein and carbohydrates, have been found at sites dating from as early as the Middle and early Upper Palaeolithic [129–131] alongside phytolith evidence for the exploitation of grasses and sedges (reviewed in [132]). Archaeobotanical finds from Ohalo II, Kharaneh IV and Hayonim cave in the southern Levant, and the Öküzini and Karain B caves on the south Anatolian coast have demonstrated that a diverse spectrum of grasses, legumes, nuts and tubers/sedges were variously exploited by Epipalaeolithic foragers across the region [133–136]. The recurrent presence at PG of small-seeded grasses and legumes is also compatible with the evidence from the uniquely well-preserved waterlogged assemblage of Ohalo II indicating the prominent place of these plant groups as staples in SW Asian pre-agricultural plant subsistence economies [137].

The bulk of fuel wood was procured from wild almond and deciduous oak shrubs and trees, which must have been common in the vegetation proximate to the cave. Salicaceae (willow/poplar) shrubs were another source of firewood, most likely collected from the wadi adjacent to the cave. Other wood taxa were only sporadically present in Phase 1 samples therefore it is not possible to evaluate more precisely their contribution to late Palaeolithic fuel wood use. Nonetheless, they are useful indicators of the floristic composition of the local plant habitats, thus providing a baseline for the qualitative assessment of late Pleistocene woodland vegetation and climate conditions.

#### Late Pleistocene vegetation and climate

Previous palaeovegetation and palaeoclimatic reconstructions for the late Pleistocene in northern Iraq have largely drawn on the results of palaeoecological analyses undertaken at the high-altitude (~1300m a.s.l.) intramontane lake Zeribar (35°32'0"N, 46°7'0"E) located ~90km measured as a straight line to the east of PG, on the Iranian Zagros rain shadow (Figs 1 and 2 and S1 Fig). Based on these studies, it has been proposed that the period between the LGM and the start of the Holocene at ~11,700 cal BP was characterised by dry and cold climatic conditions that inhibited the establishment of woodland vegetation [138, 139] (see also Fig 4). Pollen analysis previously undertaken by Leroi-Gourhan at Zarzi (~26km NE of PG) on sediment samples taken from the terrace in front of the rock-shelter excavated by Wahida in 1971, appeared to confirm this picture. Trees (*Quercus*, *Amygdalus* and *Pinus*) and grasses registered very low frequencies (<1% and <5% respectively) contrasting with the abundance of Cichorioideae (syn. Liguliflorae; a subfamily of the Asteraceae including dandelions and chicories) that dominated the pollen assemblage (>80%) ([10]: pp.33-36). Leroi-Gourhan also studied a small pollen assemblage (NISP = 84) from Layer B2 at Shanidar cave (~160km NE of PG). It contained somewhat higher proportions of trees (*Quercus* 6%; *Pistacia* ~1%; *Juniperus* ~4%; *Pinus* ~3%). Grasses were better represented (~30%) than at Zarzi, while the Cichorioideae frequencies were much lower (<10%) ([10]: pp.33-36).

Despite its high pollen count (NISP = 4502) the Zarzi pollen assemblage is particularly problematic due to the over-representation of the Cichorioideae and the absence of *Artemisia*, a typical indicator of herbaceous steppe vegetation across SW Asia. Bottema [140] has observed that unusually high proportions of the Cichorioideae in fossil pollen spectra are suspect, as they have no known parallels in modern vegetation analogues and pollen productivity estimates. Cichorioideae pollen accumulations near the entrances of caves and rock-shelters located in areas characterised by warm and dry summers are likely to result from recent pollen infiltration caused by burrowing bees. Cichorioideae pollen grains are furthermore easily identifiable and highly resistant to corrosion. For all these reasons Bottema concluded that Cichorioideae should be excluded from quantitative evaluations of prehistoric pollen spectra. Based on his observations, it seems reasonable to infer that Zarzi represents a typical example of a poorly preserved and highly biased archaeological pollen assemblage that is unrepresentative of the local late Pleistocene vegetation and climatic conditions.

For the timespan corresponding to the PG late Pleistocene habitation the nearest reliable source of pollen data is lake Zeribar [30, 138, 139]. The PG Area A sequence (~19,600–13,000 cal BP) overlaps chronologically with the Zeribar pollen assemblage zones (PAZ) 3b/middle-upper and 4/lower correlating respectively with the late Pleniglacial and the Lateglacial, which at Zeribar are dated between ~21,000–12,600 cal BP (see also Fig 4). PAZ 3b/middle-upper points to the prevalence of Chenopodiaceae-*Artemisia* dwarf shrub steppe. *Quercus* was absent from the Zeribar upland catchment while *Pistacia*, *Acer* and *Juniperus* were sporadically present. In PAZ 4/lower an increase in temperature is indicated by a reduction in *Artemisia* and a coeval increase in Chenopodiaceae, accompanied by a modest increase in grasses and *Pistacia*. However, the continuing absence of *Quercus* suggests that conditions were still too dry for oak growth, with annual precipitation <300mm. Comparisons drawn by van Zeist [139] with two pollen sequences obtained from the Lalaband and Nilofar springs situated at ~1300m a.s.l. in the Iranian central Zagros near the city of Kermanshah (Figs 1 and 2) provide additional support for the prevalence of dwarf shrub steppe vegetation in the central Zagros during the Pleniglacial.

The PG Area A macrobotanical assemblage provides the first radiometrically dated palaeovegetation archive for the late Pleniglacial and Lateglacial vegetation of the Iraqi Zagros piedmont zone. Amongst the arboreal taxa, the frequency and ubiquity of *Amygdalus* and the regular presence of deciduous *Quercus* in Phase 1 (late Pleniglacial) samples indicate that during this period the Baranand limestone ridge and the adjacent Bazian valley provided sufficient conditions for the growth of *Amygdalus-Quercus* woodland, with deciduous oak charcoals possibly originating from the drought-tolerant *Q*. *brantii*. The occasional presence of *Pistacia* nut fragments amongst the non-wood charred macrobotanical remains of Phase 1 further suggests that *Pistacia* was probably present near the cave during this period. Wood charcoals of other constituent taxa of the Kurdo-Zagrosian forest such as *Rhamnus*, *Prunus spinosa*, *Celtis* and *Fraxinus* also occur in some of the better-preserved Phase 1 anthracological samples. The sporadic presence of Salicaceae charcoals and Cyperaceae in samples from all phases, further points to the existence of riparian woodlands and wetland habitats along watercourses in the Bazian valley and the wadi adjacent to PG.

By contrast, there is no evidence for the use as fuelwood of shrubby Chenopodiaceae and *Artemisia*, the two key indicator taxa of dwarf-shrub steppe vegetation. Both *Artemisia* (including *A*. *fragrans* and *A*. *herba-alba*–according to van Zeist the species most likely represented by *Artemisia* pollen in the Zeribar sequence) and perennial shrubby chenopods are ethnographically well-known and historically intensively exploited fuelwood sources in the Iranian plateau and the lowland plains of Iraq [141–144]. Some chenopod shrubs are also reported to be good sources of readily available deadwood as they die back to the ground after they have shed their seeds ([145]: p.63). The complete absence of Chenopodiaceae and *Artemisia* wood charcoals from the Area A anthracological samples suggests that neither taxon was routinely gathered as fuel by the late Pleistocene inhabitants of the cave. They are therefore highly unlikely to have been dominant components of the local vegetation.

In common with other insect-pollinated members of the Rosaceae *Amygdalus* is notoriously under-represented in pollen diagrams. Its absence from the Zeribar pollen sequence, dominated by the wind-pollinated *Artemisia* and Chenopodiaceae, might thus mask the actual presence of almond shrubs in the Zeribar catchment during the late Pleniglacial. At present, *Amygdalus*-dominated woodland habitats are known from few areas in the Iranian southern Zagros, including the *Amygdalus scoparia* open woodlands found in Khuzestan and on the western slopes of the Fars mountains bordering the Kurdo-Zagrosian oak forest at altitudes ~750-1250m a.s.l. and ~250-300mm of annual precipitation. Zohary describes their structure as comprising widely spaced stands of 1-3m high almond shrubs with rare *Pistacia atlantica* trees and a ground cover of traganthic and herbaceous species ([40]: pp.35, 43). If a similar vegetation structure existed in the Zeribar lake catchment it would have left little if any trace in the pollen record except for the under-representation of *Pistacia*. Unfortunately, due to the lack of systematic archaeobotanical sampling, anthracological records from late Palaeolithic sites in the Iranian Zagros are extremely limited [146]. However, *Amygdalus* alongside the other two poor pollen dispersers *Juniperus* and *Acer* are well-known constituents of late Pleistocene pioneer vegetation communities in the Eastern Mediterranean. All three have been reported from late Palaeolithic anthracological assemblages in central and southern Anatolia, the Levant and the south and central Greek mainland [124, 147–152].

On the whole, the taxonomic composition of the Area A archaeobotanical assemblage indicates that the late Pleniglacial and Lateglacial vegetation of the NW Zagros piedmont zone consisted of *Amygdalus*-*Quercus* open woodland, with a ground flora dominated by small- and large-seeded grasses. The cold-tolerant *Vicia/Lathyrus* are likely to have been abundant in open woodland habitats on rocky hillslopes as well as in valleys and upland meadows. *Pistacia* and a suite of other Kurdo-Zagrosian and riparian woodland taxa (*Celtis*, *Prunus*, *Fraxinus*, *Rhamnus*, Salicaceae) also occurred in sheltered habitats on the Bazian valley and the wadis draining into it. Although *Prunus spinosa* is largely absent from the regional woodland vegetation today, it has been previously reported by Zohary ([153]: p.376) from the Qaradagh ridge ~40km to the SE of PG (S2 Fig) one of the few areas on the Iraqi Zagros piedmont zone in which Kurdo-Zagrosian forest persists in significant density today. The absence from the PG anthracological assemblage of Maloideae (*Pyrus* and *Crataegus*, both major components of the Kurdo-Zagrosian forest) and the potential presence of the cold- and drought-tolerant *Juniperus* suggest that climatic conditions were cooler and drier than at present. This observation accords well with other late Palaeolithic anthracological assemblages from the Eastern Mediterranean in which Maloideae do not become abundant until the start of the Holocene [124].

These data, alongside the absence of Chenopodiaceae and *Artemisia* from the Area A anthracological assemblage, suggest that climate was cool and moderately dry, with annual precipitation averages in the range of ~330-500mm (i.e., higher than at Zeribar judging from the absence of the wind-pollinated *Quercus* from its catchment). At present, higher precipitation on the windward side of the western Zagros is caused by moisture bearing winds from the Eastern Mediterranean reaching this area through the Homs Gap in western Syria alongside winter rainfall from the Arabian Peninsula. This generates a strong orographic effect on the distribution of precipitation, with maximum values (>1000mm) recorded for the highest parts of the NW Zagros range, while on its rain shadow towards the Iranian plateau precipitation decreases abruptly to 300-500mm ([153]: p.163, [154]). Wright [154] had hypothesised that this contrast was probably more accentuated during the last glacial phase of the Pleistocene: the outer flanks of the Iraqi Zagros range experienced higher precipitation (which, combined with the lower temperatures, probably resulted in the depression of its snowline to ~1500-1200m a.s.l.) due to the increased frequency and intensity of the storms that reached Mesopotamia from the Mediterranean. At the same time, drier conditions prevailed in the Iranian plateau due to the intensified Siberian anticyclone in the winter blocking the penetration of these storms beyond the western Zagros crests. The PG archaeobotanical record adds substantive new data to Wright’s hypothesis by pinpointing clear differences in the floristic composition of the late Pleniglacial vegetation of the NW Zagros piedmont zone and the Zeribar intramontane basin located in the cooler high Zagros upland zone closer to the Iranian plateau. Furthermore, these data provide the first direct evidence anchored to a secure radiometric chronology for the existence of late Pleistocene grassland and woodland refugia (including deciduous oaks, legumes and grasses) on the NW Zagros piedmont zone, as originally contemplated by van Zeist [155].

The taxonomic composition of the Area A archaeobotanical assemblage does not change much between Phase 1 (late Pleniglacial) and Phase 3 (Lateglacial) with regard to the presence of the principal charcoal taxa (*Amygdalus*, *Quercus*) and the persistent occurrence of grasses, legumes, other seed taxa and tubers. It is possible that the slightly higher diversity of seed taxa found in Phase 3 is related to an expansion of grassland habitats in the Lateglacial, as indicated by the zooarchaeological evidence for increasing onager hunting during this period. Although less charcoal and fewer arboreal taxa were retrieved from the Phase 3 flotation samples (Fig 52 and S16A and S16B Table) this more likely reflects the poorer preservation of charred plant macrofossils in the upper part of the PG sequence than a diachronic shift in the availability and floristic composition of the local woodland vegetation. The sole exception may be the timespan corresponding to the Phases 2–3 boundary. The Area A age model has indicated a possible gap in the habitation of the cave between the end of Phase 2 at ~16,600 cal BP and the start of Phase 3 at ~14,200 cal BP, which in turn suggests a synchronous drop in local plant resource availability. The intervening ~2 kyr timespan partly overlaps with the climatic aridity peak previously identified in the Zeribar sequence between ~16,000–15,300 cal BP–coeval with the Heinrich Event 1 (HE1) peaks at ~16.2 and 15.1 ka BP [156]–described by the authors of the Zeribar study as “the highest [aridity] levels of the entire record” ([30]: p.313) (see also Fig 4). High aridity levels were caused by the dramatic reduction in the strength and frequency of storms over the Eastern Mediterranean region during this period [157]. Zarzian habitation at PG resumed following the onset of the Lateglacial at ~14,700 cal BP, which suggests that by the start of Phase 3 at ~14,200 cal BP local vegetation resources had been re-established at levels at least similar to those of Phase 1.

## Discussion

The EFEC project excavations at PG and the new radiometric chronology for the cave presented in this paper provide the first conclusive evidence that the Epipalaeolithic of the NW Zagros extends from the LGM to the end of the Lateglacial (~19,600–13,000 cal BP). In conjunction with the materials previously published from the sole other Zarzian site that has been radiometrically dated, TB75 in the Fars province of southern Iran, the results of our studies firmly establish that the Zarzian cultural horizon developed synchronously across the vast geographical expanse of the Zagros range (Fig 1) encompassing highly diverse and contrasting ecologies and landscape settings (for a recent summary of undated Zarzian sites discovered by field surveys in the central and southern Zagros see [158]). This evidence settles the question whether the NW Zagros piedmont zone was inhabited (and indeed habitable) during the late Pleniglacial. *Contra* previously widely accepted hypotheses, the PG archaeobotanical record, also supported by the zooarchaeological habitat analysis results, demonstrates conclusively that the ‘hilly flanks’ hosted a diverse range of grassland, woodland and wetland habitats that were extensively exploited by Zarzian groups, in common with patterns of plant exploitation observed across SW Asia and the Eastern Mediterranean throughout the Epipalaeolithic period.

The settlement typology originally proposed by Hole and Flannery ([31]: pp.162-165) for the late Palaeolithic of the Zagros modelled a habitation pattern comprising three main occupation types: seasonal base camps, butchering stations, and transitory stations/hunting watches. Hole and Flannery tentatively placed PG in the seasonal base camp category ([31]: p.163) on the basis of its location overlooking the Bazian valley, proximity to water, fuel and game, and the presence of dense artefact concentrations and food remains in the occupation deposits excavated by the Iraq-Jarmo project. Their prediction was that base camp sites would contain all skeletal parts of goat-sized animals but only selected carcass parts for larger cattle/onager/cervid sized taxa. Olszewski ([33]: pp.214-215) also deployed mammalian skeletal part representation to argue that the presence of crania at Warwasi indicates that whole carcasses must have been brought to the site, even if some parts may have been transported elsewhere after initial processing on-site.

Turnbull and Reed’s 1974 study of the PG faunal remains contradicted Hole and Flannery’s scheme. In their report the mammalian skeletal-part patterns demonstrated the predominance of foot bones ([65]: p.130 & Table 3) leading them to propose that animals would have been hunted at a distance from the cave, with meat butchered at the kill returned to the cave in skins with feet attached (an example of the ‘schlepping’ theory popular at the time). They argued that whole carcasses were not brought to the site for processing; many elements would have been left at the kill, which led them to conclude that PG did not serve as a (semi-) permanent camp but rather a hunting base ([65]: pp.140-141). Turnbull and Reed also found hunting (of the dominant onager in particular) to have focused on adult animals rather than juveniles. While they did not find evidence for hunting seasonality, they hypothesised that occupation would have avoided the wet winter-spring months ([65]: p.140).

The EFEC project zooarchaeological results from PG contribute new evidence to these earlier interpretations of site function. The inclusion of undiagnostic mammal size-class counts to the skeletal part analyses shows the presence of numerous fragments of skull, girdle bones (scapula and pelvis) and limb-bones of large and medium-sized animals. Although these fragments were too broken to be diagnostic of species, their inclusion changes the body-part representation entirely. Far from being made up of only (or even primarily) foot bones and mandibles, the PG bone assemblage provides strong indications that complete carcasses of all game animals (aurochs, red deer, onager, fallow deer, boar, caprines and gazelle) were brought on-site for processing and thus, by inference, that they were hunted in close proximity to the cave. The full suite of carcass processing–skinning, evisceration, dismemberment and meat removal–likely took place on-site or close by, even if not visible, since these are prerequisites to intensive marrow extraction of which the PG faunal assemblage has provided strong evidence even on small meatless bones. Overall, the inferred range of processing activities alongside the diversity of faunal taxa (large hunted prey, smaller trapped animals such as fox and hare, and small collected resources like tortoise) suggest that PG likely represents a long-term seasonal occupation. This new evidence does not support an interpretation of PG as a temporary hunting and/or butchering locale, which would have been expected to contain a narrower range of skeletal elements and faunal taxa.

A similar picture arises from the consideration of the chipped stone evidence. Raw material procurement involved the provision of largely unworked nodules even from moderately distant sources located in the western Chamchamal valley ~30-35km to the W of PG. This in itself suggests moderately lengthy occupation episodes ranging from a few weeks to months in the spring, summer and autumn seasons (as indicated by the macrobotanical and faunal remains) also considering the significant proportions of this raw material group in the studied lithic sample. The Bazian riverbed raw material group proximate to PG was in all likelihood directly procured in tandem with foraging and hunting in the Bazian valley. The western Chamchamal valley chert sources could also have been exploited by direct procurement. The large proportions of this raw material group in the chipped stone assemblage indicate a strong orientation to movement and interaction in this direction, possibly involving logistical forays from PG and/or recurrent relocation of resident groups in the Chamchamal area. In addition, the finds of obsidian and curated short dentalia segments point to the existence of long-distance exchange networks linking the NW Zagros Epipalaeolithic groups with source areas in eastern Anatolia and the Levantine and/or Gulf coasts.

Local chipped stone production was aimed at provisioning the full range of Zarzian tool types in finished form. The presence of complete reduction sequences for most of the knapping strategies also points to prolonged occupation periods at PG ranging from several weeks to months. The complex taphonomic and post-depositional histories of the sampled stratigraphy have almost certainly contributed to the creation of time-averaged ‘palimpsest assemblages’ [159] comprising the commingled remains of short-lived task-specific activities that took place in different parts of the cave chamber during the millennia of its Epipalaeolithic occupation. However, the diversity of the tool repertoires found across all Area A phases and in Area B, and the spatial variation observed in the distribution of tool types, also point to a prevailing pattern of generalised habitation. The inhabitants of PG engaged in a very broad range of tasks performed on-site including stone and bone tool production, woodworking, hunting, butchery and marrow extraction, plant collection and processing, etc. Furthermore, although our analyses are still at an early stage (and the bulk of the materials from Howe’s trench remain unpublished) both the Iraq-Jarmo and the EFEC project excavations unearthed finished bone tools and shell beads from Zarzian strata that are also likely to have been produced on-site.

Taphonomic and preservation issues aside, perhaps the most striking aspect of the combined lithic, faunal and archaeobotanical records is the remarkable stability evidenced in material culture practices and in plant and faunal exploitation over ~6000 years of recurrent Zarzian occupations at PG. The close similarities observed in the proportions of different tool types, including quite specific scraper and microlith types, through the different phases excavated in Area A underscore the existence of strong continuities in the nature, diversity and technologies of tool use. As Olszewski and al-Nahar [159] have argued elsewhere, such patterns are often characteristic of ‘persistent places’–sites containing the cumulative palimpsests of long-term repeated and highly similar uses of particular locales in the landscape. Their assemblages, representing time-averaged behavioural signals accumulated over successive human generations and at millennial timescales, are thus strongly suggestive of enduring connections to the landscape and repetitive practices recursively constructing community identity and coherence.

More than 70 years after Robert and Linda Braidwood led the first Iraq-Jarmo project expedition in the region, it remains the case that much more fieldwork is still required, involving intensive survey and systematic excavation deploying the full suite of contemporary field sampling and laboratory techniques for the recovery and study of archaeobiological remains, before we can achieve reasonably representative reconstructions of the diversity and diachronic development of Epipalaeolithic habitation patterns in the NW Zagros. The first results of our research at PG indicate that caves and rock-shelters located on the piedmont ridges adjacent to river valleys and plains probably functioned as ‘persistent places’ that were repeatedly occupied over several millennia. Zarzi is located in a similar setting; although it remains radiometrically undated, its relatively short distance (~23km) to the NE of PG, the strong affinities displayed between the chipped stone assemblages of both sites, and (unlike PG) the apparent activity focus on the terrace in front of the rock-shelter, all raise intriguing possibilities with regard to inter-site variation and the nature of Epipalaeolithic territorial behaviours, and habitation and mobility patterns. Recent excavations by Tsuneki [116] at the open-air site of Turkaka (undated) in the Chamchamal area ~19km to the W of PG (Fig 2 and S1 and S2 Figs) have also pinpointed its potential function as a transient task-oriented camp for chert reduction and the production of tool blanks in direct proximity to raw material sources. The absence of habitation structures, the low frequencies of animal bone and artefacts other than chipped stone, and the apparent complete lack of charred plant preservation all support this interpretation. However, the lack of radiocarbon dates and the characteristics of the Turkaka chipped stone assemblage (notably the absence of microliths from the excavated deposits alongside the presence of a range of tool types) preclude for now a more precise evaluation of its potential relationship to more permanently inhabited Zarzian sites such as PG.

To date there is no evidence for the existence in the NW Zagros during the late Pleniglacial of the diversity of habitation practices characterising contemporaneous Levantine early and middle Epipalaeolithic entities. It is possible that the distinctive topography and micro ecologies of the piedmont zone, consisting of limestone ridges separated by narrow valleys hosting resource-rich if spatially restricted mosaics of open woodland, grassland and riparian habitats, were not conducive to the creation of large multi-seasonal aggregation locales such as Kharaneh IV and Jilat 6 in the expansive wetlands of the Azraq basin [160, 161] or year-round occupied settlements with structures such as Ohalo II on the shore of the Sea of Galilee [162]. Furthermore, although long-distance exchange networks and the use of personal ornaments and ochre are attested at PG, at the same time there is no evidence for other types of social behaviours found in some Levantine sites of this period including, for example, burials [163, 164]. The differences observed in the nature, diversity and scales of settlement patterns and material culture practices become even more pronounced once the chronologically later Zarzian phases known from PG and claimed at other sites, are compared to the Levantine late Epipalaeolithic (Natufian) entities. The climatic amelioration that marked the start of the Lateglacial does not appear to have been accompanied by coeval shifts in Zarzian lithic technologies, habitation patterns and subsistence practices. The new PG chronology suggests that the prevailing Epipalaeolithic habitation pattern in the NW Zagros (centred on moderately long, generalised occupations of small caves and rock-shelters alongside ephemeral, task-oriented occupations of camps) remained an enduring characteristic of the Zarzian horizon throughout the late Pleniglacial and the Lateglacial. In turn, the new PG archaeobiological, material culture and radiometric data also disprove suggestions that this pattern might be explained by resource-poor landscapes, adverse climatic conditions, and/or geographic and cultural isolation.

The only sites in the NW Zagros that have provided some indications of cultural shifts potentially associated with a ‘late Zarzian’ facies are Shanidar B and Zawi Chemi Shanidar B. Their precise position in the diachronic evolution of the Zarzian horizon remains unknown due to the continuing lack of reliable radiocarbon determinations, which in the case of Shanidar B is further compounded by unresolved stratigraphic ambiguities. The Zawi Chemi B assemblage is particularly interesting, due to the discovery of a twice rebuilt circular structure found associated with a concentration of caprine skulls and raptor wing bones, its chipped stone assemblage containing a significant component of geometric microliths dominated by lunates, and its diverse repertoire of ground stone items (axes, querns, mortars, pestles, etc.) and bone tools and beads [6]. The technological affinities of the Zawi Chemi B microlithic component with the lithic sample retrieved from the disturbed upper part of Howe’s trench have long been treated in the literature as indicative of a ‘final Zarzian’ phase, possibly dated to the terminal Pleistocene and/or the beginning of the Holocene [2, 26]. A different approach to the question of the ‘end’ of the Epipalaeolithic horizon in the NW Zagros and what succeeded it on the putative path to ‘Neolithisation’ entails focusing on reconstructing the ways in which Zarzian habitation patterns and cultural practices that we now know had persisted on the piedmont zone for millennia since the LGM, were transformed coevally with widely shared conceptions of community identity and coherence. Across SW Asia, the rapid decadal-scale climate improvement that marked the start of the Holocene at ~11,700 cal BP and associated scalar increases in plant and animal resource levels and diversity, had a major impact in transforming people’s experiences of the lived landscape [165, 166]. The story of how these changes unfolded in the piedmont zone, and the types of reconfigurations they triggered in human lifeways that had endured at quasi geological timescales, remain open questions in search of data-informed answers in the prehistory of the NW Zagros.

## Supporting information

S1 File The micromorphology of Palegawra Phase 1 upper and Phase 2. (PDF)

S2 File Biometrical analysis of Palegawra equids. (PDF)

S3 File First results of phytolith analysis from Palegawra. (PDF)

S1 Table Palegawra 2016–2017 seasons flotation sample register. (XLSX)

S2 Table List of Palegawra micromorphological blocks fabric attributes and components. (XLSX)

S3 Table List of Palegawra bone samples tested for collagen preservation. (XLSX)

S4 Table Palegawra EFEC project radiocarbon sample attributes. (XLSX)

S5 Table Palegawra Area A sequence Bayesian age model. (XLSX)

S6 Table Palegawra chipped stone items grouped by context, phase and lithic category. (XLSX)

S7 Table Palegawra equid measurements. (XLSX)

S8 Table Counts and relative abundances of diagnostic, undiagnostic and indeterminate bones (hare size and above) grouped by context and phase. (XLSX)

S9 Table Average fragment size and frequency of burning of undiagnostic long bone fragments grouped by context, phase and size category. (XLSX)

S10 Table Summary of ungulate taxa fusion data. (XLSX)

S11 Table Crown heights of equid cheek teeth and the estimated age range they correspond to (after Levine 1982: Appendix table IIIa). (XLSX)

S12 Table Skeletal element counts of the mammalian diagnostic bone assemblage grouped by size category. (XLSX)

S13 Table Skeletal element counts of the undiagnostic bone assemblage grouped by context, phase and size category. (XLSX)

S14 Table Habitat fidelity scores for mammalian taxa. (XLSX)

S15 Table Laboratory processing protocol for phytolith extraction. (XLSX)

S16 Table Palegawra Area A (Phases 1–3) charred plant macro-remains. (A) Per sampled context (n = 52) charred macro-remain counts and density ratios; (B) Per phase charred macro-remain density ratios.
(XLSX)

S1 Fig Topographic map showing location of sites in the Sulaymaniyah-Kirkuk area (map by E Asouti). Map created using QGIS 3.10.7 (free and open source) with data from NASA Shuttle Radar Topography Mission (SRTM) (2013). Shuttle Radar Topography Mission (SRTM) Global. Distributed by OpenTopography. https://doi.org/10.5069/G9445JDF (Accessed: 2020-06-29).
(TIF)

S2 Fig Topographic map showing location of sites in the Sulaymaniyah area (map by E Asouti). Map created using QGIS 3.10.7 (free and open source) with data from NASA Shuttle Radar Topography Mission (SRTM) (2013). Shuttle Radar Topography Mission (SRTM) Global. Distributed by OpenTopography. https://doi.org/10.5069/G9445JDF (Accessed: 2020-06-29).
(TIF)

S3 Fig Topographic map showing the location of Palegawra adjacent to the Bazian valley (map by E Asouti). Map created using QGIS 3.10.7 (free and open source) with data from NASA Shuttle Radar Topography Mission (SRTM) (2013). Shuttle Radar Topography Mission (SRTM) Global. Distributed by OpenTopography. https://doi.org/10.5069/G9445JDF (Accessed: 2020-06-29).
(TIFF)

S4 Fig Phase 1 deposits excavated in the western half of Trench A in 2017 (photos by E Asouti). (A-C): Phase 1 upper; (D-G) Phase 1 lower; (H) basal fill (Scale: 0.50m except for H: scale 0.30m).
(TIF)

S5 Fig Phase 1 deposits excavated in the eastern half of Trench A in 2017 (photos by E Asouti). (A-C): Phase 1 lower; (D) basal fill (A-B scale: 0.50m; C-D scale: 0.30m).
(TIF)

S6 Fig Palegawra Iraq-Jarmo project unmodelled calibrated dates BP. (TIF)

S7 Fig Palegawra EFEC project modelled calibrated dates BP. (TIF)

S8 Fig Microlithic tools from Phase 1 contexts (photos by E Asouti). (A) backed and obliquely truncated plus arched truncation (Small Find no.107/context ACS); (B) backed and obliquely truncated (SF126/ACT); (C) backed bladelet (SF180/ADG) (images displayed in S8–S13 Figs were captured using a Keyence VX7100 4K ultra-high accuracy digital microscope at x50 magnification).
(TIF)

S9 Fig Microlithic tools from Phase 2 contexts (photos by E Asouti). (A) backed piercer (SF41/ABY); (B) backed and obliquely blunted (SF29/ABT).
(TIF)

S10 Fig Microlithic shouldered piece or piercer (photo by E Asouti). (Phase 2-SF51/ACF).
(TIF)

S11 Fig Dorsal (left) and ventral (right) surfaces of a scalene bladelet (Photos by E Asouti). (Phase 2-SF41/ABY).
(TIF)

S12 Fig Microlithic tools from Phase 2 contexts (photos by E Asouti). (A) arch-backed and truncated bladelet (SF31/ABT); (B) backed bladelet fragment (SF33/ABV).
(TIF)

S13 Fig Tools from Phase 3 contexts (photos by E Asouti). (A) piercer (ABY/Flotation sample register no. 16011); (B) backed and obliquely truncated (ABG/Flot. sample reg. no. 17009).
(TIF)

S14 Fig Transverse planes of *Amygdalus* sp. wood charcoals showing curved rings and thick rays (photos by E Asouti). (A-B) ACZ/Flot sample register no. 17042.
(TIF)

S15 Fig Transverse planes of *Amygdalus* sp. wood charcoals showing curved rings with irregular growth rings and thick (often discontinuous) rays (photos by E Asouti). (A) ADA/17043; (B) ACX/17039.
(TIF)

S16 Fig Transverse planes of *Amygdalus* sp. wood charcoals showing thick rays (photos by E Asouti). (A) ADD/17045; (B) ACV/17037.
(TIF)
