# Supplementary material for: The Zagros Epipalaeolithic revisited: New excavations and 14C dates from Palegawra cave in Iraqi Kurdistan
Source: PLoS One. 2020 Sep 21;15(9):e0239564. doi: 10.1371/journal.pone.0239564 (PMC7505476; doi:10.1371/journal.pone.0239564)
Supplement: S1 File — (PDF) [file pone.0239564.s001.pdf]

S1 Supporting Information File. The micromorphology of Palegawra Phase 1 upper and Phase 2

Methods

4 micromorphological block (MB) samples were cut from select sections in Trench A in 2017, in order to explore further site formation processes, human activities, and the taphonomic alterations of the archaeological deposits. Sampling in the field was constrained primarily by the density of rock debris, particularly within Phase 1 and the basal deposits excavated in Area A. We prioritised for analysis the Phases 1-2 boundary, Phase 1 upper and Phase 2, focusing on areas in which distinctive concreted yellowish clay surfaces, and clay nodules and patches were noted during excavation. MB1 and MB2 were cut from a temporary section running N-S along what became the centre of Area A in 2017, when the trench was expanded westwards towards the interior of the cave (Fig. S1.1). Both overlap the Phases 1-2 boundary. MB3 (Phase 1 upper) and MB4 (Phase 2) were cut from the western section of Area A (Fig. S1.2).

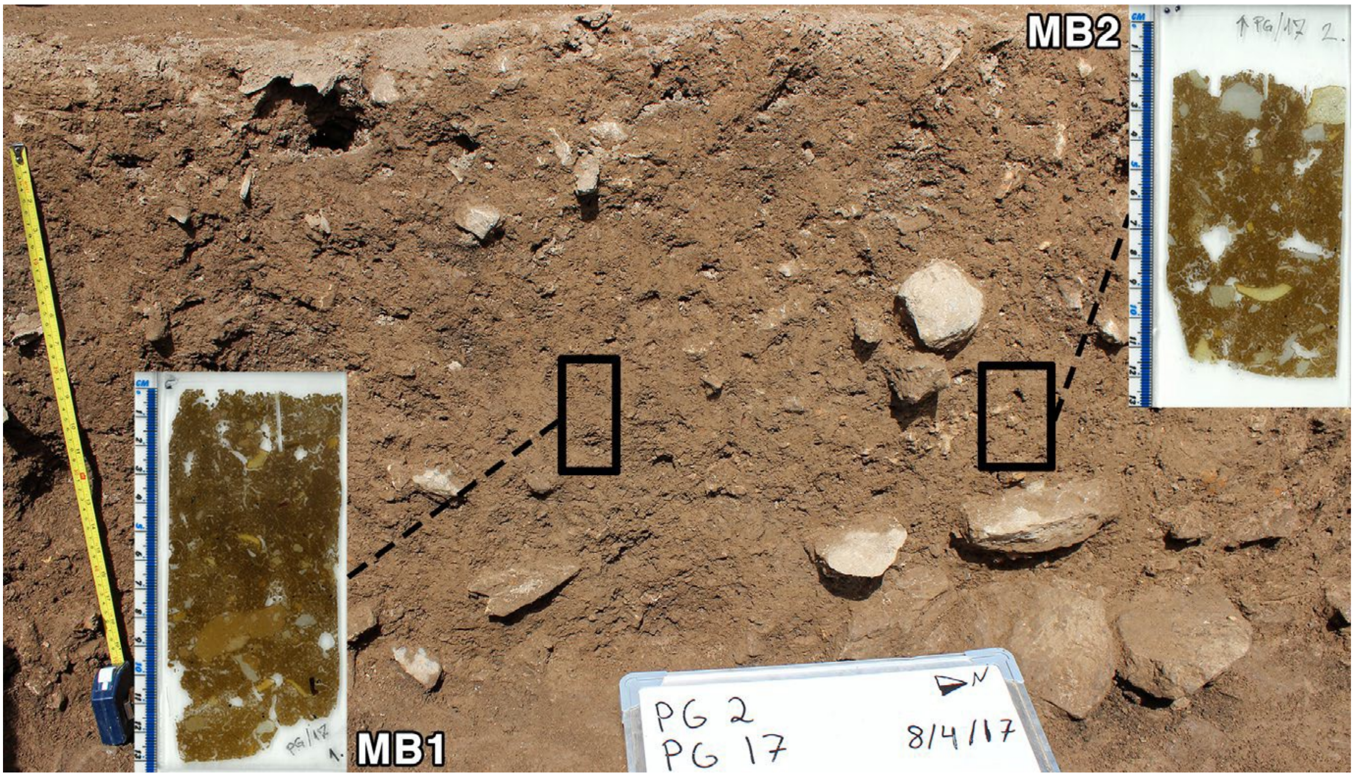

Fig. S1.1. Location of MB1 and MB2 on the temporary section running N-S along the centre of Trench A before it was expanded to the west in 2017 (scale: 0.50m) (Photos by E Asouti and A García-Suárez).

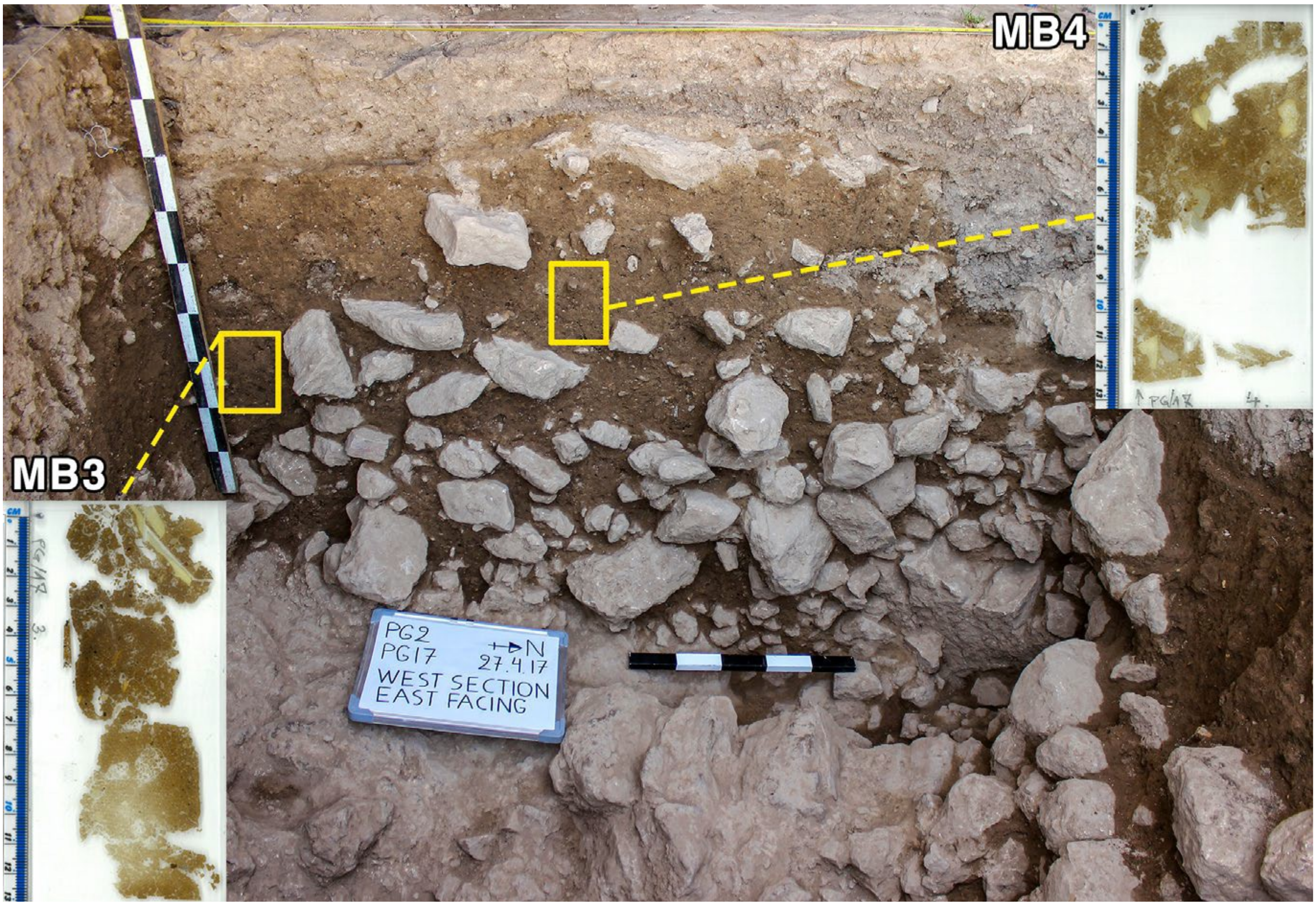

Fig. S1.2. Location of MB3 and MB4 on the western section of Trench A (Photos by E Asouti and A García-Suárez).

The sediment blocks were processed at the McBurney Laboratory for Geoarchaeology of the University of Cambridge. Following oven-drying at 40°C, they were impregnated with polyester crystic resin and dried for several weeks. The hardened blocks were then cut and trimmed with an abrasive saw into approximately 1cm thick slices, which were subjected to surface grinding in order to produce flat uniform surfaces suitable for bonding to a glass slide. After mounting them to large-format microscopy slides (~14 x 7cm) they were ground and polished to the standard thickness of 30μm and cover-slipped. The resulting micromorphological thin sections were analysed using a Leica DMLSP microscope (magnifications x40-x500) under plane (PPL) and cross-polarised (XPL) light. Photomicrographs were captured using a DFC420 camera and processed with the LeicaV2.3 image analysis software. Thin sections were described using the standard terminology developed by Stoops (2003).

Results

The main attributes and components of MB1-4 are summarised in S2 Table and in Figs. S1.3-S1.6. Replicating macroscopic observations in the field during excavation, thin sections too comprise large depositional units; microscopic evidence for short, discrete depositional events is absent. Overall

the analysed units have very similar mineralogical composition: calcite is the main mineral, followed by a lesser component of quartz, with traces of chert, feldspar and iron oxides. Limestone rock fragments are ubiquitous throughout the analysed sequence, especially in the lower parts of the sampled stratigraphy.

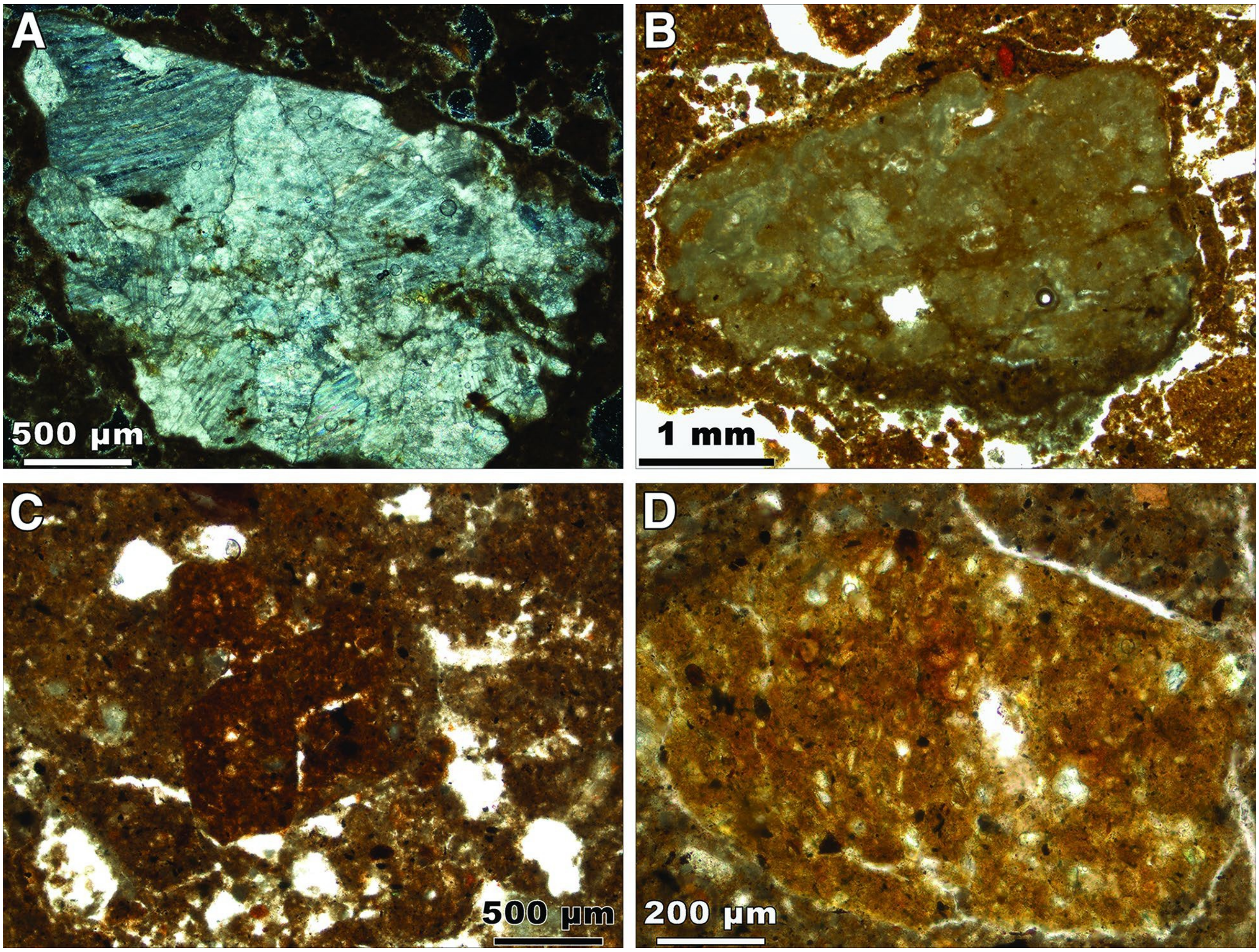

Fig. S1.3. Photomicrographs of key inorganic components observed in the PG MB samples (Photos by A García-Suárez).

(A) Subangular limestone fragment (XPL); (B) Amorphous calcareous aggregate (PPL); (C) Darkened silty clay aggregate, possibly burnt sediment fragment (PPL); (D) Sediment aggregate constituted by yellowish brown silty clay (PPL).

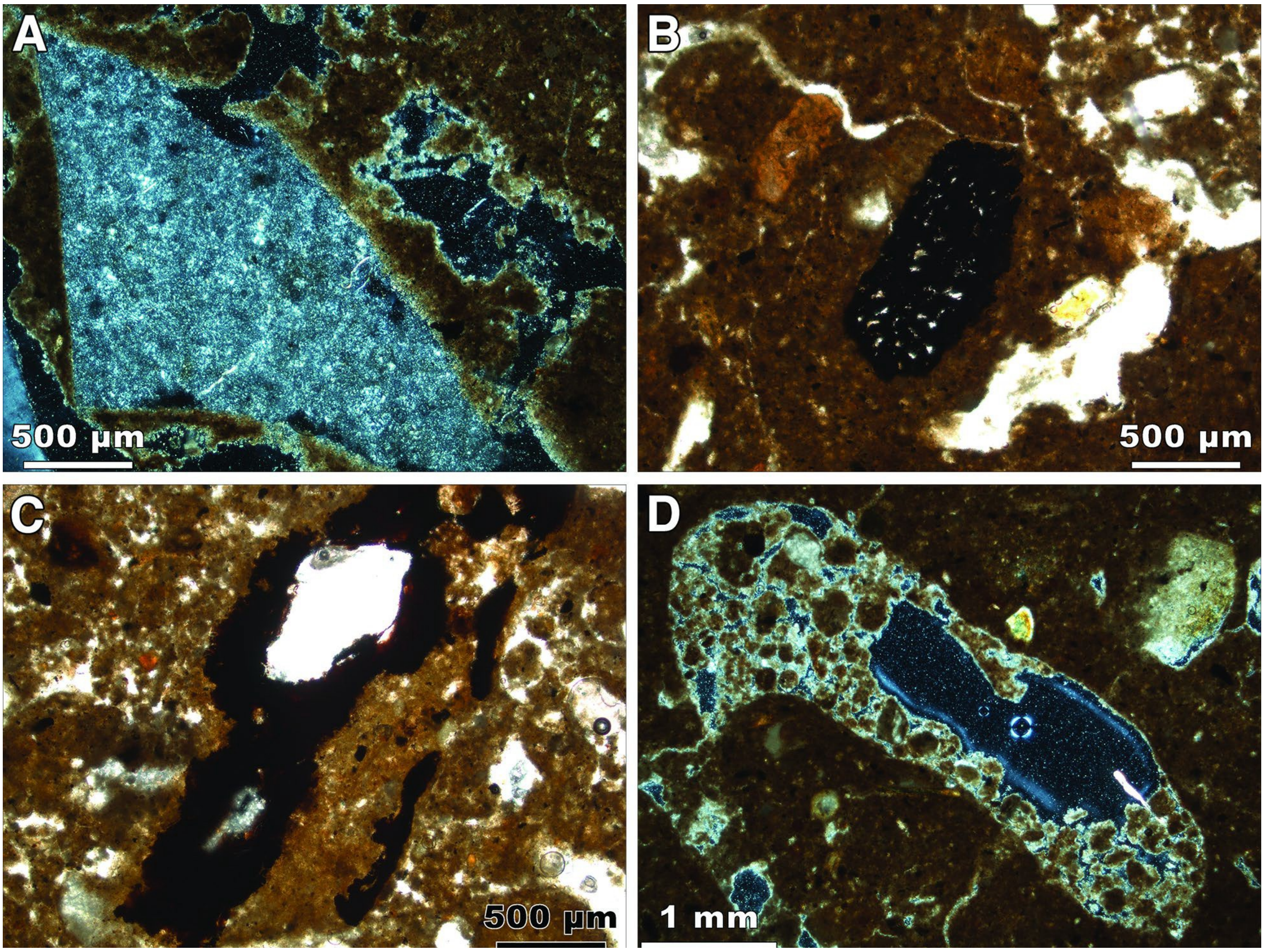

Fig. S1.4. Photomicrographs of anthropogenic components and pedogenetic features observed in the Palegawra MB samples (Photos by A García-Suárez).

(A) Angular chert fragment, possibly of lithic artefact (XPL); (B) Charcoal fragment (PPL); (C) Strongly impregnated iron hydr(oxide) hypocoating around void (PPL); (D) Channel displaying loose discontinuous infillings of sediment crumbs and thin calcitic coatings (XPL).

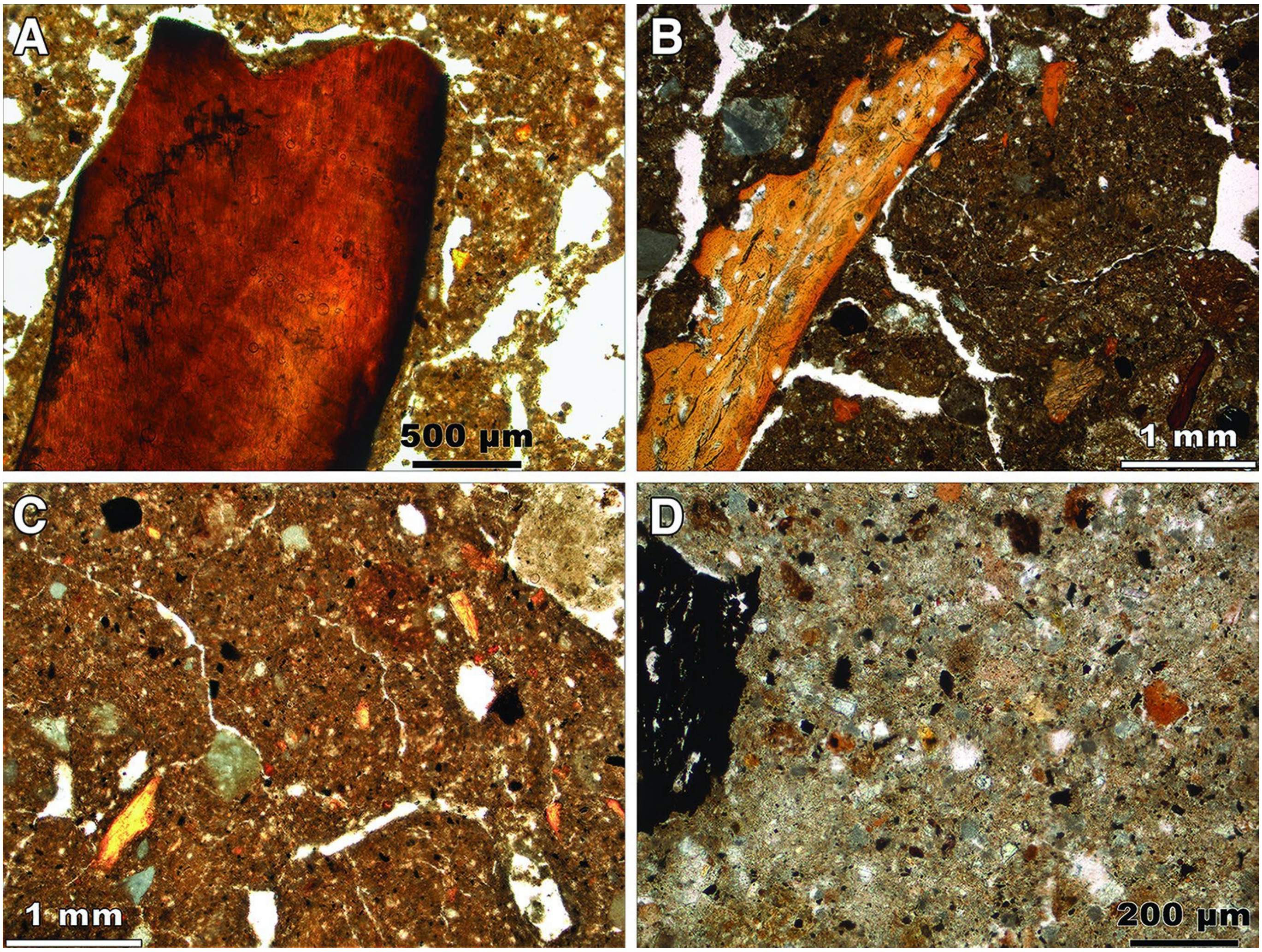

Fig. S1.5. Photomicrographs of bone fragments, micro-charcoal and deposit fabrics observed in the Palegawra MB samples (Photos by A García-Suárez).

(A) Burnt bone with darkened edges (PPL); (B) Burnt bone displaying structural alterations in the form of microfractures (PPL); (C) Phase 1 (Upper) brown clayish matrix with vugh voids, calcareous aggregates, charred plants and bone remains (PPL); (D) Phase 2 brownish grey groundmass with multiple fragments of charred plants (PPL).

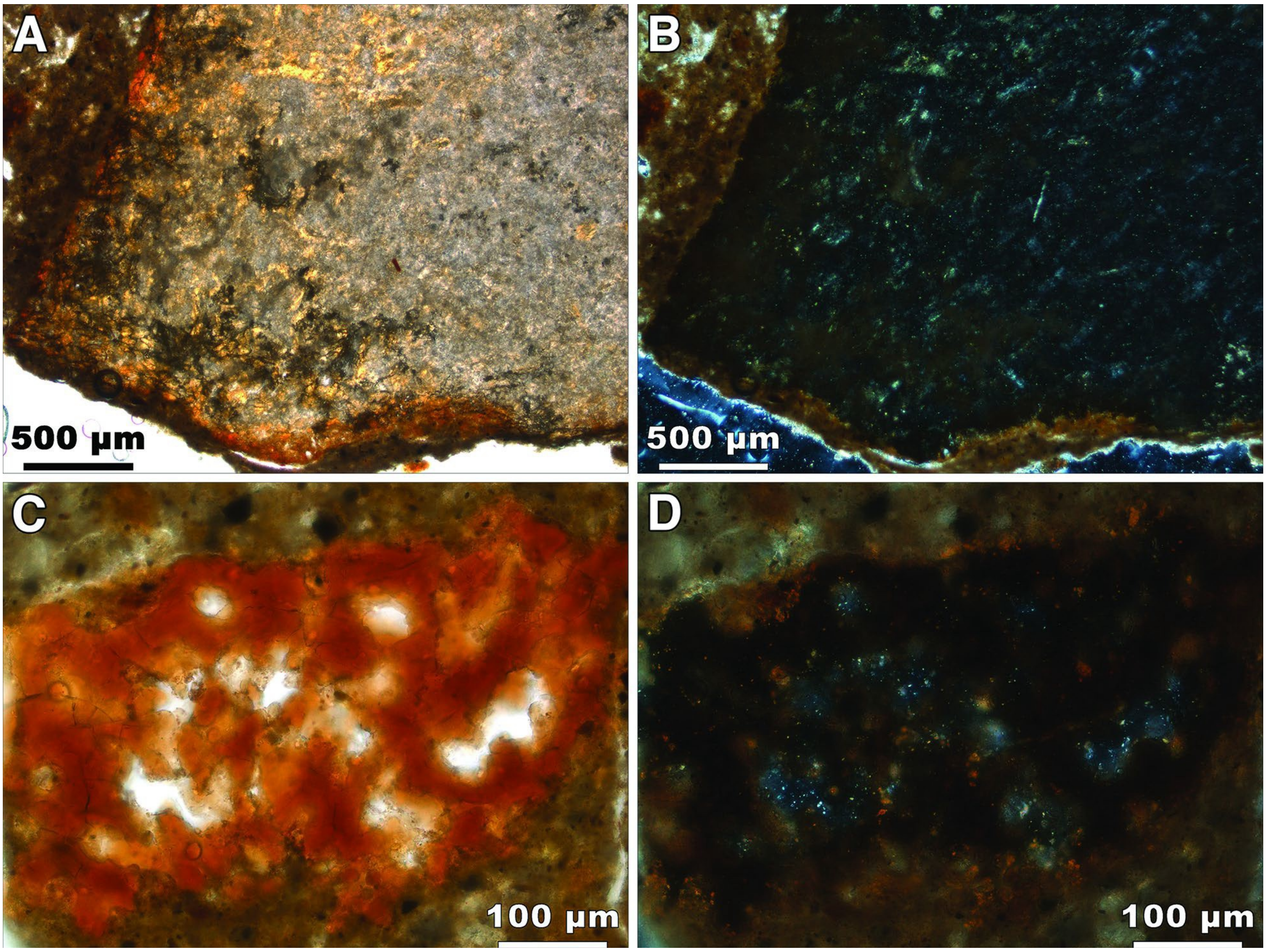

Fig. S1.6. Photomicrographs of bone fragments observed in the Palegawra MB samples (Photos by A García-Suárez).

(A-B) altered bone fragment displaying a thin iron coating (A: PPL, B: XPL); (C) Degraded bone: orange-brown massive homogeneous material pointing to chemical/mineralogical changes, PPL; (D) same as (C) under XPL, showing the lack of birefringence of the altered and darkened bone mass.

Phase 1 upper and Phases 1-2 boundary: MB1-3

The fabric of these deposits is formed by brown silty clay with a small component of moderately-sorted sands and calcareous sediments derived from the weathering of the cave wall (Fig. S1.5C). Subangular limestone clasts (500µm-1.8cm) are abundant (~10%) arranged mostly in vertical/oblique orientation (Fig. S1.3A). Some contain embedded bivalve shells. Occasionally they display iron staining and chemical degradation around the edges, while some also show thin coatings of aligned clay particles. Voids constitute approximately 20-25% of the sampled deposits. Oval channels (500µm-2cm) are the most frequent type followed by irregular-shaped vughs and, to a lesser extent, vertically oriented narrow highly accommodated planes. They have smooth to slightly irregular boundaries and are occasionally infilled with calcareous sediments. Pedogenic carbonates such as calcitic hypocoatings around channels are less common (Fig. S1.4D). Rounded soft calcareous aggregates (~20-350µm) occasionally displaying internal voids comprise ~5-10% of the sampled deposits (Fig. S1.3B). Yellowish brown silty clay aggregates (~10-30%) are larger (500µm-4cm) (Fig. S1.3D) and appear to have a higher calcitic content. They likely correspond to the light clay nodules encountered in the field, being particularly abundant in the boundary between Phase 2 and Phase 1 (as observed in MB1). Embedded in them are randomly oriented and distributed anthropogenic materials (microcharcoal and burnt bone). Darkened reddish-brown silty clay aggregates occur in very low proportions (~ 1%). These may represent re-deposited burnt sediment fragments (Fig. S1.3C). Reddish brown to very dark brown rounded iron (hydr)oxide nodules (350-800µm) comprise ~5% of the sampled deposits. They are moderately strongly impregnated and display a continuous fabric(identical to that of the groundmass of the sampled units) and distinct, clear boundaries. These nodules are randomly oriented and distributed, occasionally occurring as void hypocoatings (Fig S1.4C) and were formed *in situ* by post-depositional pedogenetic processes.

Fire-related activities are indicated by the presence of randomly distributed burnt sub-angular to sub-rounded bones and dentine (~10-15% of the sampled deposit). Bone fragments (200µm-3.6 cm) display a vertical to subvertical referred orientation and are highly variable with regard to size, burning degree and preservation. Most appear charred to various degrees (Fig. S1.5A-B). ~1% of the bone assemblage is calcined, while a few bones display iron (hydr)oxide impregnative features and coatings (Fig.S1.6A-B). Some of the smallest fragments appear very degraded and, in extreme cases, display a melted appearance following the degradation of their hydroxyapatite microstructure (Fig. S1.6C-D). Bleaching and micro-fissures are also evidenced (Fig. S1.5B) although evidence of *in situ* bone breakage is absent. Micro-charcoal (~5%) is ubiquitous (Fig. S1.4B) being randomly if evenly distributed. Charcoal particles vary in size (5µm-2mm) with most occurring as micro-residues of <20µm. Very little lithic debris has been identified in the sampled deposits, comprising randomly oriented and distributed angular fragments of micro-chert (up to 2mm) (Fig. S1.4A).

Phase 2: MB4

This deposit is slightly more fine-grained containing fewer limestone clasts and clay aggregates (Fig. S1.5D). Its groundmass consists of brownish grey silty clay with a small component of sub-angular/ sub-rounded sands and ~7% limestone clasts. Rock fragments vary in size (500µm-2.1cm) and display a vertical/oblique orientation. A few clasts show post-depositional alterations (mainly ferric mottling and mild dissolution). The crystallitic birefringence fabric points to the higher calcitic content of this deposit. ~30% of it comprises void space, with channels being the most abundant type, indicating a significant degree of mechanical alteration of this sequence caused by biological action. Calcitic hypocoatings and crumby infillings are a common feature of these voids. Star-shaped vughs and plane voids also occur, the latter with highly accommodated edges (narrowly separated) and a vertical orientation. Small calcareous aggregates (~20-250µm) are slightly more abundant in this unit (up to 15%). Yellowish brown silty clay aggregates (300µm-3.8mm) are also present (~5%). These two types of aggregates have rounded forms and are randomly if evenly distributed throughout the sampled unit. Redoximorphic features (~2%) occur mainly in the form of typic iron (hydr)oxide nodules (250-600µm). These impregnative pedofeatures are rounded to sub-rounded, moderately to strongly impregnated, and were formed *in situ*.

Bone fragments are moderately abundant (~15%). Most display charring alterations, while few show signs of calcination. They are mainly sub-angular and appear arranged preferably in vertical and subvertical orientations. Post-depositional alterations include mostly micro-fissures and diagenetic chemical degradation. Overall, bone fragments vary widely in size, burning effects and preservation conditions. Micro-charcoal particles (~5%) are randomly oriented and distributed. They display a generally sub- rounded shape and vary in size (10-600µm) although most are <100µm in length.

The microstratigraphy of Phase 1 upper and Phase 2

The silty clayey deposits of Phases 1 upper and 2 are typical of low-energy runoff commonly observed in cave mouths and rock shelters (Goldberg and Macphail 2006, Mallol and Goldberg 2017). Runoff facies frequently show horizontal or sub-horizontal bedding with multiple layers of variable thickness, depending on the energy of deposition (Polo Díaz et al. 2014). The small proportion of moderately sorted sub-angular sands might also be indicative of a modest incidence of aeolian sedimentation. The sediments of the sampled sequence lack discernible anthropic alterations, with the exception of the few darkened aggregates which may represent re-deposited burnt sediment fragments. Post-depositional biological activity contributed to the obliteration of the original sediment bedding, resulting in the formation of the massive silty clay deposits observed during excavation and under the microscope.

Two main types of sediment aggregates have been microscopically identified: calcitic and clayish. The soft calcareous aggregates likely originate from the dissolution of the cave wall and the re-precipitation of its soluble calcareous content into the sediments. This is a process commonly observed in caves and rock shelters, being strongly dependent on water content, temperature, pH and ion concentration. The weathering of the cave wall is also evident in the amount of roofspall found in the analysed samples, which becomes increasingly abundant with depth. Poorly sorted limestone clasts of variable sizes are randomly distributed throughout the sampled units. They occasionally display thin coatings of aligned clayish sediment along their outer perimeters, which are indicative of rolling and transportation. Roofspall is generally well-preserved, although a few clasts show the effects of diagenetic transformations mainly as bio-reworked aggregates, dissolution (also documented in bone) and re-precipitation of carbonated solutions. Most calcareous aggregates are very small (<200µm) while the larger fragments display a porous, spongy microstructure reminiscent of tufa which appears to be re-deposited as rounded fragments.

Yellowish silty clay aggregates were observed in all analysed samples. Although no distinctive layer of clayish aggregates was identified in thin-section, MB1 (comprising the boundary between Phase 1 and Phase 2) clearly displays large (>1cm in length) clay aggregates with sharp to diffuse boundaries in sub-horizontal parallel orientation. Extensive concreted surfaces of yellowish clay, marking the boundary between Phases 1 and 2, were observed macroscopically during excavation noting that they may have resulted from the evaporation of pooled water on transient surfaces and were not necessarily associated with occupation activities. In thin section, the composition of these yellowish silty clay aggregates is relatively consistent including a moderate calcareous component. They also vary widely in size and shape, possibly due to fragmentation and translocation caused by percolating water and further post-depositional biological activity. Although they contained some unsorted charred fragments of angular bone and micro-charcoal, these were found embedded in their groundmass, similar to macroscopic observations in the field for the inclusion of such materials in the matrix of these deposits. Evidence of living surfaces such as smooth, compacted upper boundaries has not been detected in thin section. However, clay patches with compacted smooth surfaces were dug in other parts of the Area A sequence.

Iron (hydr)oxide nodules are ubiquitous, comprising approximately 5% of the sampled deposits. These redoximorphic features are associated with wetness causing the reduction, translocation and oxidation of iron compounds in sediments after water saturation, and may be linked to runoff events (Lindbo et al. 2010). The coats of iron oxides observed on some bone fragments resulted from the decomposition of organic matter through microbial activity.

Channel voids (~25% of the sampled deposits, with a higher proportion found in Phase 2) represent the product of biological activity caused by exposure to environmental conditions after accumulation. The preferred vertical and subvertical orientation some of these voids points to root

action, although some faunal activity is also evident in the crumby and slightly crescentic passage features observed in the analysed samples. The occasional presence of moderately to well-developed calcitic hypocoatings around channels may also indicate root action. Some of the largest channels display infillings of lighter clayish sediments, possibly derived from upper deposits washed down by percolating water.

Microscopic bone comprises unsorted fragments displaying multiple degrees of burning and a slightly dominant vertical orientation indicating secondary deposition. Some appear intensively bleached, with a characteristic whitish colouration and increased porosity due to the mechanical erosion of bone tissue by water (Hedges and Millard 1995). Others are severely altered, appearing as masses of orange-brown non-birefringent material caused by the chemical deterioration of bone structure (Huisman et al. 2017). The same process may also be responsible for the micro-fissures observed in several bone fragments, possibly linked to rapid collagen loss. Overall, the evidence reflects highly variable preservation conditions, pointing to diverse depositional pathways for bone micro-remains and/or the impacts of localised fluctuations in pH and moisture content. Significantly, bone fragments are much larger than microcharcoal particles, which (with few exceptions) are <100µm in size and are found dispersed across the sampled units. The high degree of charcoal fragmentation could be the result of human actions such as trampling, and/or natural processes such as sediment runoff and gravital redeposition.

The microscopic analysis of MB sediment samples from Phase 1 upper and Phase 2 did not reveal evidence for discernible *in situ* occupation surfaces and/or activity areas. Unambiguous trampling indicators (compacted sediment, fissuring, micro-aggregation and *in situ* fracturing of bone and charcoal inclusions (Courty et al. 1989, Gé et al. 1993, Rentzel et al. 2017) were also absent. On the whole, the micromorphological evidence suggests that this part of the Area A sequence represents primarily reworked runoff deposits including translocated anthropic residues derived from activities that took place in the inner cave chamber, which were further transformed by various post-depositional processes.

References

Courty MA, Goldberg P, Macphail RI (1989) Soils and micromorphology in archaeology. Cambridge: CUP.

Gé T, Courty MA, Matthews W, Wattez J (1993) Sedimentary formation processes of occupation deposits. In: Goldberg P, Nash DT, Petraglia MD, editors. Formation processes in archaeological context, pp.149–164. Madison: Prehistory Press.

Goldberg P, Macphail RI (2006) Practical and theoretical geoarchaeology. Oxford: Blackwell.

Hedges RE, Millard AR (1995) Bones and groundwater: towards the modelling of diagenetic processes. J. Archaeol. Sci. 22: 155–164.

Huisman H, Ismail-Meyer K, Sageidet BM, Joosten I (2017) Micromorphological indicators for

degradation processes in archaeological bone from temperate European wetland sites. J. Archaeol. Sci. 85: 13–29.

Lindbo DL, Stolt MH, Verpraskas MJ (2010) Redoximorphic features. In: Stoops G, Marcelino V, Mees F, editors. Interpretation of micromorphological features of soils and regoliths, pp.129–148. Amsterdam: Elsevier.

Mallol C, Goldberg P (2017). Cave and rock shelter sediments. In: Nicosia S, Stoops G, editors. Archaeological soil and sediment micromorphology, pp.359–381. Oxford: Wiley.

Polo Díaz A, Martínez-Moreno J, Benito-Calvo A, Mora R (2014) Prehistoric herding facilities: site formation processes and archaeological dynamics in Cova Gran de Santa Linya (southeastern Prepyrenees, Iberia). J. Archaeol. Sci. 41: 784-800.

Rentzel P, Nicosia C, Gebhardt A, Brönnimann D, Pümpin C, Ismail-Meyer K (2017) Trampling, poaching and the effect of traffic. In: Nicosia S, Stoops G, editors. Archaeological soil and sediment micromorphology, pp.281–298. Oxford: Wiley.

Stoops G (2003) Guidelines for analysis and description of soil and regolith thin sections. Madison, Wisconsin: Soil Science Society of America.
