# Supplementary material for: The Zagros Epipalaeolithic revisited: New excavations and 14C dates from Palegawra cave in Iraqi Kurdistan
Source: PLoS One. 2020 Sep 21;15(9):e0239564. doi: 10.1371/journal.pone.0239564 (PMC7505476; doi:10.1371/journal.pone.0239564)
Supplement: S2 File — (PDF) [file pone.0239564.s002.pdf]

S2 Supporting Information File. Biometrical analysis of Palegawra equids

Comparison of Palegawra *Equus* sp. metapodia and phalanx I Bd measurements with measurements from a modern *Equus hemionus* population.

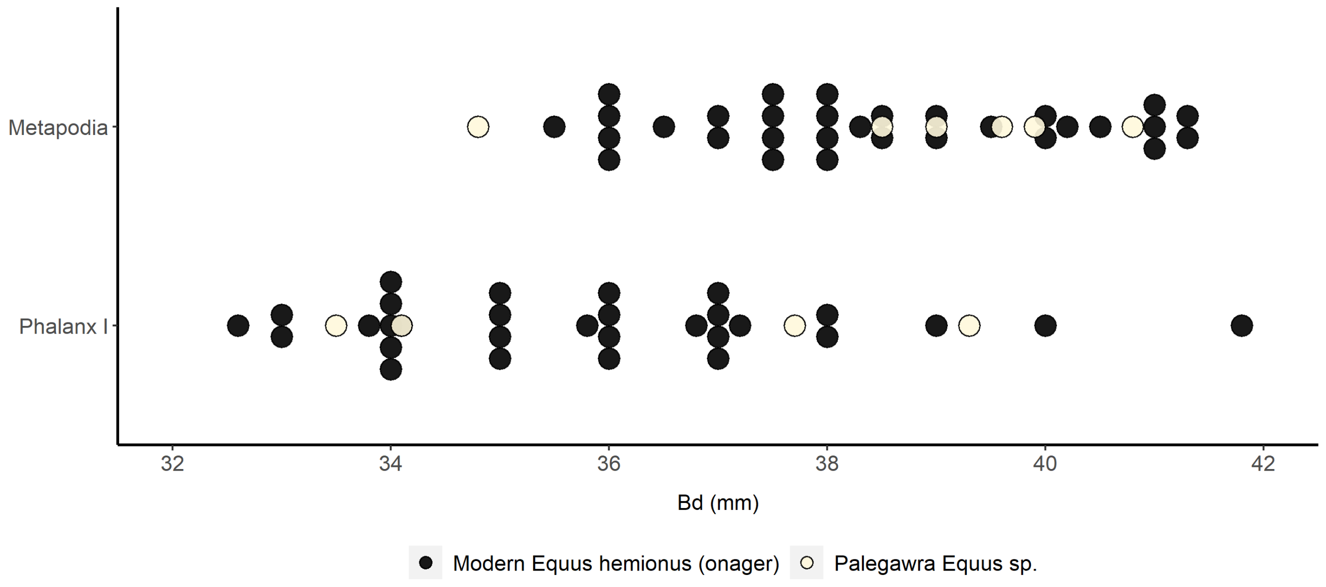

Modern *Equus hemionus* measurements come from an Iranian onager population (Eisenmann 1986a-b). Metapodia and phalanx I were selected as they represent the most abundant measured element in the Palegawra faunal assemblage that also have a good number of published comparative measurements from modern onager populations. Bd was measured following von den Driesch (1976) and was selected for this analysis as it is the most frequently recorded measurement.

Ranges of *Equus* sp. Bd measurements from Palegawra and a modern onager population (see S7 Table for raw measurements).

**Element**	**Palegawra (this paper)**		**Palegawra (Turnbull and Reed 1974)**		**Modern onager**	
	no.	range	no.	range	no.	range
Metapodia Bd (mm)	6	34.8-40.8	37	29.0-42.0	31	35.5-41.3
Phalanx 1 Bd (mm)	4	33.5-39.3	26	30.5-36.0	30	32.6-41.8
Phalanx 2 Bd (mm)	13	23.8-40.6	96	32.5-44.0	n/a	n/a

References

von den Driesch A (1976) A guide to the measurement of animal bones from archaeological sites. Cambridge MA: Peabody Museum of Archaeology and Ethnology.

Eisenmann V (1986a) Identification and discrimination of metapodials from Pleistocene and modern Equus, wild and domestic. In: Meadow R, Uerpmann HP, editors. Equids in the ancient world, pp.117–163. Wiesbaden: Reichert.

Eisenmann V (1986b) Identification and discrimination of first phalanges from Pleistocene and modern Equus, wild and domestic. In: Meadow R, Uerpmann HP, editors. Equids in the ancient world, pp. 278–333. Wiesbaden: Reichert.

Turnbull PF, Reed CA (1974) The fauna from the terminal Pleistocene of Palegawra cave, a Zarzian occupation site in northeastern Iraq. Fieldiana, Anthropol. 63(3): 81–146.
