# Supplementary material for: The Zagros Epipalaeolithic revisited: New excavations and 14C dates from Palegawra cave in Iraqi Kurdistan
Source: PLoS One. 2020 Sep 21;15(9):e0239564. doi: 10.1371/journal.pone.0239564 (PMC7505476; doi:10.1371/journal.pone.0239564)
Supplement: S3 File — (PDF) [file pone.0239564.s003.pdf]

S3 Supporting Information File. First results of phytolith analysis from Palegawra

Methods

Phytolith samples were collected in the field and processed at the Department of Archaeology and Anthropology of Bournemouth University (the laboratory processing protocol is listed in S15 Table). Initially 20 sediment samples were processed with a starting weight of 1 gram (g) the standard sample size for Southwest Asian sites. These produced few to no phytoliths. A further 27 samples were processed using a sample size of 2g, which also produced minimal results. Finally, 15 samples (10 from Area A and 5 from Area B) were processed with an initial sediment sample weight of 5g, which produced a sufficient phytolith sample for analysis.

Phytoliths were counted using a Meiji polarising light microscope (magnification x400) and the Southwest Asian phytolith reference collection held at the Department of Archaeology and Anthropology, Bournemouth University. Classification largely followed the International Code for Phytolith Nomenclature (ICPN) 2 (Neumann et al. 2019) except for the 'cone', 'bulliform', 'hair base' and 'jigsaw piece' types. Cone and hair base lack appropriate ICPN referents, while bulliform and jigsaw piece are already well-established types in the literature. Due to their different taxonomic origin we did not combine under a single 'blocky' type the dicot blocky forms and the monocot bulliforms (*contra* ICPN 2 guidelines). The 'elongate entire' type was split into 'smooth' and 'rod' types, which correspond to grasses (generally 'smooth') and Cyperaceae ('rod'). Only 10 conjoined forms were found, which were recorded separately. The number and type of conjoined cells were noted and the individual cell count of all forms comprising the conjoined form was added to the main phytolith count.

Results

In total 3,097 phytolith forms were counted (see Table S3.1). Siliceous aggregates were excluded as their size and number are highly susceptible to mechanical breakdown and vary depending on the taphonomic histories of the samples. Non-phytolith forms were also encountered including starch granules, Chrysophyceae stomatocysts (freshwater algae), sponge spicules and diatoms (Fig. S3.1). On the whole, the phytolith assemblage is characterised by low densities and overall poor preservation. % phytolith weight is low (0.005 on average for all samples) while many phytoliths were pitted, cracked and broken (Fig. S3.2A-E). There were only 10 conjoined forms in the assemblage, each comprising a few cells (Fig. S3.2G). Generally, in cave environments alkaline pH contributes to phytolith dissolution while sediment leaching can also exacerbate its taphonomic effects, which in turn pose distinctive challenges for evaluating and interpreting the relative proportions of different phytolith forms (Albert et al. 2003, Cabanes and Shahack-Gross 2015).

Table S3.1. Phytolith forms found at Palegawra listed by sampled context and main morphotype group (monocots, dicots).

CONTEXT/Phytolith sample	AAH <8>	AAF <7>	ABT <43>	AAS <23>	AAS <26>	AAS <27>	AAS <28>	AAS <29>	AAS <33>	AAS <38>
**PHASE**	**3**	**3**	**2**	**1**	**1**	**1**	**1**	**1**	**1**	**1**
% weight	0.0043	0.0121	0.002	0.0063	0.003	0.0051	0.0096	0.0085	0.0053	0.0026
**MONOCOT %**										
bulliform	11.7	8.4	0.3	6.1	3.4	2.8	5.6	4.1	4	3.7
cone	0.6				0.3	0.6	0.6			2.7
crenate	2.2	1.5		3.8	1.2		5.3		1.2	0.7
elongate dendritic	0.3	3		3.2	0.3	0.3	2.5	0.7	0.6	
elongate entire (rod)	3.8	5.7		3.2	1.2	0.3	5.6	1.4	3.7	5.8
elongate sinuate	1.3	1.2			0.3	4.5	0.6		0.6	
elongate entire (smooth)	20.2	14.1	0.6	17.6	7.6		18.6	7.1	21.4	11.2
trapezoid	3.5	0.9	0.3	6.4	0.9		1.9			0.3
hair base	0.3								0.6	
acute bulbosus	3.8	2.7		6.7	4	1.1	3.4	2.4	3.7	1.4
bulliform flabellate	20.5	3.6	1.8	5.8	18	3.1	9.3	17.2	11.5	12.2
ovate psilate		0.6				2.2				
rondel	3.2	2.1		2.2		0.6	1.6		1.9	
saddle	0.3									
**Total monocot %**	**71.6**	**43.8**	**3**	**55.1**	**37.2**	**15.4**	**55**	**32.8**	**49.4**	**38**
**DICOT %**										
rectangular psilate				1.3	0.3	0.8	0.3			
spheroid granulate				0.3		0.8				
spheroid psilate	0.6	3.6		1.6	1.2	1.4	1.9	1	0.9	3.4
jigsaw piece						0.3				
tabular irregular	23.7	38.4	87.8	35.9	60.7	69	38.2	58.1	47.2	55.9
rectangular psilate	0.3			0.3	0.6	0.8	0.3	3.4	1.9	1.4
tracheary annulate/helical		0.3		0.3						
tracheary pitted				1.3			0.6			
silica aggregate	3.2	11.1	3.9	2.2		8.4	3.1	4.1	0.6	0.7
polygonal granulate		1.5								
polygonal psilate	0.6	1.2	2.4	1.6		3.1	0.6	0.7		0.7
**Total dicot %**	**28.4**	**56.2**	**97**	**44.9**	**62.8**	**84.6**	**45**	**67.2**	**50.6**	**62**
siliceous aggregate (presence)	+	+	+	+			+	+	+	+
**non-phytolith forms (presence)**										
starch	+	+	+	+		+		+		
diatom							+			
sponge spicule	+	+	+	+	+	+	+	+	+	+
Chrysophyceae stomatocysts	+	+	+	+	+	+	+	+	+	+

(% weight reflects the total phytolith weight relative to the weight of each sediment sample; % phytolith values reflect the proportion of each type relative to total phytolith counts; siliceous aggregates and non-phytolith forms are denoted only as presence/absence data).

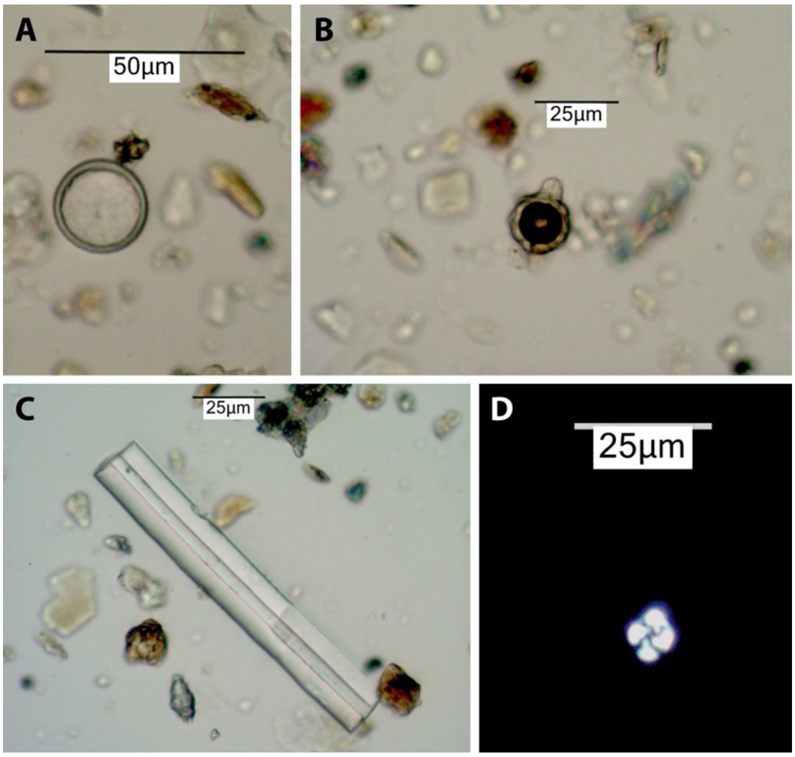

Fig. S3.1. Non-phytolith forms from Area A contexts (Photos by E Jenkins).
(A-B) Chrysophyceae stomatocysts (context AAS s.28/26); (C) sponge spicule (AAS s.38); (D) starch granule in cross polarised light (AAF).

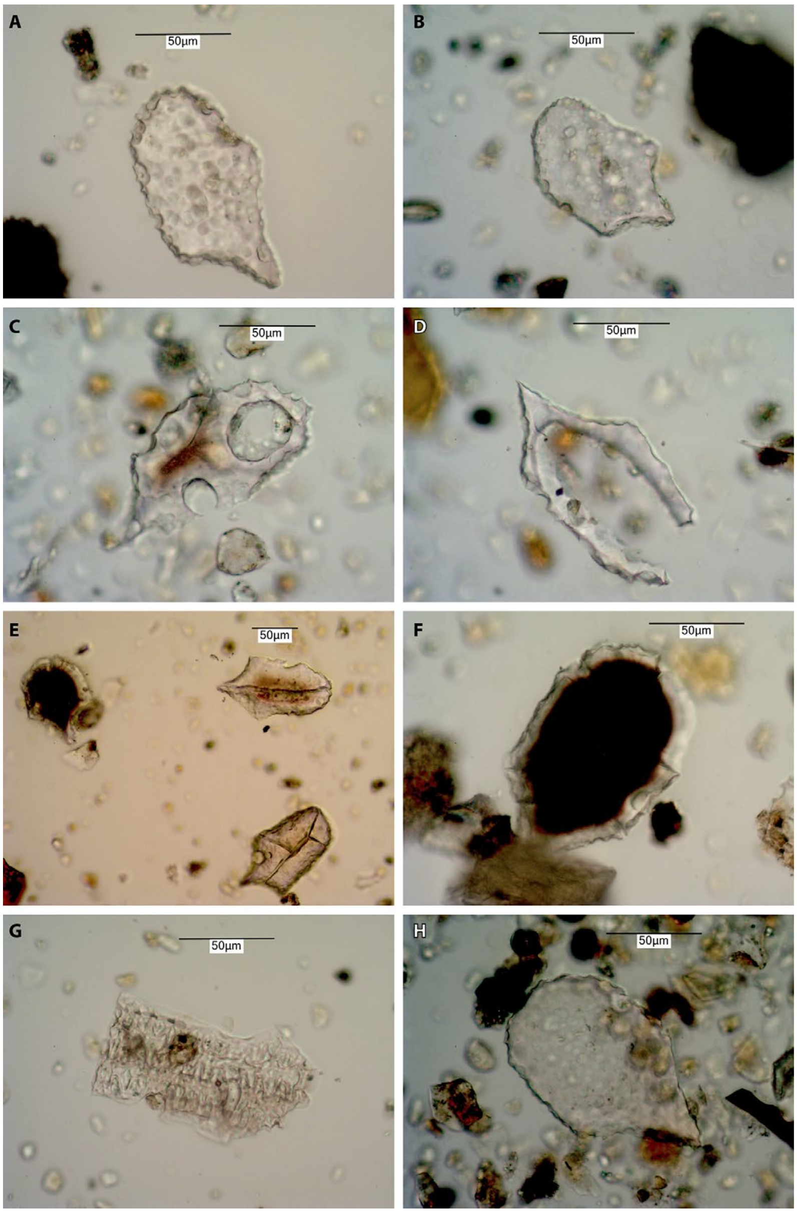

Fig. S3.2. Phytoliths from Area A contexts (Photos by E Jenkins).
(A-B) pitted bulliform flabellate (AAH/AAS s.38); (C-D) corroded bulliform flabellate (AAS s.27); (E) bulliform flabellate showing burning (left) and 2 more (right) showing cracking (AAH); (F) burnt bulliform flabellate (AAH); (G) conjoined form of cf. *Phragmites* stem (AAF); (H) bulliform flabellate cf. *Phragmites*.

Fig. S3.3A shows the relative proportions of monocot to dicot phytoliths in the analysed assemblage. AAH (Phase 3) recorded the highest concentration of monocots and ABT (Phase 2) the lowest. In ABT dicots are of the tabular irregular type, which is formed in dicot leaves rather than wood or bark (Albert et al. 2001, 2003). As Fig. S3.3B demonstrates, the majority of monocot morphotypes comprise elongate entire/sinuate phytoliths derived from grass leaves and stems rather than husks. Some experimental studies have shown that grass husk forms can be more susceptible to degradation due to chemical dissolution compared to stem and leaf forms (Cabanes and Shahack-Gross 2015). Fig. S3.3C shows the relative proportions of grass leaves bulliforms and Cyperaceae cones/elongate entires (rods). The bulliform phytoliths appear typical of *Phragmites* (see Fig S3.2A-B, H) (Wang and Lu 1992) This interpretation is further supported by the find of a conjoined reed stem form in AAF, while similar forms have been found in samples from context BAJ in Area B (analysis is ongoing) (see Fig. S3.2G). Other wetland habitat signals in the assemblage include the Chrysophyceae stomatocysts (originating from freshwater algae) and the sponge spicules encountered in all sampled contexts, alongside a diatom found in AAS (Table S3.1, Fig. S3.1).

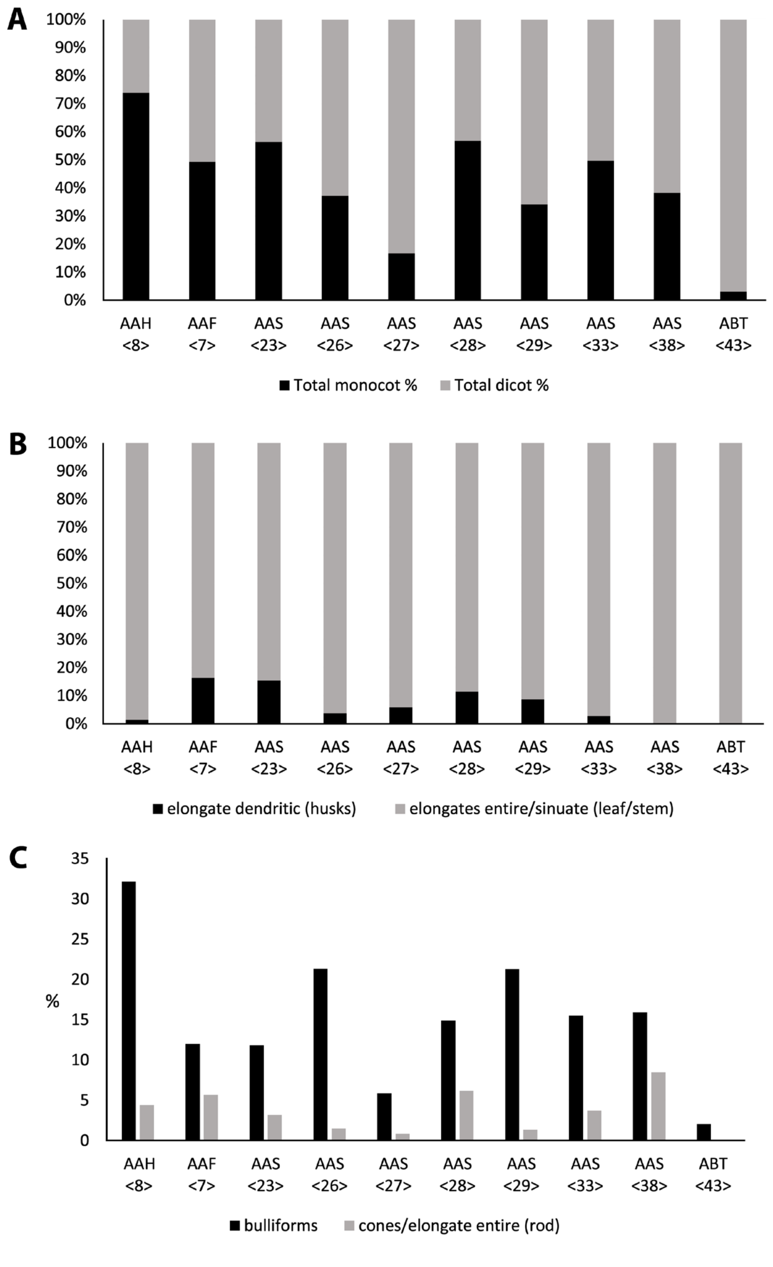

Fig. S3.3. Relative proportions (%) of phytolith forms in sampled Area A contexts. (A) monocot and dicot forms; (B) elongate dendritic (grass husks) and elongate entire (smooth)/sinuate (grass leaves/stems); (C) bulliforms (grass leaves) and cones and elongate entire (rod) (Cyperaceae).

References

Albert R, Weiner S (2001) Study of phytoliths in prehistoric ash layers using a quantitative approach. In: Meunier JD, Fabrice C, editors. Phytoliths - Applications in earth science and human history, pp.251–266. Lisse: Balkema.

Albert RM, Bar-Yosef O, Meignen L, Weiner S (2003) Quantitative phytolith study of hearths from the Natufian and Middle Palaeolithic levels of Hayonim cave (Galilee, Israel). J. Archaeol. Sci. 30: 461–480.

Cabanes D, Shahack-Gross R (2015) Understanding fossil phytolith preservation: the role of partial dissolution in paleoecology and archaeology. PLoS ONE 10(5): e0125532. doi:10.1371/journal.pone.0125532

Neumann K, Strömberg CAE, Ball T, Albert RM, Vrydaghs L, Scott Cummings L (2019) International Code for Phytolith Nomenclature (ICPN) 2.0. Ann. Bot. 124: 189–199.

Wang Y, Lu H (1992) The study of phytolith and its application. Beijing: Ocean Press.
