# Supplementary figures and images for: The Zagros Epipalaeolithic revisited: New excavations and 14C dates from Palegawra cave in Iraqi Kurdistan
Source: PLoS One. 2020 Sep 21;15(9):e0239564. doi: 10.1371/journal.pone.0239564 (PMC7505476; doi:10.1371/journal.pone.0239564)

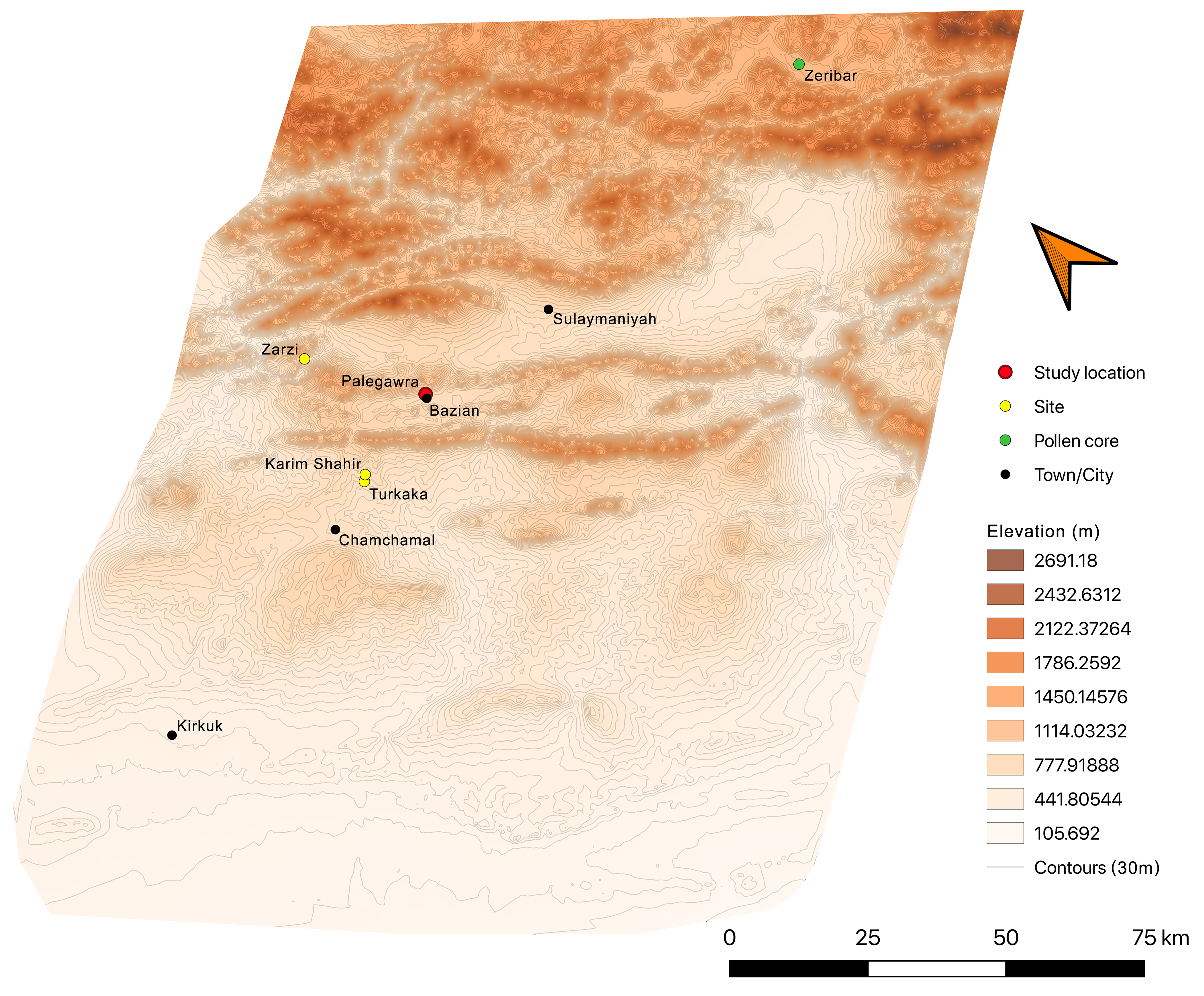

Supplement: S1 Fig — Map created using QGIS 3.10.7 (free and open source) with data from NASA Shuttle Radar Topography Mission (SRTM) (2013). Shuttle Radar Topography Mission (SRTM) Global. Distributed by OpenTopography. https://doi.org/10.5069/G9445JDF (Accessed: 2020-06-29). (TIF) [file pone.0239564.s020.tif]

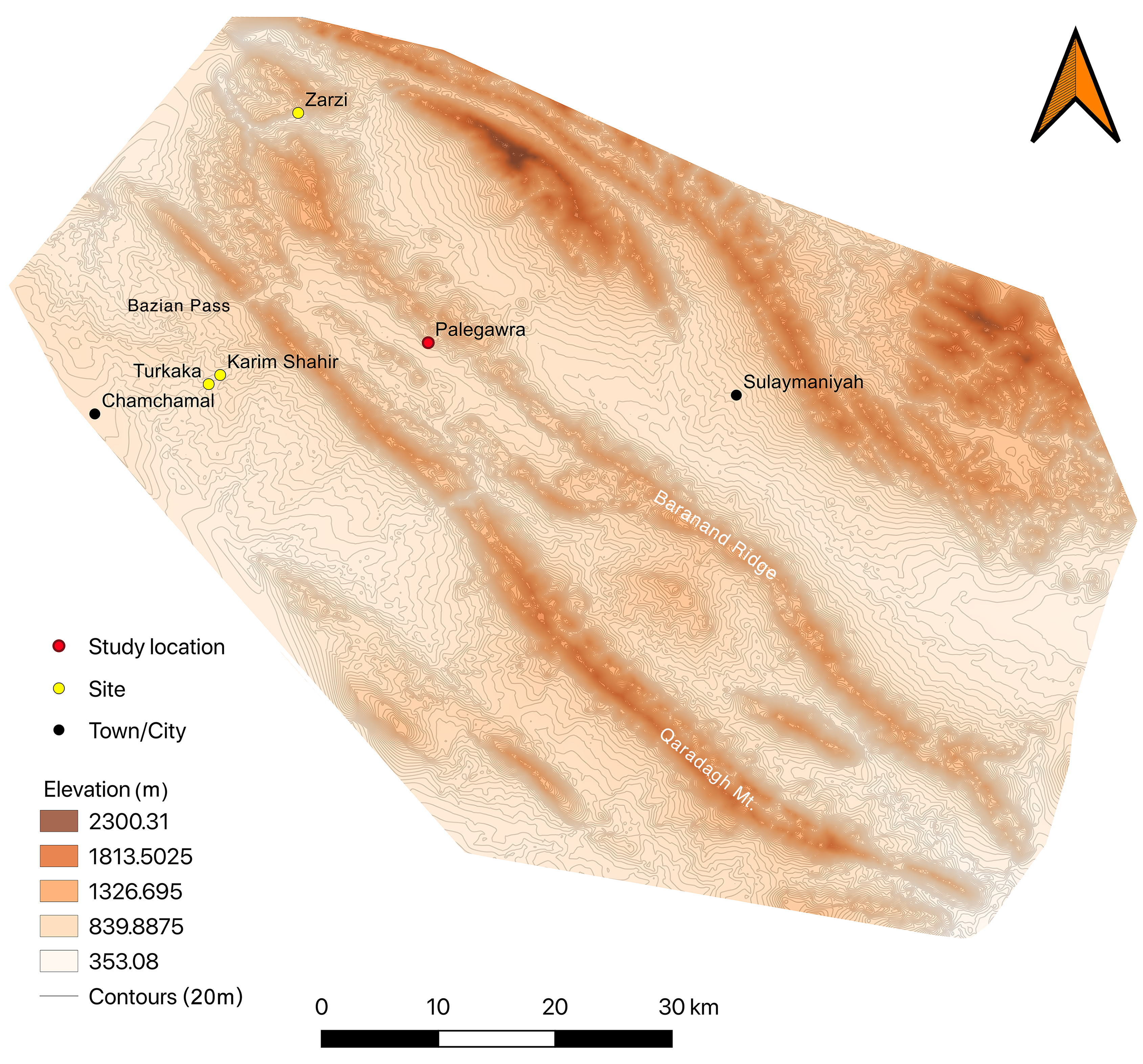

Supplement: S2 Fig — Map created using QGIS 3.10.7 (free and open source) with data from NASA Shuttle Radar Topography Mission (SRTM) (2013). Shuttle Radar Topography Mission (SRTM) Global. Distributed by OpenTopography. https://doi.org/10.5069/G9445JDF (Accessed: 2020-06-29). (TIF) [file pone.0239564.s021.tif]

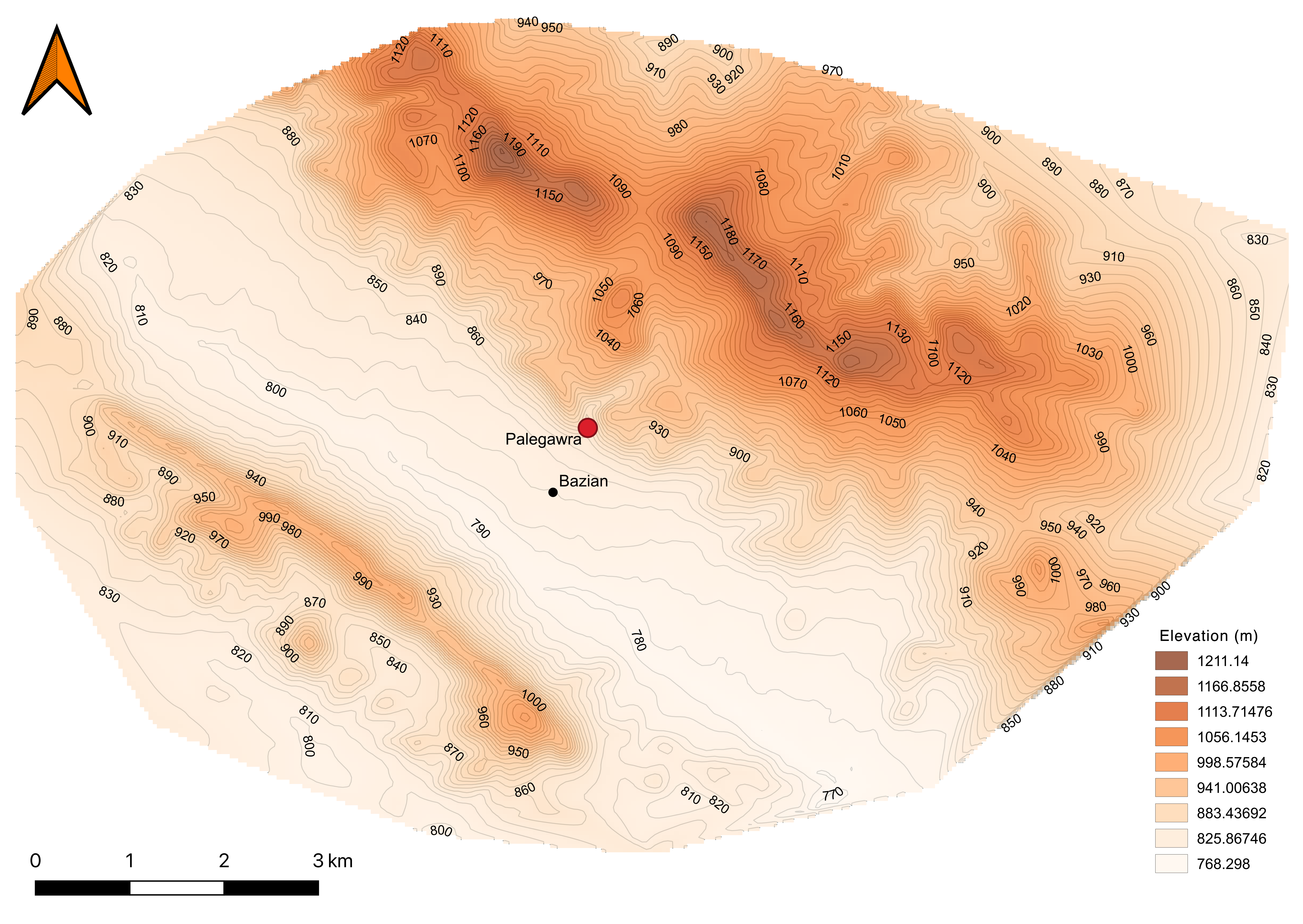

Supplement: S3 Fig — Map created using QGIS 3.10.7 (free and open source) with data from NASA Shuttle Radar Topography Mission (SRTM) (2013). Shuttle Radar Topography Mission (SRTM) Global. Distributed by OpenTopography. https://doi.org/10.5069/G9445JDF (Accessed: 2020-06-29). (TIFF) [file pone.0239564.s022.tiff]

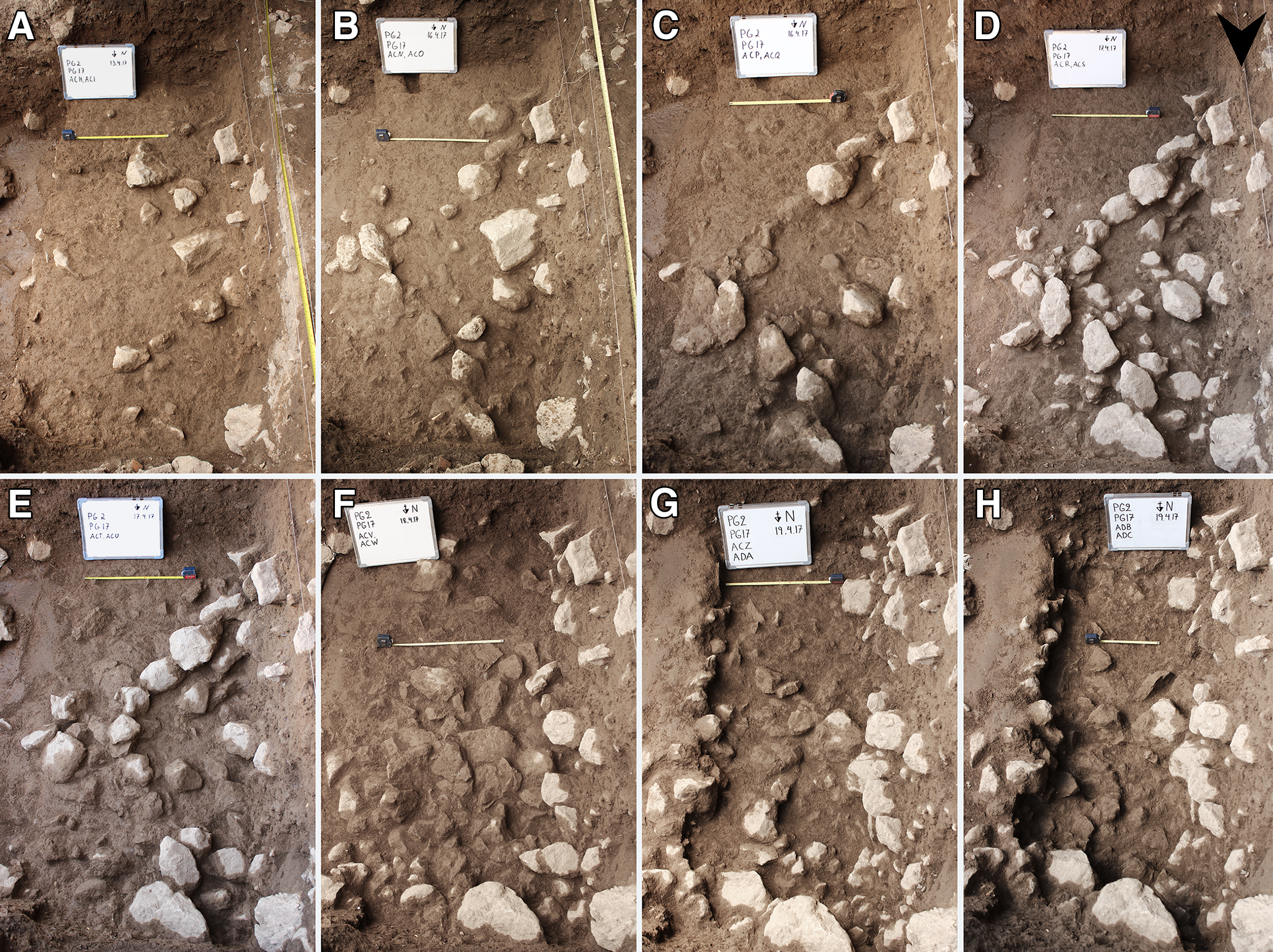

Supplement: S4 Fig — (A-C): Phase 1 upper; (D-G) Phase 1 lower; (H) basal fill (Scale: 0.50m except for H: scale 0.30m). (TIF) [file pone.0239564.s023.tif]

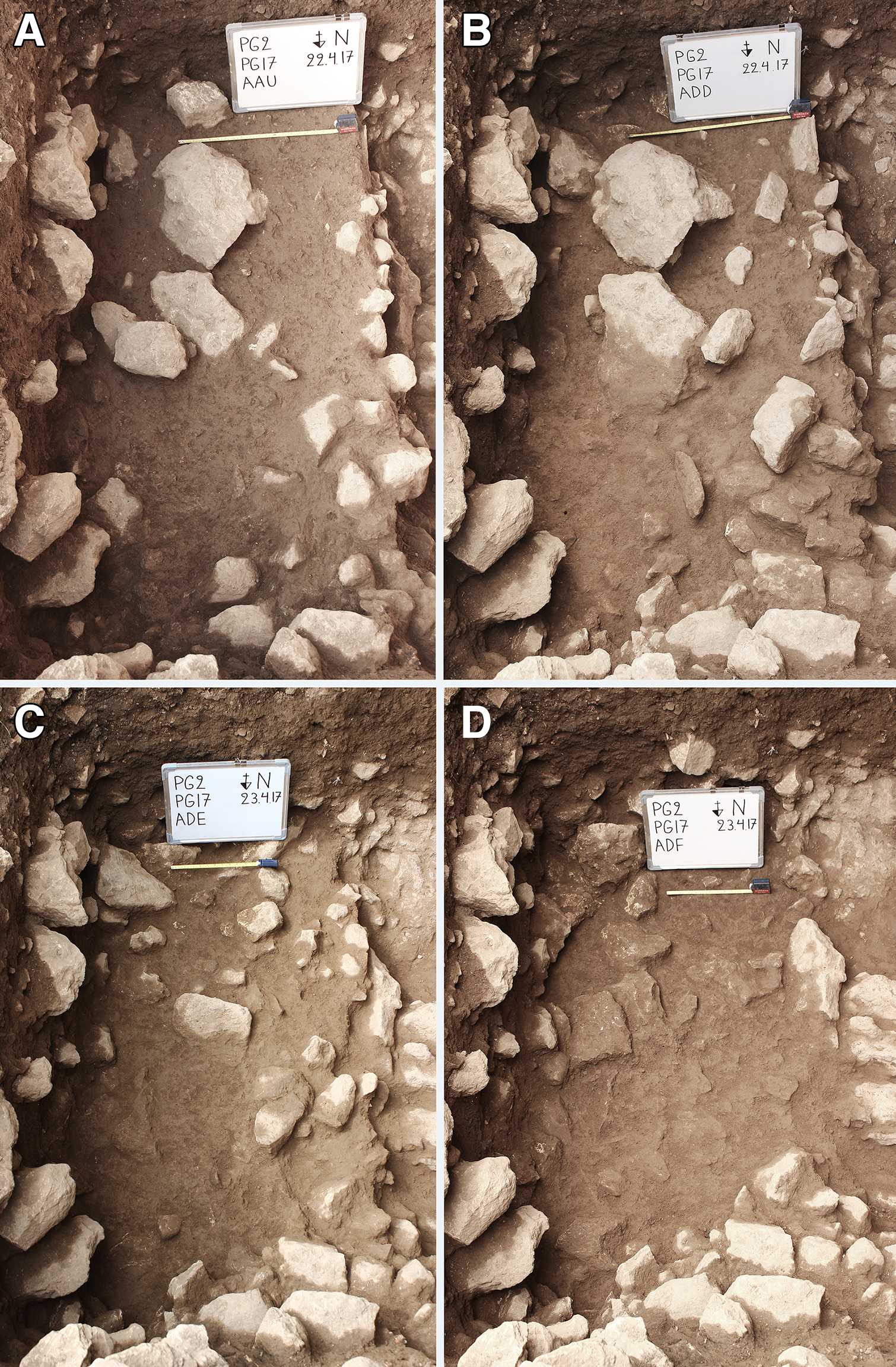

Supplement: S5 Fig — (A-C): Phase 1 lower; (D) basal fill (A-B scale: 0.50m; C-D scale: 0.30m). (TIF) [file pone.0239564.s024.tif]

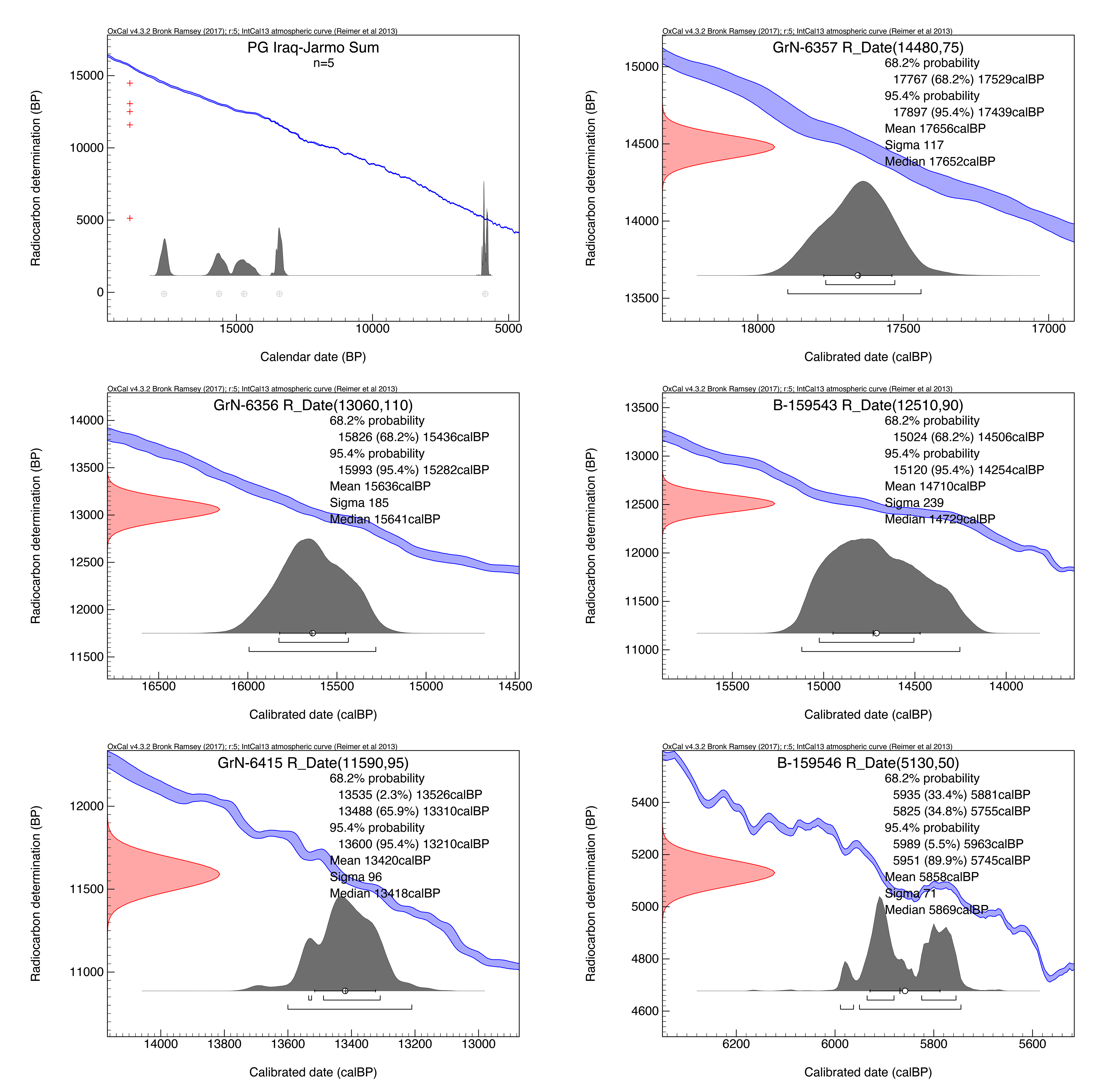

Supplement: S6 Fig — (TIF) [file pone.0239564.s025.tif]

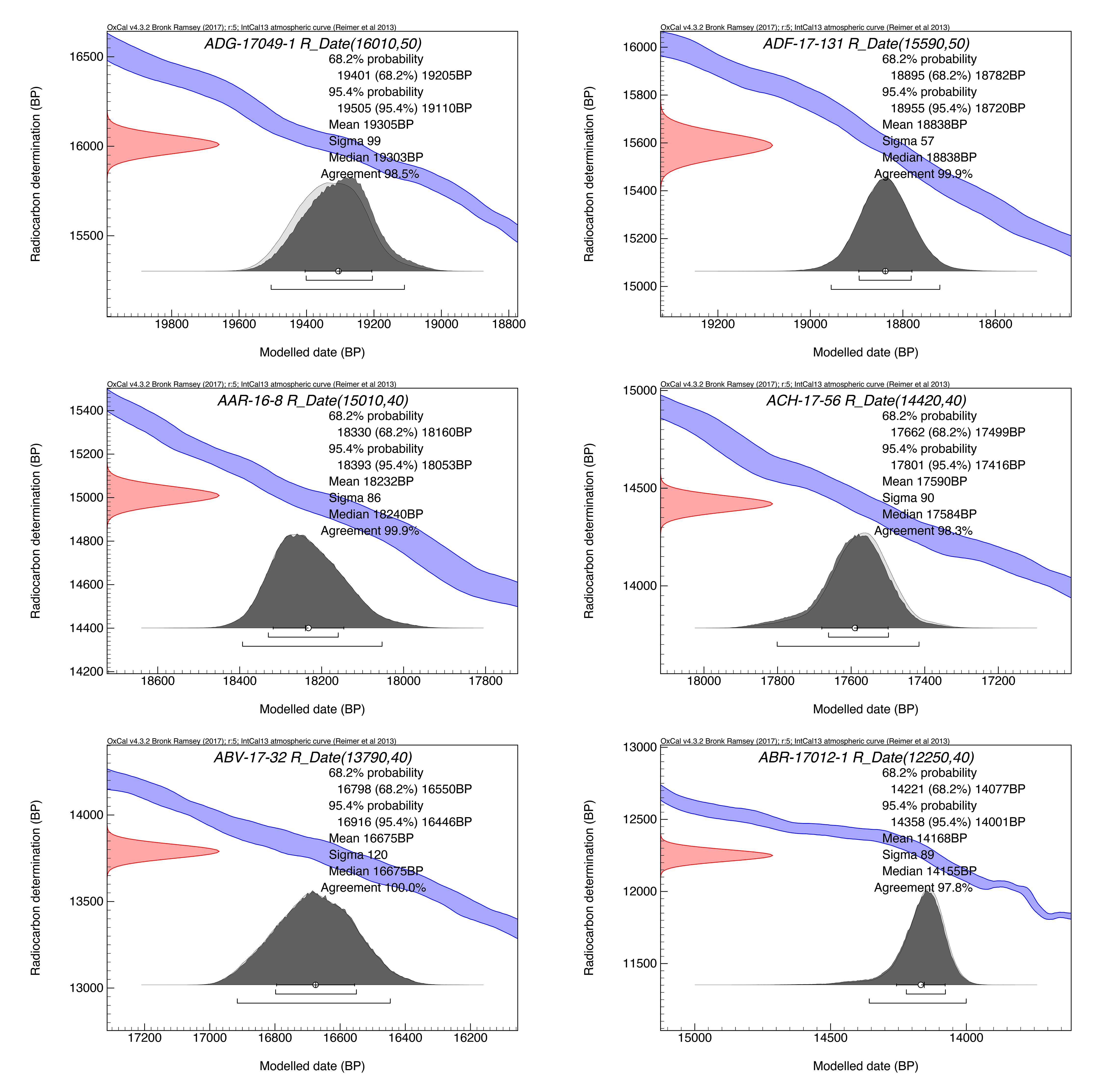

Supplement: S7 Fig — (TIF) [file pone.0239564.s026.tif]

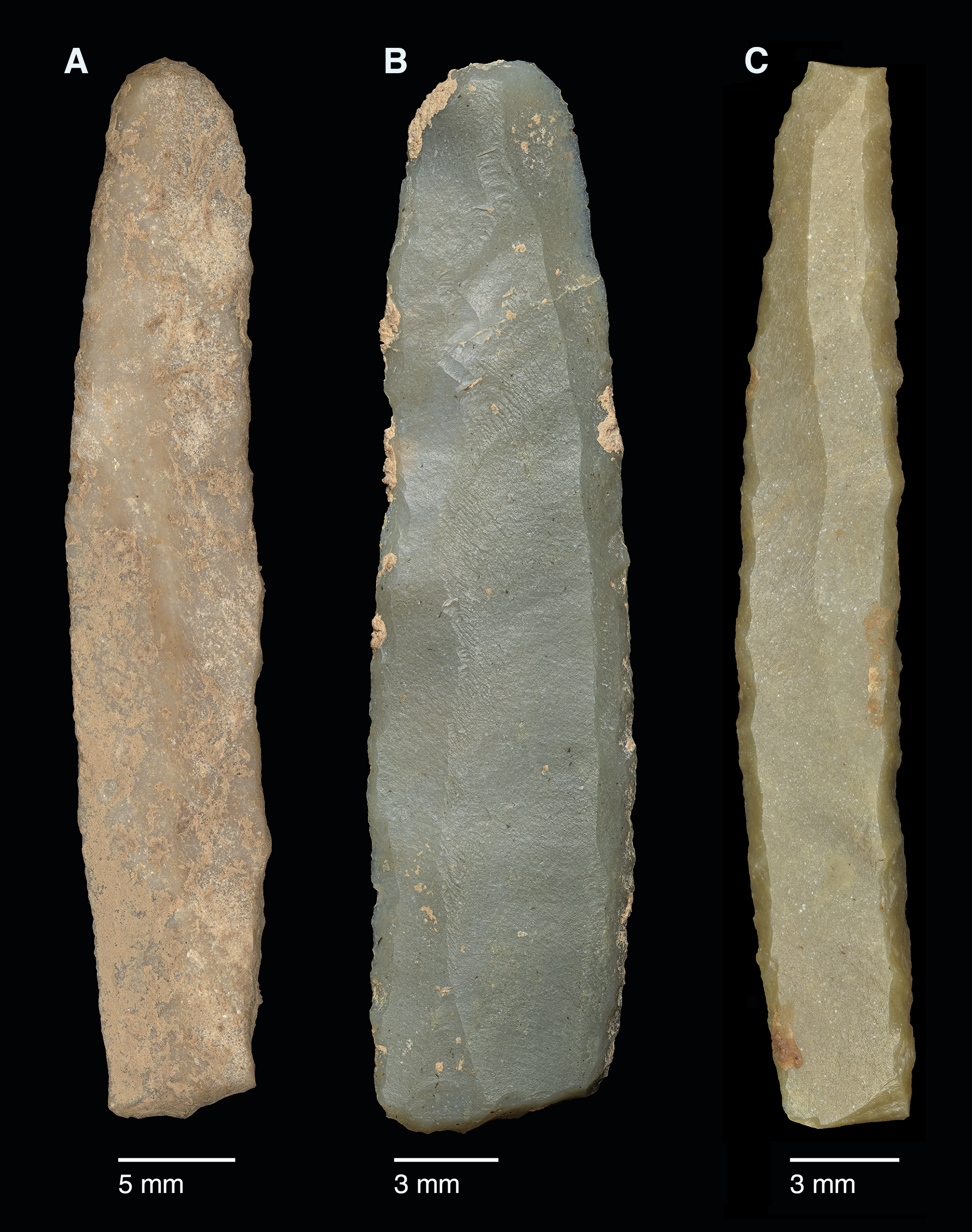

Supplement: S8 Fig — (A) backed and obliquely truncated plus arched truncation (Small Find no.107/context ACS); (B) backed and obliquely truncated (SF126/ACT); (C) backed bladelet (SF180/ADG) (images displayed in S8–S13 Figs were captured using a Keyence VX7100 4K ultra-high accuracy digital microscope at x50 magnification). (TIF) [file pone.0239564.s027.tif]

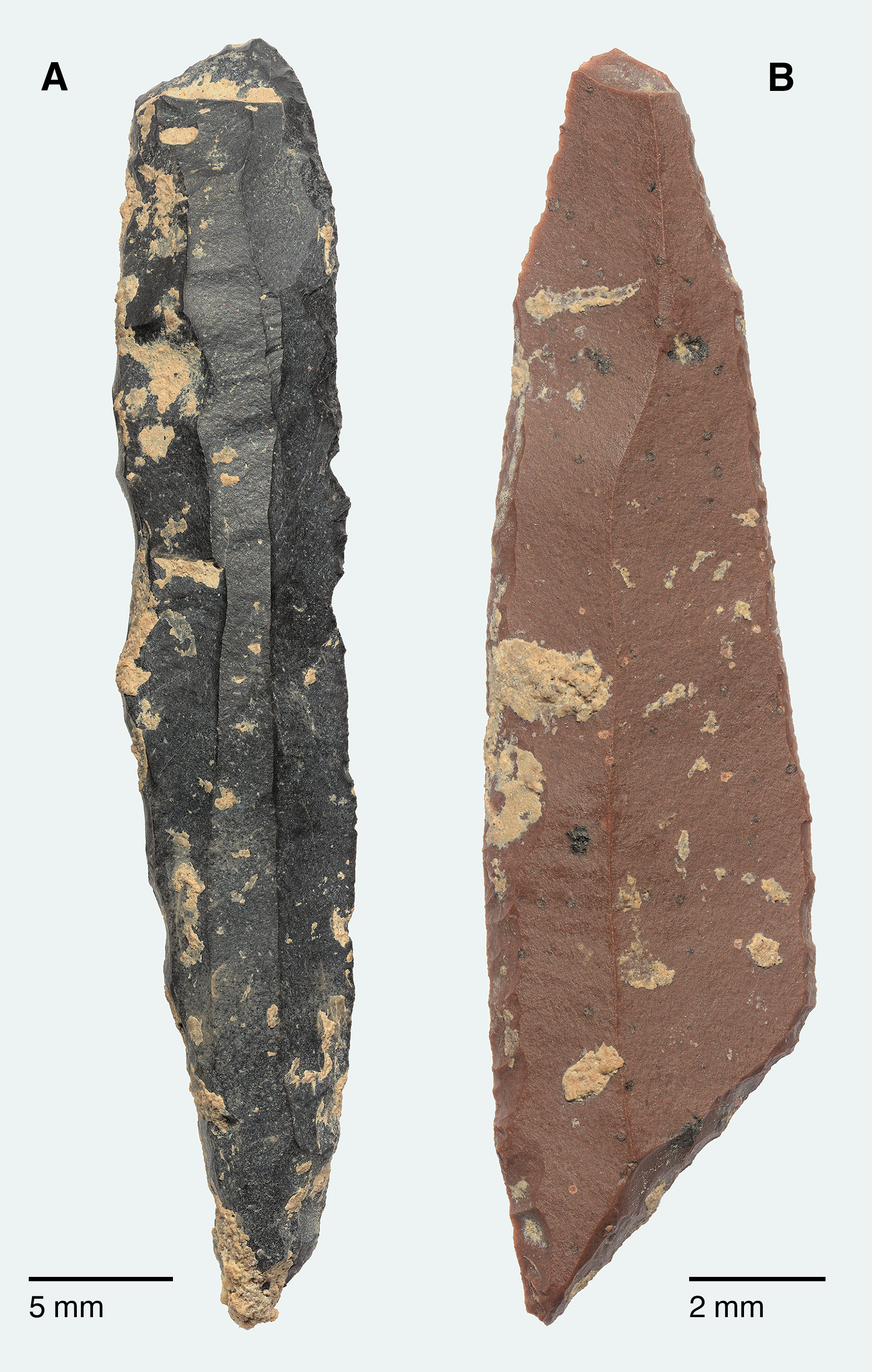

Supplement: S9 Fig — (A) backed piercer (SF41/ABY); (B) backed and obliquely blunted (SF29/ABT). (TIF) [file pone.0239564.s028.tif]

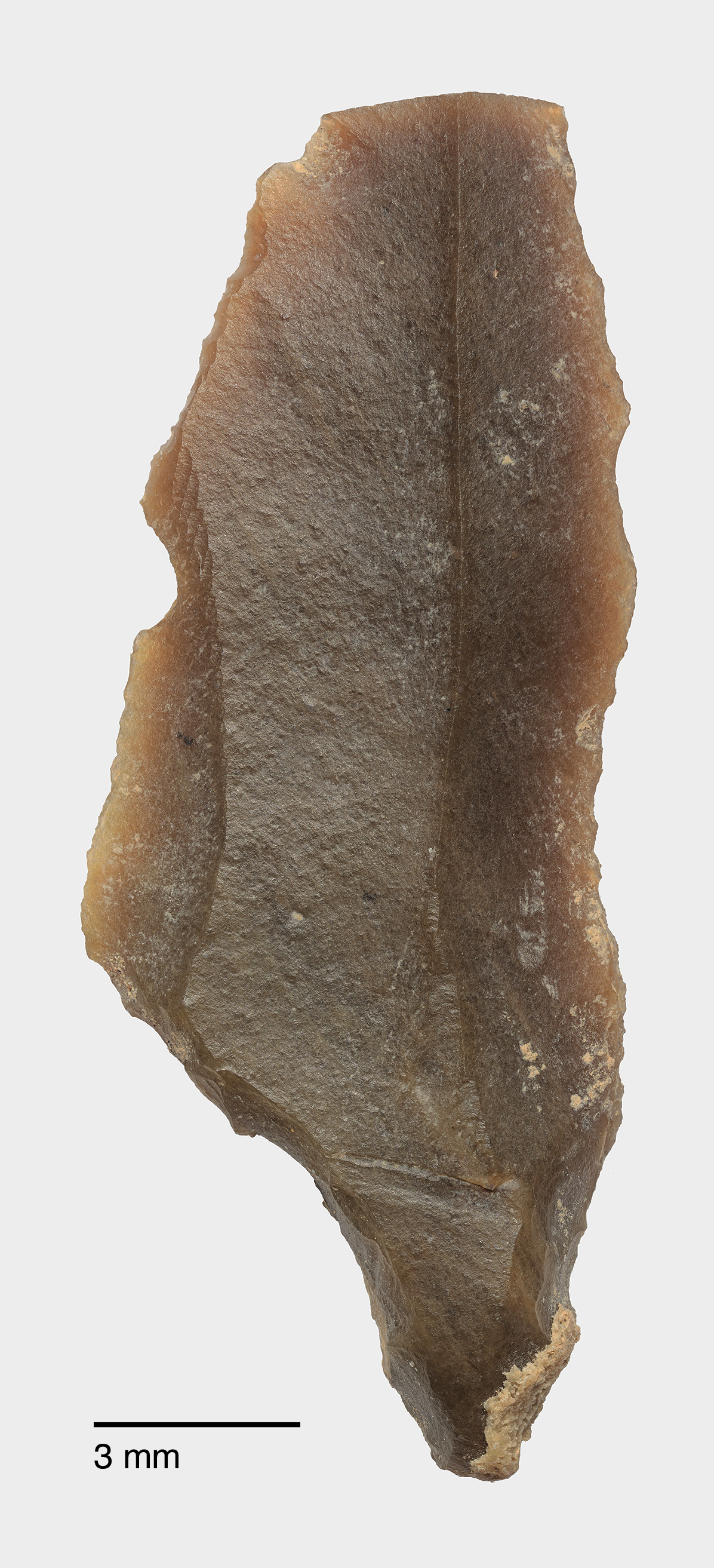

Supplement: S10 Fig — (Phase 2-SF51/ACF). (TIF) [file pone.0239564.s029.tif]

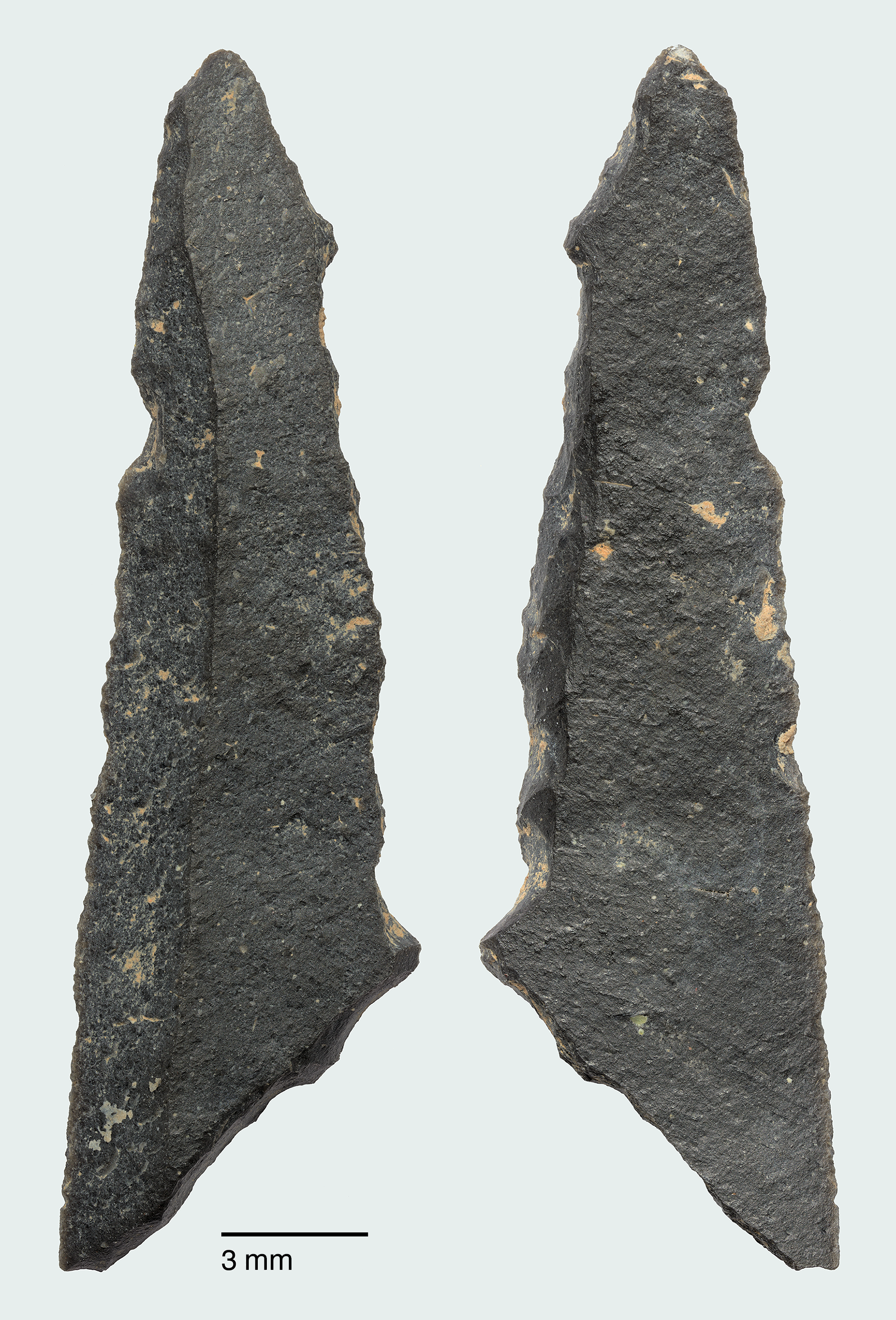

Supplement: S11 Fig — (Phase 2-SF41/ABY). (TIF) [file pone.0239564.s030.tif]

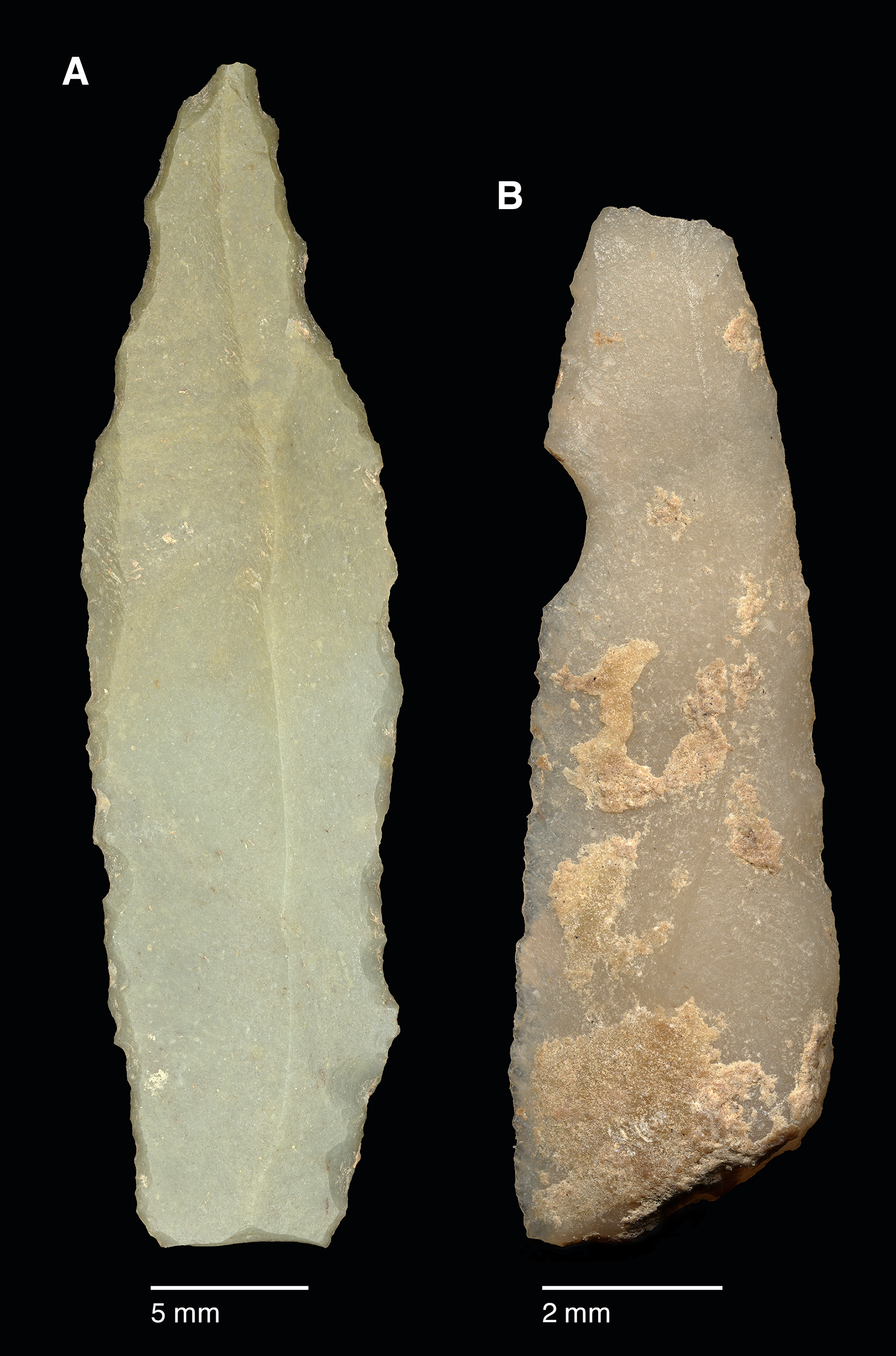

Supplement: S12 Fig — (A) arch-backed and truncated bladelet (SF31/ABT); (B) backed bladelet fragment (SF33/ABV). (TIF) [file pone.0239564.s031.tif]

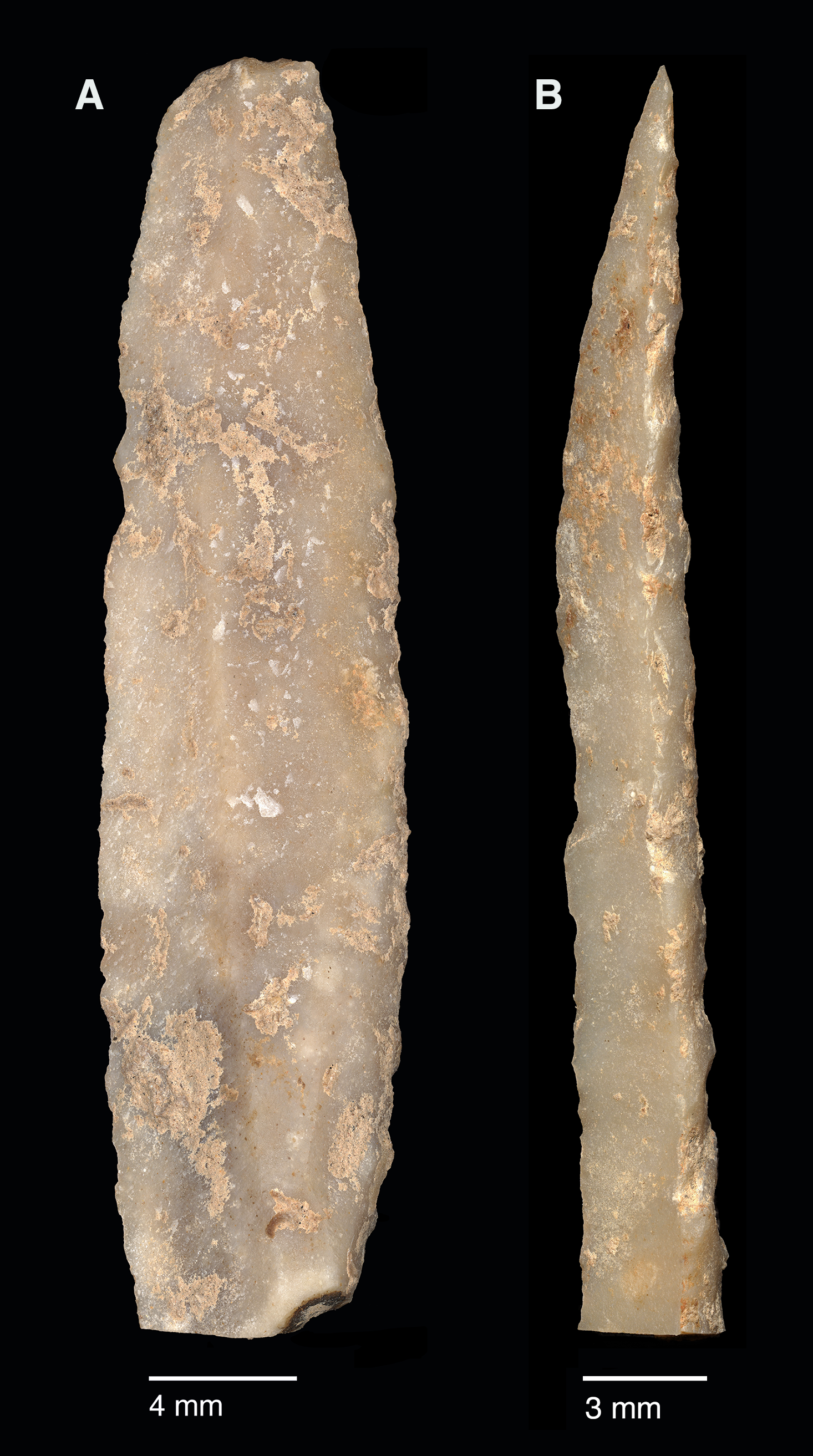

Supplement: S13 Fig — (A) piercer (ABY/Flotation sample register no. 16011); (B) backed and obliquely truncated (ABG/Flot. sample reg. no. 17009). (TIF) [file pone.0239564.s032.tif]

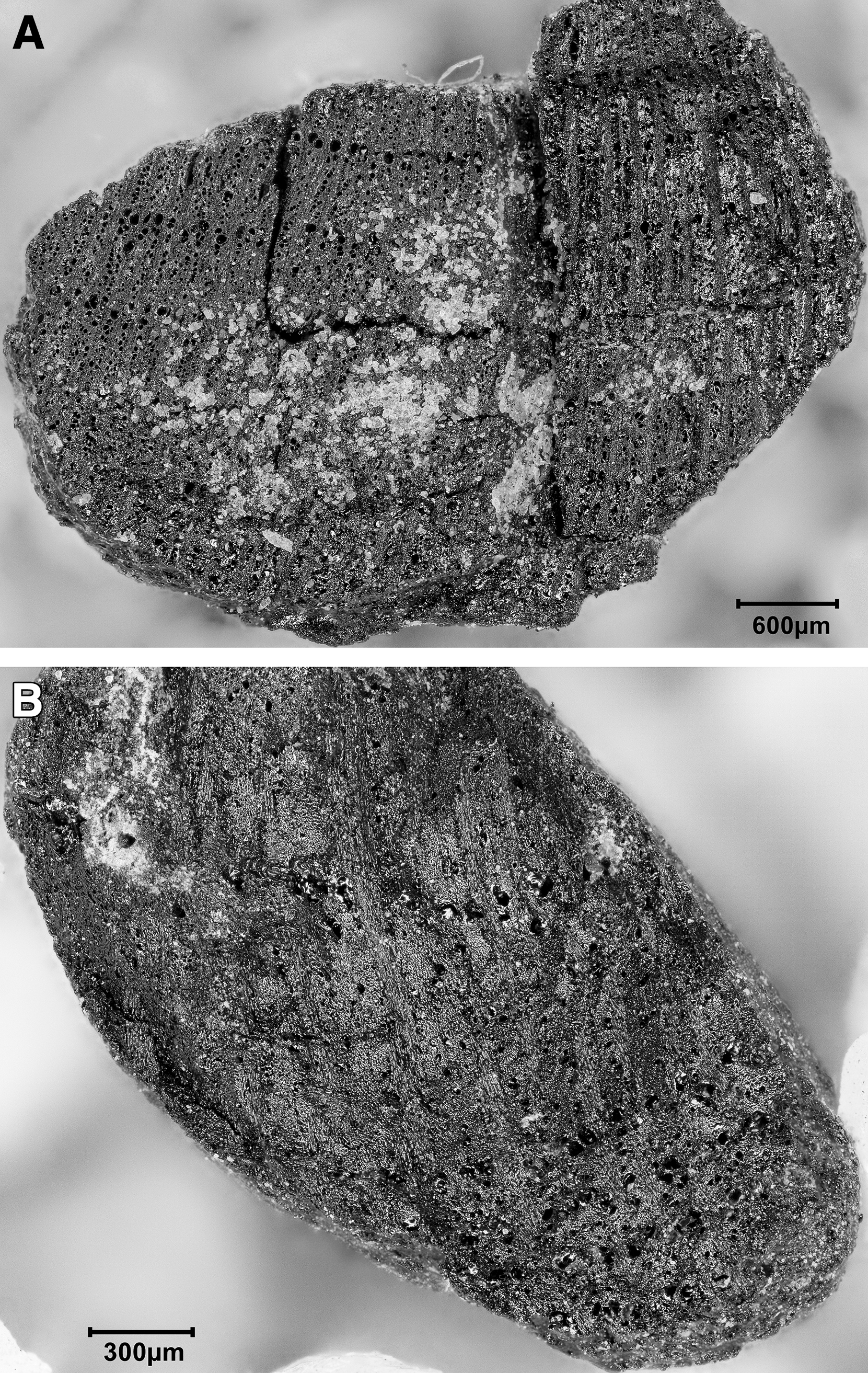

Supplement: S14 Fig — (A-B) ACZ/Flot sample register no. 17042. (TIF) [file pone.0239564.s033.tif]

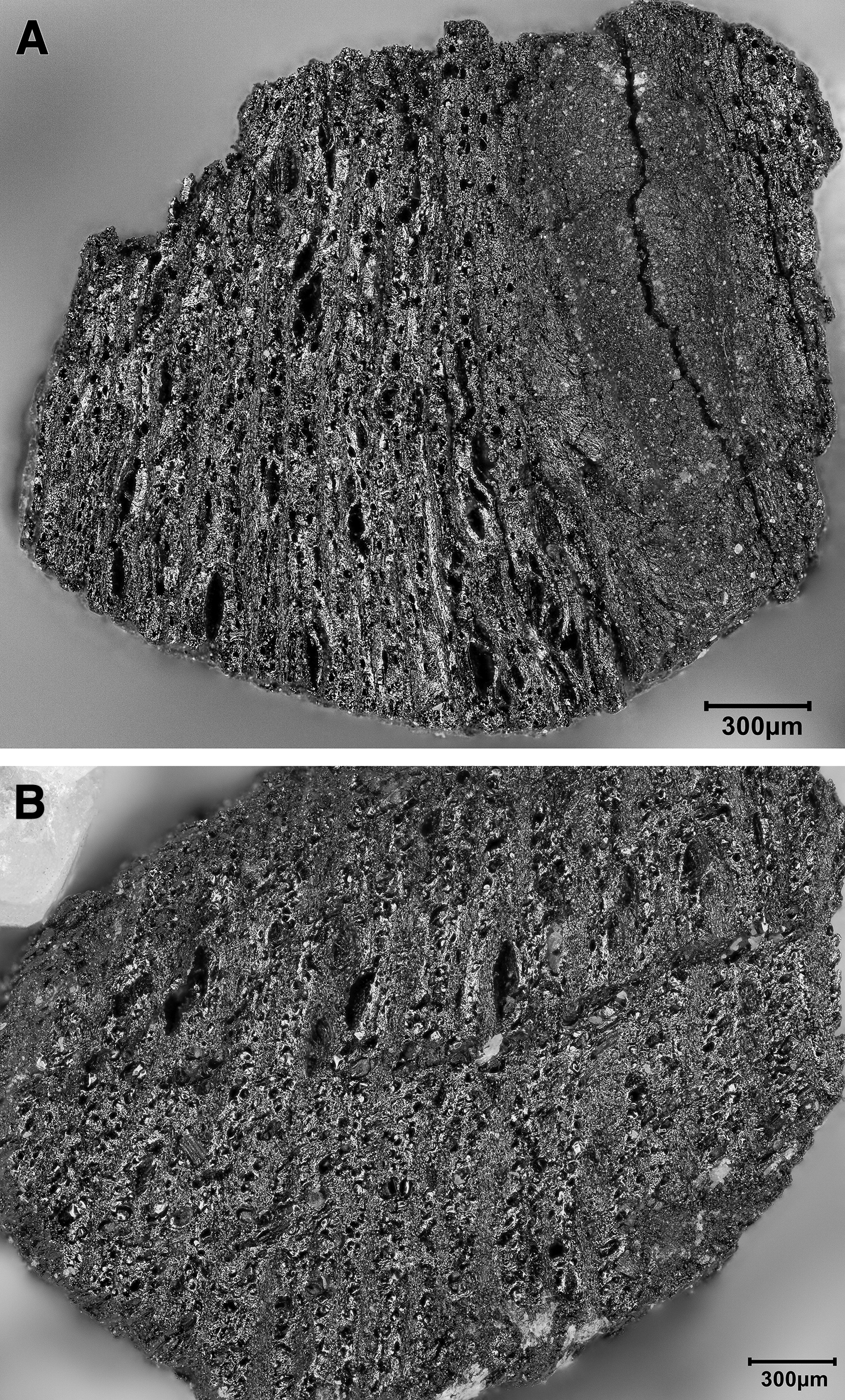

Supplement: S15 Fig — (A) ADA/17043; (B) ACX/17039. (TIF) [file pone.0239564.s034.tif]

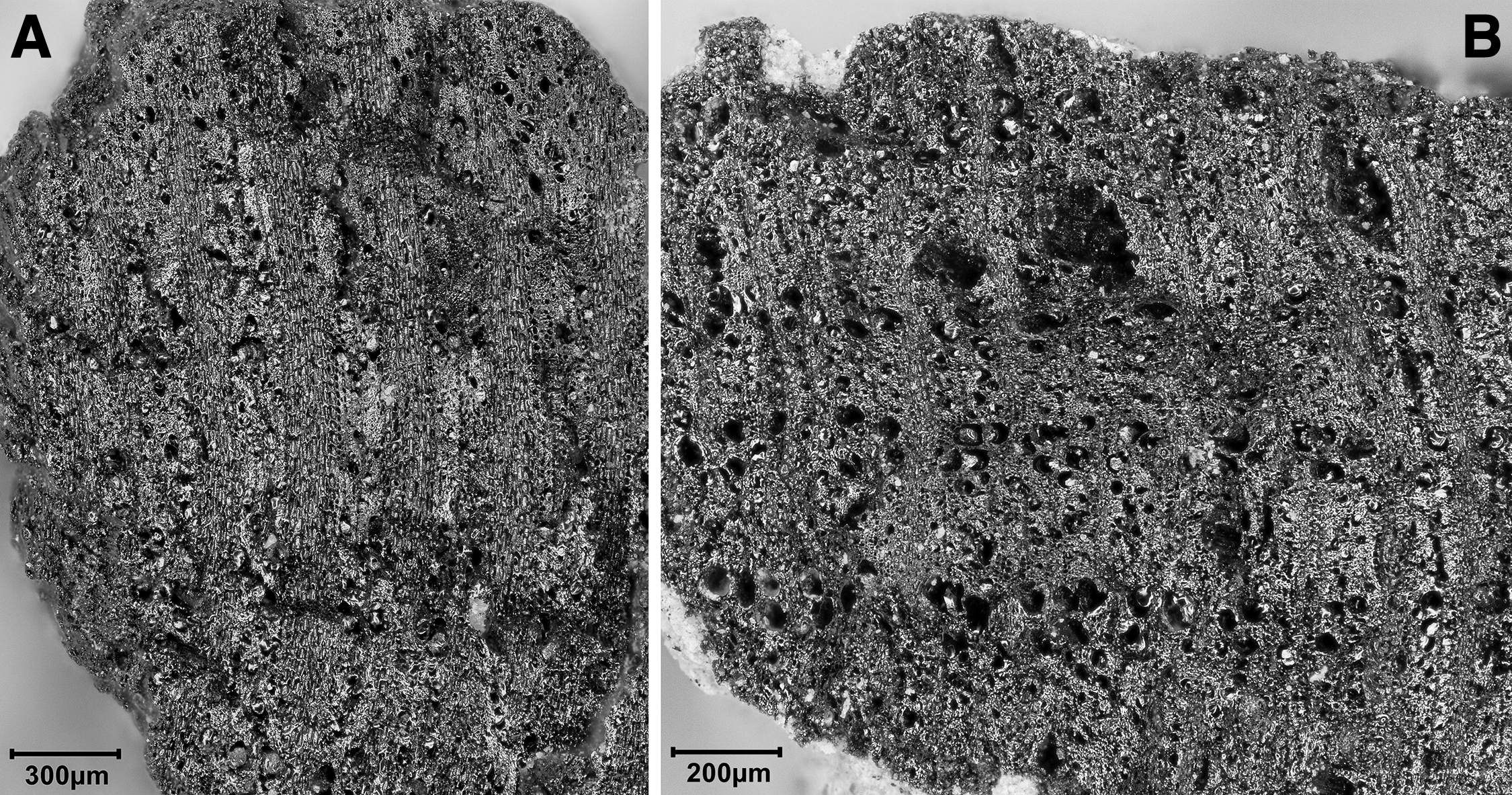

Supplement: S16 Fig — (A) ADD/17045; (B) ACV/17037. (TIF) [file pone.0239564.s035.tif]
